# Radical and Ionic Mechanisms in Rearrangements of *o*-Tolyl Aryl Ethers and Amines Initiated by the Grubbs–Stoltz Reagent, Et_3_SiH/KO*^t^*Bu

**DOI:** 10.3390/molecules26226879

**Published:** 2021-11-15

**Authors:** Krystian Kolodziejczak, Alexander J. Stewart, Tell Tuttle, John A. Murphy

**Affiliations:** Department of Pure and Applied Chemistry, University of Strathclyde, 295 Cathedral Street, Glasgow G1 1XL, UK; Krystian.Kolodziejczak@strath.ac.uk (K.K.); Alexander.Stewart.2015@uni.strath.ac.uk (A.J.S.)

**Keywords:** Truce–Smiles rearrangement, Grubbs–Stoltz reagent, radical, electron transfer, aryl substitution, diarylmethanes, dihydroacridines, triethylsilane, potassium *tert-*butoxide, DFT, carbanion

## Abstract

Rearrangements of *o-*tolyl aryl ethers, amines, and sulfides with the Grubbs–Stoltz reagent (Et_3_SiH + KO*^t^*Bu) were recently announced, in which the ethers were converted to *o*-hydroxydiarylmethanes, while the (*o-*tol)(Ar)NH amines were transformed into dihydroacridines. Radical mechanisms were proposed, based on prior evidence for triethylsilyl radicals in this reagent system. A detailed computational investigation of the rearrangements of the aryl tolyl ethers now instead supports an anionic Truce–Smiles rearrangement, where the initial benzyl anion can be formed by either of two pathways: (i) direct deprotonation of the tolyl methyl group under basic conditions or (ii) electron transfer to an initially formed benzyl radical. By contrast, the rearrangements of *o-*tolyl aryl amines depend on the nature of the amine. Secondary amines undergo deprotonation of the N-H followed by a radical rearrangement, to form dihydroacridines, while tertiary amines form both dihydroacridines and diarylmethanes through radical and/or anionic pathways. Overall, this study highlights the competition between the reactive intermediates formed by the Et_3_SiH/KO*^t^*Bu system.

## 1. Introduction

**Scheme 1:**
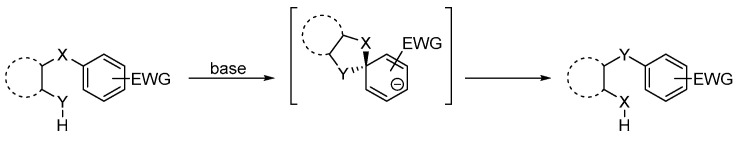
General representation of a Smiles rearrangement [9].

The first aryl migration reaction was published by Wieland in 1911 [1]. Since then, many studies have graced the literature, presenting synthetically useful transformations [2,3]. The Smiles rearrangement, discovered in 1930 [4,5], an intramolecular S_N_Ar reaction taking place at the ipso position of a substituted aromatic system, is an example of an aryl migration under basic conditions (Scheme 1) [6,7]. This S_N_Ar reaction, like the vicarious nucleophilic substitution, investigated by the team of Mąkosza [8], often features activation using electron-withdrawing groups, usually a nitro group [6,7,9,10,11,12]. However, the first identification of this rearrangement by Smiles was on naphthalene derivatives which lacked activating groups [5].

The Truce–Smiles rearrangement, discovered in 1958, is a derivative of the Smiles rearrangement in which the attacking atom is a carbanion [7]. The rearrangement of unactivated substrates in the Smiles rearrangements requires the use of strong bases and forcing conditions [13,14,15,16]. The Truce–Smiles rearrangement does not require the use of activating groups in the migrating aryl group, however, forcing conditions are still necessary for the generation of a carbanion [7,9,17]. The Smiles rearrangement, originally a two-electron process, has since then also been developed as a radical rearrangement [3,9,17,18,19,20,21,22,23,24,25,26,27]. Very recently, a series of radical cation Smiles rearrangements was reported [19] and a DFT study of the radical Smiles rearrangement has also been published [28].

**Scheme 2:**
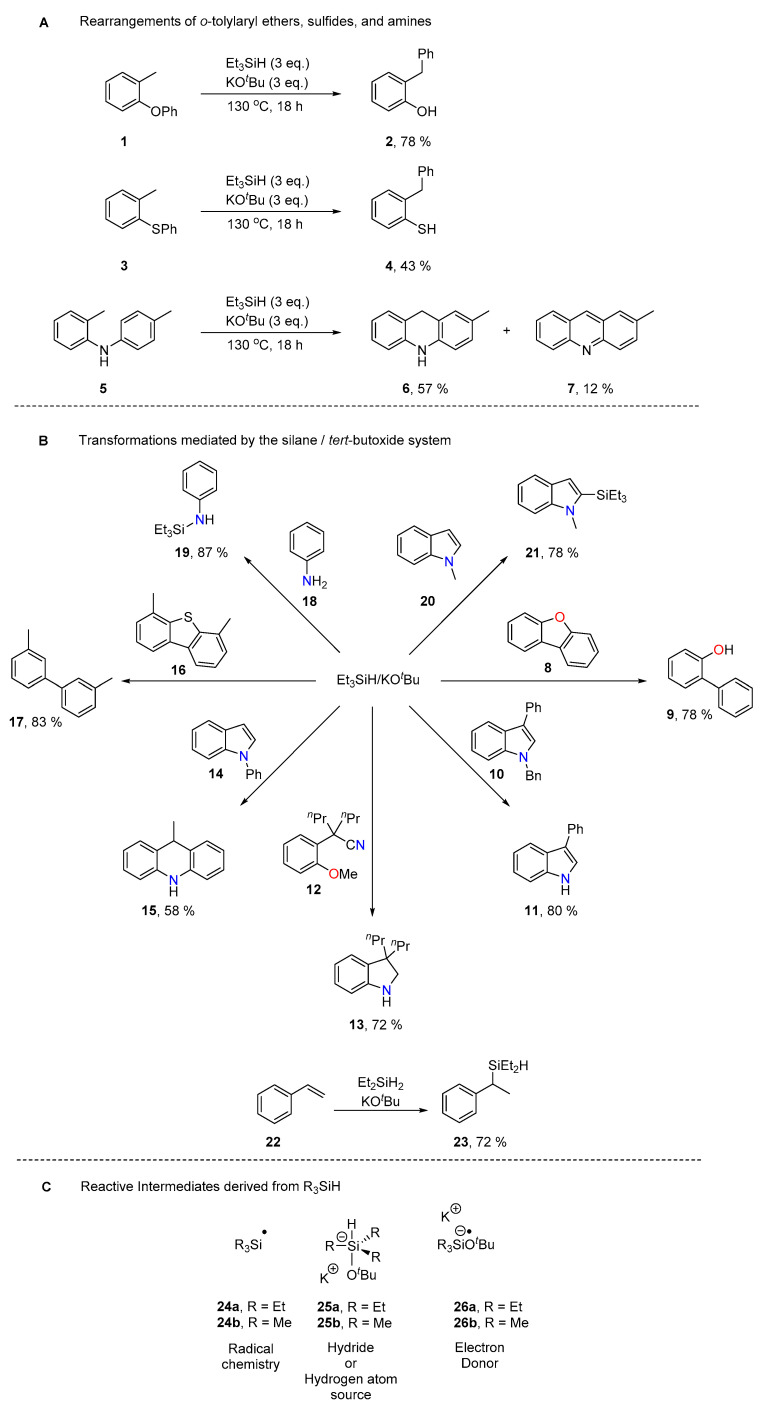
Chemistry of the Et_3_SiH/KO^t^Bu system. (**A**) Rearrangements of o-tolylaryl ethers, sulfides, and amines, (**B**) Transformations mediated by the silane/tert-butoxide system, (**C**) Reactive Intermediates derived from R3SiH.

In our recent publication [29], *o*-tolylaryl ethers **1** and sulfides **3** underwent rearrangement to diarylmethanes **2** and **4,** respectively, while *o*-tolylaryl amine **5** yielded oxidatively cyclised products **6** and **7** (Scheme 2A). The reactions were mediated by triethylsilane and potassium *tert*-butoxide. This novel reagent pair was first reported by Grubbs and Stoltz in 2013 [30]. The original discovery presented a new method for the cleavage of strong C–O bonds in aryl ethers (**8**→**9**, Scheme 2B). Since then, the reagent pair has proven to be remarkably versatile by facilitating the wide range of transformations shown in Scheme 2B. Three reaction intermediates **24a**–**26a** (Scheme 2C) are proposed to be responsible for the diverse chemistry observed [30,31,32,33,34,35,36,37,38]. Triethylsilyl radicals **24a** were previously identified by detection of a TEMPO-SiEt_3_ adduct [32]. In addition, a ReactIR study on the combination of triethylsilane and potassium *tert*-butoxide had revealed the formation of a new species in situ, suggested to be pentavalent silicate **25a**. [33]. This intermediate can be a source of a hydrogen atom or a hydride ion [33,34]. Smith et al. proposed radical anion **26a** as an intermediate in the debenzylation of *N*-benzylindoles [35]. Accordingly, substrates treated with the triethylsilane/potassium *tert-*butoxide system are subjected to radical, base, hydrogen atom transfer, hydride ion, and electron transfer chemistry simultaneously, allowing for diverse reaction outcomes and mechanisms. Following our publication [29], we decided to launch a computational and experimental study to understand the difference in reactivity between the ether and amine substrate classes. The results of this investigation are presented within this paper.

Theoretical details: DFT calculations were carried out using the M06-2X functional [39,40] with the 6-311++G(d,p) [41,42,43,44] basis set on all atoms. All calculations were performed using the C-PCM [44] implicit solvent model with parameters for triethylamine as solvent. No silane (Me_3_SiH or Et_3_SiH) solvents are parametrised in Gaussian 16, so triethylamine was chosen as the closest model to actual silane solvent since it has a similar dielectric constant (ε = 2.3832) compared to triethylsilane (ε = 2.323) [45]. All calculations were performed in Gaussian 16 [46] at 403.15 K. While experimental reactions used triethylsilane, yielding intermediates **24a**–**26a**, theoretical studies made use of the corresponding trimethylsilane-derived intermediates, **24b**–**26b** which were used for computational economy.

## 2. Results and Discussion

**Scheme 3:**
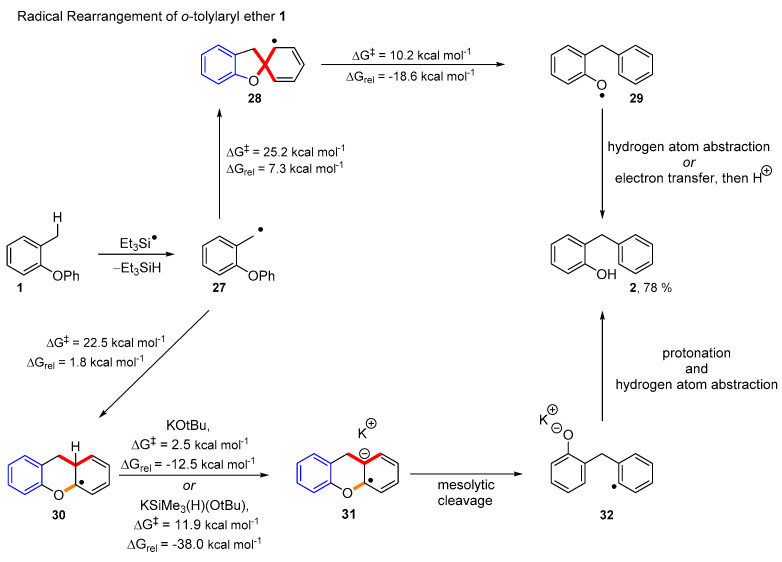
Energy barriers and relative energy changes for rearrangement of *o*-tolylaryl ether **1**.

The *o*-tolylaryl ethers, represented by **1**, were considered first (Scheme 3). Thus, after hydrogen atom abstraction by triethylsilyl radicals **24a** to form benzyl radical **27**, two possibilities for cyclisation were considered. 5-*Exo-*trig cyclisation gives the spiro intermediate **28**, which then fragments to yield phenoxyl radical **29**. This species is transformed to the isolated product **2** either by electron transfer followed by protonation, or by hydrogen atom transfer. In the alternative route, benzyl radical **27** undergoes a 6-aryl cyclisation (we prefer to refer to such cyclisations as ‘6-aryl’, since they could potentially be regarded as 6-*exo* or 6-*endo* depending on the initial Kekulé representation of the Ph group in **27**) to give cyclohexadienyl radical **30**, which likely suffers rapid deprotonation by either KO*^t^*Bu or pentavalent silicate **25b** to form radical anion **31** [47]. To proceed to product **2**, this would be followed by C–O fragmentation to give distal radical anion **32**. Hydrogen atom abstraction and protonation would then yield product **2**. Scheme 3 reports the energy changes for the two competing cyclisation steps. The 6-aryl cyclisation is favoured here, with a lower transition state for **27**→**30** (22.5 kcal mol^−1^) than for **27**→**28** (25.2 kcal mol^−1^). In addition, the formation of **30** is less endergonic (1.8 kcal mol^−1^) compared to **28** (7.3 kcal mol^−1^). Based on these results, the 6-aryl cyclisation is kinetically and thermodynamically favoured; however, the steps following either cyclisation mode towards the product are exergonic (**28**→**29**, **30**→**31**). Given these figures, and the accuracy of computational predictions (accuracy to within 2.0 kcal mol^−1^ [39]) one might expect that the 6-aryl cyclisation is favoured or that both cyclisation routes are in contention.

**Scheme 4:**
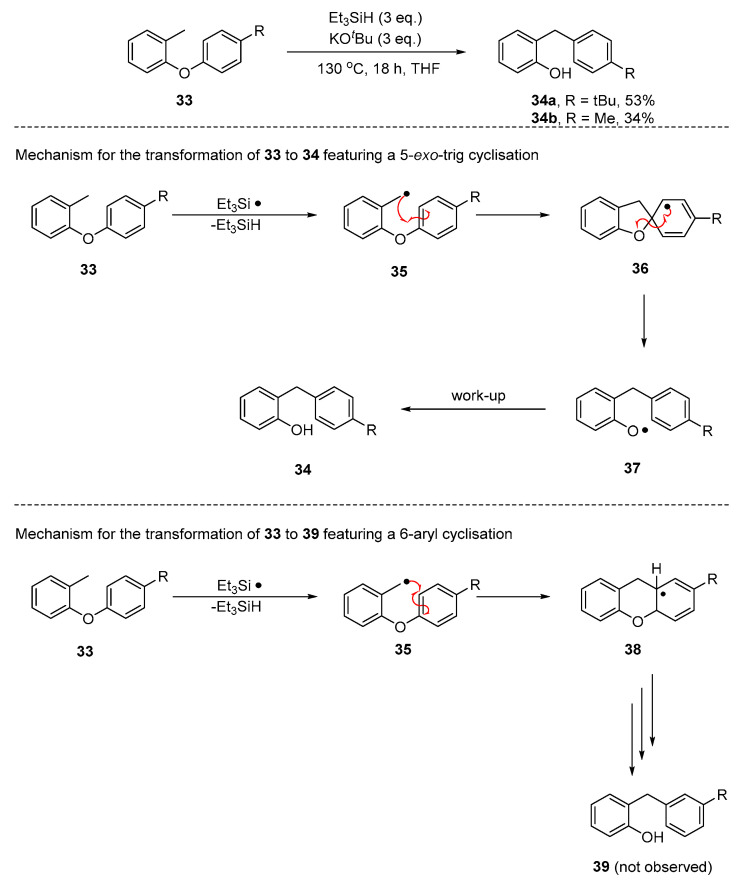
Reaction of *o*-tolylaryl ether **33** with the Et_3_SiH/KO*^t^*Bu [25].

However, our recent paper showed that the rearranged ethers must arise only by the 5-*exo* cyclisation route using substrates **33** (Scheme 4). In these cases, a different product **34** or **39**, would arise, depending on the cyclisation mode. Thus, 5-*exo* cyclisation of **35** would give spiro intermediate **36**, resulting in product **34**, where the R group is *para*- to the benzylic CH_2_. Alternatively, 6-aryl cyclisation of **35** would lead to product **39**, where the relationship is *meta*. The outcome of these experiments was that products **34** were isolated and no **39** was ever detected. Therefore, the laboratory reaction must proceed through a 5-*exo* cyclisation. The discrepancy between this result and the energy-based predictions of Scheme 3 suggested that further mechanistic possibilities should be considered.

The simplest change to consider was how the competition between the two modes of cyclisation would be affected by complexation to a potassium cation. Experiments and computational evidence testify to the important role that π-cation complexes involving K^+^ ions can have on organic reactions of aryl substrates [37,48,49,50,51,52].

**Scheme 5:**
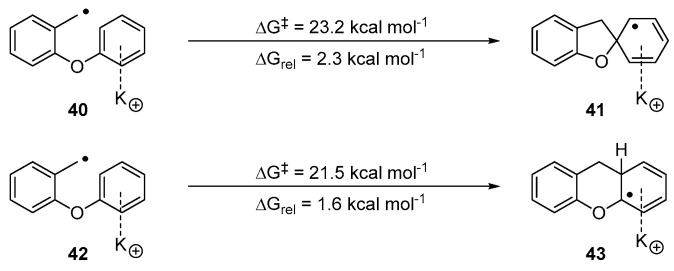
Probing potential cation-π interactions.

Scheme 5 indicates that the presence of a potassium cation does affect the energy profile of the reactions, but with the numbers still favouring the 6-aryl case (**42**→**43**) slightly over 5-*exo* (**40**→**41**). Thus, the computational results did not reflect the experimental results.

**Scheme 6:**
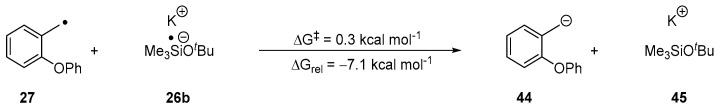
SET reduction of benzylic radical **27** to benzylic anion **44**.

Accordingly, the investigation of the mechanism was extended to anionic intermediates, and pathways to access benzyl anions were considered. As mentioned above, radical anion **26a** has been proposed as an intermediate that is formed from heating triethylsilane and KO*^t^*Bu. According to our computational studies, **26b** (without a potassium counterion) is an extremely strong electron donor [31,53], with *E*_ox_ = −3.74 V vs. SCE (MeCN). Therefore, it would be capable of reducing an intermediate benzylic radical [*E*_red_ = −1.43 V vs. SCE (MeCN)] [54] to an anion. This single electron transfer (SET) was probed using the Nelsen Four-Point method [55] (Scheme 6).

The reduction of benzyl radical **27** was almost barrierless with an activation energy of 0.3 kcal mol^−1^. The reduction was also exergonic and, so, it is likely to happen in situ prior to the cyclisation. Therefore, it was appropriate to explore the energy profiles for cyclisations of benzyl anions.

**Scheme 7:**
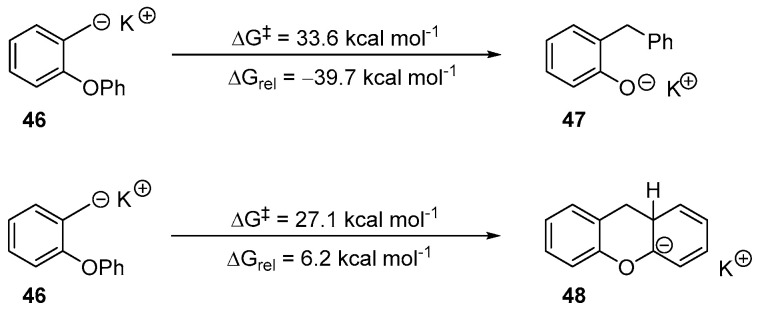
Examination of the two cyclisation modes available to salt **46**.

Anion **44** is likely to complex with a potassium cation in situ to form a salt, **46**. This salt was used to investigate the two cyclisation modes available to it (Scheme 7).

Thermodynamics predict that the 5-*exo*-trig cyclisation [ΔG_rel =_ −39.7 kcal mol^−1^] leading to **47** is favoured over the 6-aryl cyclisation [ΔG_rel_ = +6.2 kcal mol^−1^] that would lead to **48**. The activation energy for the 5-*exo*-trig pathway (ΔG^‡^ = 33.6 kcal mol^−1^) is achievable under the conditions of the reaction. The 6-aryl cyclisation does possess a lower activation energy favouring it as the kinetic cyclisation. However, it is also endergonic by 6.2 kcal mol^−1^. The experimental results have already shown that this cyclisation mode is not featured in the products (Scheme 3). Therefore, if it occurs, intermediate **48** reverts to intermediate **46** which undergoes an irreversible 5-*exo*-trig cyclisation.

Our computations also show that this Truce–Smiles rearrangement from **46** to **47** is concerted. Generally, Smiles and Truce–Smiles rearrangements proceed through the formation of a spiro intermediate, which subsequently undergoes a ring-opening to yield the product [6,17]. Mechanisms featuring a spiro transition state rather than a spiro intermediate have been proposed [7], with several examples being identified by Clayden et al. for the Smiles and Truce–Smiles rearrangements [56,57,58,59,60,61,62]. Concerted reaction pathways have also been identified for other Smiles-type rearrangements [63,64,65]. In our case, the conversion of salt **46** to **47** presents a new example of a concerted Truce–Smiles rearrangement. This was confirmed through an intrinsic reaction coordinate (IRC) calculation which displayed the simultaneous contraction of the C–C bond being formed and the elongation C–O bond being broken. Attempts to optimise a spiro intermediate all resulted in the ring-opening of the dihydrofuran ring yielding salt **47**.

**Scheme 8:**
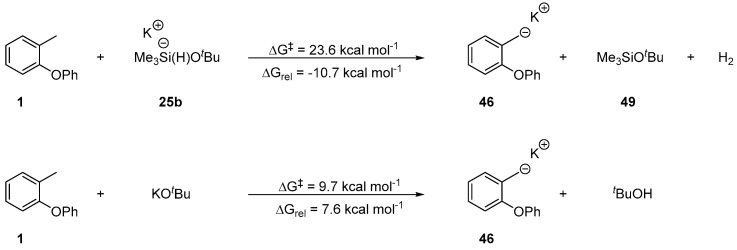
Anionic rearrangement route for **1** utilising pentavalent silicate **25b** or KO*^t^*Bu as the active base.

The formation of anion **46** through a radical-polar crossover from radical **27** however, is not the only possible route for formation of **46**, and so we considered the direct deprotonation of the methyl group of aryl ether **1** by either pentavalent silicate **25** or KO*^t^*Bu (Scheme 8). The initiation route featuring pentavalent silicate **25b** was found to be exergonic (ΔG_rel =_ −10.7 kcal mol^−1^) with an attainable activation energy (ΔG^‡^ = 23.6 kcal mol^−1^), making it a competitive initiation route with the radical-polar crossover pathway. KO*^t^*Bu was also identified as a base potentially capable of initiating the reaction; the deprotonation has a surmountable barrier, but it is accompanied by an endergonic change in energy disfavouring the formation of salt **46**. The following concerted Truce–Smiles rearrangement is sufficiently exergonic to push the reaction forward. Hence, a control experiment using only KO*^t^*Bu and aryl ether **1** was carried out to probe this possibility. However, the reaction did not yield the product and only starting material was recovered. This result rules out KO*^t^*Bu as a base capable of initiating the reaction via direct deprotonation of the methyl group of the *o*-tolyl ring. Complete reaction coordinate diagrams utilising both initiation routes are shown in the SI (Figures S3 and S4).

In summary, *o*-tolyl aryl ethers were identified to yield *o*-hydroxydiarylmethanes through a concerted Truce–Smiles rearrangement. The *o*-tolyl anion responsible for the rearrangement is generated through two competitive pathways: (i) a radical polar crossover route featuring a SET reduction of the initial benzylic radical formed via hydrogen atom abstraction by a triethylsilyl radical **24a** (Scheme 6), (ii) the direct deprotonation of the *o*-tolyl methyl group by pentavalent silicate **25a** (Scheme 8). The carbanion generated by these initiation pathways has the option to undergo either a 5-*exo*-trig cyclisation or a 6-aryl cyclisation. The former cyclisation mode is supported by experimental evidence from the treatment of strategically substituted aryl ether **33** (Scheme 4). Computationally, the 6-aryl cyclisation was established to be kinetically favoured over the 5-*exo*-trig cyclisation. However, the 6-aryl cyclisation was ruled out based on experimental evidence (Scheme 4). This is compatible with an endergonic and reversible 6-aryl cyclisation, which ultimately results in the carbanion undergoing the thermodynamically favoured irreversible 5-*exo* trig cyclisation (Scheme 7). 

**Scheme 9:**
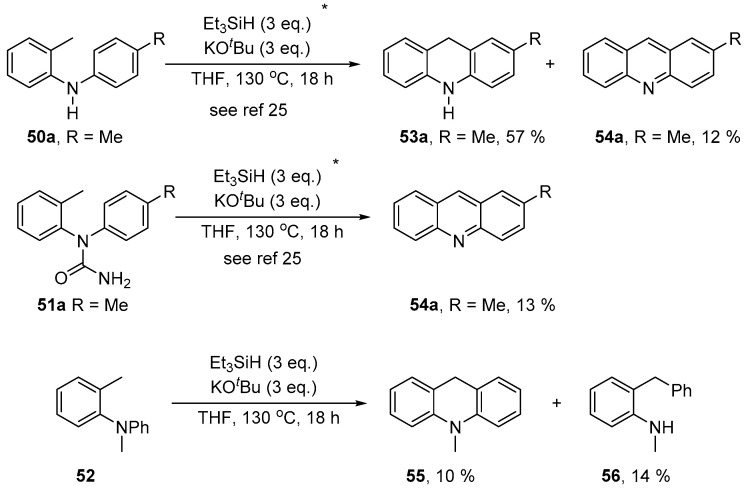
Treatment of *o*-tolyl aryl amines with the Et_3_SiH/KO*^t^*Bu system.

Nitrogen series: Our recent experimental findings showed that the structure of the *o*-tolyl amine substrate governs which type(s) of product are formed. When the starting amine is secondary (**50a**) or when it has a group bonded to the nitrogen, as in **51a**, which is cleavable under the reaction conditions, only acridine-type products are formed (Scheme 9). To test what happens when the starting amine is tertiary, the *N*-methyl amine **52** was prepared and subjected to the reaction conditions. This yielded both dihydroacridine **55** (10%) and diarylmethane **56** (14%).

**Scheme 10:**
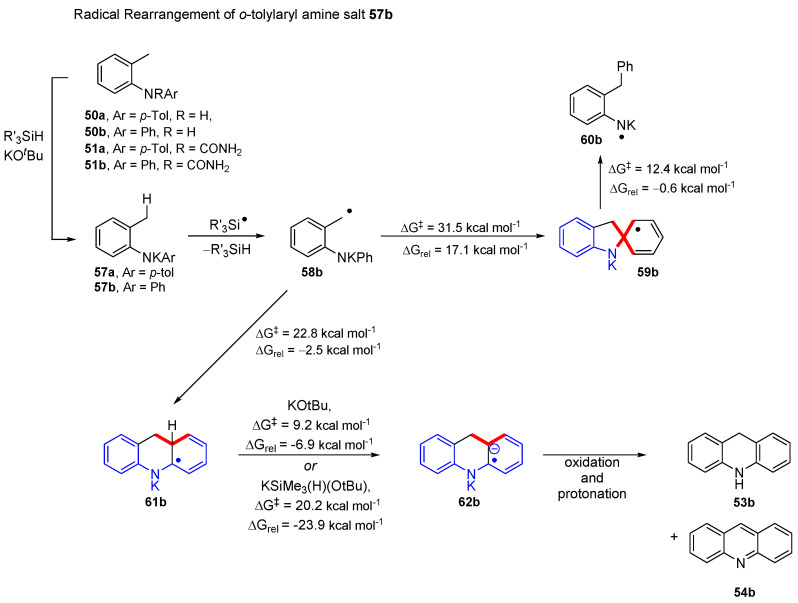
Energy barriers and relative changes in energy for rearrangement of *o*-tolylaryl amine salts **57**.

Substrates **50a** and **51a** will be considered first. Under strongly basic conditions, they are converted to potassium salt **57a**. A radical mechanism for the transformation of salt **57** was initially probed (Scheme 10). For computational economy, the simpler case **57b**, derived from **50b** and **51b** was explored. Benzyl radical **58b**, formed via hydrogen atom abstraction by a trimethylsilyl radical (ΔG^‡^ = 19.4 kcal mol^−1^; ΔG_rel_ = 0.9 kcal mol^−1^) could undergo either a 5-*exo*-trig cyclisation to **59b** or a 6-aryl cyclisation to **61b**. The latter is preferred, having a lower activation (ΔG^‡^ = 22.8 kcal mol^−1^) and a favourable change in Gibbs free energy (ΔG_rel_ = −2.5 kcal mol^−1^) versus the 5-*exo*-trig cyclisation mode (ΔG^‡^ = 31.5 kcal mol^−1^, (ΔG_rel_ = 11.7 kcal mol^−1^). The 6-aryl cyclisation intermediate **61b** is subsequently deprotonated by either pentavalent silicate **25a** or KO*^t^*Bu, yielding the corresponding radical anion **62b**. Oxidation and protonation of **62** on workup yields dihydroacridine **53b** which can be further oxidised by air during purification to yield acridine **54b**.

**Scheme 11:**
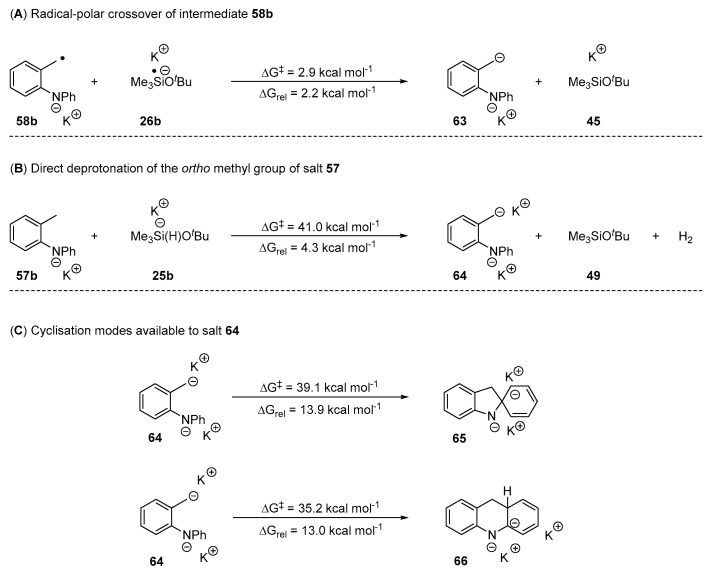
(**A**) Investigation of a radical-polar crossover of benzylic radical **58b** to anion **63** via SET from silyl radical anion **26b**. (**B**) Investigation into the direct deprotonation of the ortho methyl group of salt **57b** by pentavalent silicate **25b**. (**C**) Investigation of the two cyclisation modes available to salt **64**.

Having studied the behaviour of the benzyl radicals, the next stage was to study the corresponding benzyl anions. These might be formed by: (i) reduction of the initially formed benzyl radical to a benzyl anion by single electron transfer and (ii) formation of the benzyl anion by direct deprotonation of the methyl group of the *o*-tolyl ring. Both of these routes were also now investigated for salt **57b** (Scheme 11).

The conversion of **58b** to **63** by electron transfer (Nelsen Four-Point method) [55] was endergonic (Scheme 11A). In solution, the product anion might be rapidly stabilised by complexation with a potassium cation to form **64** (for an analogous stabilisation, see Figure S14). The alternative route to the benzyl anion **64** utilising pentavalent silicate **25b** as base, was also found to be unproductive, as the activation energy (ΔG^‡^ = 41.0 kcal mol^−1^) exceeded the attainable limit at 130 °C. Assuming **64** was formed by the electron transfer route, its cyclisation by 5-*exo*-trig or 6-aryl cyclisation was not feasible due to the high activation barriers in both cases (Scheme 11C); this rules out an anionic cyclisation mechanism for *o*-tolylaryl amines that are converted to the analogous potassium salt **57** under the reaction conditions. Therefore, *o*-tolyl aryl amines which yield the corresponding amide salt in situ prior to the rearrangement proceed through a radical mechanism by 6-aryl cyclisation to yield the observed acridine-type products (Scheme 10).

**Figure 1:**
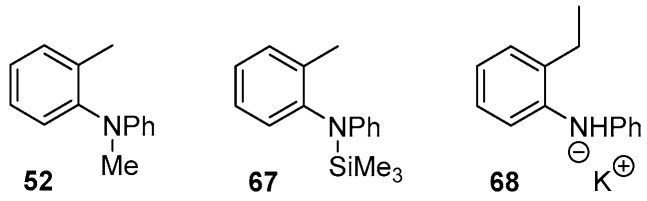
Additional *o*-tolyl aryl amine substrates investigated.

The above discussion assumes that salt **57** is the reactive species in solution. However, it has recently been shown by Palumbo et al. [36] that amide anions can be silylated by Et_3_SiH/KO^t^Bu. Therefore, a substrate containing a SiMe_3_ group bonded to the nitrogen atom, **67**, was explored (Figure 1). Effectively, substrate **67** features a tertiary amine, as does substrate **52**, so the reactivity of substrate **52** is considered below, after that of **67**. Subsequently, our studies on an additional substrate, **68,** will be reported below. Its relevance lies in the fact that, although all of our substrates to date have been *ortho*-tolyl amines and ethers, our experimental interests lie in extending studies to more complex substrates, where the tolyl methyl group is replaced by an extended chain, for which substrate **68** would be the simplest computational model.

Cyclisation of substrate **67** was studied via both benzyl radical and benzyl anion intermediates and the energy profiles for formation of these intermediates are shown in Table 1. The initial hydrogen atom abstraction of the ortho methyl by a trimethylsilyl radical **24b** has attainable activation energy (17.8 kcal mol^−1^) and is almost thermoneutral (entry 1, Table 1A).

The energy profiles for the cyclisation of benzyl radical **69** were closely balanced: for 6-aryl cyclisation, ΔG^‡^ = 24.6 kcal mol^−1^ and ΔG_rel_ = 2.3 kcal mol^−1^, while for 5-*exo* cyclisation, ΔG^‡^ = 25.1 kcal mol^−1^ and ΔG_rel_ = 7.9 kcal mol^−1^, (see Figures S7 and S8) and so the expectation would be that a mixture of diarylmethane and dihydroacridine products would be produced by this pathway.

Addressing possible anionic pathways, reduction of radical **69** to anion **71** was found to be feasible (entry 1, Table 1B), making the benzyl anion a likely participant in the reaction. Direct deprotonation of the methyl group of the *o*-tolyl ring of **67** by pentavalent silicate **25b** was also energetically viable (entry 1, Table 1C). Anionic cyclisations of the benzyl anion intermediates formed from these initiation routes were investigated next (Table 2). The anionic 5-*exo*-trig cyclisation of **73** has a very achievable activation energy (ΔG^‡^ = 26.5 kcal mol^−1^) [66,67] similar to the alternative 6-aryl cyclisation (ΔG^‡^ = 27.7 kcal mol^−1^), and so the expectation would be that a mixture of diarylmethane and dihydroacridine products would also be produced by this pathway.

As both the radical and the anionic routes predict that a diarylmethane product should be formed from the silylated substrate **67**, and as no such product is observed experimentally, we conclude that silylation of secondary amine substrates plays no role in their conversion to products.

Turning now to examine substrate **52**, conversion to the benzyl radical **70** is easily achieved (Table 1A, entry 2). Radical cyclisations of substrate **52** showed a preference for 6-aryl cyclisation (ΔG^‡^= 19.8 kcal mol^−1^, ΔG_rel_ = −2.7 kcal mol^−1^) versus 5-*exo* cyclisation (ΔG^‡^= 23.1 kcal mol^−1^, ΔG_rel_ = 4.8 kcal mol^−1^) (see Figures S11 and S12). Accordingly, the radical cyclisation pathway favours formation of dihydroacridine product, **55** (Scheme 9).

Reduction of radical **70** to anion **72** witnesses a low barrier (3.8 kcal mol^−1^) and is exergonic. Anion **72** would be further stabilised by complexing with a potassium ion to form **74** (see Figures S15 and S16). Salt **74** could alternatively arise by direct deprotonation of substrate **52** by strong base **25b**. Table 1C (entry 2) shows that this is also an energetically accessible route.

Table 2 (entry 2) reports the energy profile for 5-*exo* and 6-aryl cyclisations of salt **74**. 5-*Exo*-trig cyclisation is almost thermoneutral and has an accessible barrier, while 6-aryl cyclisation is not at all favoured, with its very high barrier (ΔG^‡^ = 31.0 kcal mol^−1^); furthermore, it is quite endergonic (ΔG_rel_ = 8.5 kcal mol^−1^). Therefore, the anionic pathway favours the pathway that leads towards a diarylmethane product **56**

Scheme 9 shows that both dihydroacridine **55** and diarylmethane **56** are isolated from the reaction of substrate **52**, suggesting that both radical and anionic pathways contribute to product formation. Computation clearly shows that both cyclisation routes are accessible, but it is challenging to define the relative contribution of each pathway. 

The final substrate to be examined was substrate **68** (Figure 1). It is a close analogue of substrate **50b**. In that case, we have reported above that the pathway through benzyl radical was operative, while that through a benzyl anion was not. Accordingly, here we investigated solely the radical pathway. Table 3 presents the energy changes for the cyclisation step of the radical pathway for radical **79** derived from substrate **68** and compares them with radical **58b** derived from substrate **50b**. As for **50b**, the 6-aryl cyclisation is the preferred pathway, with the cyclisation being somewhat facilitated for the more highly substituted **68**.

The computations report that the cyclisation of substrates like **68** proceed through a benzylic radical intermediate rather than through an anionic counterpart. In terms of future plans, this informs us that more complex sidechains for analogues of **68** should be designed with substituents that do not intercept the benzyl radical more rapidly than it attacks the target aryl ring.

In this paper, we have relied on computational assessment of the mechanism. This is appropriate, as the computation can compare the favourability of cyclisation of radical and anionic intermediates that could arise from identical substrates. There are of course, experimental approaches to determining whether a reaction is radical [68] or anionic [69], and indeed we have used such methods in previous studies on different chemistry [70,71]. A benzyl radical intermediate might be probed by means of an adjacent radical clock or by trapping with a commercial persistent radical, e.g., TEMPO. However, TEMPO would likely intercept the first radical intermediates in our pathway (silyl radicals) and this would inhibit that pathway before the benzyl radicals were formed, while radical clocks would necessarily intercept the relevant benzyl radical intermediate prior to cyclisation, and thus the test substrates would not actually cyclise (detecting a benzyl radical would not mean that it was normally responsible for the cyclisation). On the other hand, a benzyl anion intermediate might be reported by a leaving group (e.g., OMe) on the adjacent carbon, elimination of which would give rise to a styrene. However, this substrate would then not cyclise, and so it would not be clear which intermediate was responsible for the cyclisation.

## 3. Materials and Methods

### 3.1. Experimental Details

All reagents and solvents were obtained from commercial suppliers and were used without further purification unless mentioned otherwise. Anhydrous diethyl ether, THF and dichloromethane (DCM) were dried using a Pure-Solv 400 solvent purification system (Innovative Technology Inc., USA). DMF was dried over 3 Å pre-activated molecular sieves. Molecular sieves were activated by three heating cycles in the microwave, followed by evacuation under vacuum.

The glovebox was supplied by Innovative Technology Inc., Herndon, VA, USA, which is operated with a nitrogen atmosphere.

Thin Layer Chromatography was performed on silica gel pre-coated aluminium plates (60 Å, F254 UV indicator) purchased from Merck. The thin layer chromatograms were analysed by UV (254 nm, UVP mineralight UVG-11 lamp) and staining either with basic KMnO_4_ [KMnO_4_ (6 g), K_2_CO_3_ (40 g), NaOH (5 mL, 10% *w*/*w*) in water (600 mL)] or an ethanolic solution of phosphomolybdic acid [phosphomolybdic acid hydrate (10 g) in ethanol (100 mL)]. 

Flash Column Chromatography purification was performed with 35-70 μm particle size silica gel 60 Å (200–400 mesh) purchased from Prolabo. 

NMR spectra were measured on a Bruker AV400 instrument. ^1^H and ^13^C NMR spectra were obtained at 400 and 101 MHz, respectively. Spectra were recorded in chloroform-*d*_1_. The frequency was locked against the deuterated solvent signal and the final spectra were referenced against the residual non-deuterated solvent signal (for ^1^H spectra) or the deuterated solvent signal (for ^13^C spectra). Chemical shifts are reported as δ (ppm) with respect to tetramethylsilane. The following multiplet abbreviations are used: s = singlet, d = doublet, t = triplet, td = triplet of doublets, m = multiplet, b = broad. 

Infrared spectra were recorded on a Shimadzu IRAffinity-1 instrument. GC-(EI)MS analysis was performed on an Agilent Technologies 7890A GC system connected to an Agilent Technologies 5975C inert XL EI/CI MSD triple axis-mass detector. The GC was equipped with a Rxi-5Sil MS column (30 m × 0.25 mm × 0.25 μm). Helium was used as the carrier gas (1.0 mL/min flow rate). The injector temperature was 320 °C and was operated in splitless mode. 

High resolution mass spectrometry (HRMS) was conducted at the University of Glasgow using a Bruker microTOFq High Resolution Mass Spectrometer. This instrument has an Electrospray (ESI) ion source coupled to a time-of-flight (ToF) analyser.

### 3.2. Substrate Synthesis

#### 3.2.1. Preparation of 1-methyl-2-phenoxybenzene (1)

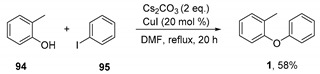



This substrate was prepared according to a literature procedure [29].

**1 ^1^H NMR** (400MHz, CDCl_3_) δ 7.32–7.26 (m, 2H), 7.25–7.23 (m, 1 H), 7.19–7.13 (m, 1 H), 7.11–6.99 (m, 2H), 6.93–6.87 (m, 3H), 2.24 (s, 3H); **^13^C NMR** (101MHz, CDCl_3_) δ 158.0, 154.6, 131.6, 130.2, 129.8, 127.2, 124.1, 122.4, 119.9, 117.4, 16.3; **ATR-IR ν_max_** (neat)/cm^−1^ 1582, 1485, 1233, 1111, 874, 748, 691; **GC-MS** [*m/z* (%)] (11.33 min): 184.3 (99, [M]+), 165.2 (73), 155.2 (60), 141.2 (100), 128.2 (51), 115.2 (76), 106.2 (80), 91.2 (88), 78.2 (89), 65.2 (96), 50.2 (73). Analytical data in agreement with those reported in the literature [29]. 

#### 3.2.2. Preparation of N,2-dimethyl-N-phenylaniline (52)

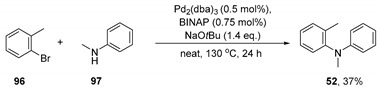



This substrate was prepared according to a literature procedure [72]. To an oven-dried pressure tube equipped with a stirrer bar was added NaO*t*Bu (2.11 g, 22 mmol, 1.4 equiv.), Pd_2_(dba)_3_ (73 mg, 0.5 mol %), BINAP (74 mg, 0.75 mol %), *N*-methylaniline **97** (2 mL, 19 mmol, 1.2 equiv.), and 2-bromotoluene **96** (1.93 mL, 16 mmol, 1 equiv.). The liquid substrates were added last. The vial was flushed with a stream of argon and tightly capped. The mixture was refluxed in a pre-heated oil bath at 130 ℃ for 24 h. The mixture was allowed to cool to room temperature, taken up in ether (50 mL), filtered, and concentrated. The crude product was then purified by column chromatography (100% hexane 3% EtOAc in hexane) to afford *N*,2-dimethyl-*N*-phenylaniline **52** as a colourless oil (1.16 g, 37%). 

**52 ^1^H NMR** (400 MHz, CDCl_3_) δ 7.31–7.27 (m, 1H), 7.25–7.12 (m, 5H), 6.74–6.68 (tt, *J* = 7.3 Hz, 1.0 Hz, 1H), 6.56–6.50 (dd, *J* = 8.8, 1.0 Hz, 2H), 3.22 (s, 3H), 2.15 (s, 3H); **^13^C NMR** (101 MHz, CDCl_3_) δ 148.7, 146.3, 136.3, 130.9, 128.5, 127.8, 127.0, 125.9, 116.3, 112.3, 38.5, 17.3; **ATR-IR ν_max_** (neat)/cm^−1^ 3022, 2877, 1593, 1490, 1338, 1251, 1112, 746, 727, 691; **GC-MS** [*m/z* (%)] (12.56) min: 198.3 (100, [M]+), 183.2 (89), 165.2 (62), 155.2 (66), 107.2 (63), 91.2 (97), 77.2 (93), 65.2 (96), 51.2 (82). Analytical data are in agreement with those reported in the literature [73].

### 3.3. Reactions of Substrates

#### 3.3.1. Reaction of 1-methyl-2-phenoxybenzene 1 with KOtBu—Neat

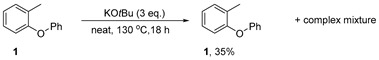



Substrate **1** (92 mg, 1.0 equiv., 0.5 mmol) and KO^*t*^Bu (3.0 equiv., 1.5 mmol, 168 mg) were sealed in a pressure tube in a nitrogen-filled glovebox. This experiment was carried out neat and the contents subjected to the reaction conditions as is. The mixture was stirred at 130 °C for 18 h before the pressure tube was cooled to room temperature, opened to air and diluted with water (50 mL). The organic products were extracted into Et_2_O (3 × 50 mL). The combined organic phases were dried over Na_2_SO_4_, filtered and concentrated. Analysis of the crude mixture afforded starting material **1** (34 mg, 35%) alongside traces of an unidentifiable complex mixture.

#### 3.3.2. Reaction of 1-methyl-2-phenoxybenzene 1 with KOtBu—in THF

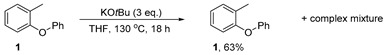



Substrate **1** (92 mg, 1.0 equiv., 0.5 mmol) and KO^*t*^Bu (3.0 equiv., 1.5 mmol, 168 mg) were added to a pressure tube, followed by THF (5 mL) in a nitrogen-filled glovebox. The tube was sealed, removed from the glovebox, and the mixture stirred at 130 °C for 18 h. After reaction, the pressure tube was cooled to room temperature, opened to air and diluted with water (50 mL). The organic products were extracted into Et_2_O (3 × 50 mL). The combined organic phases were dried over Na_2_SO_4_, filtered and concentrated. Analysis of the crude mixture afforded starting material **1** (58.4 mg, 63%) alongside traces of an unidentifiable complex mixture.

#### 3.3.3. Reaction of N,2-dimethyl-N-phenylaniline 52 with KOtBu/Et_3_SiH—in THF

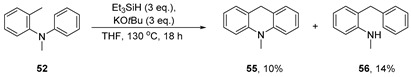



Substrate **52** (99 mg, 1.0 equiv., 0.5 mmol), KO^*t*^Bu (3.0 equiv., 1.5 mmol, 168 mg), and Et_3_SiH (3.0 equiv., 1.5 mmol, 240 μL) were dissolved in THF (5 mL) and sealed in a pressure tube in a nitrogen-filled glovebox. The contents of the pressure tube were stirred at 130 °C for 18 h before the pressure tube was cooled to room temperature, opened to air and diluted with water (50 mL). The organic products were extracted into Et_2_O (3 × 50 mL). The combined organic phases were dried over Na_2_SO_4_ and concentrated *in vacuo*. The crude material was purified using column chromatography (50% hexane in toluene→100% toluene) affording 10-methyl-9,10-dihydroacridine **55** as a yellow oil (10.8 mg, 10%) and 2-benzyl-*N*-methylaniline **56** as a yellow oil (15.4 mg, 14%).

**55 ^1^H NMR** (400 MHz, CDCl_3_) δ 7.22–7.14 (m, 4H), 6.96–6.85 (m, 4H), 3.89 (s, 2H), 3.38 (s, 3H); **^13^C NMR** (101 MHz, CDCl_3_) 143.8, 127.7, 127.0, 124.5, 120.7, 112.0, 33.4, 33.3 **ATR-IR ν_max_** (neat)/cm^−1^ 2922, 1635, 1595, 1494, 1460, 1367, 1178, 752; **GC-MS** [*m/z* (%)] (14.37 min): 194 (100, [M-H]+), 176 (42), 152 (14), 126 (4), 97 (9), 63 (10). Analytical data are in agreement with those reported in the literature [74]. 

**56 ^1^H NMR** (400 MHz, CDCl_3_) δ 7.32–7.26 (m, 2H), 7.24–7.18 (m, 2H), 7.16 (d, *J* = 7.3 Hz, 2H), 7.05–7.00 (d, *J* = 7.1 Hz, 1H), 6.77 (t, *J* = 7.4 Hz, 1H), 6.65 (d, *J* = 8.1 Hz, 1H), 3.87 (s, 2H), 3.53 (bs, 1H), 2.77 (s, 3H); **^13^C NMR** (101 MHz, CDCl_3_) δ 147.3, 139.4, 130.6, 128.8, 128.6, 128.0, 126.5, 124.7, 117.1, 110.1, 38.0, 30.9; **ATR-IR ν_max_** (neat)/cm^−1^: 3431, 2893, 1604, 1512, 1307, 1161, 729. **HRMS (ESI)**: calculated for C_14_H_16_N ([M+H]^+^): 198.1277 found: 198.1277. NMR data are in agreement with those reported in the literature [75]. 

#### 3.3.4. Reaction of N,2-dimethyl-N-phenylaniline 52 with KOtBu/Et_3_SiH—Neat

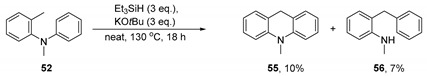



Substrate **52** (99 mg, 1.0 equiv., 0.5 mmol), KO^*t*^Bu (3.0 equiv., 1.5 mmol, 168 mg), and Et_3_SiH (3.0 equiv., 1.5 mmol, 240 μL) were sealed in a pressure tube in a nitrogen-filled glovebox. The contents of the pressure tube were stirred at 130 °C for 18 h before the pressure tube was cooled to room temperature, opened to air and diluted with water (50 mL). The organic products were extracted into Et_2_O (3 × 50 mL). The combined organic phases were dried over Na_2_SO_4_ and concentrated *in vacuo*. The crude material was purified using column chromatography (50% hexane in toluene→100% toluene), affording 10-methyl-9,10-dihydroacridine **55** as a yellow oil (10.6 mg, 10%) and 2-benzyl-*N*-methylaniline **56** as a yellow oil (6.8 mg, 7%).

Analytical data for **61** and **62** are in agreement with the corresponding data reported above.

## 4. Conclusions

In summary, subjecting *o*-tolylaryl ethers and amines to the Et_3_SiH/KO*^t^*Bu system yields rearranged products. *o*-Tolylaryl ethers undergo a concerted Truce–Smiles rearrangement to yield diarylmethane products that is initiated by benzyl anions formed by two competitive routes: a radical-polar crossover consisting of a hydrogen atom abstraction by a trialkylsilyl radical **24a** followed by a SET reduction via silyl radical anion **26a**, and/or the direct deprotonation of the ortho methyl group by the pentavalent silicate base that is formed in situ.

*o*-Tolyl arylamines that are secondary, or that contain a labile group bonded to the nitrogen atom, result in the formation of dihydroacridine products through a radical pathway when treated with the Et_3_SiH/KO*^t^*Bu system. Tertiary amines form both dihydroacridines and diarylmethanes through radical and anionic pathways respectively. Overall, this study showcases how the reactive intermediates of the Et_3_SiH/KO*^t^*Bu compete with one another during the reaction mechanisms allowing for a broad range of chemical outcomes and possibilities.

This research provides mechanistic detail on another of the expanding family of transformations that can be achieved by KO*^t^*Bu + Et_3_SiH. This reagent pair unusually produces at least three silicon-based reactive intermediates, making determination of mechanism both challenging and important; the knowledge from our study can contribute to future understanding of the Grubbs–Stoltz system. In terms of the development of this specific project, the results reported here allow us to plan the synthesis of more complex substrates, e.g., based on **68**. Knowing that a benzyl radical is the intermediate that cyclises allows us to plan extended side-chains that will not intercept the benzyl radical before it cyclises onto the target arene ring.

## Figures and Tables

**Table 1:** Investigation into (**A**) the hydrogen atom abstraction facilitated by **24b**, (**B**) the radical-polar crossover mediated by **26b**, and (**C**) direct deprotonation of the ortho methyl group via pentavalent silicate **25b** for substrates **65** and **58**.

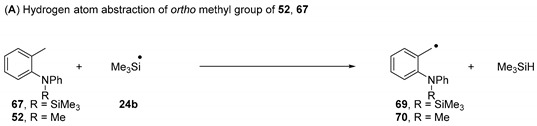			
**Entry**	**R**	**ΔG^‡^ (kcal mol^−1^)**	**ΔG_rel_ (kcal mol^−1^)**
**1**	SiMe_3_	17.8	1.8
**2**	Me	18.2	0.4
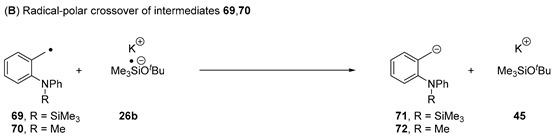			
**Entry**	**R**	**ΔG^‡^ (kcal mol^−1^)**	**ΔG_rel_ (kcal mol^−1^)**
**1**	SiMe_3_	2.0	−7.3
**2**	Me	3.8	−8.6
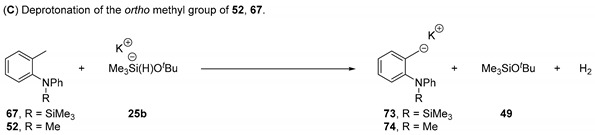			
**Entry**	**R**	**ΔG^‡^ (kcal mol^−1^)**	**ΔG_rel_ (kcal mol^−1^)**
**1**	SiMe_3_	28.2	−7.1
**2**	Me	25.3	−7.8

**Table 2:**
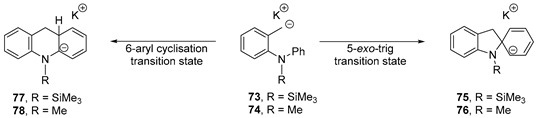
Summary of activation energies and relative changes in Gibbs free energy for the cyclisation step of the anionic mechanisms for anions **73** and **74**.

**Entry**	**Anion**	**5-*exo*-trig**		**6-aryl**	
**-**	**-**	**ΔG^‡^ (kcal mol^−1^)**	**ΔG_rel_ (kcal mol^−1^)**	**ΔG^‡^ (kcal mol^−1^)**	**ΔG_rel_ (kcal mol^−1^)**
**1**	**73**	26.5	3.0	27.7	8.6
**2**	**74**	26.5	1.1	31.0	8.5

**Table 3:**
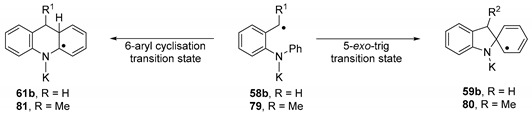
Summary of activation energies and relative change in Gibbs free energy for the cyclisation steps for radical **79** compared to **58b**.

**Entry**	**Radical**	**5-*exo*-trig**		**6-aryl**	
**-**	**-**	**ΔG^‡^ (kcal mol^−1^)**	**ΔG_rel_ (kcal mol^−1^)**	**ΔG^‡^ (kcal mol^−1^)**	**ΔG_rel_ (kcal mol^−1^)**
**1**	**58b**	31.5	17.1	22.8	−2.5
**2**	**79**	27.9	16.6	20.2	−0.8

## Data Availability

Data are contained within the article or Supplementary Material.

## Supplementary Materials

The following are available online, xyz coordinates of all computed structures, and NMR spectra.

## Sample Availability

Samples of the compounds are not available from the authors.
