# Supplementary material for: Radical and Ionic Mechanisms in Rearrangements of o-Tolyl Aryl Ethers and Amines Initiated by the Grubbs–Stoltz Reagent, Et3SiH/KOtBu
Source: Molecules. 2021 Nov 15;26(22):6879. doi: 10.3390/molecules26226879 (PMC8619283; doi:10.3390/molecules26226879)
Supplement: Supplementary file 1 [file molecules-26-06879-s001.zip › molecules-1440583-supplementary.pdf]

Supplementary Information for:

Radical and Ionic Mechanisms in Rearrangements of o-tolyl aryl ethers and amines initiated by the Grubbs Stoltz reagent, Et_3SiH/KO^tBu

K. Kolodziejczak, A. J. Stewart, T. Tuttle, J.A. Murphy

Table of Contents

Computational Details S3

Reaction Coordinate Diagrams S3

Additional Computational Schemes S14

NMR Data S16

Substrates S16

Products S18

References S20

XYZ Coordinates S21

O-containing S21

Substrates S21

Intermediates S23

Transition States S62

N-containing S79

Substrates S79

Intermediates S84

Transition States S180

Computational Details

DFT calculations were carried out using the M06-2X functional [1,2] with the 6-311++G(d,p) [3-5] basis set on all atoms. All calculations were performed using the C-PCM [6] implicit solvent model with parameters for triethylamine as solvent. No silane (Me_3SiH or Et_3SiH) solvents are parametrised in Gaussian 16, so triethylamine was chosen as solvent since it has a similar dielectric constant (ε = 2.3832) to triethylsilane (ε = 2.323) [7]. All calculations were performed in Gaussian 16 [8] at 403.15 K. While experimental reactions used triethylsilane, affording intermediates **24a-26a**, theoretical studies made use of the corresponding trimethylsilane-derived intermediates, **24b-26b** which were used for computational economy.

Reaction Coordinate Diagrams

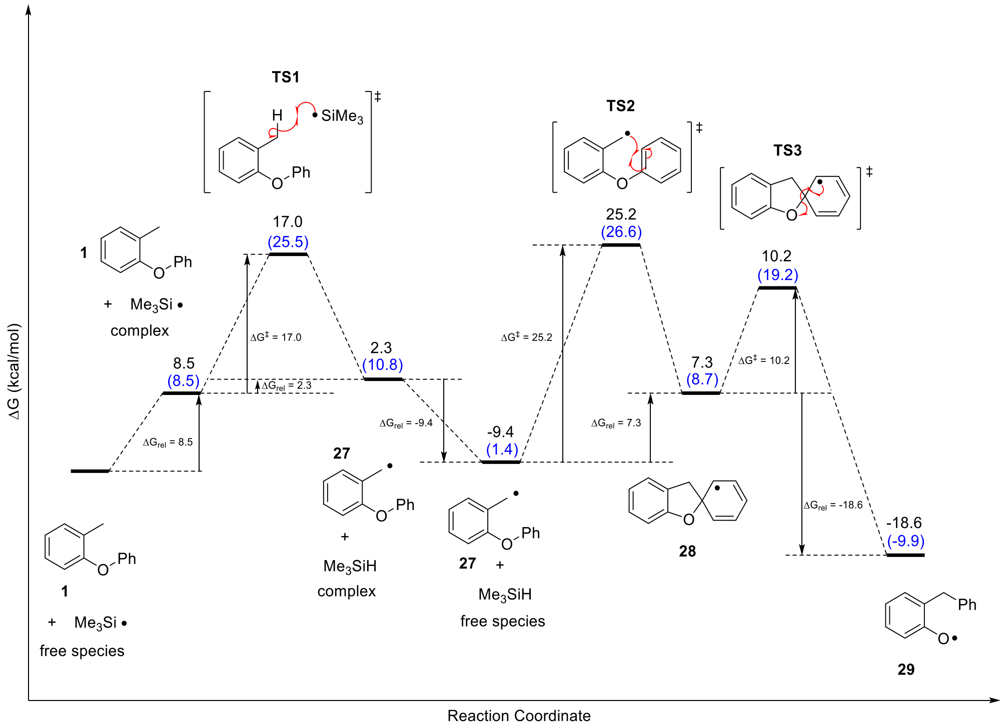

Figure S1**. Reaction coordinate diagram displaying the originally proposed mechanism for the aryl migration reaction of o-tolyl aryl ether **1

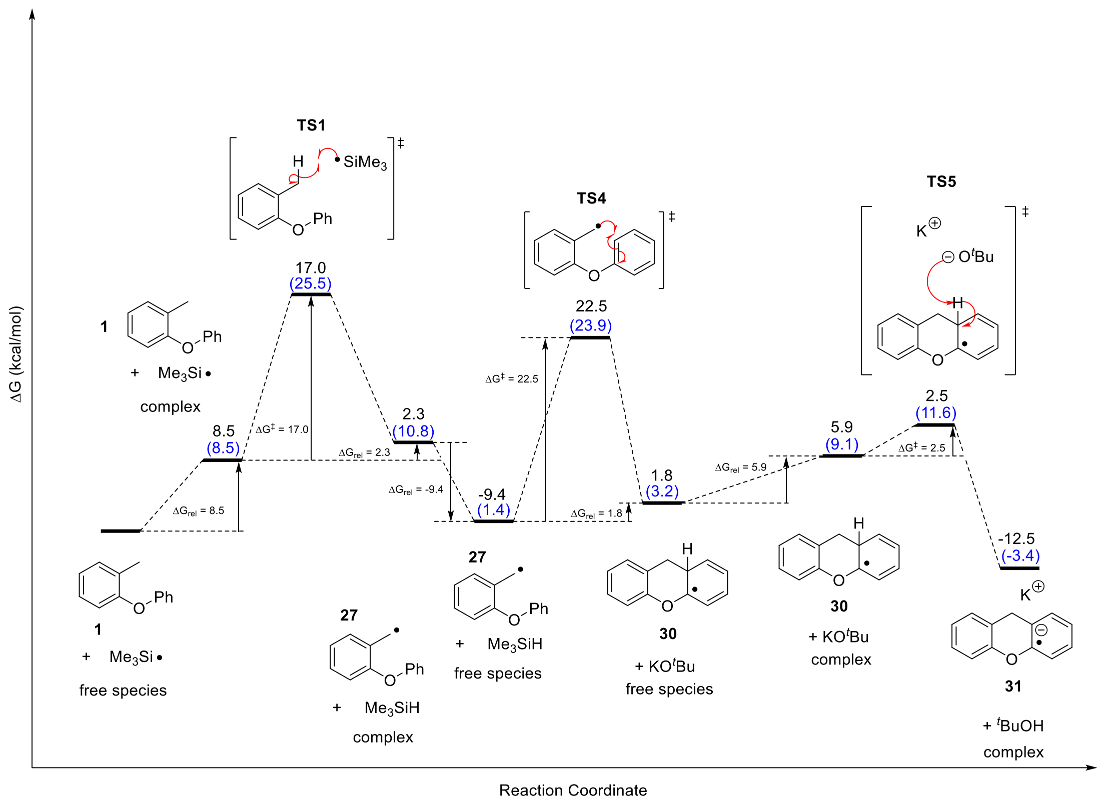

***Figure S2**. Reaction coordinate diagram for the rearrangement of o-tolyl aryl ether **1** featuring a 6-aryl cyclisation*

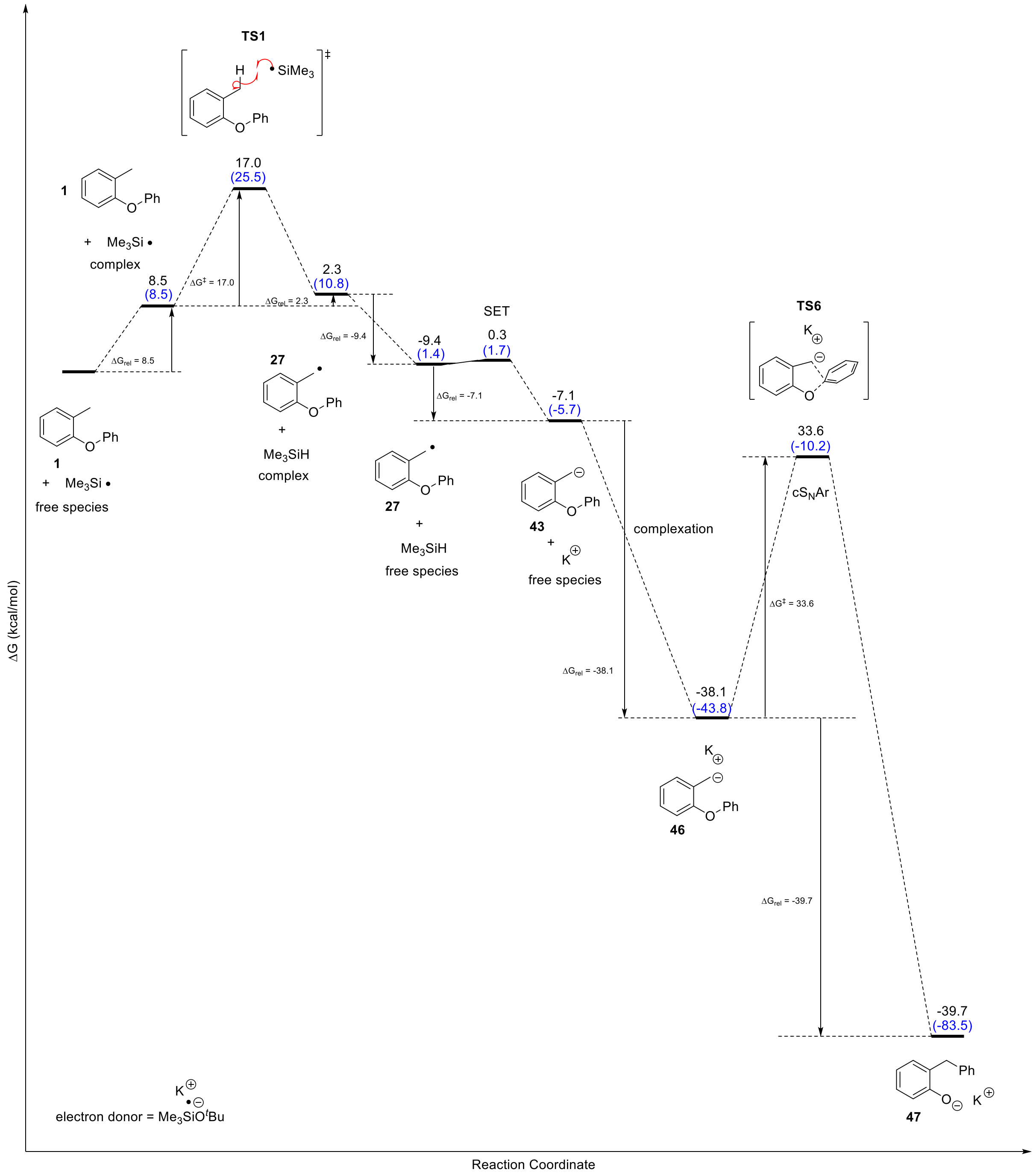

***Figure S3**. Reaction coordinate diagram displaying the Truce-Smiles rearrangement of o-tolyl aryl ether **1** featuring the radical-polar crossover initiation pathway.*

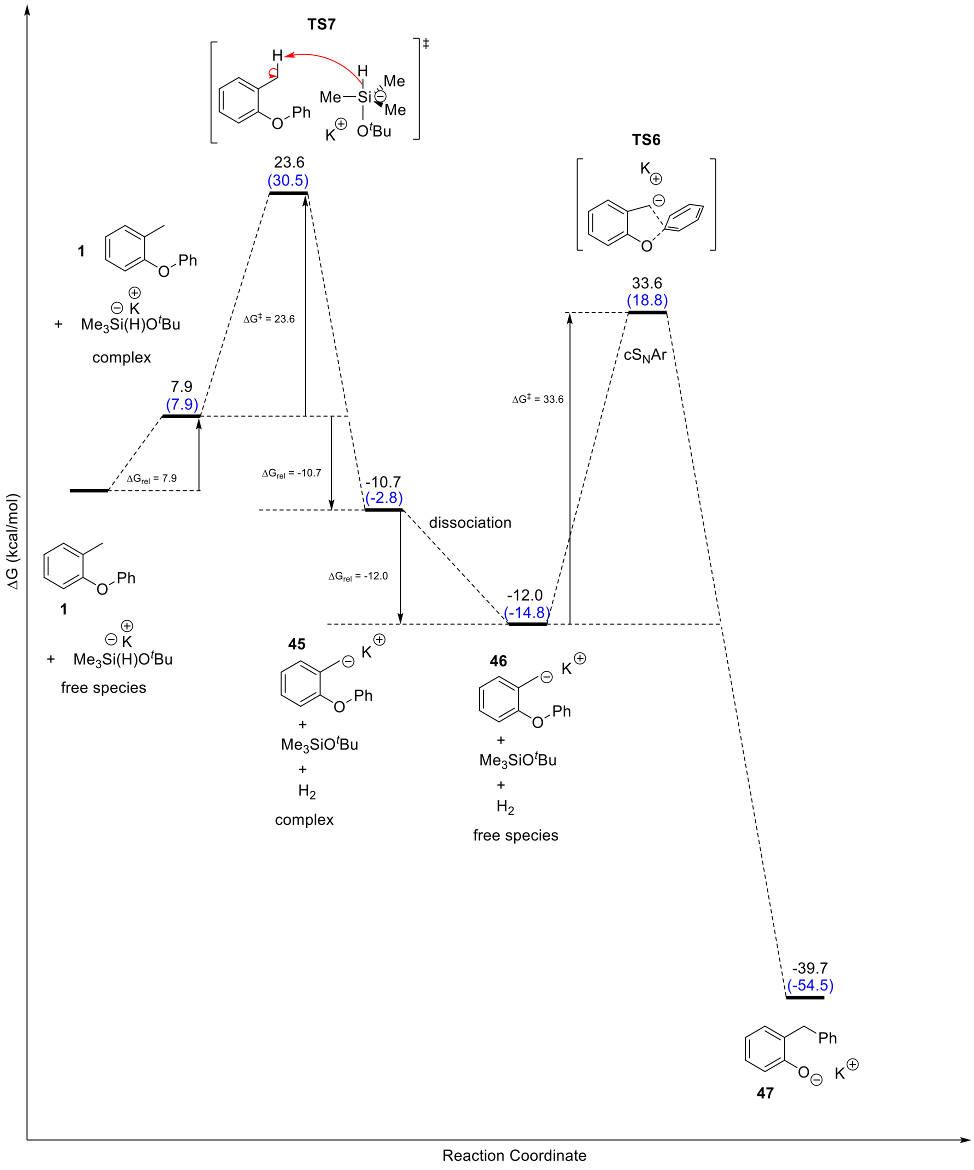

Figure S4. *Reaction coordinate diagram displaying the Truce-Smiles rearrangement of o-tolyl aryl ether **1** featuring the initiation route consisting of a direct deprotonation of the methyl group of the o-tolyl ring by the pentavalent silicate*

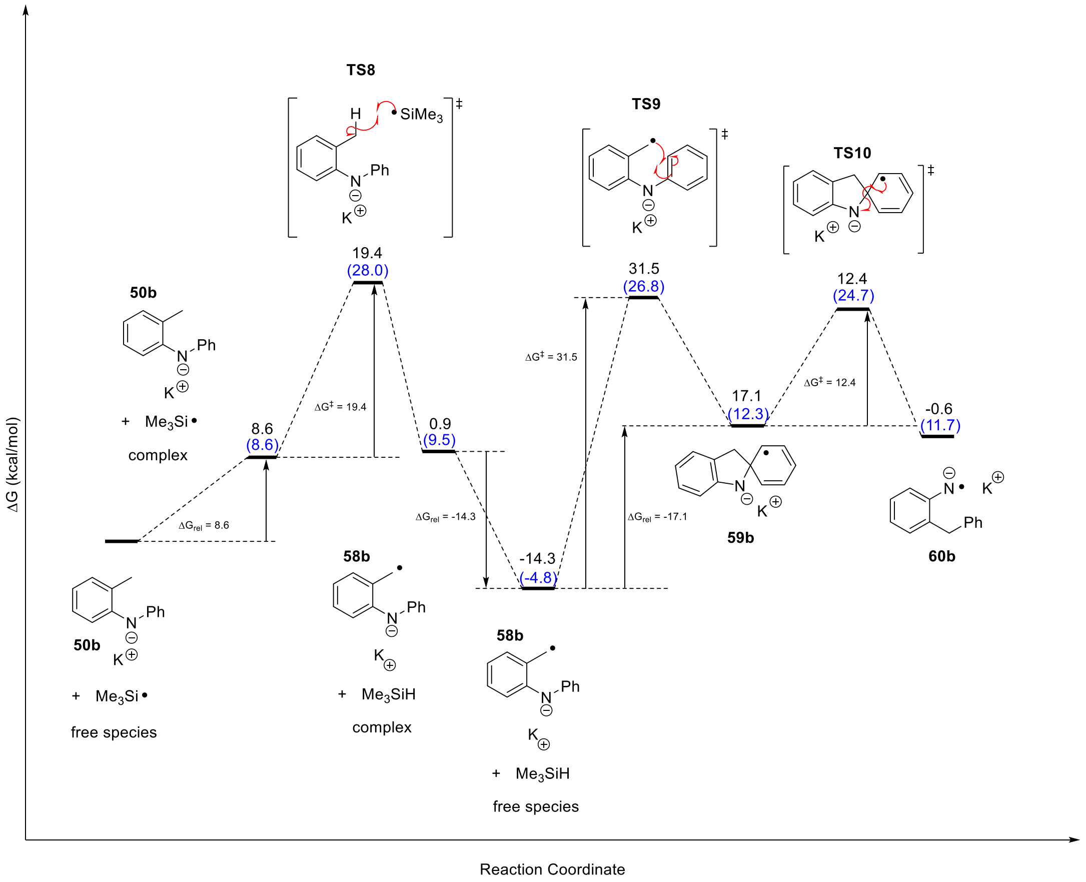

Figure S5. *Reaction coordinate diagram displaying the progress of salt* ***50b*** *through the Smiles rearrangement*

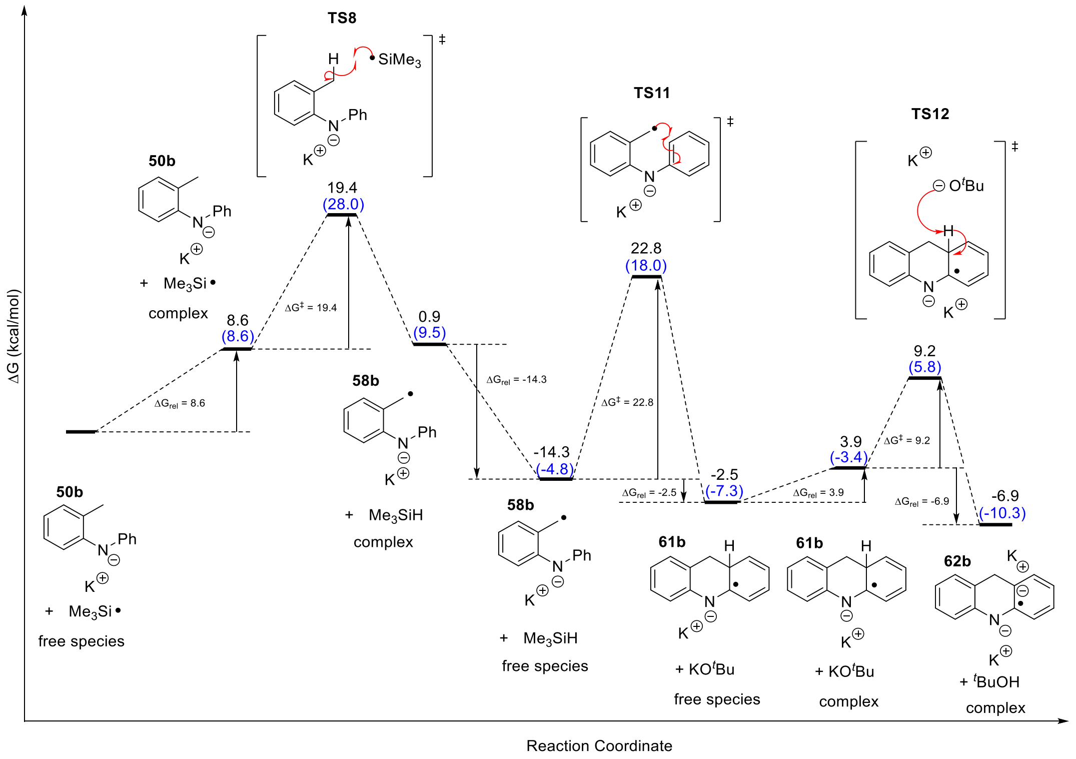

Figure S5*. Reaction coordinate diagram displaying the progress of salt* ***50b*** *through a rearrangement mechanism featuring a 6-aryl cyclisation*

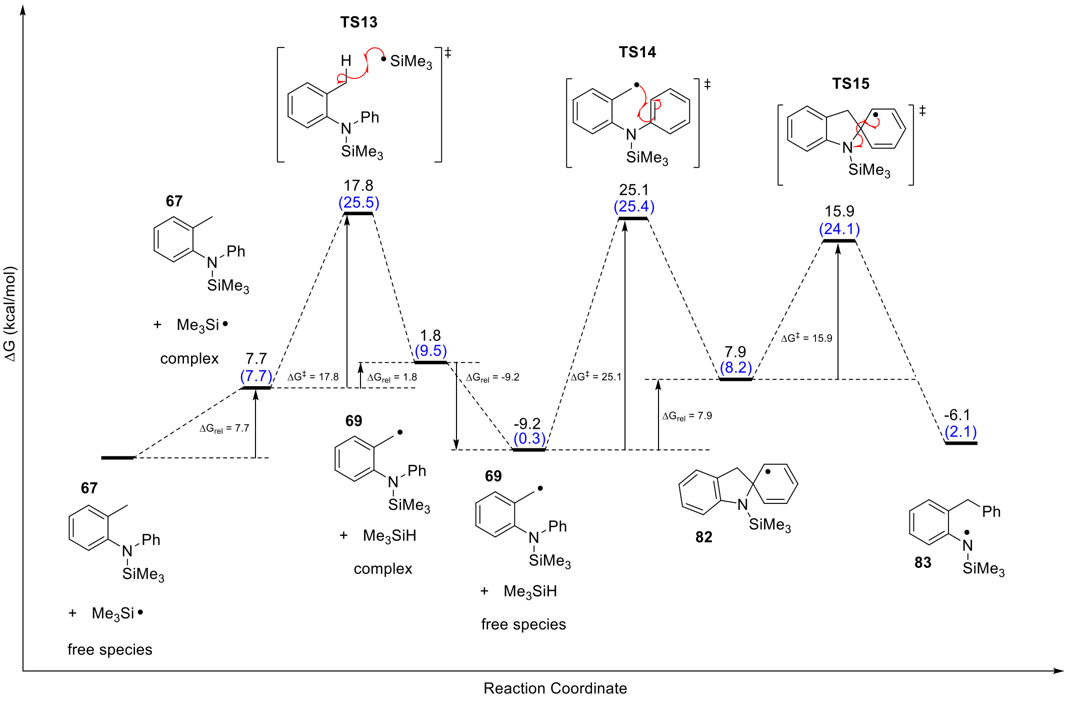

Figure S6*. Reaction coordinate diagram displaying the progress of o-tolyl aryl amine* ***67*** *through the Smiles rearrangement*

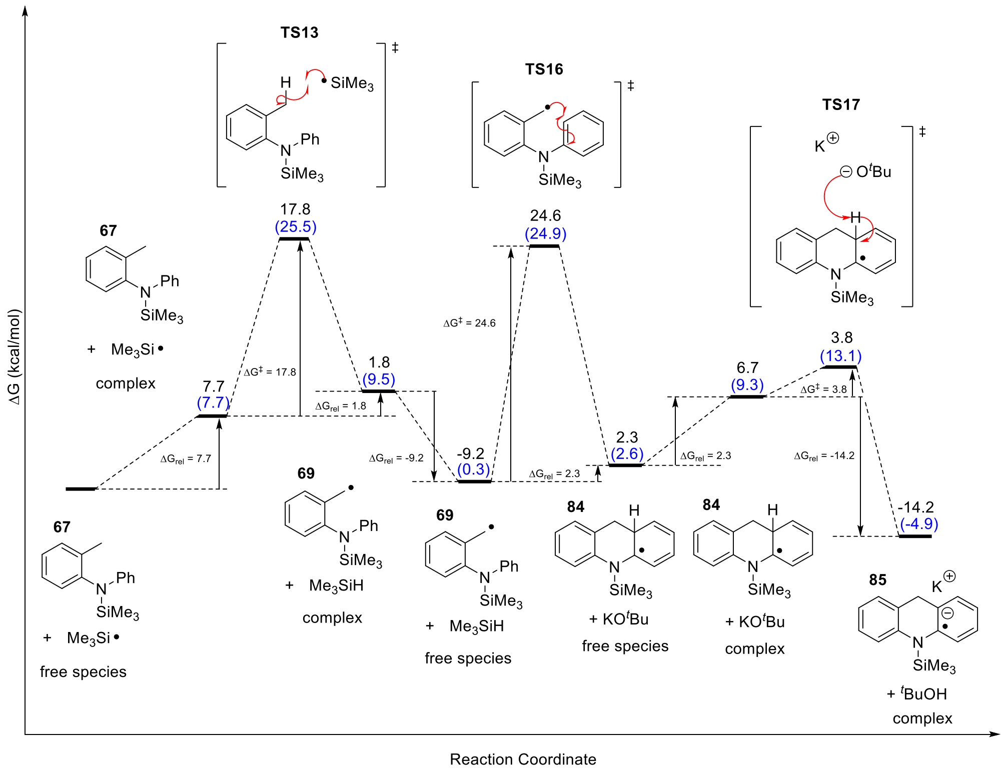

Figure S7*. Reaction coordinate diagram displaying the progress of o-tolyl aryl amine* ***67*** *through a rearrangement mechanism featuring a 6-aryl cyclisation*

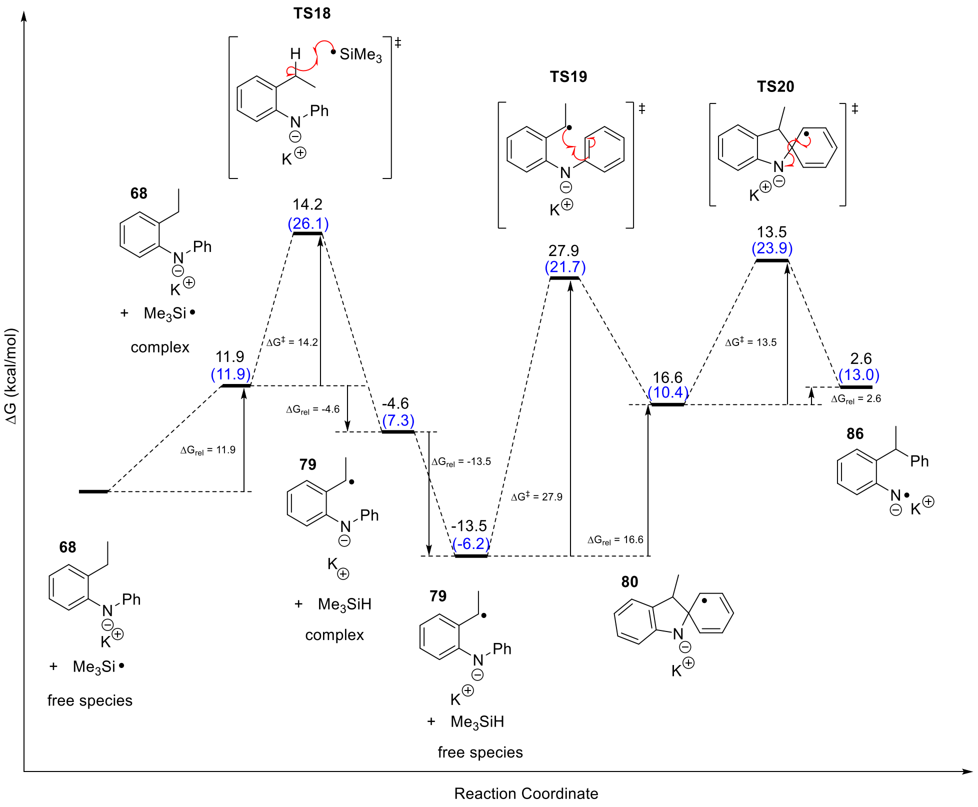

***Figure S8**. Reaction coordinate diagram displaying the progress of o-tolyl aryl amine **68** through the Smiles rearrangement*

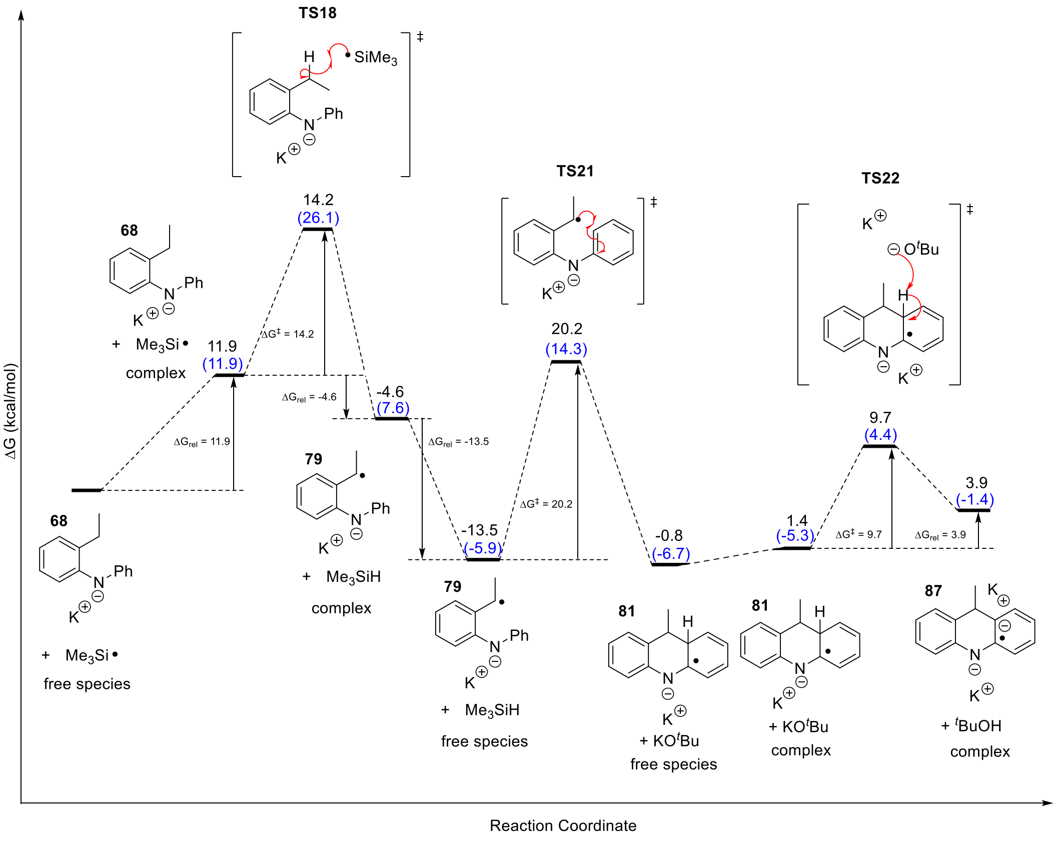

***Figure S9**. Reaction coordinate diagram displaying the progress of o-tolyl aryl amine **68** through a rearrangement mechanism featuring a 6-aryl cyclisation*

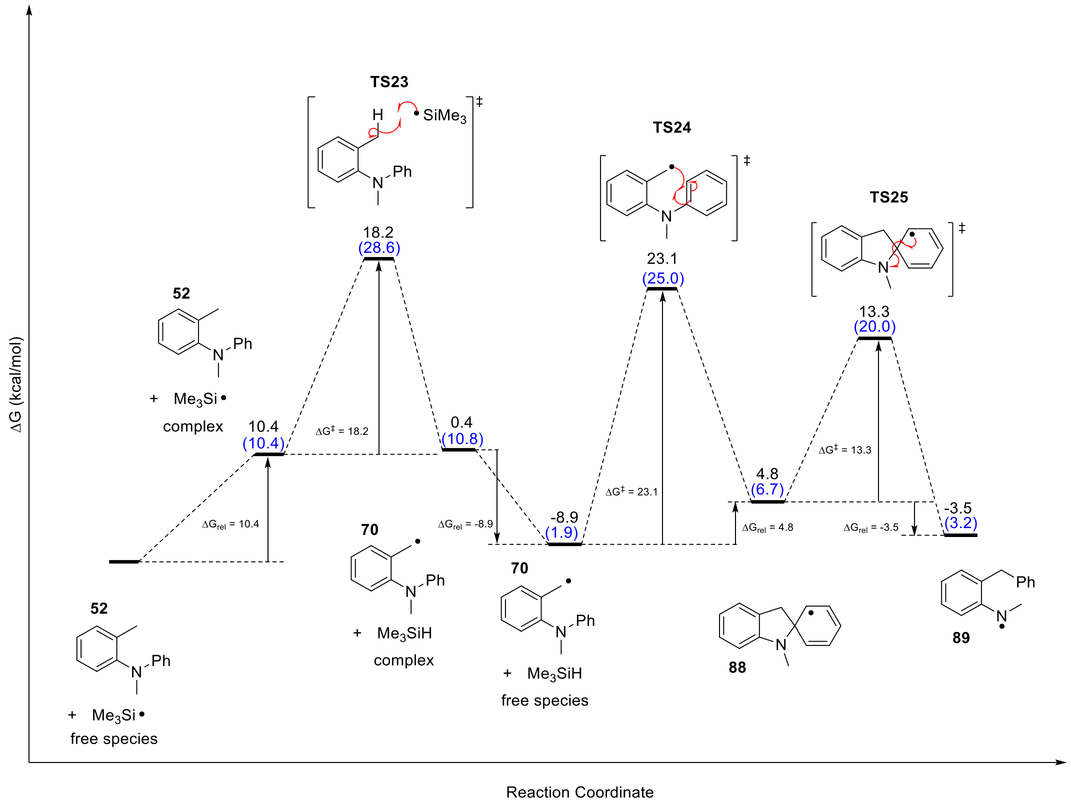

***Figure S11**. Reaction coordinate diagram displaying the progress of o-tolyl aryl amine **52** through the Smiles rearrangement*

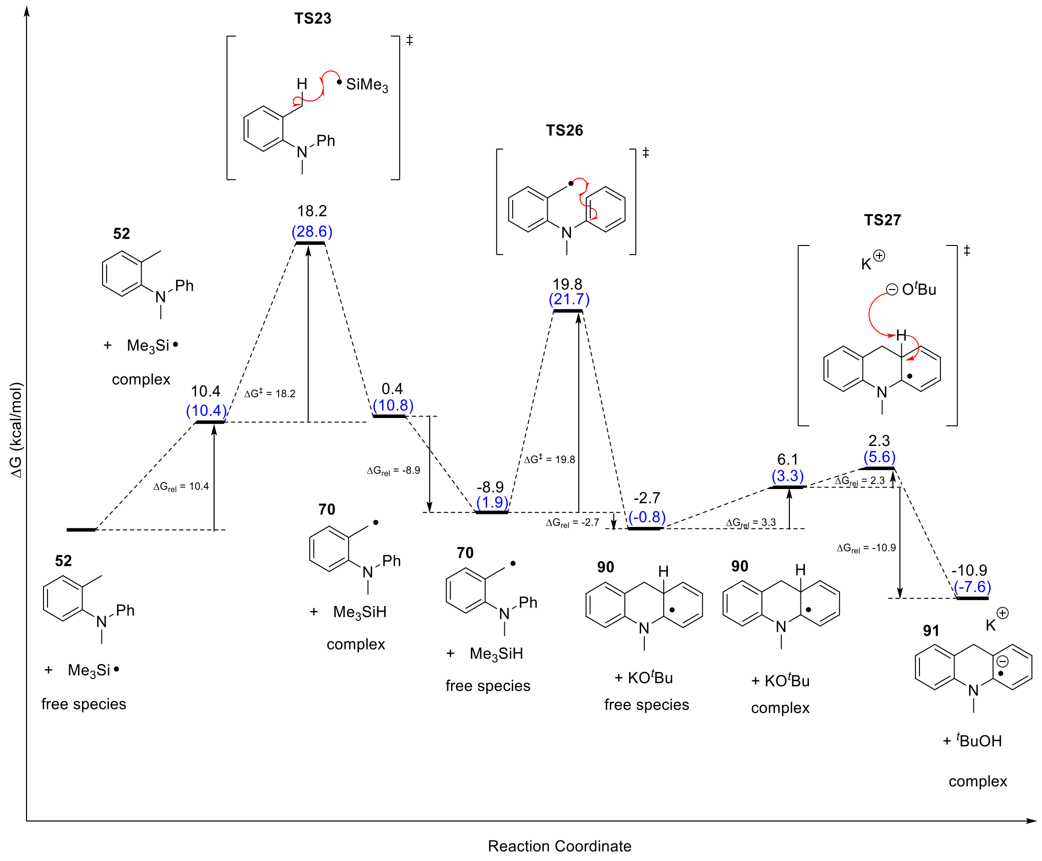

***Figure S12**. Reaction coordinate diagram displaying the progress of o-tolyl aryl amine **52** through a rearrangement mechanism featuring a 6-aryl cyclisation*

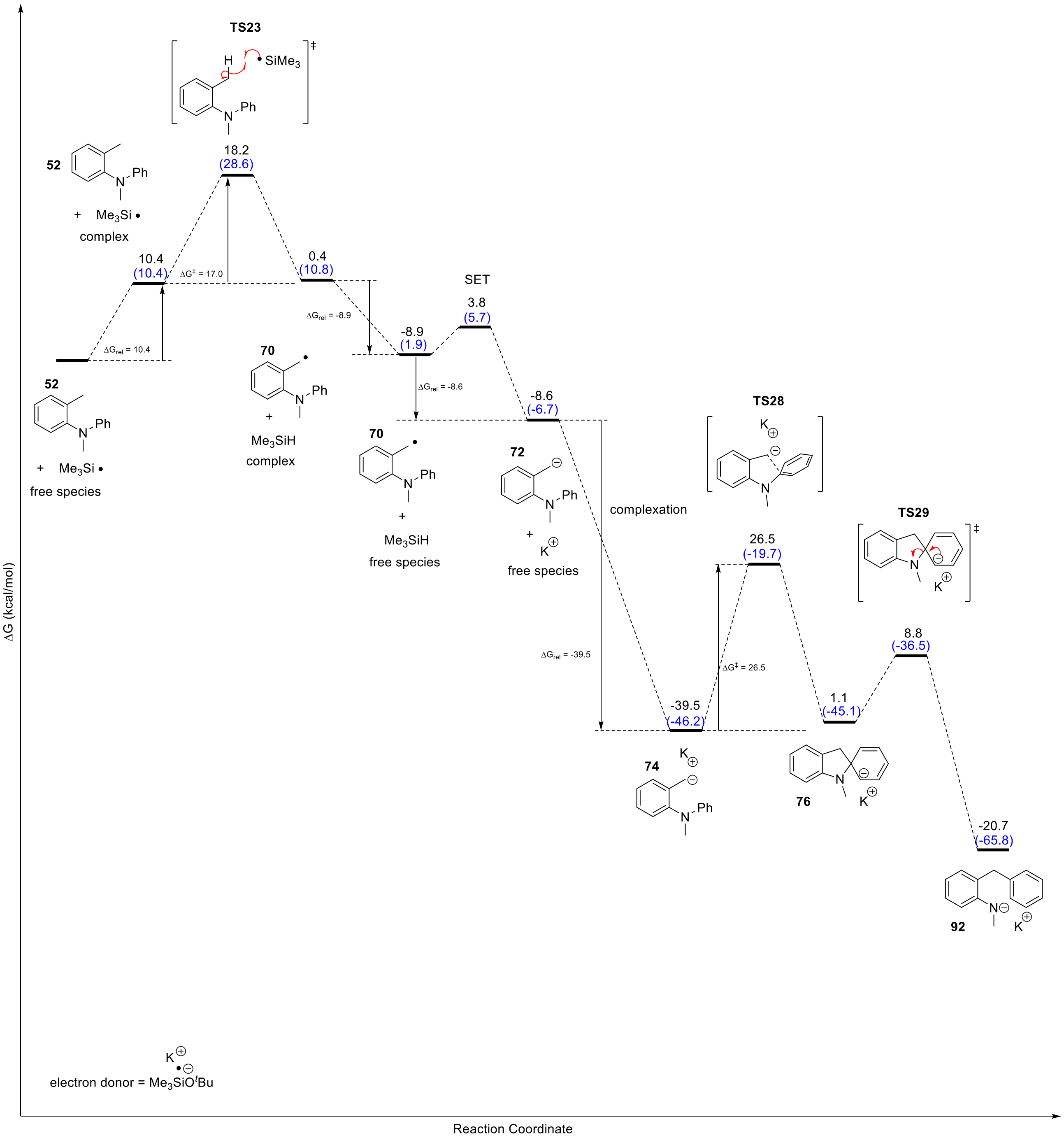

Figure S13. *. Reaction coordinate diagram displaying the progress of o-tolyl aryl amine* ***52*** *through a Truce-Smiles rearrangement featuring the radical-polar crossover initiation route*

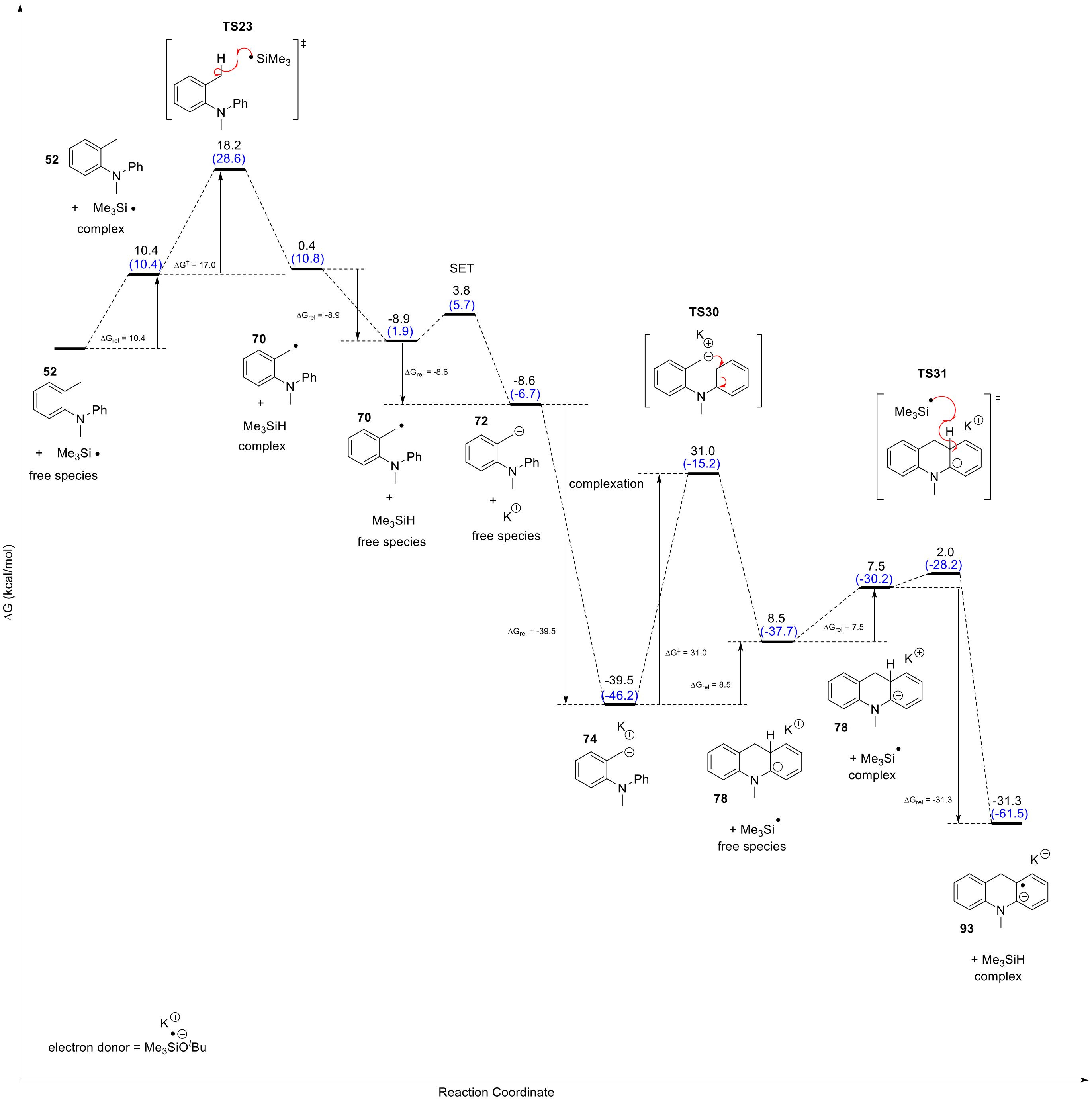

***Figure S14**. Reaction coordinate diagram displaying the progress of o-tolyl aryl amine **52** through an anionic rearrangement mechanism featuring a 6-aryl cyclisation and the radical-polar crossover initiation route*

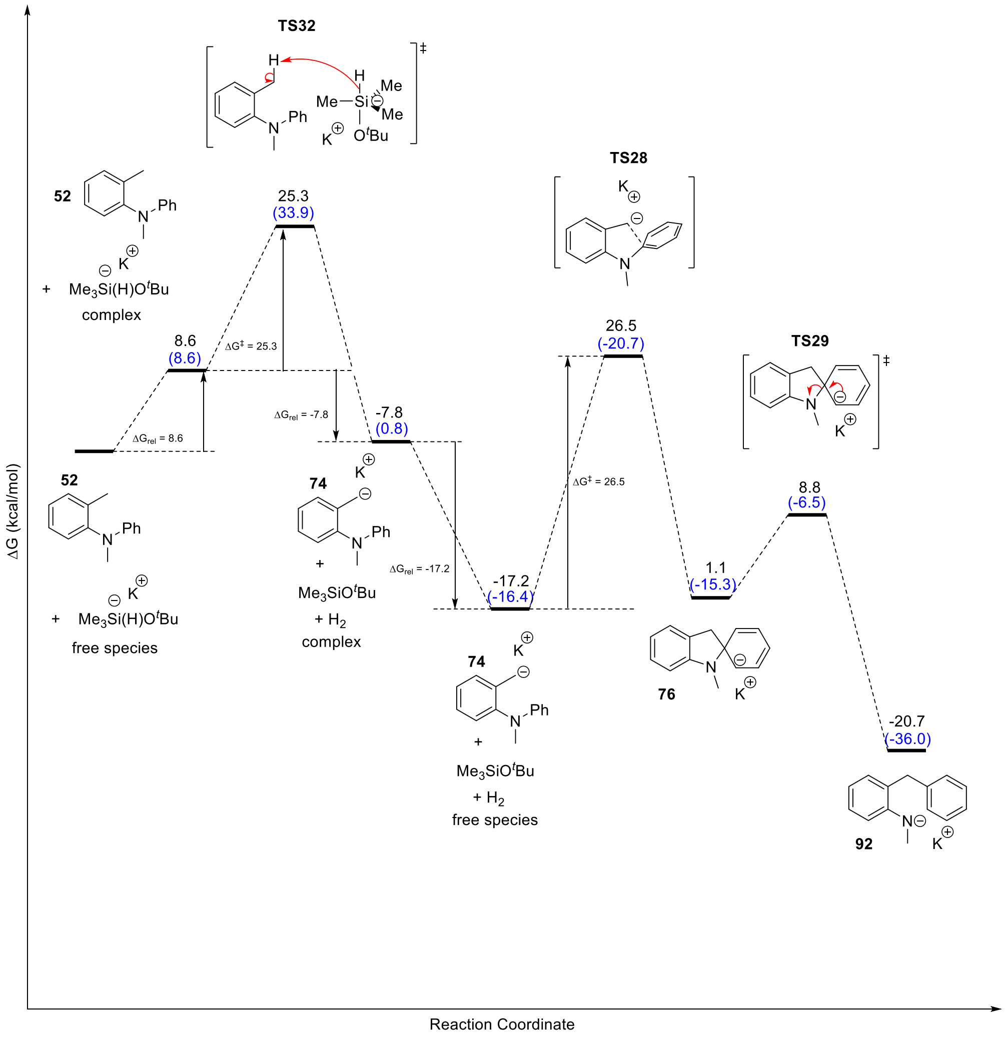

***Figure S15**. Reaction coordinate diagram displaying the progress of o-tolyl aryl amine **52** through a Truce-Smiles rearrangement featuring the initiation route consisting of a direct deprotonation of the methyl group of the o-tolyl ring by the pentavalent silicate*

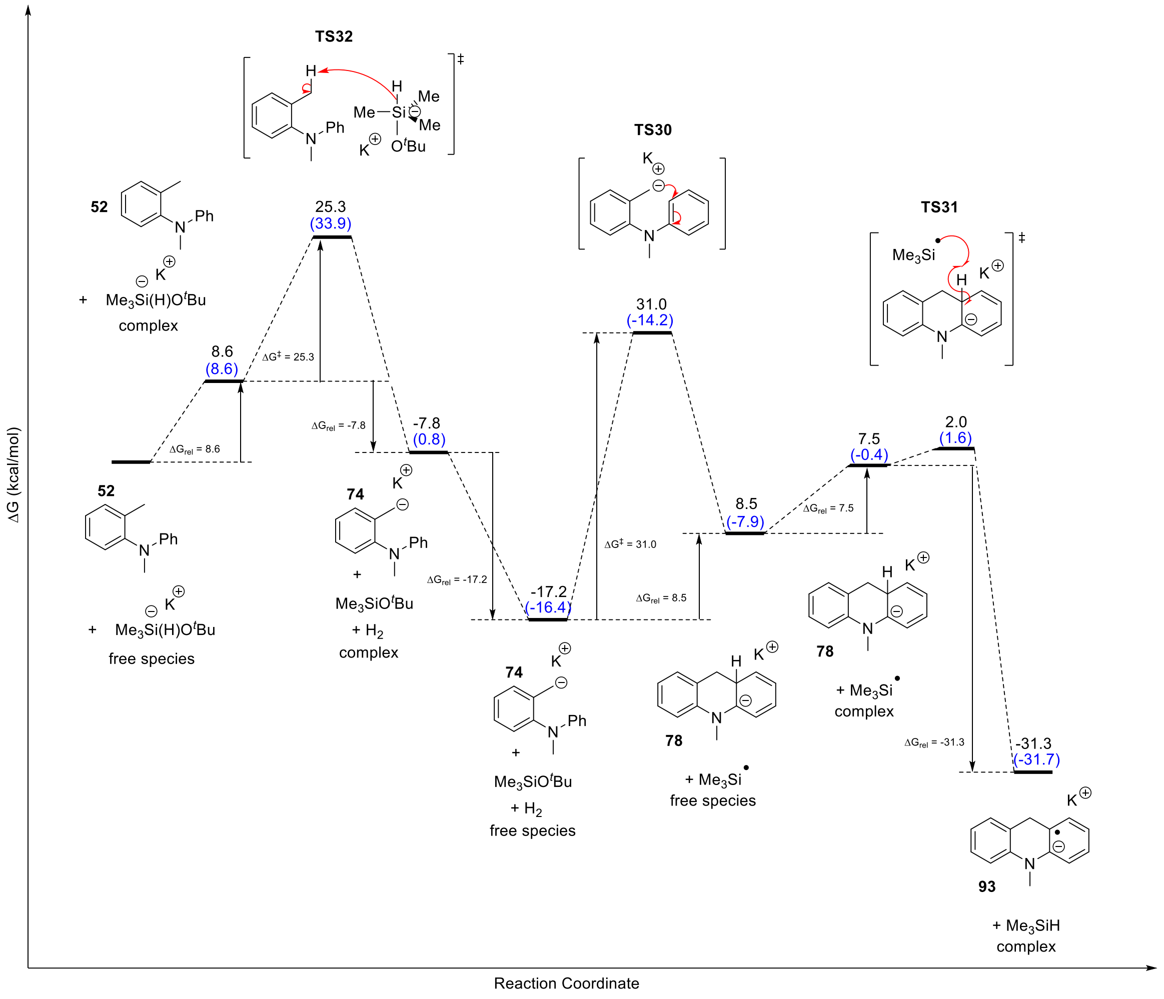

Figure S16 *Reaction coordinate diagram displaying the progress of diarylamine* ***52*** *through an anionic rearrangement mechanism featuring a 6-aryl cyclisation and the initiation route consisting of a direct deprotonation of the methyl group of the o-tolyl ring by the pentavalent silicate*

Additional Computational/Experimental Schemes

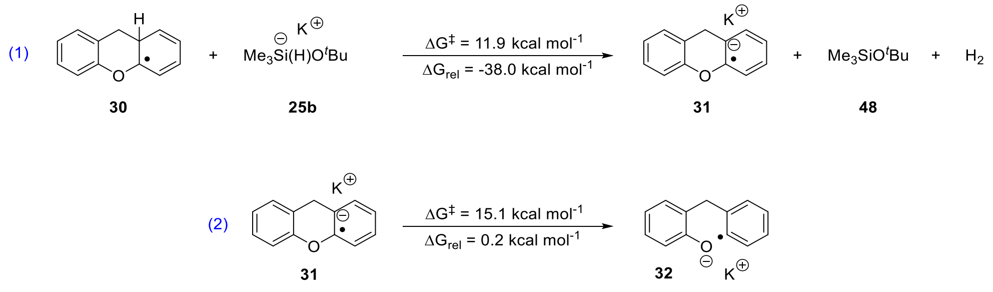

Scheme S1. *(1) Deprotonation of intermediate* ***30*** *via pentavalent silicate* ***25b*** *affording radical anion* ***31*** *(2) Mesolytic cleavage of C–O bond of intermediate* ***31***

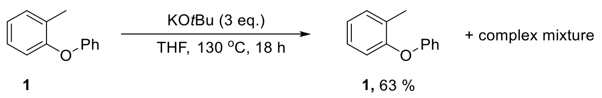

Scheme S2. *Control experiment; treatment o-tolyl aryl ether* ***1*** *with only KO^{t}Bu*

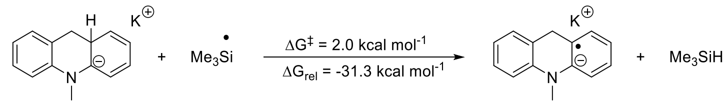

78 **24b** **93**

Scheme S3. *Hydrogen atom abstraction from intermediate* ***78*** *by a trimethyl silyl radical* ***24b*** *affording* Me_3SiH *and radical anion* ***93***

NMR Data

Substrates

^{1}H-NMR of 1-methyl-2-phenoxybenzene **1**

1

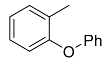

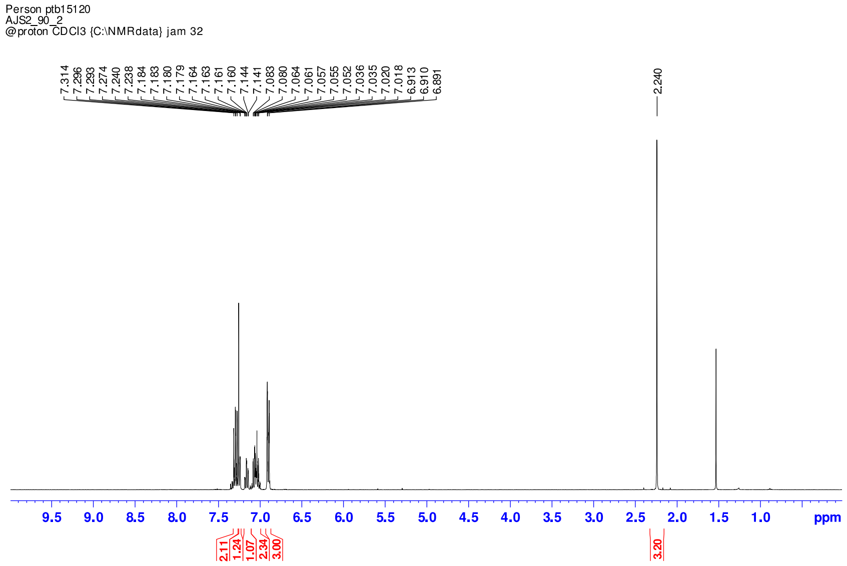

^{13}C-NMR of 1-methyl-2-phenoxybenzene **1**

1

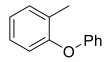

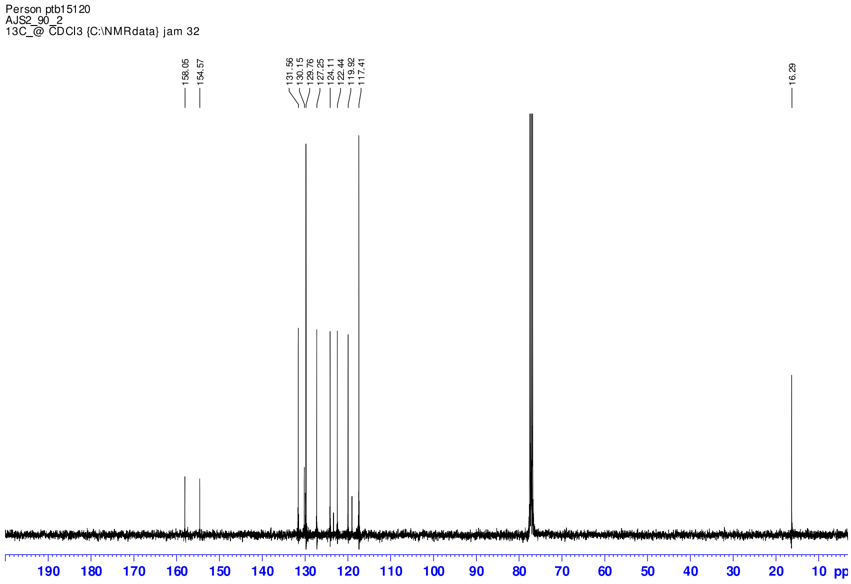

^{1}H-NMR of *N*,2-dimethyl-*N*-phenylaniline **52**

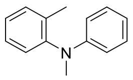

52

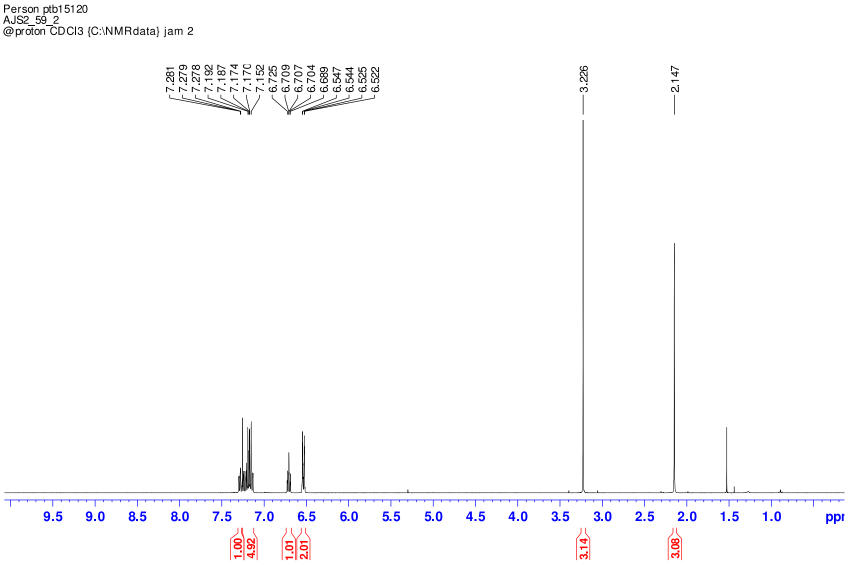

^{13}C-NMR of *N*,2-dimethyl-*N*-phenylaniline **52**

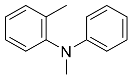

52

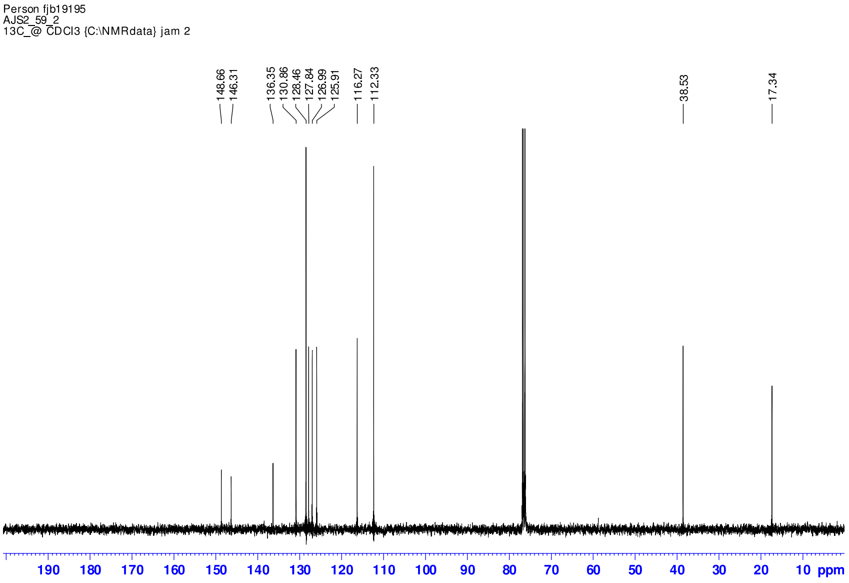

Products

^{1}H-NMR of 10-methyl-9,10-dihydroacridine **55**

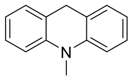

55

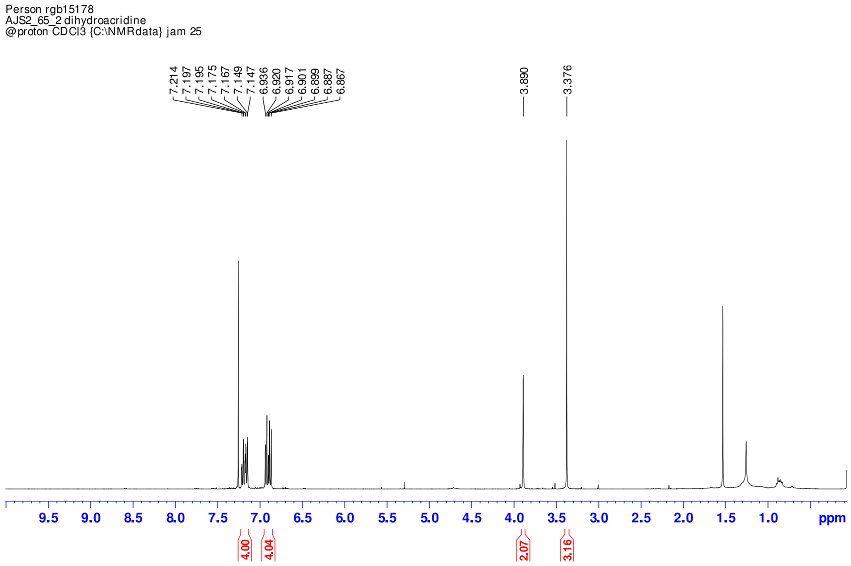

^{13}C-NMR of 10-methyl-9,10-dihydroacridine **55**

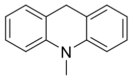

55

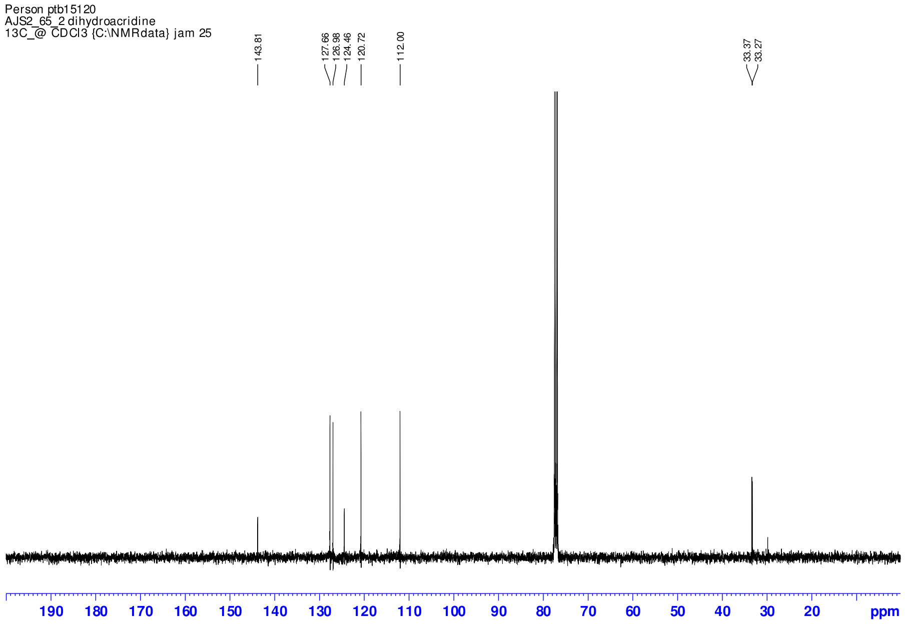

^{1}H-NMR 2-benzyl-*N*-methylaniline **56**

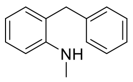

56

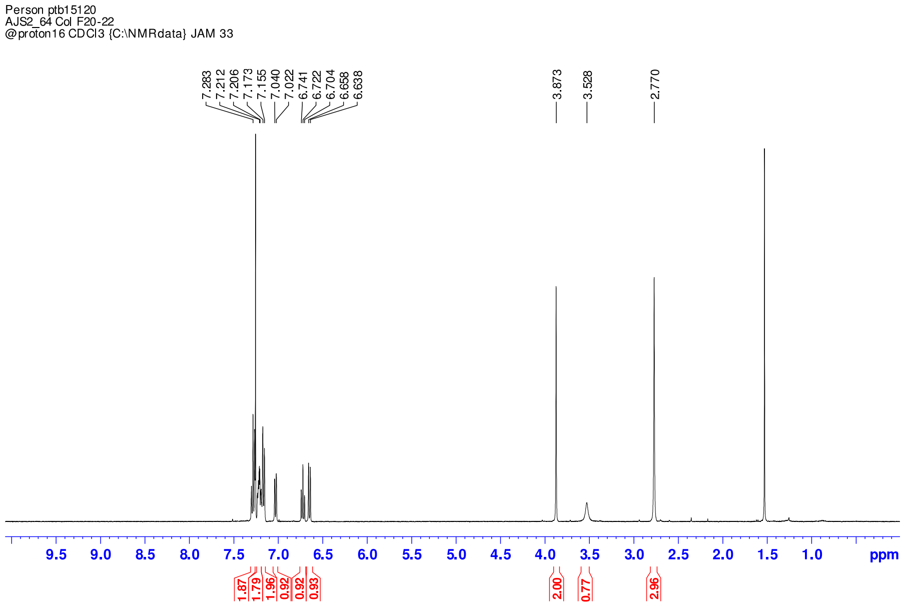

^{13}C-NMR 2-benzyl-*N*-methylaniline **56**

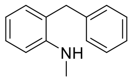

56

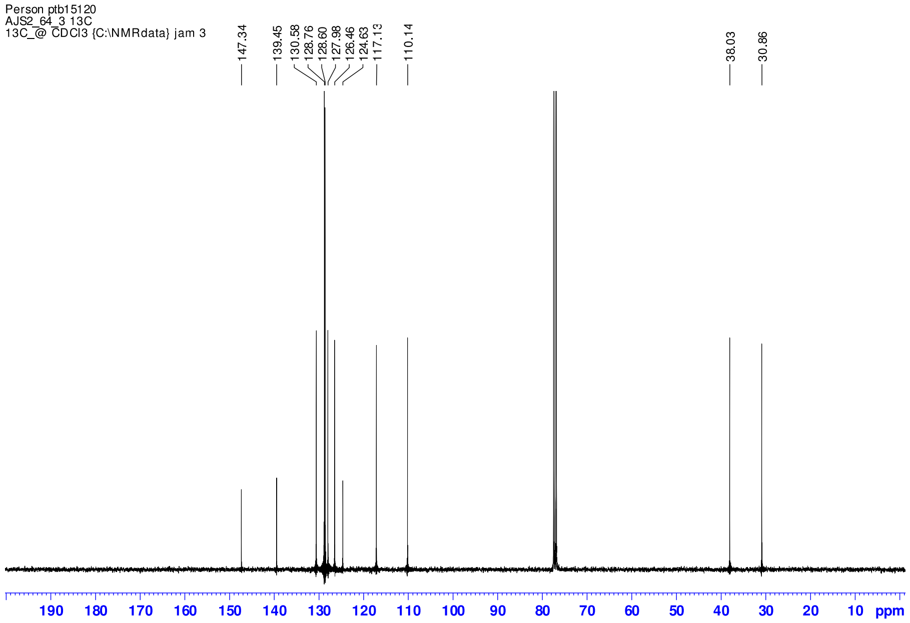

References

1. Zhao, Y.; Truhlar D. G. The M06 suite of density functionals for main group thermochemistry, thermochemical kinetics, noncovalent interactions, excited states, and transition elements: two new functionals and systematic testing of four M06-class functionals and 12 other functionals. *Theor. Chem. Acc*. **2008**, *120*, 215–241. doi:10.1007/s00214-007-0310-x
2. Zhao, Y.; Truhlar, D. G. Density Functionals with Broad Applicability in Chemistry. *Acc. Chem. Res*. **2008**, *41*, 157–167. doi:10.1021/ar700111a
3. Hariharan, P. C.; Pople, J. A. The influence of polarization functions on molecular orbital hydrogenation energies. *Theor. Chim. Acta* **1973**, *28*, 213–222. doi:10.1007/BF00533485
4. Hehre, W. J., Ditchfield, R.; Pople, Self-Consistent Molecular Orbital Methods. XII. Further Extensions of Gaussian—Type Basis Sets for Use in Molecular Orbital Studies of Organic MoleculesJ. A. *J. Chem. Phys*. **1972**, *56*, 2257–2261. doi:10.1063/1.1677527
5. Andersson, M. P.; Uvdal, P. New Scale Factors for Harmonic Vibrational Frequencies Using the B3LYP Density Functional Method with the Triple-ζ Basis Set 6-311+G(d,p). *J. Phys. Chem. A*. **2005**, *109*, 2937–2941. doi:10.1021/jp045733a
6. Barone, V.; Cossi, M.; Tomasi, J. A new definition of cavities for the computation of solvation free energies by the polarizable continuum model. *J. Chem. Phys*. **1997**, *107*, 3210–3221. doi:10.1063/1.474671
7. Altshuller, A. P.; Rosenblum, L. Dielectric Properties of Some Alkylsilanes. *J. Am. Chem. Soc*. **1955**, *77*, 272–274. doi:10.1021/ja01607a007
8. Gaussian 16, Revision C.01,Frisch, M. J.; Trucks, G. W.; Schlegel, H. B.; Scuseria, G. E.; Robb, M. A.; Cheeseman, J. R.; Scalmani, G.; Barone, V.; Petersson, G. A.; Nakatsuji, H.; Li, X. ; Caricato, M.; Marenich, A. V.; Bloino, J.; Janesko, B. G. ; Gomperts, R.; Mennucci, B.; Hratchian, H. P.; Ortiz, J. V.; Izmaylow, A. F.; Sonnenberg, J. L.; Williams-Young, D.; Ding, F.; Lipparini, F.; Edigi, F.; Goings, J.; Peng, B.; Petrone, A.; Henderson, T.; Ranasinghe, D.; Zakrzewski, V. G.; Gao, J.; Rega, N.; Zheng, G.; Liang, W.; Hada, M.; Ehara, M.; Toyota, K.; Fukuda, R.; Hasegawa, J.; Ishida, M.; Nakajima, T.; Honda, Y.; Kitao, O.; Nakai, H.; Vreven, T.; Throssell, K. ; Montgomery, J. A.; Peralta, J. J. E.; Ogliaro, F.; Bearpark, M. J.; Heyd, J. J.; Brothers, E. N.; Kudin, K. N.; Staroverov, V. N.; Keith, T. A.; Kobayashi, R.; Normand, J.; Raghavachari, K.; Rendell, A. P.; Burant, J. C.; Iyengar, S. S.; Tomasi, J.; Cossi, M.; Millam, J. M.; Klene, M.; Adamo, C.; Cammi, R.; Ochterski, J. W.; Martin, R. L.; Morokuma, K.; Farkas, O.; Foresman, J. B.; Fox, D. J., Gaussian, Inc., Wallingford CT, 2016. Available from www.gaussian.com

XYZ Coordinates

O-containing

Substrates

1-methyl-2-phenoxybenzene **1**

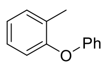

1

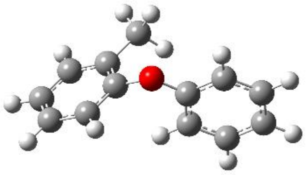

C	1.87942	-1.41578	-0.49269
C	1.11782	-0.25944	-0.57612
C	1.56702	0.96513	-0.07408
C	2.82180	0.98326	0.53484
C	3.59855	-0.16793	0.63518
C	3.12934	-1.37008	0.11699
H	1.48182	-2.33446	-0.90719
H	3.19696	1.92207	0.92833
H	4.57095	-0.12252	1.11053
H	3.73003	-2.26889	0.18469
C	0.72278	2.20416	-0.19569
H	-0.10311	2.18858	0.52133
H	1.32248	3.09458	-0.00734
H	0.28335	2.27819	-1.19245
O	-0.09998	-0.32276	-1.23164
C	-1.24719	-0.25560	-0.47838
C	-2.42728	-0.01953	-1.18032
C	-1.26543	-0.43304	0.90221
C	-3.63182	0.04159	-0.49339
H	-2.37867	0.11316	-2.25427
C	-2.48238	-0.36431	1.57723
H	-0.34567	-0.61944	1.44303
C	-3.66700	-0.12813	0.88975
H	-4.54798	0.22596	-1.04194

H	-2.49635	-0.50052	2.65223
H	-4.60787	-0.07634	1.42319

KO*t*Bu

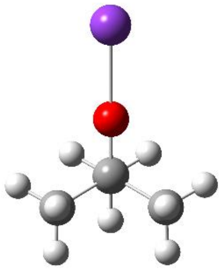

C	1.08820	-0.00002	0.00012
C	1.62899	1.19274	-0.81442
H	1.26016	1.12807	-1.84243
H	1.26271	2.12704	-0.37845
H	2.72376	1.22464	-0.83820
C	1.63444	0.10802	1.43833
H	1.26961	1.03137	1.89831
H	1.26895	-0.73610	2.03086
H	2.72936	0.11026	1.47335
C	1.62924	-1.30086	-0.62730
H	1.26308	-2.15939	-0.05643
H	1.26072	-1.39098	-1.65364
H	2.72401	-1.33576	-0.64557
O	-0.28665	0.00021	0.00312
K	-2.59760	-0.00001	0.00036

Me_3SiH

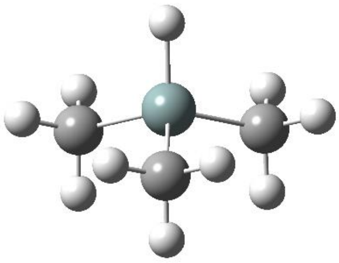

Si	0.00000	0.00000	0.38453
C	0.00000	1.77726	-0.22514
H	0.88405	2.31522	0.12511
H	0.00000	1.80579	-1.31817
H	-0.88405	2.31522	0.12511
C	-1.53915	-0.88863	-0.22514
H	-1.56301	-1.92322	0.12511
H	-2.44706	-0.39200	0.12511
H	-1.56386	-0.90289	-1.31817
C	1.53915	-0.88863	-0.22514
H	1.56301	-1.92322	0.12511
H	1.56386	-0.90289	-1.31817
H	2.44706	-0.39200	0.12511
H	0.00000	0.00000	1.87300

Intermediates

Me_3Si radical **24b**

$Me_3Si^{\bullet}$

24b

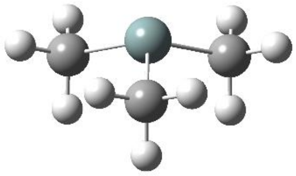

Si	0.00025	-0.00017	-0.43349
C	-0.52548	1.70296	0.17999
H	-1.52934	1.95702	-0.16672
H	-0.53121	1.72079	1.27547
H	0.16028	2.47903	-0.16631

C	1.73808	-0.39664	0.18013
H	2.06815	-1.37766	-0.16783
H	2.45969	0.34683	-0.16490
H	1.75600	-0.40254	1.27561
C	-1.21285	-1.30622	0.18003
H	-0.93092	-2.30345	-0.16414
H	-1.22761	-1.31788	1.27552
H	-2.22702	-1.10041	-0.16867

1-methyl-2-phenoxybenzene **1** + Me_3Si radical **24b** complex

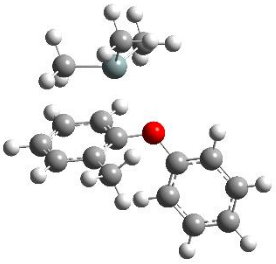

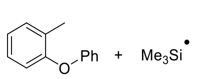

1 24b

C	-0.39345	1.67689	-1.46928
C	0.04299	0.91156	-0.39636
C	-0.19111	1.29112	0.92978
C	-0.87249	2.49073	1.13742
C	-1.30804	3.27752	0.07469
C	-1.07366	2.86772	-1.23336
H	-0.19040	1.32820	-2.47504
H	-1.07469	2.80363	2.15645
H	-1.83861	4.20173	0.26953
H	-1.41603	3.46830	-2.06730
C	0.27226	0.43938	2.07987
H	1.32509	0.6291	2.30958
H	-0.31735	0.65508	2.97165
H	0.17229	-0.62169	1.84111
O	0.66550	-0.29169	-0.67491
C	1.99489	-0.43358	-0.35902
C	2.47192	-1.74058	-0.29475

C	2.84573	0.64623	-0.14217
C	3.81171	-1.96623	-0.01004
H	1.78072	-2.55693	-0.46771
C	4.18643	0.40285	0.14772
H	2.46744	1.65992	-0.19625
C	4.67645	-0.89640	0.21513
H	4.18129	-2.98362	0.04015
H	4.84957	1.24270	0.31880
H	5.72022	-1.07570	0.44078
Si	-2.55206	-1.41784	0.12792
C	-3.55613	0.17239	0.24122
H	-3.74107	0.45371	1.28078
H	-3.02676	0.99734	-0.24239
H	-4.52518	0.04587	-0.25444
C	-2.14155	-1.79217	-1.67213
H	-1.55160	-2.70747	-1.75977
H	-3.06079	-1.92140	-2.25390
H	-1.56450	-0.97685	-2.11322
C	-3.52874	-2.85100	0.87441
H	-4.46679	-2.99217	0.32593
H	-3.77605	-2.66007	1.92101
H	-2.96503	-3.78513	0.82375

1-methyl-2-phenoxybenzene radical **27** + Me_3SiH complex

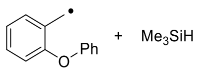

27

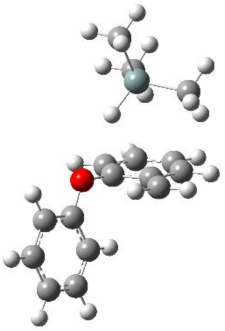

C	0.08403	1.77530	-1.42889
C	0.29518	0.82451	-0.44987

C	-0.17676	0.99550	0.87809
C	-0.86968	2.20150	1.15401
C	-1.07696	3.15740	0.17603
C	-0.60501	2.94931	-1.12243
H	0.46428	1.58749	-2.42596
H	-1.24143	2.36162	2.16052
H	-1.61135	4.06811	0.41813
H	-0.77076	3.69347	-1.89135
C	0.02888	0.01497	1.87019
H	0.52881	-0.91629	1.64635
H	-0.32843	0.18118	2.87743
H	-1.51150	-1.64249	-0.13004
O	0.94941	-0.34082	-0.79001
C	2.25904	-0.49849	-0.39950
C	2.80559	-1.76294	-0.60862
C	3.02194	0.52350	0.15744
C	4.12642	-2.00351	-0.25696
H	2.18343	-2.53540	-1.04401
C	4.34577	0.26429	0.50653
H	2.59321	1.50483	0.31763
C	4.90454	-0.99194	0.30376
H	4.54945	-2.98757	-0.42090
H	4.94048	1.05932	0.94076
H	5.93427	-1.18316	0.57863
Si	-2.94574	-1.25780	-0.05553
C	-3.26471	0.19841	-1.19760
H	-2.71900	1.08401	-0.86242
H	-2.95294	-0.02370	-2.22125
H	-4.33116	0.44156	-1.21340
C	-3.99013	-2.72994	-0.58720
H	-3.82518	-3.58934	0.06731
H	-5.05362	-2.47820	-0.55023

H	-3.75188	-3.03423	-1.60929
C	-3.38778	-0.78457	1.71097
H	-4.47033	-0.66191	1.80852
H	-2.91406	0.15827	1.99392
H	-3.06938	-1.55157	2.42149

Intermediate **27**

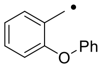

27

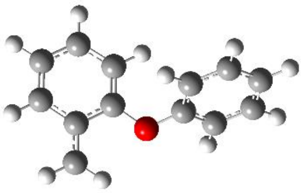

C	-3.42635	0.06515	0.21440
C	-2.25113	0.86068	0.17662
C	-1.04594	0.20425	-0.20093
C	-1.02302	-1.14300	-0.52024
C	-2.19994	-1.88925	-0.46505
C	-3.40256	-1.28013	-0.09534
H	-4.35630	0.54433	0.50007
H	-0.09063	-1.60965	-0.81383
H	-2.17508	-2.94204	-0.71756
H	-4.31502	-1.86220	-0.05390
O	0.06581	1.00290	-0.28444
C	-2.27236	2.22786	0.49955
H	-3.20297	2.70107	0.78208
H	-1.37135	2.82209	0.47467
C	1.31527	0.44966	-0.09279
C	1.60187	-0.30106	1.04359
C	2.29568	0.72970	-1.03450
C	2.89276	-0.78153	1.22769
H	0.81969	-0.50118	1.76659
C	3.58681	0.24852	-0.83523
H	2.03711	1.32093	-1.90429

C	3.88829	-0.50966	0.29139
H	3.12286	-1.36505	2.11112
H	4.35599	0.46626	-1.56651
H	4.89290	-0.88495	0.44227

Intermediate **28**

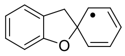

28

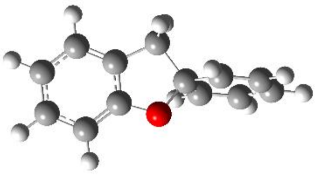

C	-1.22469	-0.61645	-0.22213
C	-1.32337	0.76776	-0.11397
C	-2.55167	1.35515	0.13724
C	-3.67616	0.53646	0.27513
C	-3.55792	-0.84651	0.15673
C	-2.32373	-1.44951	-0.09253
C	0.04348	1.34568	-0.36705
H	-2.64134	2.43225	0.22306
H	-4.64393	0.97914	0.47580
H	-4.43733	-1.47013	0.26650
H	-2.21911	-2.52352	-0.17899
H	0.33809	2.12371	0.33765
H	0.11377	1.75436	-1.37965
C	0.95791	0.08749	-0.22446
C	2.04331	0.03768	-1.24766
C	1.46892	-0.04109	1.17840
C	3.35807	-0.03769	-0.91373
H	1.72592	0.07351	-2.28418
C	2.79320	-0.11994	1.46905
H	0.71784	-0.06103	1.96226
C	3.76840	-0.10727	0.44082
H	4.10842	-0.05425	-1.69576
H	3.11267	-0.20226	2.50155

H	4.81980	-0.17152	0.68750
O	0.04383	-1.04384	-0.47134

Intermediate **29**

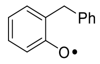

29

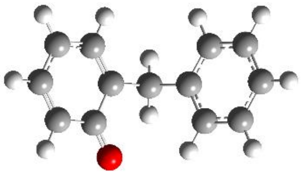

C	-1.66842	-1.46068	0.31040
C	-1.13544	-0.24179	0.67189
C	-1.75465	0.98414	0.16927
C	-2.90719	0.85143	-0.70198
C	-3.40062	-0.37907	-1.03591
C	-2.78552	-1.54329	-0.53022
H	-1.21484	-2.37317	0.68284
H	-3.35319	1.76964	-1.06534
H	-4.26290	-0.46866	-1.68538
H	-3.18308	-2.51558	-0.79481
O	-1.31315	2.10558	0.48473
C	0.09488	-0.12763	1.52889
C	1.34385	-0.07645	0.66607
C	1.77168	1.12819	0.10505
C	2.06121	-1.24187	0.39503
C	2.90025	1.16443	-0.70788
H	1.21052	2.03388	0.30755
C	3.18994	-1.20704	-0.41887
H	1.73908	-2.18253	0.83047
C	3.61217	-0.00254	-0.97292
H	3.22534	2.10650	-1.13389
H	3.74102	-2.11929	-0.61576
H	4.49170	0.02740	-1.60507
H	0.02906	0.77933	2.13139
H	0.14957	-0.98906	2.19730

Silyl electron donor **26b**

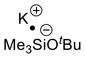

26b

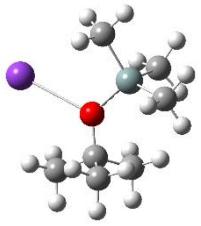

Si	1.28077	-0.59302	-0.00238
O	-0.23075	0.14714	-0.12215
C	-0.55689	1.54734	-0.01308
C	0.21241	2.19101	1.13933
C	-0.24196	2.23648	-1.33933
C	-2.05417	1.62551	0.26618
H	0.00666	1.66257	2.07363
H	1.29021	2.17848	0.95753
H	-0.09363	3.23316	1.25376
H	-0.75453	1.71873	-2.15358
H	-0.58322	3.27423	-1.31539
H	0.82988	2.23383	-1.54257
H	-2.36697	2.66706	0.37095
H	-2.62334	1.18417	-0.55785
H	-2.30016	1.09783	1.19279
C	1.80868	-0.73325	1.78866
H	2.63760	-1.44262	1.87330
H	0.97986	-1.11399	2.39391
C	2.59454	0.29406	-1.00244
H	3.52031	-0.28691	-0.94459
H	2.81434	1.29808	-0.63286
C	1.03929	-2.30819	-0.71077
H	2.01740	-2.77101	-0.87768
H	0.51522	-2.27784	-1.67086
H	2.13420	0.21837	2.21354
H	0.48230	-2.96471	-0.03582
H	2.31275	0.36760	-2.05573

K	-2.30079	-1.69840	-0.01596

Silyl intermediate **44**

$K^{\oplus}$
Me_3SiO^tBu
44

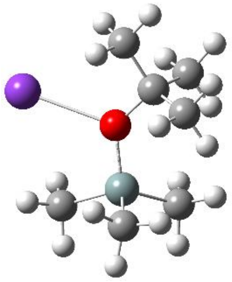

Si	1.39350	-0.24154	-0.00240
O	-0.27759	0.04588	-0.01344
C	-0.96572	1.32088	0.00361
C	-0.28866	2.28215	0.97620
C	-0.98584	1.89573	-1.40984
C	-2.38846	1.02713	0.47012
H	-0.23365	1.84595	1.97622
H	0.72197	2.53465	0.64541
H	-0.85788	3.21180	1.03345
H	-1.41917	1.17345	-2.10564
H	-1.58934	2.80568	-1.43592
H	0.02015	2.14584	-1.74887
H	-2.95689	1.95348	0.56738
H	-2.91724	0.40490	-0.25909
H	-2.37842	0.53403	1.44604
C	2.13247	0.07801	1.68369
H	3.11656	-0.39673	1.73927
H	1.51219	-0.35057	2.47527
C	2.28651	0.72704	-1.32485
H	3.32823	0.39434	-1.35916
H	2.29528	1.80113	-1.12599
C	1.49793	-2.07767	-0.37401
H	2.54910	-2.35749	-0.48511
H	1.00371	-2.34450	-1.31305

H	2.26578	1.14053	1.89304
H	1.10570	-2.69754	0.43870
H	1.85000	0.56239	-2.31273
K	-1.70704	-2.15086	-0.00409

Single point energy calculation of silyl electron donor **26b** as a cation

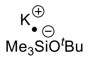

26b

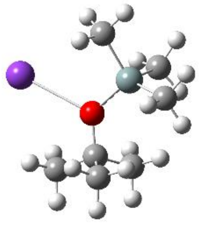

Si	1.28077	-0.59302	-0.00238
O	-0.23075	0.14714	-0.12215
C	-0.55689	1.54734	-0.01308
C	0.21241	2.19101	1.13933
C	-0.24196	2.23648	-1.33933
C	-2.05417	1.62551	0.26618
H	0.00666	1.66257	2.07363
H	1.29021	2.17848	0.95753
H	-0.09363	3.23316	1.25376
H	-0.75453	1.71873	-2.15358
H	-0.58322	3.27423	-1.31539
H	0.82988	2.23383	-1.54257
H	-2.36697	2.66706	0.37095
H	-2.62334	1.18417	-0.55785
H	-2.30016	1.09783	1.19279
C	1.80868	-0.73325	1.78866
H	2.63760	-1.44262	1.87330
H	0.97986	-1.11399	2.39391
C	2.59454	0.29406	-1.00244
H	3.52031	-0.28691	-0.94459
H	2.81434	1.29808	-0.63286
C	1.03929	-2.30819	-0.71077

H	2.01740	-2.77101	-0.87768
H	0.51522	-2.27784	-1.67086
H	2.13420	0.21837	2.21354
H	0.48230	-2.96471	-0.03582
H	2.31275	0.36760	-2.05573
K	-2.30079	-1.69840	-0.01596

Single point energy calculation of silyl cation **44** as a neutral species

$K^{\oplus}$

Me_3SiO^tBu

44

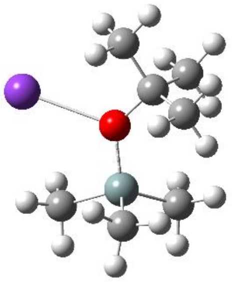

Si	1.39350	-0.24154	-0.00240
O	-0.27759	0.04588	-0.01344
C	-0.96572	1.32088	0.00361
C	-0.28866	2.28215	0.97620
C	-0.98584	1.89573	-1.40984
C	-2.38846	1.02713	0.47012
H	-0.23365	1.84595	1.97622
H	0.72197	2.53465	0.64541
H	-0.85788	3.21180	1.03345
H	-1.41917	1.17345	-2.10564
H	-1.58934	2.80568	-1.43592
H	0.02015	2.14584	-1.74887
H	-2.95689	1.95348	0.56738
H	-2.91724	0.40490	-0.25909
H	-2.37842	0.53403	1.44604
C	2.13247	0.07801	1.68369
H	3.11656	-0.39673	1.73927
H	1.51219	-0.35057	2.47527
C	2.28651	0.72704	-1.32485

H	3.32823	0.39434	-1.35916
H	2.29528	1.80113	-1.12599
C	1.49793	-2.07767	-0.37401
H	2.54910	-2.35749	-0.48511
H	1.00371	-2.34450	-1.31305
H	2.26578	1.14053	1.89304
H	1.10570	-2.69754	0.43870
H	1.85000	0.56239	-2.31273
K	-1.70704	-2.15086	-0.00409

Intermediate **30**

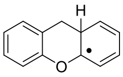

30

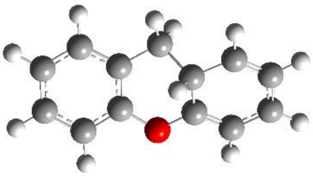

C	-3.64568	0.64994	-0.08417
C	-2.46019	1.35918	-0.23726
C	-1.22119	0.72170	-0.18969
C	-1.20484	-0.65745	0.02930
C	-2.38065	-1.38331	0.19276
C	-3.60138	-0.72603	0.13212
C	0.08556	1.45609	-0.33179
C	1.16298	-0.72625	0.06960
C	1.15890	0.71000	0.48794
C	2.52429	1.32061	0.38834
H	2.60449	2.37590	0.62635
C	3.62406	0.60388	0.03892
C	3.54049	-0.77798	-0.27689
C	2.28369	-1.42018	-0.26953
H	0.39995	1.49436	-1.38000
H	-4.59664	1.16612	-0.12717
H	-2.48580	2.43236	-0.39544
H	-2.31648	-2.45129	0.36131

H	-4.51842	-1.28902	0.25688
H	4.58926	1.09588	-0.00585
H	4.42824	-1.33164	-0.55004
H	2.18671	-2.46327	-0.54692
O	-0.03609	-1.38096	0.06283
H	-0.01411	2.48457	0.02279
H	0.81526	0.75244	1.53760

Intermediate **30** and KO^{t}Bu complex

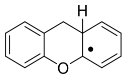

30

+ KO^{t}Bu complex

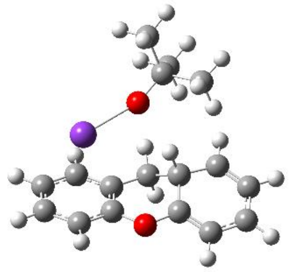

C	3.46263	1.15513	1.10444
C	2.28391	0.65550	1.65203
C	1.55091	-0.34890	1.01911
C	2.02599	-0.82969	-0.20722
C	3.20459	-0.33976	-0.77170
C	3.92462	0.64840	-0.11092
C	0.27756	-0.91342	1.58667
C	0.14876	-2.24538	-0.49702
C	-0.62702	-1.35806	0.41907
C	-1.89622	-2.01791	0.86321
H	-2.49546	-1.48434	1.59312
C	-2.32199	-3.21063	0.36845
C	-1.55703	-3.93366	-0.58282
C	-0.29913	-3.43286	-0.98552
H	0.49761	-1.76782	2.23578
H	4.01844	1.92660	1.62277
H	1.91492	1.04693	2.59422
H	3.53937	-0.75194	-1.71609

H	4.84563	1.01978	-0.54413
H	-3.26377	-3.62459	0.71123
H	-1.91254	-4.87835	-0.97082
H	0.33187	-3.98889	-1.66920
O	1.38756	-1.81227	-0.90789
H	-0.23492	-0.15424	2.18140
H	-0.86471	-0.40621	-0.10718
C	-2.35364	2.01194	-0.07703
C	-2.72117	3.46753	-0.43074
H	-2.08266	4.15273	0.13559
H	-2.54573	3.63776	-1.49747
H	-3.76746	3.70438	-0.20877
C	-3.28186	1.07382	-0.87623
H	-3.10685	1.21565	-1.94691
H	-3.06302	0.02993	-0.63147
H	-4.34132	1.26042	-0.66938
C	-2.62220	1.80026	1.42651
H	-2.38321	0.76801	1.69723
H	-1.97347	2.46226	2.00856
H	-3.66369	1.99784	1.70250
O	-1.03132	1.76301	-0.37090
K	1.18468	2.16496	-1.03645

Intermediate **31** and HO^{t}Bu complex

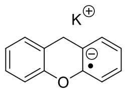

31

+ ^{t}BuOH

complex

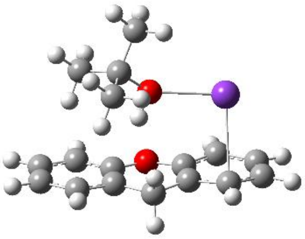

C	3.81808	-0.51147	0.81055
C	2.59138	-0.48224	1.52926
C	1.39994	-0.96476	0.87206
C	1.45982	-1.26497	-0.46744

C	2.64070	-1.12643	-1.24212
C	3.84853	-0.82157	-0.53334
C	0.14046	-1.13227	1.66952
C	-0.85760	-1.76032	-0.56670
C	-1.03327	-1.48410	0.79202
C	-2.33169	-1.55587	1.30992
H	-2.47613	-1.34475	2.36537
C	-3.41718	-1.89738	0.51545
C	-3.21594	-2.17232	-0.83812
C	-1.94146	-2.10033	-1.37891
H	0.27621	-1.90257	2.44156
H	4.74492	-0.28143	1.32688
H	2.57595	-0.33749	2.60274
H	2.63941	-1.45448	-2.27236
H	4.79437	-0.83608	-1.06404
H	-4.41067	-1.95074	0.94345
H	-4.05296	-2.44013	-1.47179
H	-1.75538	-2.30561	-2.42621
O	0.35620	-1.71283	-1.18288
H	-0.07326	-0.20108	2.22189
H	-0.53344	0.66739	-0.05469
C	-1.56995	2.33912	-0.04365
C	-1.25832	3.75814	-0.49522
H	-0.44373	4.17552	0.10228
H	-0.96487	3.76448	-1.54741
H	-2.13533	4.39690	-0.37495
C	-2.66838	1.71156	-0.89633
H	-2.37797	1.71499	-1.94933
H	-2.84641	0.67663	-0.58694
H	-3.60331	2.26624	-0.78769
C	-1.94054	2.30110	1.43644
H	-2.13600	1.27165	1.75036

H	-1.12269	2.70129	2.04055
H	-2.83994	2.89200	1.62508
O	-0.34986	1.59887	-0.23726
K	2.24753	1.70651	-0.44022

Intermediate **31**

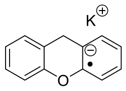

31

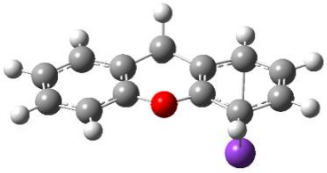

C	4.06773	0.56340	0.36940
C	2.93599	1.33569	0.14178
C	1.70618	0.75876	-0.18362
C	1.64023	-0.63255	-0.26324
C	2.76746	-1.42265	-0.03802
C	3.97959	-0.82505	0.27470
C	0.49057	1.60331	-0.46154
C	-0.70234	-0.60041	-0.61981
C	-0.75462	0.77078	-0.55837
C	-2.04539	1.42182	-0.60958
H	-2.08577	2.50251	-0.67466
C	-3.18965	0.62758	-0.89755
C	-3.10825	-0.74860	-0.94877
C	-1.86073	-1.41905	-0.71593
H	0.37434	2.35682	0.33117
H	5.01014	1.03700	0.61582
H	2.99627	2.41775	0.20805
H	2.66800	-2.49879	-0.11503
H	4.85350	-1.44210	0.44639
H	-4.14465	1.11249	-1.07518
H	-3.99437	-1.33513	-1.16595
H	-1.74365	-2.48529	-0.85274
O	0.49051	-1.30469	-0.56288

C	-3.18965	0.62758	-0.89755
C	-3.10825	-0.74860	-0.94877
C	-1.86073	-1.41905	-0.71593
H	0.37434	2.35682	0.33117
H	5.01014	1.03700	0.61582
H	2.99627	2.41775	0.20805
H	2.66800	-2.49879	-0.11503
H	4.85350	-1.44210	0.44639
H	-4.14465	1.11249	-1.07518
H	-3.99437	-1.33513	-1.16595
H	-1.74365	-2.48529	-0.85274
O	0.49051	-1.30469	-0.56288
H	0.63657	2.17534	-1.38895
K	-2.31869	-0.10533	1.85459

Intermediate **32**

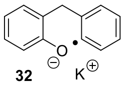

32

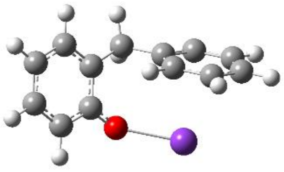

C	3.81414	0.94023	-0.38219
C	2.67024	1.43418	0.24491
C	1.59385	0.61223	0.55944
C	1.62061	-0.78127	0.23630
C	2.79871	-1.25812	-0.39719
C	3.86215	-0.41826	-0.69622
C	0.37366	1.15219	1.25564
C	-2.15368	1.03948	1.05736
C	-0.91175	1.03489	0.45634
C	-0.93203	0.90507	-0.94099
H	0.01489	0.88043	-1.47142
C	-2.13494	0.77458	-1.63505
C	-3.35620	0.77201	-0.96082

C	-3.37237	0.91324	0.43297
H	0.21529	0.62291	2.20110
H	4.64374	1.59616	-0.61522
H	2.61356	2.48927	0.50118
H	2.84338	-2.31431	-0.64297
H	4.74277	-0.82824	-1.18114
H	-2.11819	0.67227	-2.71386
H	-4.28699	0.67298	-1.50745
H	-4.30596	0.92884	0.98420
O	0.61424	-1.54884	0.50099
C	-2.13494	0.77458	-1.63505
C	-3.35620	0.77201	-0.96082
C	-3.37237	0.91324	0.43297
H	0.21529	0.62291	2.20110
H	4.64374	1.59616	-0.61522
H	2.61356	2.48927	0.50118
H	2.84338	-2.31431	-0.64297
H	4.74277	-0.82824	-1.18114
H	-2.11819	0.67227	-2.71386
H	-4.28699	0.67298	-1.50745
H	-4.30596	0.92884	0.98420
O	0.61424	-1.54884	0.50099
H	0.52611	2.20730	1.50446
K	-1.73878	-1.96104	0.18685

Intermediate **40**

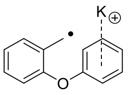

40

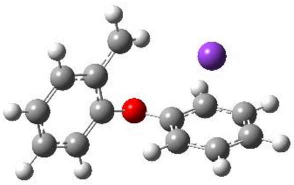

C	-2.96657	1.22233	0.24593
C	-1.80016	0.83674	-0.46258
C	-1.55052	-0.55609	-0.56732

C	-2.41422	-1.50066	-0.05627
C	-3.56467	-1.08404	0.61612
C	-3.82871	0.27942	0.77252
H	-3.18617	2.27953	0.34464
H	-2.17993	-2.55130	-0.17926
H	-4.24726	-1.82135	1.01919
H	-4.71994	0.59871	1.29825
O	-0.40506	-0.96265	-1.23080
C	-0.94687	1.78286	-1.06824
H	-1.17425	2.83887	-0.99864
H	-0.17434	1.46441	-1.75720
C	0.76909	-0.93048	-0.52871
C	0.82580	-0.96295	0.86604
C	1.94233	-0.88735	-1.28476
C	2.07091	-0.97119	1.49600
H	-0.08525	-1.00629	1.44994
C	3.17512	-0.89078	-0.64139
H	1.86723	-0.87081	-2.36546
C	3.24793	-0.93022	0.75252
H	2.11309	-1.02103	2.57773
H	4.08277	-0.87155	-1.23275
H	4.20979	-0.95050	1.24924
K	1.94621	1.95616	0.40008

Intermediate **41**

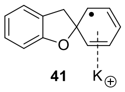

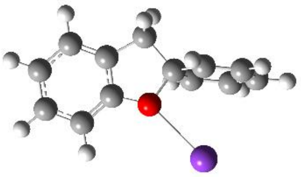

C	1.46840	0.42046	-0.18512
C	1.84942	-0.91339	-0.13853
C	3.17818	-1.23937	0.07853
C	4.10561	-0.20799	0.24170

C	3.70076	1.12315	0.18368
C	2.36343	1.46278	-0.03028
C	0.63631	-1.76659	-0.40179
H	3.49344	-2.27549	0.11814
H	5.14772	-0.44498	0.41489
H	4.43195	1.91208	0.31127
H	2.04071	2.49542	-0.07401
H	0.53105	-2.60948	0.28174
H	0.64249	-2.15394	-1.42437
C	-0.52675	-0.75242	-0.21153
C	-1.60501	-0.86724	-1.23988
C	-1.04870	-0.76981	1.19436
C	-2.92172	-0.96702	-0.90927
H	-1.28454	-0.87266	-2.27610
C	-2.37551	-0.86430	1.48093
H	-0.30607	-0.70298	1.98375
C	-3.34118	-0.96675	0.44630
H	-3.66485	-1.06067	-1.69275
H	-2.70284	-0.87589	2.51412
H	-4.39116	-1.05947	0.68979
O	0.11877	0.57617	-0.40585
K	-1.98882	2.15208	-0.03444

Intermediate **42**

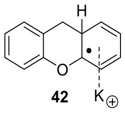

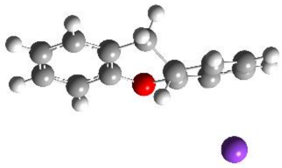

C	-4.25760	0.21206	0.50322
C	-3.20569	1.12027	0.50260
C	-1.92806	0.74417	0.09066
C	-1.73890	-0.57806	-0.31078
C	-2.77396	-1.50398	-0.31211

C	-4.03857	-1.10203	0.09559
C	-0.75271	1.68653	0.09467
C	0.57807	-0.24397	-0.64733
C	0.52064	0.85376	0.37130
C	1.78527	1.66033	0.39457
H	1.82379	2.48676	1.09591
C	2.82680	1.42243	-0.44964
C	2.79369	0.35389	-1.38889
C	1.65039	-0.47294	-1.46467
H	-0.64701	2.19411	-0.86933
H	-5.24282	0.52582	0.82422
H	-3.36929	2.14212	0.82755
H	-2.57548	-2.51719	-0.63907
H	-4.85269	-1.81613	0.09466
H	3.69841	2.06676	-0.41640
H	3.61516	0.20376	-2.07654
H	1.58119	-1.26586	-2.20042
O	-0.51467	-1.03165	-0.77549
H	-0.87524	2.45107	0.86434
H	0.34518	0.39848	1.36277
K	3.25579	-1.23849	1.18216

Intermediate **43**

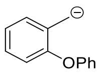

43

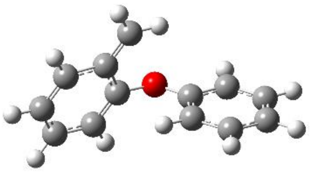

C	-2.91087	1.03358	0.26046
C	-1.61015	0.98614	-0.36605
C	-1.11440	-0.35894	-0.49596
C	-1.80618	-1.48140	-0.10375
C	-3.07475	-1.38600	0.48541
C	-3.59916	-0.09433	0.65794

H	-3.35908	2.01219	0.40802
H	-1.33458	-2.44672	-0.26480
H	-3.61752	-2.26922	0.79569
H	-4.57752	0.02760	1.11570
O	0.12601	-0.54885	-1.12732
C	-0.91126	2.10560	-0.78364
H	-1.34275	3.09188	-0.66075
H	0.06101	2.02680	-1.25102
C	1.25634	-0.29738	-0.41743
C	1.26069	0.08877	0.92447
C	2.46502	-0.46053	-1.09888
C	2.47565	0.30002	1.56904
H	0.31910	0.22953	1.43971
C	3.66939	-0.24624	-0.44142
H	2.43405	-0.75521	-2.14127
C	3.68499	0.13456	0.89925
H	2.47177	0.60599	2.60934
H	4.60106	-0.37847	-0.98031
H	4.62450	0.30328	1.41164

Single point energy calculation of intermediate **27** as an anion

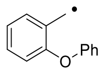

27

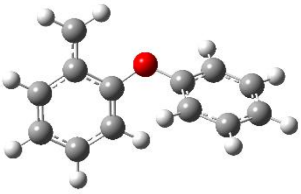

C	-3.42635	0.06515	0.21440
C	-2.25113	0.86068	0.17662
C	-1.04594	0.20425	-0.20093
C	-1.02302	-1.14300	-0.52024
C	-2.19994	-1.88925	-0.46505
C	-3.40256	-1.28013	-0.09534
H	-4.35630	0.54433	0.50007

H	-0.09063	-1.60965	-0.81383
H	-2.17508	-2.94204	-0.71756
H	-4.31502	-1.86220	-0.05390
O	0.06581	1.00290	-0.28444
C	-2.27236	2.22786	0.49955
H	-3.20297	2.70107	0.78208
H	-1.37135	2.82209	0.47467
C	1.31527	0.44966	-0.09279
C	1.60187	-0.30106	1.04359
C	2.29568	0.72970	-1.03450
C	2.89276	-0.78153	1.22769
H	0.81969	-0.50118	1.76659
C	3.58681	0.24852	-0.83523
H	2.03711	1.32093	-1.90429
C	3.88829	-0.50966	0.29139
H	3.12286	-1.36505	2.11112
H	4.35599	0.46626	-1.56651
H	4.89290	-0.88495	0.44227

Single point energy calculation of intermediate **43** as a neutral species

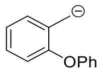

43

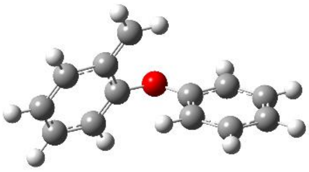

C	-2.91087	1.03358	0.26046
C	-1.61015	0.98614	-0.36605
C	-1.11440	-0.35894	-0.49596
C	-1.80618	-1.48140	-0.10375
C	-3.07475	-1.38600	0.48541
C	-3.59916	-0.09433	0.65794
H	-3.35908	2.01219	0.40802
H	-1.33458	-2.44672	-0.26480
H	-3.61752	-2.26922	0.79569

H	-4.57752	0.02760	1.11570
O	0.12601	-0.54885	-1.12732
C	-0.91126	2.10560	-0.78364
H	-1.34275	3.09188	-0.66075
H	0.06101	2.02680	-1.25102
C	1.25634	-0.29738	-0.41743
C	1.26069	0.08877	0.92447
C	2.46502	-0.46053	-1.09888
C	2.47565	0.30002	1.56904
H	0.31910	0.22953	1.43971
C	3.66939	-0.24624	-0.44142
H	2.43405	-0.75521	-2.14127
C	3.68499	0.13456	0.89925
H	2.47177	0.60599	2.60934
H	4.60106	-0.37847	-0.98031
H	4.62450	0.30328	1.41164

Intermediate **46**

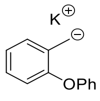

46

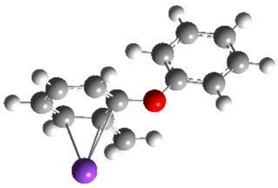

C	2.13504	1.16378	1.06792
C	1.00326	0.26920	0.94682
C	0.57760	0.09020	-0.42357
C	1.28288	0.56278	-1.50671
C	2.46139	1.31201	-1.34232
C	2.83440	1.63567	-0.02596
H	2.46981	1.41734	2.06906
H	0.90478	0.32724	-2.49674
H	2.99950	1.69928	-2.19650
H	3.69732	2.27324	0.14195
O	-0.50018	-0.75858	-0.67321

C	0.45571	-0.42449	2.00739
H	0.80024	-0.22603	3.01525
H	-0.42856	-1.03484	1.88497
C	-1.76019	-0.32497	-0.35690
C	-2.04269	0.96958	0.07192
C	-2.78486	-1.25889	-0.50492
C	-3.36172	1.31946	0.34829
H	-1.23988	1.68600	0.19278
C	-4.09561	-0.89421	-0.22720
H	-2.53472	-2.25903	-0.83831
C	-4.39318	0.39781	0.20177
H	-3.57914	2.32619	0.68606
H	-4.88806	-1.62400	-0.34631
H	-5.41549	0.68052	0.42044
K	3.01808	-1.47920	0.06915

Intermediate **47**

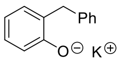

47

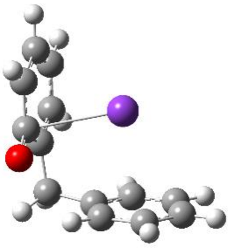

C	1.60301	-1.16226	-0.22881
C	1.13807	-0.52793	0.98553
C	1.67545	0.68010	1.41302
C	2.68622	1.33934	0.70480
C	3.18250	0.72972	-0.45316
C	2.67360	-0.48020	-0.90186
C	-0.07920	-1.12100	1.66285
H	1.28353	1.13142	2.32207
H	3.09706	2.27415	1.06456
H	3.98252	1.20812	-1.01038
H	3.06221	-0.94149	-1.80430

H	-0.11227	-0.82289	2.71349
H	-0.04282	-2.20951	1.59966
C	-1.30635	-0.60286	0.94158
C	-1.82852	-1.28031	-0.16601
C	-1.85065	0.64192	1.28103
C	-2.84930	-0.71200	-0.92774
H	-1.39405	-2.23348	-0.44206
C	-2.87133	1.20992	0.52247
H	-1.46035	1.17384	2.14336
C	-3.36786	0.53772	-0.59414
H	-3.24033	-1.24832	-1.78494
H	-3.28262	2.17244	0.80474
H	-4.16229	0.97620	-1.18645
O	1.04967	-2.20269	-0.69786
K	0.05464	1.08569	-1.27759

Intermediate **47**

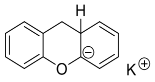

47

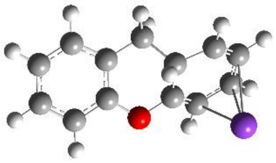

C	4.26569	0.35328	-0.27122
C	3.16919	1.20694	-0.27044
C	1.87146	0.74641	-0.03870
C	1.69817	-0.63090	0.17416
C	2.79025	-1.50049	0.16399
C	4.06909	-1.00950	-0.05186
C	0.67189	1.66123	-0.08110
C	-0.62978	-0.34789	0.43830
C	-0.56217	0.82512	-0.50217
C	-1.89237	1.54910	-0.47770
H	-2.02867	2.36068	-1.18568
C	-2.82565	1.34178	0.51417

C	-2.74384	0.29654	1.45969
C	-1.57857	-0.53131	1.39034
H	0.46843	2.09219	0.90421
H	5.26091	0.74363	-0.44517
H	3.31121	2.26756	-0.45352
H	2.61075	-2.55589	0.33214
H	4.91183	-1.69085	-0.05101
H	-3.70174	1.98767	0.52782
H	-3.46418	0.18285	2.25677
H	-1.43744	-1.35112	2.09014
O	0.47859	-1.20309	0.39898
H	0.85162	2.48797	-0.77690
H	-0.32217	0.46358	-1.52032
K	-3.16365	-1.11186	-1.02921

1-methyl-2phenoxybenzene **1** and pentavalent silicate **25b** complex

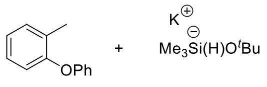

1 **25b**

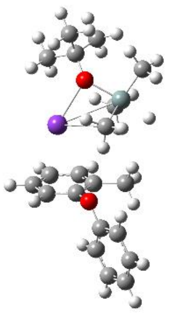

C	1.86652	2.06215	-1.03492
C	1.97372	0.91036	-0.26367
C	1.50956	0.85156	1.05675
C	0.92722	2.01057	1.57819
C	0.81507	3.17672	0.82210
C	1.28504	3.20462	-0.48872
H	2.25098	2.04870	-2.04809
H	0.55920	1.99426	2.59838
H	0.36810	4.06118	1.25993
H	1.21002	4.10889	-1.08056

C	1.64274	-0.40949	1.86457
H	2.67598	-0.76387	1.86872
H	1.33143	-0.23556	2.89452
H	1.01802	-1.20893	1.45305
O	2.51544	-0.21859	-0.84083
C	3.82467	-0.53719	-0.54936
C	4.24568	-1.80079	-0.95530
C	4.69653	0.33482	0.09410
C	5.55481	-2.19224	-0.71355
H	3.53767	-2.45497	-1.44915
C	6.00701	-0.07610	0.33239
H	4.36452	1.31692	0.40759
C	6.44281	-1.33322	-0.06746
H	5.88187	-3.17610	-1.02817
H	6.68771	0.60059	0.83523
H	7.46264	-1.64361	0.12219
Si	-2.14425	-1.58255	0.57016
C	-0.85924	-1.62877	-0.85884
H	0.09782	-1.16201	-0.59415
H	-0.62253	-2.68438	-1.02092
H	-1.21101	-1.22404	-1.81224
C	-3.41618	-2.99798	0.57894
H	-3.82006	-3.13120	1.58812
H	-4.24635	-2.88227	-0.11850
H	-2.87721	-3.91854	0.33525
C	-2.03746	-0.19888	1.91500
H	-2.49642	0.76997	1.68636
H	-0.99446	-0.04601	2.20681
H	-2.54011	-0.58631	2.80864
H	-1.16790	-2.44390	1.48021
O	-3.20493	-0.42088	-0.55472
C	-4.52864	0.04765	-0.45763

C	-5.35752	-0.56531	-1.59475
H	-5.39044	-1.65193	-1.49405
H	-6.38384	-0.18573	-1.59621
H	-4.89361	-0.32719	-2.55548
C	-4.51206	1.57591	-0.63826
H	-4.05199	1.83521	-1.59823
H	-5.52076	1.99739	-0.62913
H	-3.95017	2.04982	0.17507
C	-5.21023	-0.26168	0.88663
H	-4.65577	0.17548	1.71934
H	-6.21991	0.15878	0.89451
H	-5.29229	-1.33583	1.05236
K	-1.30506	1.27265	-0.74823

Intermediate **45**, Me_3SiO^tBu and H_2 complex

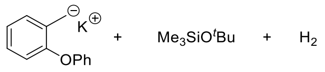

45 48

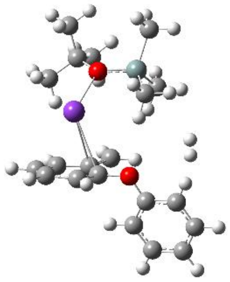

C	1.94030	2.25684	-1.28325
C	1.79871	1.17359	-0.44586
C	1.02775	1.20220	0.77896
C	0.54930	2.53129	1.10543
C	0.69972	3.61124	0.25824
C	1.36027	3.50076	-0.97875
H	2.51097	2.11649	-2.19627
H	0.02835	2.65927	2.04936
H	0.29435	4.57197	0.56205
H	1.49476	4.35359	-1.62971
C	0.73365	0.07653	1.51597
H	1.14771	-0.88835	1.25757

H	0.16610	0.16271	2.43556
H	1.12408	-3.31546	0.36800
O	2.31898	-0.04975	-0.87635
C	3.45031	-0.52863	-0.27376
C	3.96651	-1.71410	-0.79737
C	4.08523	0.10491	0.79241
C	5.11585	-2.26907	-0.25018
H	3.45531	-2.18027	-1.63169
C	5.23693	-0.46398	1.33003
H	3.67842	1.02341	1.19552
C	5.75864	-1.64784	0.81881
H	5.51262	-3.18886	-0.66419
H	5.72654	0.02794	2.16261
H	6.65476	-2.08090	1.24580
Si	-2.31849	-1.81580	-0.42259
C	-0.90481	-1.64748	-1.63738
H	-0.03994	-1.15522	-1.17714
H	-0.57036	-2.64166	-1.94756
H	-1.19826	-1.11459	-2.54738
C	-3.75041	-2.74628	-1.19655
H	-4.56496	-2.90521	-0.48462
H	-4.15243	-2.21361	-2.06221
H	-3.41225	-3.73103	-1.53221
C	-1.72398	-2.73163	1.09544
H	-0.97212	-2.14400	1.62783
H	-1.25679	-3.66657	0.76967
H	-2.52917	-2.98971	1.78481
H	1.82899	-3.10771	0.26280
O	-2.76827	-0.21619	-0.13150
C	-3.79570	0.32886	0.71506
C	-5.09773	0.39511	-0.07943
H	-5.43709	-0.60708	-0.34943

H	-5.88068	0.87747	0.51065
H	-4.94892	0.96997	-0.99645
C	-3.32567	1.73026	1.09736
H	-3.29780	2.38365	0.21979
H	-4.00986	2.18521	1.81621
H	-2.32912	1.68107	1.54645
C	-3.97419	-0.51451	1.97467
H	-3.02675	-0.60711	2.51075
H	-4.70518	-0.04238	2.63422
H	-4.34237	-1.51447	1.73065
K	-1.04209	1.51273	-1.19639

Potassium cation

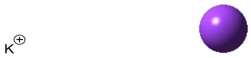

K	0.00000	0.00000	0.00000

H_2

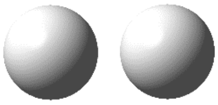

H	0.00000	0.00000	0.37036
H	0.00000	0.00000	-0.37036

Me_3SiO*t*Bu **48**

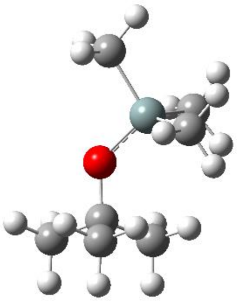

Si	1.24154	-0.00330	0.06132
O	-0.29898	-0.01614	0.70178
C	-1.58864	0.00188	0.08620

C	-1.60892	0.95505	-1.10867
C	-1.95087	-1.41664	-0.35256
C	-2.55706	0.48646	1.16048
H	-1.30376	1.95800	-0.80081
H	-0.93562	0.61110	-1.89907
H	-2.61602	1.01088	-1.52797
H	-1.89411	-2.09443	0.50195
H	-2.96634	-1.44507	-0.75514
H	-1.26526	-1.77157	-1.12488
H	-3.58048	0.50492	0.77874
H	-2.51556	-0.17933	2.02489
H	-2.28300	1.49283	1.48399
C	1.68567	1.68493	-0.62695
H	2.76081	1.73060	-0.82471
H	1.44464	2.47173	0.09287
C	1.46136	-1.29657	-1.28124
H	2.50964	-1.31768	-1.59448
H	0.85772	-1.08639	-2.16792
C	2.34420	-0.39676	1.51613
H	3.39640	-0.39722	1.21955
H	2.10624	-1.37967	1.92963
H	1.16458	1.90659	-1.56064
H	2.21572	0.34397	2.30913
H	1.20030	-2.29390	-0.91814

Intermediate **30** and pentavalent silicate **25b** complex

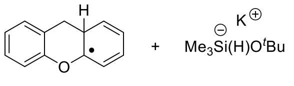

30 25b

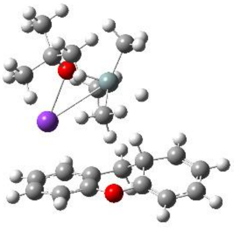

C	1.07716	3.70832	0.84272
C	1.63432	2.60655	1.48643

C	2.14841	1.52345	0.77048
C	2.06304	1.56281	-0.62691
C	1.48542	2.64903	-1.28741
C	1.00696	3.72598	-0.55161
C	2.80148	0.33987	1.43440
C	3.08773	-0.56036	-0.85235
C	2.63955	-0.89900	0.53124
C	3.33686	-2.11764	1.05319
H	3.10260	-2.41146	2.07074
C	4.18512	-2.85872	0.29427
C	4.48851	-2.49945	-1.04549
C	3.93245	-1.32343	-1.59500
H	3.86700	0.53898	1.58959
H	0.70804	4.54644	1.42095
H	1.68970	2.58225	2.56996
H	1.44809	2.63759	-2.37001
H	0.58281	4.57952	-1.06715
H	4.64253	-3.74599	0.71722
H	5.16044	-3.10588	-1.63698
H	4.17880	-1.00475	-2.60106
O	2.57460	0.58085	-1.42278
H	2.35402	0.15562	2.41436
H	1.55650	-1.12216	0.49325
Si	-1.43595	-1.54017	0.43621
C	-0.84703	-0.59631	2.00863
H	-0.62828	-1.35403	2.76640
H	0.09997	-0.06551	1.84821
H	-1.57996	0.09610	2.43233
C	-0.81551	-0.95498	-1.29994
H	-0.81457	-1.83370	-1.95425
H	-1.39942	-0.17931	-1.80803
H	0.23181	-0.63356	-1.24935

C	-2.53279	-3.06298	0.74779
H	-3.57967	-2.84530	0.96128
H	-2.10434	-3.60186	1.59817
H	-2.48152	-3.74207	-0.10967
H	-0.13943	-2.45768	0.50840
O	-2.89640	-0.29261	0.35501
C	-4.08033	-0.26384	-0.40775
C	-4.18864	1.11948	-1.07267
H	-3.36263	1.27157	-1.77712
H	-5.12098	1.22862	-1.63290
H	-4.16552	1.90481	-0.30910
C	-5.27953	-0.44482	0.53259
H	-5.25792	0.32638	1.30703
H	-6.22892	-0.37460	-0.00693
H	-5.23117	-1.41944	1.02226
C	-4.14243	-1.32922	-1.51591
H	-4.13387	-2.33655	-1.10000
H	-5.06504	-1.21029	-2.09105
H	-3.30046	-1.23251	-2.20414
K	-1.28187	1.69036	0.20803

Intermediate **31**, $Me_3SiOtBu$ **48** and H_2 complex

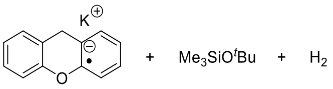

31 48

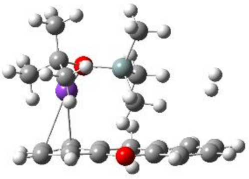

C	-0.34856	3.99112	0.09390
C	-1.35871	3.33875	-0.65346
C	-1.82830	2.04519	-0.20396
C	-1.19989	1.45548	0.86706
C	-0.08344	2.02598	1.52819
C	0.28500	3.36226	1.14927
C	-2.97829	1.38993	-0.91169

C	-2.60013	-0.45985	0.76517
C	-3.29475	0.03251	-0.33977
C	-4.28701	-0.77643	-0.89767
H	-4.83019	-0.40364	-1.76079
C	-4.59103	-2.02759	-0.37519
C	-3.89332	-2.49164	0.73909
C	-2.89754	-1.71015	1.30772
H	-3.86958	2.03122	-0.86032
H	-0.06125	5.00439	-0.17066
H	-1.91115	3.86157	-1.42507
H	0.30050	1.54974	2.42028
H	1.05571	3.88292	1.70699
H	-5.36754	-2.63281	-0.82685
H	-4.12334	-3.46148	1.16458
H	-2.33857	-2.04297	2.17416
O	-1.58947	0.22520	1.37411
H	-2.75214	1.29340	-1.98585
H	-1.78096	-4.17627	-0.37379
Si	1.34382	-1.80841	-0.35305
C	-0.07690	-1.28913	-1.45649
H	-0.79982	-2.10493	-1.54609
H	-0.62893	-0.44266	-1.03416
H	0.26164	-1.03559	-2.46578
C	0.69893	-2.19708	1.35712
H	1.44511	-2.66965	1.99807
H	0.32173	-1.29440	1.84411
H	-0.14131	-2.89069	1.25105
C	2.21323	-3.30926	-1.06194
H	2.64613	-3.09708	-2.04273
H	1.49345	-4.12498	-1.17812
H	3.01022	-3.66676	-0.40436
H	-1.33810	-4.72269	-0.61199

O	2.35713	-0.45987	-0.40131
C	3.63423	-0.22750	0.21834
C	3.78142	1.28846	0.33112
H	2.95384	1.70629	0.91378
H	4.71790	1.54724	0.82904
H	3.80239	1.74633	-0.66278
C	4.72732	-0.80380	-0.67843
H	4.65886	-0.37079	-1.67895
H	5.71455	-0.57882	-0.26770
H	4.62854	-1.88794	-0.76177
C	3.68636	-0.85497	1.60885
H	3.62934	-1.94522	1.55107
H	4.62766	-0.59549	2.09792
H	2.85883	-0.48927	2.22208
K	1.01736	1.71119	-1.22518

1-methyl-2-phenoxybenzene **1** and KO*t*Bu complex

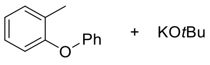

1

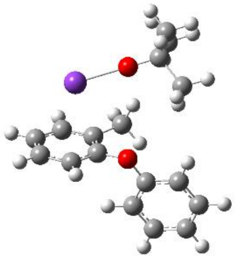

C	0.75760	2.35122	-1.19430
C	0.71329	1.24690	-0.35166
C	0.26745	1.34180	0.97415
C	-0.11447	2.60732	1.42613
C	-0.07824	3.72635	0.59452
C	0.35265	3.59835	-0.72251
H	1.10678	2.21505	-2.21106
H	-0.46117	2.70906	2.44900
H	-0.38412	4.69286	0.97679
H	0.38639	4.46096	-1.37685

C	0.12283	0.11769	1.83110
H	1.02987	-0.48985	1.83346
H	-0.11474	0.39758	2.85773
H	-0.70049	-0.48046	1.42459
O	1.04633	0.01942	-0.88168
C	2.22396	-0.57321	-0.48881
C	2.34527	-1.93161	-0.77017
C	3.25725	0.12485	0.12791
C	3.51546	-2.59523	-0.42813
H	1.51572	-2.44199	-1.24429
C	4.42321	-0.55790	0.46890
H	3.15554	1.18183	0.34202
C	4.56003	-1.91353	0.19459
H	1.02987	-0.48985	1.83346
H	-0.11474	0.39758	2.85773
H	-0.70049	-0.48046	1.42459
O	1.04633	0.01942	-0.88168
C	2.22396	-0.57321	-0.48881
C	2.34527	-1.93161	-0.77017
C	3.25725	0.12485	0.12791
C	3.51546	-2.59523	-0.42813
H	1.51572	-2.44199	-1.24429
C	4.42321	-0.55790	0.46890
H	3.15554	1.18183	0.34202
C	4.56003	-1.91353	0.19459
H	3.60940	-3.65275	-0.64465
H	5.22890	-0.01715	0.95130
H	5.46973	-2.43579	0.46333
C	-2.81304	-1.92193	0.07887
C	-4.00901	-2.39133	-0.77559
H	-4.91631	-1.87465	-0.44796

H	-3.82951	-2.13471	-1.82415
H	-4.17896	-3.47145	-0.70570
C	-1.55597	-2.68207	-0.39705
H	-1.35079	-2.42401	-1.44085
H	-0.68994	-2.37839	0.19932
H	-1.66869	-3.76925	-0.31990
C	-3.09084	-2.31183	1.54621
H	5.22890	-0.01715	0.95130
H	5.46973	-2.43579	0.46333
C	-2.81304	-1.92193	0.07887
C	-4.00901	-2.39133	-0.77559
H	-4.91631	-1.87465	-0.44796
H	-3.82951	-2.13471	-1.82415
H	-4.17896	-3.47145	-0.70570
C	-1.55597	-2.68207	-0.39705
H	-1.35079	-2.42401	-1.44085
H	-0.68994	-2.37839	0.19932
H	-1.66869	-3.76925	-0.31990
C	-3.09084	-2.31183	1.54621
H	-2.24862	-2.01084	2.17664
H	-3.98308	-1.78562	1.89837
H	-3.24823	-3.38870	1.67158
O	-2.63460	-0.56329	-0.04081
K	-2.43611	1.68601	-0.64914

Intermediate **45** and HO*t*Bu complex

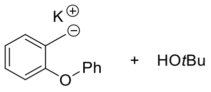

45

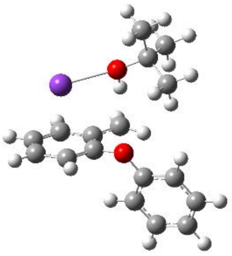

C	0.26286	2.31511	-1.14580
C	0.40948	1.23021	-0.30738
C	-0.05281	1.20419	1.05678
C	-0.69825	2.43018	1.45468
C	-0.84347	3.51557	0.60925
C	-0.38536	3.48461	-0.71768
H	0.65894	2.23171	-2.15315
H	-1.06388	2.49575	2.47465
H	-1.32549	4.41181	0.98803
H	-0.47603	4.34266	-1.37005
C	0.04639	0.08499	1.87652
H	0.6770	-0.74982	1.59925
H	-0.24566	0.16608	2.91705
H	-1.51238	-0.64845	0.91958
O	0.97428	0.07292	-0.83897
C	2.24523	-0.26960	-0.46504
C	2.70441	-1.50564	-0.91971
C	3.06698	0.54785	0.30749
C	3.99027	-1.92223	-0.60102
H	2.04189	-2.11868	-1.51939
C	4.35298	0.11529	0.61934
H	2.69893	1.50161	0.66440
C	4.82335	-1.11476	0.17099
H	4.34290	-2.88277	-0.95850
H	4.98963	0.75050	1.22435
H	5.82524	-1.44164	0.42038
C	-2.28058	-2.29352	0.10994
C	-3.49536	-2.58650	-0.76088
H	-4.40964	-2.26888	-0.25379
H	-3.41651	-2.05303	-1.71202
H	-3.56823	-3.65563	-0.97206
C	-0.99103	-2.69524	-0.60596

H	-0.89983	-2.15574	-1.55171
H	-0.12211	-2.44286	0.00679
H	-0.98071	-3.76978	-0.80696
C	-2.39262	-3.01055	1.45483
H	-1.53842	-2.75569	2.08781
H	-3.30777	-2.70739	1.96867
H	-2.41003	-4.09443	1.31661
O	-2.28116	-0.87917	0.33157
K	-2.63567	1.42710	-0.73942

Transition States

Hydrogen atom abstraction from 1-methyl-2-phenoxybenzene **1** by a Me_3Si radical **24b** (**TS1**)

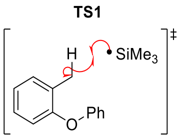

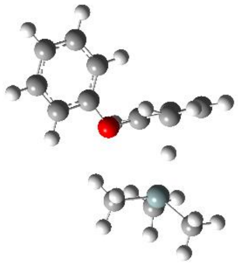

C	0.20457	2.24826	-1.31790
C	0.16225	1.21116	-0.40239
C	-0.44424	1.34464	0.85855
C	-1.03819	2.58569	1.14522
C	-1.00627	3.63296	0.23431
C	-0.38310	3.47114	-1.00201
H	0.68615	2.08101	-2.27435
H	-1.52702	2.71717	2.10472
H	-1.46999	4.57902	0.48757
H	-0.35806	4.28469	-1.71627
C	-0.55270	0.20811	1.75734
H	0.23839	-0.53502	1.72910
H	-0.93656	0.41800	2.75147
H	-1.60868	-0.58915	1.18795

O	0.66913	-0.02465	-0.77798
C	1.92547	-0.37836	-0.34848
C	2.36943	-1.64010	-0.74147
C	2.73297	0.44677	0.42940
C	3.62615	-2.07804	-0.34832
H	1.72321	-2.25488	-1.35615
C	3.99203	-0.00890	0.81603
H	2.38683	1.42765	0.72984
C	4.44533	-1.26581	0.43441
H	3.96801	-3.05884	-0.65721
H	4.61964	0.63322	1.42279
H	5.42526	-1.61013	0.74040
Si	-2.58510	-1.42627	0.09463
C	-2.73084	-0.29910	-1.40419
H	-3.05290	0.70574	-1.11878
H	-1.76690	-0.21307	-1.91067
H	-3.45969	-0.70300	-2.11441
C	-1.64721	-2.99442	-0.34571
H	-1.58263	-3.67374	0.50726
H	-2.13639	-3.52533	-1.16801
H	-0.63179	-2.74093	-0.65970
C	-4.28366	-1.82516	0.80070
H	-4.88232	-2.36282	0.05839
H	-4.82205	-0.91420	1.07139
H	-4.20619	-2.45332	1.69076

5-*exo*-trig cyclisation TS of intermediate **27** (**TS2**)

TS2

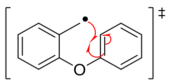

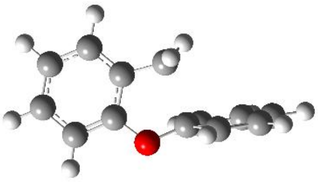

C	-1.24663	-0.65562	-0.12263
C	-1.24345	0.71042	-0.41321

C	-2.42820	1.42402	-0.25464
C	-3.58990	0.76936	0.15471
C	-3.56859	-0.59867	0.41142
C	-2.38775	-1.32777	0.28184
C	0.06574	1.22010	-0.86609
H	-2.44432	2.48713	-0.46639
H	-4.51049	1.32794	0.27094
H	-4.47306	-1.10370	0.72874
H	-2.34364	2.38767	0.49895
H	0.49638	2.08447	-0.37090
H	0.28071	1.14729	-1.92878
C	1.07007	-0.47998	-0.12047
C	2.11962	-0.67046	-1.05545
C	1.39699	-0.02620	1.18954
C	3.37523	-0.16589	-0.78145
H	1.89093	-1.16075	-1.99400
C	2.65729	0.47508	1.43986
H	0.62292	-0.02672	1.94889
C	3.64833	0.43698	0.45132
H	4.16076	-0.25897	-1.52203
H	2.88776	0.87654	2.41972
H	4.63727	0.82601	0.65846
O	-0.05220	-1.30998	-0.26651

Ring-opening of spiro intermediate **28** (**TS3**)

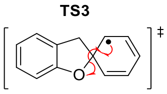

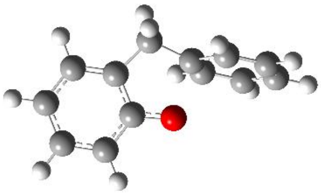

C	-1.13906	-0.56277	-0.42558
C	-1.33553	0.75776	0.00873
C	-2.58655	1.18598	0.41789

C	-3.66843	0.30332	0.38439
C	-3.47424	-1.00405	-0.05353
C	-2.21712	-1.45498	-0.44927
C	-0.06323	1.56515	-0.07710
H	-2.72211	2.20552	0.76401
H	-4.64888	0.63184	0.70547
H	-4.31087	-1.69324	-0.07628
H	-2.06081	-2.47331	-0.78263
H	0.01848	2.27455	0.75009
H	-0.03869	2.13772	-1.00939
C	1.10949	0.60395	-0.04868
C	2.15166	0.71149	-1.02301
C	1.42937	-0.04608	1.18818
C	3.35785	0.08169	-0.83472
H	1.95047	1.26357	-1.93377
C	2.64283	-0.66974	1.36646
H	0.66969	-0.07383	1.96255
C	3.60834	-0.62251	0.35381
H	4.12463	0.13745	-1.59770
H	2.85538	-1.19104	2.29162
H	4.56129	-1.11823	0.49395
O	0.08863	-0.89953	-0.80167

6-aryl cyclisation of intermediate **27** (**TS4**)

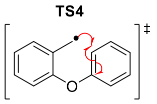

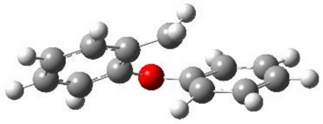

C	-3.62501	0.66434	0.03444
C	-2.47109	1.34165	-0.33815
C	-1.23322	0.69059	-0.42897
C	-1.19188	-0.66431	-0.05214
C	-2.33797	-1.35244	0.32067
C	-3.55998	-0.69061	0.35198

C	-0.00542	1.39916	-0.79005
C	1.17376	-0.72270	0.10719
C	1.21448	0.56202	0.71740
C	2.48448	1.18718	0.82127
H	2.55784	2.12686	1.35549
C	3.60407	0.62898	0.23279
C	3.51791	-0.61660	-0.39835
C	2.29664	-1.29208	-0.45651
H	0.58471	1.03857	-1.62525
H	-4.57067	1.19055	0.07660
H	-2.51788	2.39511	-0.59203
H	-2.25116	-2.39702	0.59396
H	-4.45412	-1.22938	0.64076
H	4.55704	1.14119	0.28824
H	4.39781	-1.06461	-0.84293
H	2.20215	-2.24942	-0.95443
O	-0.01707	-1.38400	-0.05369
H	-0.01224	2.47842	-0.66977
H	0.44250	0.83072	1.42944

Deprotonation of intermediate **30** by KO^{t}Bu (**TS5**)

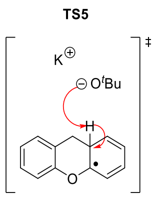

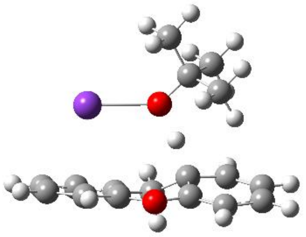

C	3.90043	-0.34189	0.70048
C	2.75141	-0.60396	1.44572
C	1.57088	-1.04591	0.84872
C	1.56009	-1.21230	-0.54544
C	2.70319	-0.94692	-1.30649
C	3.86991	-0.51938	-0.68366
C	0.31703	-1.33810	1.62730

C	-0.75322	-1.74303	-0.58981
C	-0.89862	-1.11134	0.72017
C	-2.21299	-1.38398	1.32329
H	-2.37100	-1.05794	2.34753
C	-3.23716	-1.96830	0.62543
C	-3.05779	-2.42189	-0.70083
C	-1.77364	-2.31507	-1.28892
H	0.34543	-2.37230	1.99543
H	4.80831	-0.01598	1.19299
H	2.76331	-0.47015	2.52294
H	2.65552	-1.09456	-2.37884
H	4.75526	-0.33003	-1.27921
H	-4.20281	-2.09672	1.10383
H	-3.87001	-2.88367	-1.24578
H	-1.58198	-2.68353	-2.29071
O	0.47688	-1.65653	-1.22786
H	0.26342	-0.68255	2.50149
H	-0.78122	0.17049	0.47994
C	-1.65315	2.17202	-0.09288
C	-1.24659	3.54892	-0.63513
H	-0.66113	4.08850	0.11665
H	-0.64009	3.43138	-1.53967
H	-2.11771	4.15936	-0.89123
C	-2.44842	1.41908	-1.17054
H	-1.81674	1.25402	-2.04870
H	-2.77128	0.44366	-0.79630
H	-3.33453	1.98254	-1.47888
C	-2.53084	2.35638	1.15290
H	-2.81408	1.37710	1.54782
H	-1.97158	2.89312	1.92457
H	-3.44332	2.91709	0.92798
O	-0.49035	1.46177	0.24066

K	1.88057	1.96117	-0.17874

Deprotonation of intermediate **30** by pentavalent silicate **25b**

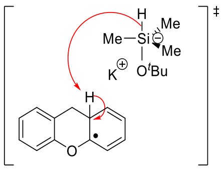

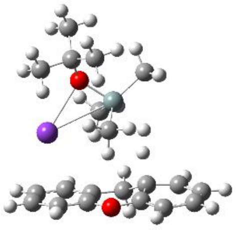

C	1.18746	3.76276	0.74331
C	1.66537	2.64161	1.42051
C	2.13585	1.51439	0.74530
C	2.09228	1.52370	-0.65610
C	1.60087	2.63705	-1.35156
C	1.16540	3.75618	-0.65336
C	2.71456	0.32297	1.46310
C	2.91402	-0.70386	-0.82479
C	2.66590	-0.92078	0.58480
C	3.33657	-2.11665	1.09449
H	3.35135	-2.26585	2.17015
C	3.82743	-3.08433	0.25560
C	3.82961	-2.90775	-1.14435
C	3.39443	-1.66880	-1.66619
H	3.75229	0.55262	1.74216
H	0.85639	4.63389	1.29534
H	1.69128	2.63746	2.50608
H	1.59931	2.60797	-2.43476
H	0.81494	4.62465	-1.19951
H	4.24509	-3.99236	0.67823
H	4.21985	-3.67163	-1.80312
H	3.45484	-1.45125	-2.72688
O	2.54316	0.50096	-1.41632
H	2.17143	0.15093	2.39882
H	1.30241	-1.36230	0.68417

Si	-1.35547	-1.15672	0.57775
C	-0.84412	-0.09160	2.07296
H	-0.55311	-0.76778	2.88009
H	0.03316	0.52865	1.87569
H	-1.66461	0.53505	2.43576
C	-0.64265	-0.82297	-1.16281
H	-0.46084	-1.78555	-1.65030
H	-1.31077	-0.24777	-1.81395
H	0.32888	-0.32676	-1.12514
C	-1.93007	-2.89711	1.02365
H	-2.99246	-2.92485	1.27968
H	-1.35747	-3.26016	1.87885
H	-1.75432	-3.58847	0.19529
H	0.40564	-1.81150	0.81061
O	-2.85940	-0.23794	0.35955
C	-4.03947	-0.48921	-0.39387
C	-4.33466	0.77802	-1.20183
H	-3.53767	0.95726	-1.93128
H	-5.27494	0.69134	-1.75147
H	-4.41257	1.63890	-0.53068
C	-5.17903	-0.74690	0.59397
H	-5.28208	0.10413	1.27124
H	-6.12800	-0.89961	0.07296
H	-4.96351	-1.63578	1.19090
C	-3.91286	-1.67737	-1.35570
H	-3.75161	-2.61142	-0.81646
H	-4.83658	-1.77907	-1.93076
H	-3.09154	-1.53532	-2.06057
K	-1.32788	1.97307	-0.03939

Ring-opening of intermediate **31**

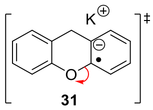

31

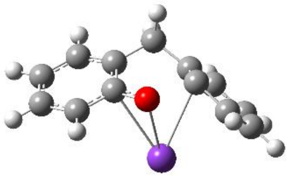

C	3.60408	0.46363	0.63851
C	2.54495	1.34274	0.43503
C	1.38762	0.96887	-0.24738
C	1.27620	-0.35753	-0.74150
C	2.34897	-1.24558	-0.51051
C	3.49648	-0.83893	0.15633
C	0.25415	1.95203	-0.44784
C	-1.30581	0.20911	-1.26102
C	-1.06024	1.22473	-0.31324
C	-1.95779	1.43141	0.72568
H	-1.77049	2.22709	1.44262
C	-3.11037	0.63544	0.85358
C	-3.35364	-0.36185	-0.09602
C	-2.46094	-0.58849	-1.14533
H	0.33613	2.76160	0.28169
H	4.49430	0.78877	1.16260
H	2.61359	2.35946	0.81110
H	2.25758	-2.25550	-0.89623
H	4.30910	-1.54280	0.29934
H	-3.81555	0.81714	1.65501
H	-4.26013	-0.95647	-0.02834
H	-2.67645	-1.35503	-1.88364
O	0.19189	-0.82190	-1.35357
H	0.31924	2.39467	-1.44850
K	-0.70128	-1.45669	1.11343

5-*exo*-trig cyclisation TS of intermediate **40**

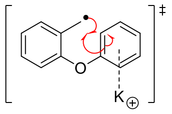

40

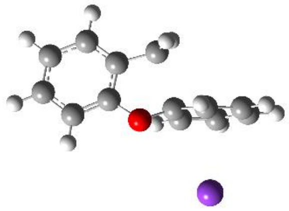

C	-1.66003	-0.52504	-0.35538
C	-2.00692	0.81295	-0.17344
C	-3.29908	1.11153	0.25050
C	-4.21966	0.08301	0.44593
C	-3.85110	-1.24144	0.22826
C	-2.55396	-1.56297	-0.16906
C	-0.91755	1.76217	-0.46959
H	-3.58781	2.14414	0.40794
H	-5.22690	0.31776	0.76646
H	-4.57199	-2.03517	0.38117
H	-2.24214	-2.58845	-0.32235
H	-0.66464	2.52550	0.25925
H	-0.78038	2.05314	-1.50738
C	0.53525	0.23709	-0.32720
C	1.52478	0.61519	-1.27442
C	0.85756	0.31830	1.06160
C	2.66310	1.27900	-0.85004
H	1.32779	0.43527	-2.32473
C	1.99868	0.98880	1.46107
H	0.15302	-0.08078	1.78343
C	2.90303	1.49063	0.51382
H	3.38057	1.62722	-1.58390
H	2.20252	1.11113	2.51855
H	3.79160	2.01863	0.83522
O	-0.35602	-0.76079	-0.73694
K	3.01168	-1.77144	0.13840

6-aryl cyclisation TS of intermediate **40**

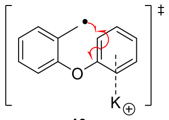

40

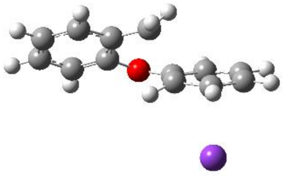

C	4.19920	0.03317	-0.60477
C	3.24508	1.04079	-0.55306
C	1.98419	0.82197	0.01746
C	1.69818	-0.47791	0.46459
C	2.63902	-1.49426	0.41791
C	3.89953	-1.23294	-0.10775
C	0.95922	1.86492	0.06287
C	-0.63675	-0.09273	0.61334
C	-0.63867	0.66020	-0.60058
C	-1.82224	1.38560	-0.90924
H	-1.87699	1.90915	-1.85625
C	-2.86409	1.47904	0.00151
C	-2.81038	0.76586	1.20545
C	-1.69313	-0.02545	1.50364
H	0.51817	2.12950	1.01785
H	5.17394	0.23464	-1.03083
H	3.47617	2.02944	-0.93366
H	2.37060	-2.47636	0.78743
H	4.63956	-2.02279	-0.14101
H	-3.72803	2.09211	-0.22554
H	-3.62157	0.83528	1.91940
H	-1.61541	-0.56108	2.44232
O	0.46127	-0.80298	0.99672
H	1.06644	2.69011	-0.63437
H	0.00844	0.35264	-1.41446
K	-3.10824	-1.53465	-0.89331

Concerted Truce-Smiles rearrangement TS of intermediate **45** (**TS6**)

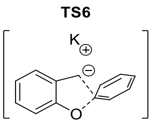

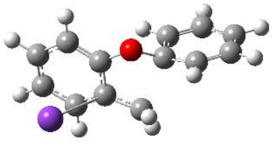

C	-0.66676	-0.17459	-0.61316
C	-0.78540	-0.07304	0.80483
C	-1.79472	-0.84200	1.41032
C	-2.72245	-1.54339	0.63060
C	-2.64783	-1.52429	-0.76269
C	-1.57504	-0.86589	-1.39270
C	0.28552	0.70263	1.35983
H	-1.87976	-0.85096	2.49219
H	-3.51303	-2.10412	1.11709
H	-3.37264	-2.06620	-1.35797
H	-1.42811	-0.92682	-2.46502
H	0.55545	0.54552	2.40127
H	0.34657	1.73681	1.03328
C	1.59810	0.24756	-0.20045
C	2.56680	1.28056	-0.32187
C	2.09931	-1.08118	-0.09206
C	3.90441	1.02687	-0.07857
H	2.22383	2.28667	-0.53915
C	3.43983	-1.31166	0.15343
H	1.39552	-1.90697	-0.13816
C	4.36724	-0.26423	0.19553
H	4.60806	1.85253	-0.12116
H	3.77786	-2.33431	0.29047
H	5.41736	-0.45488	0.37506
O	0.44329	0.41437	-1.11140
K	-3.16271	1.44441	-0.03974

6-aryl cyclisation TS of intermediate **46**

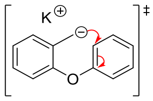

46

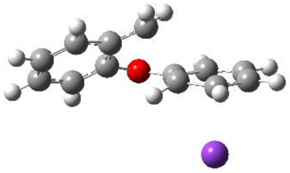

C	4.08323	-0.05069	-0.70012
C	3.19948	1.01034	-0.60785
C	1.96751	0.92003	0.08921
C	1.65028	-0.38039	0.56727
C	2.54291	-1.44277	0.50434
C	3.77617	-1.28657	-0.11714
C	1.01485	1.98294	0.14996
C	-0.68846	-0.01473	0.66394
C	-0.62338	0.69323	-0.58568
C	-1.85893	1.26585	-1.02312
H	-1.89012	1.74686	-1.99423
C	-2.97063	1.33267	-0.18867
C	-2.97057	0.70490	1.06172
C	-1.80131	0.03051	1.47394
H	0.44520	2.12720	1.05987
H	5.02805	0.08414	-1.21520
H	3.46327	1.96953	-1.04273
H	2.24288	-2.39333	0.93244
H	4.47395	-2.11325	-0.16489
H	-3.85941	1.85536	-0.52785
H	-3.83447	0.74722	1.71226
H	-1.74561	-0.45325	2.44367
O	0.42888	-0.63704	1.16645
H	1.29909	2.90741	-0.34871
H	0.08456	0.34792	-1.33092

K	-2.79291	-1.59586	-0.87274

Deprotonation of 1-methyl-2phenoxybenzene **1** by pentavalent silicate **25b** (**TS7**)

TS7

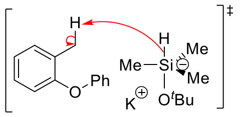

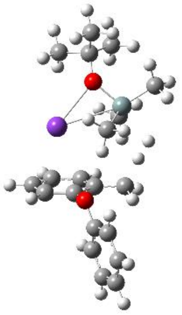

C	1.83772	2.09395	-0.96740
C	1.89707	0.90280	-0.26278
C	1.38827	0.74483	1.05668
C	0.81139	1.92786	1.60053
C	0.75056	3.12660	0.90306
C	1.25279	3.22953	-0.39888
H	2.27252	2.12103	-1.96095
H	0.43172	1.88805	2.61612
H	0.31744	3.99681	1.38484
H	1.22570	4.16691	-0.93955
C	1.41976	-0.50872	1.76098
H	2.20013	-1.19829	1.44761
H	1.37232	-0.40194	2.84442
H	0.25281	-1.42169	1.59558
O	2.47319	-0.19144	-0.89472
C	3.76463	-0.52412	-0.56774
C	4.24880	-1.70591	-1.12608
C	4.57368	0.25370	0.25592
C	5.55054	-2.10740	-0.86030
H	3.59245	-2.29150	-1.75842
C	5.87703	-0.16471	0.51462
H	4.19037	1.16695	0.69345
C	6.37360	-1.33955	-0.03802

H	5.92309	-3.02690	-1.29642
H	6.50523	0.43940	1.15883
H	7.38807	-1.65618	0.16993
Si	-2.07424	-1.30879	0.48638
C	-0.73640	-1.46461	-0.84871
H	0.21285	-0.98893	-0.60016
H	-0.51219	-2.53002	-0.94719
H	-1.08955	-1.11662	-1.82437
C	-2.90997	-2.95000	0.88726
H	-3.24403	-2.97718	1.92754
H	-3.77462	-3.13238	0.24397
H	-2.20098	-3.76799	0.74915
C	-1.99313	-0.05038	1.91399
H	-2.41343	0.92979	1.65788
H	-0.99155	0.08543	2.31334
H	-2.61313	-0.45088	2.72332
H	-0.49403	-2.04094	1.56879
O	-3.17507	-0.42806	-0.55835
C	-4.56888	-0.14795	-0.48267
C	-5.28791	-1.04156	-1.49482
H	-5.16214	-2.09338	-1.23017
H	-6.35752	-0.81823	-1.52607
H	-4.86675	-0.88533	-2.49047
C	-4.74457	1.32091	-0.87380
H	-4.31769	1.49731	-1.86535
H	-5.80019	1.60132	-0.90173
H	-4.24440	1.96735	-0.14536
C	-5.14977	-0.36941	0.91760
H	-4.67712	0.28929	1.64840
H	-6.22079	-0.15293	0.90848
H	-5.01984	-1.40360	1.24235

K	-1.22956	1.48048	-0.83837

Deprotonation of 1-methyl-2phenoxybenzene **1** by KO*t*Bu

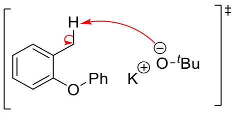

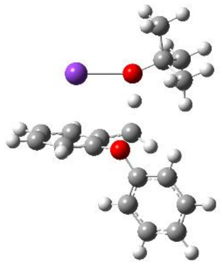

C	0.61965	2.47389	-1.02706
C	0.62658	1.28364	-0.31662
C	0.05254	1.14745	0.97178
C	-0.51318	2.33496	1.49668
C	-0.53165	3.53281	0.79195
C	0.02307	3.61568	-0.48712
H	1.07944	2.48858	-2.00919
H	-0.95904	2.28924	2.48515
H	-0.98021	4.41045	1.24459
H	0.02078	4.54727	-1.03861
C	-0.12537	-0.14083	1.60638
H	0.64301	-0.87999	1.38820
H	-0.33953	-0.07273	2.67287
H	-1.28324	-0.53242	0.92301
O	1.13915	0.16337	-0.95401
C	2.32347	-0.3652	-0.50747
C	2.65037	-1.62911	-0.99508
C	3.18172	0.30399	0.36014
C	3.84504	-2.22359	-0.61240
H	1.95715	-2.12538	-1.66357
C	4.37509	-0.30752	0.73711
H	2.91787	1.28305	0.74034
C	4.71489	-1.56719	0.25672
H	4.09584	-3.20698	-0.99256

H	5.04177	0.21207	1.41546
H	5.64459	-2.03452	0.55657
C	-2.61701	-2.05270	0.12273
C	-3.88743	-2.18819	-0.72053
H	-4.70681	-1.63754	-0.25020
H	-3.71768	-1.77455	-1.71928
H	-4.18897	-3.23443	-0.82838
C	-1.46930	-2.81821	-0.55259
H	-1.28395	-2.40416	-1.54790
H	-0.55314	-2.71290	0.03442
H	-1.70003	-3.88300	-0.65319
C	-2.86571	-2.63156	1.52261
H	-1.96557	-2.52678	2.13405
H	-3.67689	-2.08444	2.01042
H	-3.13500	-3.69093	1.47792
O	-2.29043	-0.68994	0.22014
K	-2.42720	1.53541	-0.86277

N-containing

Substrates

Substrate **50b**

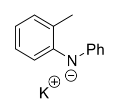

50b

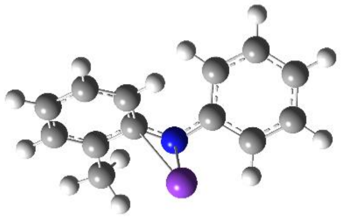

C	1.12175	-1.01626	-0.81576
C	1.04861	-0.17297	0.32471
C	2.28810	0.16470	0.95252
C	3.48084	-0.34207	0.45706
C	3.52872	-1.18068	-0.66135
C	2.33570	-1.50593	-1.29309
H	0.20765	-1.30056	-1.32627
H	4.40492	-0.07679	0.96293
H	4.47431	-1.56667	-1.02160
H	2.33948	-2.15110	-2.16579
C	2.26787	1.0682	2.15351
H	1.61725	0.66140	2.93147
H	3.27255	1.19914	2.55914
H	1.85745	2.05043	1.90132
C	-1.33510	-0.02542	0.52215
C	-1.68685	-1.39418	0.37510
C	-2.41154	0.89934	0.46499
C	-3.00100	-1.78616	0.17001
H	-0.91265	-2.14762	0.45888
C	-3.72361	0.49457	0.25223
H	-2.19012	1.94785	0.64779
C	-4.03700	-0.85287	0.09131
H	-3.22477	-2.84417	0.07807

H	-4.51102	1.24080	0.22411
H	-5.05909	-1.17089	-0.07193
N	-0.08067	0.46234	0.77070
K	-0.05106	1.83261	-1.50176

Substrate **67**

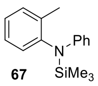

67

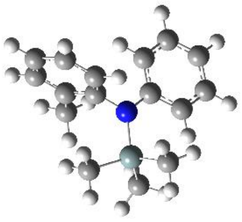

C	1.75144	-0.59196	-1.46257
C	1.13990	-0.38411	-0.22951
C	1.76715	-0.79562	0.95506
C	3.01612	-1.41003	0.86249
C	3.63245	-1.61307	-0.36947
C	2.99937	-1.20331	-1.53768
H	1.23402	-0.26766	-2.35927
H	3.51305	-1.73221	1.77160
H	4.60405	-2.09084	-0.41455
H	3.47078	-1.35883	-2.50074
C	1.09943	-0.56733	2.28429
H	1.67278	-1.02663	3.08974
H	1.00573	0.50202	2.49727
C	-1.27628	-0.54018	-0.15161
C	-1.18353	-1.94353	-0.16351
C	-2.56398	0.02624	-0.11817
C	-2.32705	-2.73326	-0.13699
H	-0.21105	-2.41817	-0.19655
C	-3.69763	-0.77512	-0.09286
H	-2.69024	1.10132	-0.11496
C	-3.59501	-2.16315	-0.10049

H	-2.21770	-3.81196	-0.14668
H	-4.67314	-0.30264	-0.06889
H	-4.48189	-2.78383	-0.08122
N	-0.13415	0.27070	-0.17017
Si	-0.14362	2.04632	-0.05427
C	-0.82676	2.79491	-1.63177
H	-1.83987	2.45212	-1.85273
H	-0.84994	3.88565	-1.55154
H	-0.18933	2.53214	-2.48017
C	1.64037	2.57308	0.15166
H	2.25186	2.27561	-0.70308
H	1.67657	3.66329	0.23456
H	2.09205	2.15062	1.05272
C	-1.15111	2.58721	1.44237
H	-0.61143	3.36363	1.99123
H	-2.12607	2.99447	1.16488
H	-1.31839	1.74770	2.12213
H	0.08865	-0.98382	2.28975

Substrate **68**

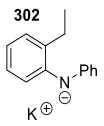

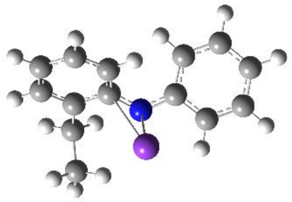

C	-0.80252	-1.52271	0.48553
C	-0.87948	-0.34083	-0.29961
C	-2.17985	0.03499	-0.76092
C	-3.28253	-0.74937	-0.44944
C	-3.18232	-1.91077	0.32137
C	-1.92843	-2.28397	0.78837
H	0.16367	-1.85262	0.85301

H	-4.25471	-0.44378	-0.82741
H	-4.06008	-2.50553	0.54217
H	-1.81545	-3.17953	1.39097
C	-2.34964	1.29901	-1.56421
H	-1.50498	1.40421	-2.24837
H	-3.26184	1.22279	-2.16289
C	1.46442	0.12147	-0.48596
C	1.94817	-1.16846	-0.83455
C	2.45385	1.07943	-0.13754
C	3.30296	-1.46336	-0.81493
H	1.24212	-1.92602	-1.15353
C	3.80805	0.76981	-0.11564
H	2.12561	2.09614	0.06413
C	4.25264	-0.50862	-0.44428
H	3.62766	-2.45799	-1.10347
H	4.52438	1.54048	0.15018
H	5.30769	-0.75176	-0.43031
N	0.15898	0.52959	-0.51223
K	0.15587	1.00764	2.09497
C	-2.43005	2.54710	-0.67689
H	-3.26922	2.47134	0.01983
H	-1.50574	2.65611	-0.10564
H	-2.56501	3.45143	-1.27537

Substrate **52**

52

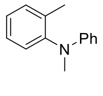

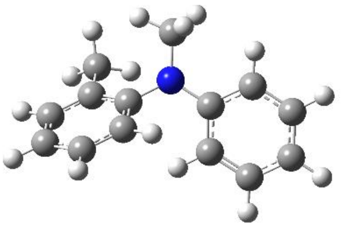

C	1.17247	-1.03849	-1.00815
C	1.07204	0.11835	-0.23250

C	2.14972	0.53644	0.56080
C	3.31855	-0.22904	0.54108
C	3.42004	-1.38074	-0.22992
C	2.33902	-1.79090	-1.00608
H	0.31940	-1.34240	-1.60533
H	4.15695	0.08155	1.15592
H	4.33602	-1.95950	-0.22038
H	2.40647	-2.68738	-1.61083
C	2.05111	1.76905	1.41925
H	1.04292	1.87874	1.82194
H	2.76303	1.71859	2.24362
H	2.27378	2.67404	0.84586
C	-1.35665	0.28240	-0.05198
C	-1.44902	-0.85412	0.77373
C	-2.53902	0.79595	-0.60639
C	-2.67533	-1.44785	1.02414
H	-0.55015	-1.26085	1.22098
C	-3.76514	0.19129	-0.33865
H	-2.51242	1.66404	-1.25088
C	-3.84851	-0.93281	0.47187
H	-2.71611	-2.32066	1.66591
H	-4.66244	0.60932	-0.78082
H	-4.80442	-1.39986	0.67287
N	-0.12288	0.89962	-0.26055
C	-0.06711	2.10793	-1.06890
H	-0.72257	2.87533	-0.65139
H	0.95350	2.48637	-1.06198
H	-0.35674	1.92047	-2.11142

Intermediates

Substrate **50b** and Me_3Si radical **24b** complex

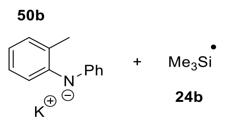

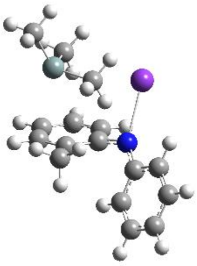

C	0.26676	2.13467	1.19909
C	-0.30069	1.17959	0.31876
C	0.12791	1.22440	-1.04347
C	1.08024	2.16872	-1.42509
C	1.63044	3.09117	-0.53624
C	1.20896	3.07020	0.78886
H	-0.09970	2.14962	2.22248
H	1.41424	2.16658	-2.45931
H	2.36959	3.80691	-0.87492
H	1.60385	3.78582	1.50217
C	-0.38758	0.25632	-2.07727
H	-1.31184	0.61794	-2.53727
H	0.35650	0.11740	-2.86512
H	-0.62179	-0.71462	-1.63807
C	-2.30326	-0.12854	0.30805
C	-2.91145	-1.36563	0.64346
C	-3.05242	0.72619	-0.54044
C	-4.15715	-1.72471	0.15445
H	-2.36814	-2.03851	1.30075
C	-4.29946	0.35429	-1.02494
H	-2.64149	1.69696	-0.79843
C	-4.87078	-0.87327	-0.69227
H	-4.58014	-2.68419	0.43500
H	-4.83917	1.04097	-1.66967

H	-5.84377	-1.15605	-1.07426
Si	2.75026	-1.18122	-0.87844
C	3.33876	-0.03365	0.50099
H	4.23878	0.50233	0.18993
H	2.58672	0.72520	0.73738
H	3.59921	-0.59970	1.40476
C	1.31970	-2.24870	-0.25701
H	0.99443	-2.93823	-1.04008
H	1.63973	-2.86206	0.59568
H	0.44526	-1.65026	0.02175
C	4.17134	-2.30719	-1.40143
H	4.51293	-2.90415	-0.54834
H	5.02072	-1.72802	-1.76972
H	3.86104	-2.99454	-2.19128
N	-1.09953	0.19384	0.86312
K	0.72043	-0.64749	2.51683

Intermediate **58b** and Me_3SiH complex

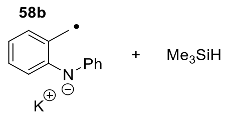

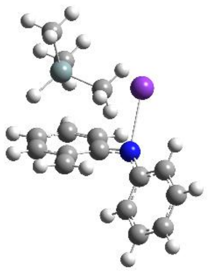

C	0.29234	2.27925	1.12649
C	-0.34188	1.31729	0.29585
C	0.06326	1.29760	-1.10916
C	1.02165	2.26667	-1.54030
C	1.59993	3.18380	-0.69233
C	1.23151	3.18741	0.66392
H	-0.03925	2.32836	2.16080
H	1.31012	2.24641	-2.58716

H	2.32760	3.89398	-1.06635
H	1.66052	3.91375	1.34509
C	-0.37496	0.33771	-2.02288
H	-1.07503	-0.44199	-1.76688
H	0.00704	0.35856	-3.03639
H	2.44152	-0.76866	-2.19261
C	-2.30585	-0.07278	0.33749
C	-2.78548	-1.34723	0.71139
C	-3.12081	0.68080	-0.54068
C	-3.98663	-1.84563	0.22345
H	-2.18552	-1.94243	1.39359
C	-4.31833	0.17487	-1.02260
H	-2.79064	1.67089	-0.83627
C	-4.76767	-1.09441	-0.65289
H	-4.31629	-2.83268	0.53082
H	-4.91633	0.78277	-1.69374
H	-5.70436	-1.48282	-1.03309
Si	2.68820	-1.37992	-0.86446
C	3.25189	-0.04770	0.34195
H	4.15610	0.43998	-0.03148
H	2.49946	0.73753	0.46711
H	3.50844	-0.48015	1.31584
C	1.12572	-2.21787	-0.22675
H	0.75187	-2.92944	-0.96783
H	1.33697	-2.79311	0.68254
H	0.31594	-1.50478	-0.03877
C	4.04637	-2.67126	-1.02358
H	4.25925	-3.13133	-0.05454
H	4.97258	-2.22567	-1.39440
H	3.75532	-3.46573	-1.71500
N	-1.14826	0.40405	0.90187

K	0.75480	-0.44495	2.47852

Intermediate **58b**

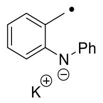

58b

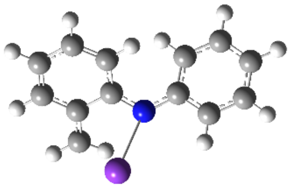

C	0.77892	-1.53191	-0.67777
C	0.82746	-0.38512	0.16932
C	2.13046	-0.03400	0.74382
C	3.27351	-0.80132	0.34338
C	3.17369	-1.88093	-0.49431
C	1.90705	-2.25157	-1.00452
H	-0.17270	-1.80945	-1.11465
H	4.23757	-0.51857	0.75553
H	4.05545	-2.45140	-0.76148
H	1.82455	-3.09846	-1.67666
C	2.25588	1.00020	1.66675
H	1.37820	1.47941	2.08098
H	3.22351	1.23097	2.09652
C	-1.51256	0.08115	0.22351
C	-2.01591	-1.19384	0.56541
C	-2.45180	1.03867	-0.21384
C	-3.36738	-1.48828	0.45045
H	-1.33165	-1.94272	0.94740
C	-3.80141	0.73566	-0.33275
H	-2.09827	2.03696	-0.45256
C	-4.27520	-0.53373	-0.00733
H	-3.71908	-2.47539	0.73110
H	-4.49064	1.49873	-0.67825
H	-5.32822	-0.77068	-0.09636

N	-0.19611	0.46526	0.38892
K	1.17146	2.47546	-0.69193

Intermediate **59b**

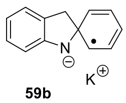

59b

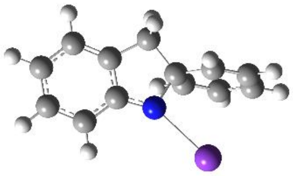

C	1.43869	0.47795	-0.18269
C	1.86194	-0.87942	-0.13089
C	3.17953	-1.22633	0.07482
C	4.14630	-0.22070	0.24134
C	3.74896	1.11483	0.17908
C	2.42017	1.48011	-0.02823
C	0.65277	-1.72570	-0.44305
H	3.47241	-2.27210	0.10137
H	5.18385	-0.47879	0.41319
H	4.49359	1.89523	0.30250
H	2.13649	2.52674	-0.06673
H	0.53589	-2.61313	0.18241
H	0.67414	-2.05236	-1.49049
C	-0.50902	-0.70487	-0.23347
C	-1.62059	-0.89246	-1.22378
C	-1.02074	-0.75719	1.18126
C	-2.93715	-0.95236	-0.87274
H	-1.32649	-0.93570	-2.26808
C	-2.34911	-0.79229	1.50307
H	-0.26164	-0.68476	1.95504
C	-3.33699	-0.88767	0.49075
H	-3.69637	-1.05967	-1.64045
H	-2.65741	-0.75598	2.54281
H	-4.38611	-0.94042	0.75336

N	0.10738	0.65448	-0.38196
K	-2.05098	2.03085	-0.07601

Intermediate **61b**

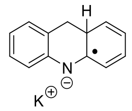

61b

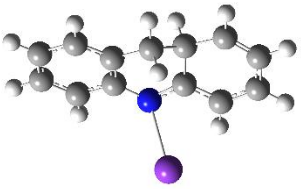

C	-3.86886	-0.73631	0.18644
C	-2.72076	-1.38251	0.64601
C	-1.45105	-0.91441	0.33380
C	-1.29994	0.23610	-0.48234
C	-2.46850	0.88311	-0.93248
C	-3.72915	0.40444	-0.60377
C	-0.19033	-1.55849	0.84314
C	1.03715	0.03263	-0.62357
C	0.91602	-1.42774	-0.21514
C	2.22312	-2.02745	0.22383
H	2.18435	-3.01569	0.67281
C	3.41771	-1.40963	0.02027
C	3.49160	-0.11564	-0.56729
C	2.30878	0.57965	-0.85283
H	0.14864	-1.05823	1.76159
H	-4.85103	-1.11746	0.43803
H	-2.81249	-2.27074	1.26496
H	-2.35488	1.76102	-1.55977
H	-4.60977	0.91897	-0.97286
H	4.33528	-1.90838	0.31634
H	4.45446	0.33890	-0.76436
H	2.35590	1.57257	-1.29634
H	-0.36055	-2.60887	1.09556

H	0.54216	-1.97277	-1.10353
N	-0.06817	0.78290	-0.79419
K	0.81317	2.55252	0.93950

Intermediate **61b** and $Me_3Si(H)O\textit{t}BuK$ **25b** complex

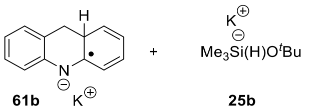

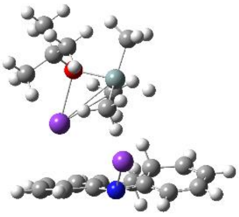

C	-0.68789	3.99777	-0.46455
C	-1.32579	3.07607	-1.30316
C	-1.89407	1.90611	-0.81442
C	-1.84274	1.61537	0.58383
C	-1.15624	2.54150	1.41165
C	-0.61186	3.71265	0.90224
C	-2.64826	0.94512	-1.69631
C	-2.98968	-0.41279	0.35243
C	-2.56574	-0.47143	-1.10431
C	-3.30913	-1.50012	-1.90457
H	-3.11801	-1.52305	-2.97324
C	-4.14494	-2.40542	-1.33441
C	-4.41440	-2.38742	0.06368
C	-3.84907	-1.38551	0.86436
H	-3.70251	1.24330	-1.75568
H	-0.27713	4.91615	-0.86524
H	-1.39659	3.27937	-2.36837
H	-1.11717	2.33732	2.47688
H	-0.12885	4.41280	1.57581
H	-4.63231	-3.15003	-1.95518
H	-5.10159	-3.10499	0.49361
H	-4.16545	-1.28899	1.90153
H	-2.24950	0.95667	-2.71532

H	-1.49294	-0.74152	-1.11671
Si	1.62948	-1.51066	-0.90489
C	1.17261	-0.41567	-2.41611
H	1.05753	-1.09380	-3.26625
H	0.19394	0.06971	-2.30177
H	1.92157	0.33363	-2.68782
C	0.78835	-1.15868	0.81246
H	0.73747	-2.12751	1.32472
H	1.27191	-0.43715	1.48161
H	-0.23697	-0.83364	0.60191
C	2.80739	-2.96488	-1.25040
H	3.86413	-2.70294	-1.31583
H	2.49617	-3.41281	-2.19892
H	2.68392	-3.73903	-0.48567
H	0.37838	-2.44179	-1.20075
O	3.00475	-0.25275	-0.50280
C	4.08747	-0.27857	0.39780
C	4.05641	1.02019	1.22158
H	3.13872	1.07352	1.81882
H	4.90287	1.08322	1.91054
H	4.10442	1.88833	0.55561
C	5.39570	-0.31493	-0.40350
H	5.43065	0.53805	-1.08595
H	6.27111	-0.27698	0.25186
H	5.44798	-1.22856	-0.99897
C	4.06747	-1.46473	1.37826
H	4.13930	-2.41669	0.85238
H	4.91886	-1.39207	2.06086
H	3.15477	-1.46814	1.97774
K	1.34047	1.72205	-0.40041
N	-2.47373	0.54942	1.15899
K	-1.53219	-1.26705	2.86391

Intermediate **62b**, $Me_3SiOtBu$ **49** and H_2 complex

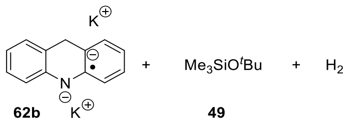

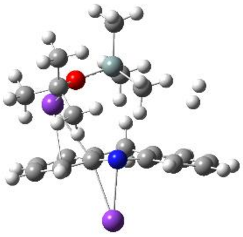

C	-0.96474	3.69796	-1.01087
C	-1.58254	2.60845	-1.67353
C	-1.67809	1.33216	-1.00387
C	-1.05774	1.12717	0.23624
C	-0.41476	2.24451	0.88138
C	-0.39378	3.52554	0.23917
C	-2.35453	0.19076	-1.71501
C	-1.96782	-1.00998	0.48716
C	-2.67406	-0.92857	-0.75173
C	-3.62712	-1.89402	-1.07323
H	-4.15595	-1.79869	-2.01886
C	-3.91002	-2.97056	-0.23519
C	-3.20033	-3.08214	0.96362
C	-2.25672	-2.12941	1.31392
H	-3.27726	0.54446	-2.19646
H	-0.93946	4.67465	-1.48525
H	-2.09798	2.75124	-2.61736
H	0.02608	2.09164	1.85924
H	0.07033	4.36931	0.74108
H	-4.65326	-3.70790	-0.51318
H	-3.38871	-3.91733	1.63050
H	-1.71545	-2.20808	2.25187
H	-1.73065	-0.18019	-2.55334
H	-1.31031	-3.64329	-1.04000
Si	2.23403	-1.68087	-0.58460

C	0.93925	-1.37304	-1.89754
H	0.57605	-2.32281	-2.29663
H	0.07880	-0.86160	-1.45339
H	1.32140	-0.79754	-2.74725
C	1.49483	-2.68718	0.80257
H	2.22922	-2.98230	1.55583
H	0.69143	-2.11507	1.27676
H	1.06056	-3.59931	0.38251
C	3.70289	-2.56594	-1.34668
H	4.20100	-1.93118	-2.08450
H	3.35642	-3.46762	-1.86074
H	4.44361	-2.87096	-0.60482
H	-0.90251	-4.15649	-1.39167
O	2.67153	-0.12323	-0.10924
C	3.40007	0.32395	1.04810
C	3.60331	1.82489	0.85311
H	2.63895	2.34263	0.88285
H	4.22530	2.23384	1.65193
H	4.09997	2.01885	-0.10176
C	4.74716	-0.39138	1.11905
H	5.30462	-0.25420	0.18904
H	5.34060	0.00339	1.94693
H	4.60749	-1.46243	1.28873
C	2.58283	0.07500	2.31566
H	2.55411	-0.98534	2.56941
H	3.03105	0.61179	3.15583
H	1.55831	0.42337	2.16904
K	1.25602	1.73213	-1.46257
N	-1.11531	-0.05397	0.95843
K	-3.27781	1.35123	1.80199

Single point energy calculation of intermediate **58b** as an anion

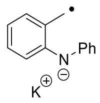

58b

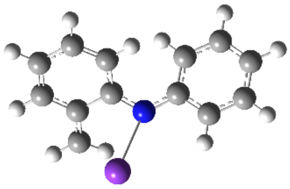

C	0.77892	-1.53191	-0.67777
C	0.82746	-0.38512	0.16932
C	2.13046	-0.03400	0.74382
C	3.27351	-0.80132	0.34338
C	3.17369	-1.88093	-0.49431
C	1.90705	-2.25157	-1.00452
H	-0.17270	-1.80945	-1.11465
H	4.23757	-0.51857	0.75553
H	4.05545	-2.45140	-0.76148
H	1.82455	-3.09846	-1.67666
C	2.25588	1.00020	1.66675
H	1.37820	1.47941	2.08098
H	3.22351	1.23097	2.09652
C	-1.51256	0.08115	0.22351
C	-2.01591	-1.19384	0.56541
C	-2.45180	1.03867	-0.21384
C	-3.36738	-1.48828	0.45045
H	-1.33165	-1.94272	0.94740
C	-3.80141	0.73566	-0.33275
H	-2.09827	2.03696	-0.45256
C	-4.27520	-0.53373	-0.00733
H	-3.71908	-2.47539	0.73110
H	-4.49064	1.49873	-0.67825
H	-5.32822	-0.77068	-0.09636
N	-0.19611	0.46526	0.38892
K	1.17146	2.47546	-0.69193

Intermediate **63**

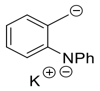

63

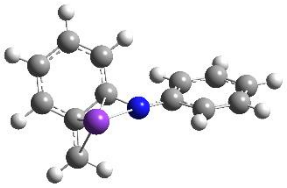

C	0.88838	-1.37743	-0.89990
C	0.81405	-0.32352	0.01665
C	1.95891	-0.04237	0.86848
C	3.08198	-0.90869	0.70044
C	3.11006	-1.95644	-0.20787
C	2.00699	-2.20869	-1.02809
H	0.02479	-1.54413	-1.53903
H	3.94928	-0.73283	1.33291
H	3.99525	-2.58311	-0.27369
H	2.01334	-3.02033	-1.74702
C	1.99062	1.06166	1.75941
H	1.04552	1.48717	2.07941
H	2.80585	1.10196	2.47710
C	-1.53445	0.15407	0.05368
C	-1.98369	-1.18446	0.30416
C	-2.58577	1.10880	-0.14344
C	-3.33122	-1.50745	0.34930
H	-1.24565	-1.95727	0.48134
C	-3.92404	0.76423	-0.09948
H	-2.29470	2.13983	-0.32350
C	-4.33207	-0.55345	0.14364
H	-3.61066	-2.53767	0.55492
H	-4.67123	1.53775	-0.25670
H	-5.38099	-0.82183	0.17446
N	-0.26163	0.57948	0.03855
K	1.48643	2.35351	-0.74379

Single point energy calculation of intermediate **63** as a neutral species

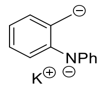

63

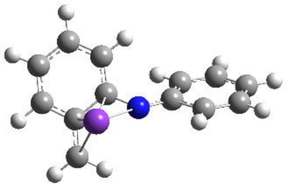

C	0.88838	-1.37743	-0.89990
C	0.81405	-0.32352	0.01665
C	1.95891	-0.04237	0.86848
C	3.08198	-0.90869	0.70044
C	3.11006	-1.95644	-0.20787
C	2.00699	-2.20869	-1.02809
H	0.02479	-1.54413	-1.53903
H	3.94928	-0.73283	1.33291
H	3.99525	-2.58311	-0.27369
H	2.01334	-3.02033	-1.74702
C	1.99062	1.06166	1.75941
H	1.04552	1.48717	2.07941
H	2.80585	1.10196	2.47710
C	-1.53445	0.15407	0.05368
C	-1.98369	-1.18446	0.30416
C	-2.58577	1.10880	-0.14344
C	-3.33122	-1.50745	0.34930
H	-1.24565	-1.95727	0.48134
C	-3.92404	0.76423	-0.09948
H	-2.29470	2.13983	-0.32350
C	-4.33207	-0.55345	0.14364
H	-3.61066	-2.53767	0.55492
H	-4.67123	1.53775	-0.25670
H	-5.38099	-0.82183	0.17446
N	-0.26163	0.57948	0.03855
K	1.48643	2.35351	-0.74379

Substrate **50b** and pentavalent silicate **25b** complex

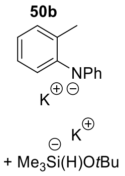

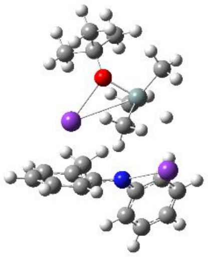

C	-1.29802	1.89523	1.80114
C	-1.71301	1.29591	0.58093
C	-1.67395	2.13022	-0.58682
C	-1.26367	3.45843	-0.46569
C	-0.86127	4.02071	0.74716
C	-0.88216	3.21633	1.88699
H	-1.32741	1.27905	2.69362
H	-1.23434	4.06765	-1.36496
H	-0.54916	5.05638	0.79893
H	-0.58343	3.62178	2.84787
C	-1.97676	1.58239	-1.95854
H	-3.05142	1.51759	-2.14909
H	-1.53289	2.22059	-2.72495
H	-1.57991	0.57012	-2.06959
C	-3.01794	-0.55736	-0.15983
C	-2.93347	-1.87127	-0.68388
C	-4.25218	0.11524	-0.34496
C	-4.01124	-2.47259	-1.32369
H	-1.97872	-2.39056	-0.64171
C	-5.31787	-0.49221	-0.99076
H	-4.35514	1.12385	0.04352
C	-5.21801	-1.79550	-1.48257
H	-3.89849	-3.47649	-1.71985
H	-6.24719	0.05614	-1.10700
H	-6.05555	-2.26426	-1.98381
Si	1.93177	-1.53183	0.39668

C	1.42551	-0.62170	2.01411
H	0.42619	-0.17500	1.93013
H	1.38709	-1.35299	2.82845
H	2.12729	0.15931	2.31862
C	3.21904	-2.91601	0.60952
H	3.12611	-3.64702	-0.20004
H	4.25287	-2.57051	0.65188
H	2.98896	-3.44500	1.53890
C	0.94502	-1.14242	-1.20322
H	1.36079	-0.36091	-1.84841
H	-0.07296	-0.86028	-0.90592
H	0.85037	-2.05333	-1.80322
H	0.76736	-2.65442	0.70768
O	3.15264	-0.14429	0.07461
C	4.19527	-0.00948	-0.86889
C	5.53167	0.02236	-0.11711
H	5.68836	-0.91873	0.41394
H	6.37217	0.17976	-0.79952
H	5.52056	0.83079	0.61841
C	4.00990	1.33146	-1.59647
H	4.00123	2.15395	-0.87331
H	4.82164	1.52119	-2.30332
H	3.07200	1.33312	-2.16280
C	4.23664	-1.12485	-1.92726
H	3.29407	-1.18778	-2.47428
H	5.03247	-0.91640	-2.64753
H	4.44004	-2.09605	-1.47641
K	1.31426	1.73279	0.12396
N	-1.97497	-0.04331	0.56848
K	-1.33347	-2.19636	1.98936

Intermediate **64**, $Me_3SiOtBu$ **49** and H_2 complex

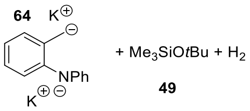

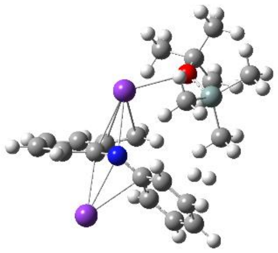

C	-3.15240	1.69986	1.05316
C	-2.17643	1.00718	0.31828
C	-1.58192	1.66573	-0.84412
C	-2.24553	2.87603	-1.25915
C	-3.25494	3.47965	-0.53958
C	-3.71391	2.91733	0.66186
H	-3.46685	1.24499	1.99313
H	-1.88378	3.35249	-2.16655
H	-3.68569	4.40706	-0.90515
H	-4.47577	3.39988	1.26138
C	-0.41573	1.22927	-1.46884
H	0.01616	0.25966	-1.26406
H	-0.03585	1.76985	-2.32953
H	0.24672	-0.85058	-3.45955
C	-1.56272	-1.31962	0.14916
C	-1.16165	-2.52289	0.81171
C	-1.92026	-1.47640	-1.22729
C	-1.05690	-3.73294	0.15402
H	-0.93876	-2.45921	1.87169
C	-1.80327	-2.70703	-1.87286
H	-2.23725	-0.60746	-1.79144
C	-1.36249	-3.84719	-1.20960
H	-0.73691	-4.60937	0.70989
H	-2.05863	-2.76540	-2.92703
H	-1.27080	-4.79470	-1.72574
Si	2.79297	-1.10608	0.57676

C	1.84997	-1.36040	2.17279
H	0.79977	-1.07132	2.07023
H	1.85559	-2.41853	2.44904
H	2.30485	-0.79472	2.99090
C	4.53970	-1.76718	0.76035
H	5.10523	-1.70611	-0.17312
H	5.09710	-1.23870	1.53747
H	4.48657	-2.82357	1.04118
C	1.95349	-2.00150	-0.83188
H	2.50871	-1.92364	-1.76878
H	0.93065	-1.65398	-0.99966
H	1.88340	-3.06233	-0.56873
H	0.60545	-1.12764	-4.04953
O	2.78002	0.57379	0.42037
C	3.50281	1.41940	-0.49446
C	4.95223	1.54548	-0.02790
H	5.47304	0.58880	-0.08618
H	5.48623	2.26263	-0.65595
H	4.98340	1.89788	1.00584
C	2.82089	2.78359	-0.44252
H	2.83943	3.17429	0.57973
H	3.34611	3.49579	-1.08246
H	1.78839	2.69958	-0.79514
C	3.42879	0.85372	-1.91055
H	2.38572	0.68724	-2.19144
H	3.87831	1.55502	-2.61731
H	3.97382	-0.09165	-1.98517
K	0.41442	1.54932	1.34126

Intermediate **64**

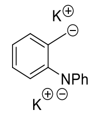

64

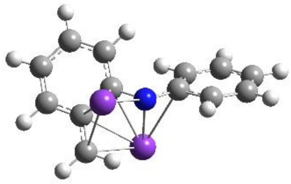

C	-0.97384	-0.90380	1.54398
C	-0.86669	-0.15673	0.36356
C	-1.98210	-0.13637	-0.57100
C	-3.09809	-0.95968	-0.22141
C	-3.15208	-1.70533	0.94303
C	-2.08941	-1.68307	1.85288
H	-0.14608	-0.85773	2.24600
H	-3.94078	-0.98253	-0.90728
H	-4.03193	-2.30721	1.14833
H	-2.12735	-2.25098	2.77460
C	-1.97618	0.65505	-1.74319
H	-1.12867	1.27642	-2.00546
H	-2.83211	0.64466	-2.40718
C	1.49211	0.33536	0.29291
C	1.96355	-0.99047	0.55099
C	2.51791	1.32060	0.14271
C	3.32409	-1.28152	0.62575
H	1.25048	-1.79122	0.71082
C	3.86105	1.01326	0.22968
H	2.20222	2.34044	-0.05395
C	4.29486	-0.29783	0.47129
H	3.62541	-2.30656	0.82099
H	4.59184	1.80696	0.10807
H	5.34885	-0.53403	0.54382
N	0.20833	0.71082	0.08065
K	-1.65260	2.57422	0.47332

K	0.38095	-1.06217	-2.07491

Intermediate **65**

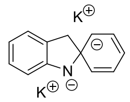

65

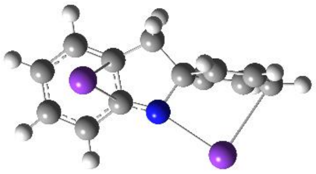

C	-1.03451	0.45527	-0.48690
C	-1.42243	-0.93647	-0.53358
C	-2.70511	-1.32394	-0.84095
C	-3.69811	-0.35614	-1.12672
C	-3.33162	0.99430	-1.12650
C	-2.03909	1.41638	-0.82081
C	-0.24143	-1.71291	-0.01951
H	-2.96772	-2.37896	-0.84262
H	-4.70388	-0.65655	-1.39112
H	-4.07895	1.74219	-1.37618
H	-1.78885	2.47215	-0.82885
H	0.02444	-2.58566	-0.61874
H	-0.42426	-2.06907	1.00572
C	0.92381	-0.66246	0.00054
C	1.77664	-0.76853	1.24337
C	1.81361	-0.81451	-1.21842
C	3.14957	-0.76937	1.23296
H	1.24475	-0.83106	2.19116
C	3.18244	-0.81305	-1.16954
H	1.30362	-0.90646	-2.17512
C	3.91807	-0.71882	0.04338
H	3.66660	-0.82059	2.18991
H	3.72755	-0.89540	-2.10850
H	4.99548	-0.81282	0.06070

N	0.20304	0.67947	-0.06811
K	2.54239	1.86467	-0.04741
K	-2.76192	0.19046	1.80038

Intermediate **66**

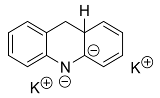

66

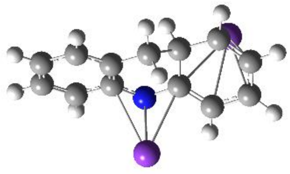

C	4.02481	-1.18349	-0.31626
C	2.90132	-1.15621	-1.14338
C	1.67529	-0.65661	-0.71843
C	1.51289	-0.19394	0.63227
C	2.67775	-0.22383	1.45290
C	3.89176	-0.70180	0.99035
C	0.45788	-0.64695	-1.61266
C	-0.76629	0.29270	0.32448
C	-0.81295	-0.79990	-0.74306
C	-2.11804	-0.78797	-1.50208
H	-2.24240	-1.52318	-2.29257
C	-3.10684	0.15277	-1.27826
C	-3.03697	1.12017	-0.25962
C	-1.80868	1.17529	0.49254
H	0.36117	0.29117	-2.17493
H	4.97153	-1.56513	-0.67778
H	2.97751	-1.52945	-2.16218
H	2.57746	0.12743	2.47522
H	4.74771	-0.70880	1.65814
H	-3.99695	0.11407	-1.90390
H	-3.79674	1.88142	-0.14421
H	-1.70136	1.90949	1.29236
H	0.51962	-1.44816	-2.35863

H	-0.64348	-1.77602	-0.22698
N	0.36904	0.35093	1.12607
K	0.95159	2.57645	-0.15175
K	-3.02048	-1.34333	1.24229

Intermediate **60b**

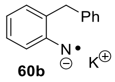

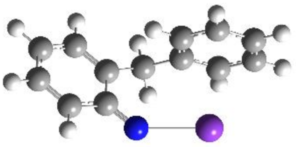

C	2.89242	1.29358	0.31360
C	1.67079	0.83055	-0.30268
C	1.61495	-0.60701	-0.54918
C	2.66596	-1.43302	-0.19193
C	3.82088	-0.92682	0.41976
C	3.92265	0.44619	0.67132
H	2.96381	2.36085	0.49680
H	2.59530	-2.50017	-0.38525
H	4.62790	-1.59655	0.69273
H	4.81652	0.84234	1.14210
C	0.38689	-1.17659	-1.20793
H	0.24010	-0.69971	-2.18114
H	0.54138	-2.24530	-1.39316
C	-0.89706	-1.01324	-0.41900
C	-2.12768	-1.00481	-1.08402
C	-0.89952	-0.90403	0.97493
C	-3.32566	-0.87069	-0.38451
H	-2.14375	-1.09542	-2.16572
C	-2.09364	-0.76636	1.67888
H	0.04681	-0.90397	1.50511
C	-3.31384	-0.74267	1.00348
H	-4.26686	-0.86758	-0.92273

H	-2.07194	-0.67777	2.75919
H	-4.24232	-0.63967	1.55243
N	0.66939	1.63988	-0.59183
K	-1.77345	1.98913	-0.13126

Intermediate **61b** and KO^{t}Bu complex

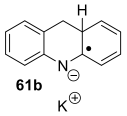

+ KO^{t}Bu complex

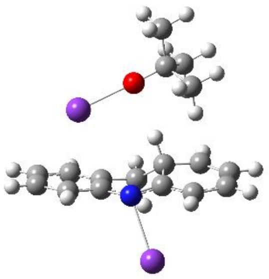

C	1.89860	3.48778	0.88815
C	1.42007	2.41073	1.64257
C	1.31012	1.13458	1.10615
C	1.67166	0.89696	-0.25270
C	2.17635	1.99223	-0.99406
C	2.28727	3.25855	-0.43377
C	0.83111	-0.05158	1.89681
C	0.83644	-1.29504	-0.26217
C	0.02029	-0.97664	0.97634
C	-0.48485	-2.20615	1.67586
H	-0.93318	-2.06597	2.65527
C	-0.46658	-3.43677	1.09915
C	0.10804	-3.63814	-0.18775
C	0.76275	-2.57432	-0.82402
H	1.69108	-0.60768	2.29605
H	1.98017	4.47422	1.32738
H	1.12661	2.56656	2.67696
H	2.45914	1.81793	-2.02694
H	2.67286	4.07573	-1.03407
H	-0.89626	-4.28324	1.62514

H	0.06155	-4.61167	-0.65944
H	1.20626	-2.72296	-1.80699
H	0.22677	0.26656	2.75090
H	-0.84620	-0.37439	0.62912
C	-3.51116	-0.01450	-0.35820
C	-4.80114	0.52656	-1.00909
H	-5.14694	1.40617	-0.45741
H	-4.58903	0.83053	-2.03872
H	-5.60790	-0.21496	-1.02428
C	-3.05740	-1.25957	-1.15024
H	-2.81286	-0.96618	-2.17609
H	-2.16004	-1.69008	-0.69519
H	-3.83096	-2.03538	-1.18547
C	-3.84683	-0.45372	1.08268
H	-2.95010	-0.86146	1.55887
H	-4.17861	0.41542	1.65900
H	-4.63267	-1.21674	1.11686
O	-2.53058	0.94996	-0.35614
K	-0.90346	2.62418	-0.55570
N	1.58816	-0.33455	-0.85256
K	3.71185	-1.90133	-0.79947

Intermediate **62b** and ^{t}BuOH complex

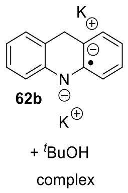

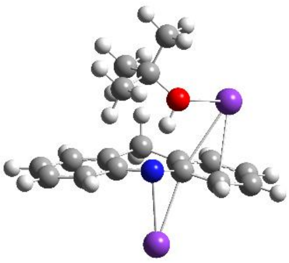

C	3.37771	-1.68126	0.61825
C	2.27103	-1.54960	1.49791
C	0.98779	-1.17050	0.95821

C	0.85558	-0.82877	-0.38738
C	1.99318	-0.94293	-1.26121
C	3.24881	-1.38286	-0.72571
C	-0.17251	-0.96390	1.89325
C	-1.48037	-0.62680	-0.23947
C	-1.48018	-0.97122	1.14098
C	-2.68580	-1.21956	1.79418
H	-2.65715	-1.48013	2.84939
C	-3.91405	-1.12002	1.14366
C	-3.92745	-0.76237	-0.20590
C	-2.73857	-0.53118	-0.88281
H	-0.17871	-1.74003	2.66993
H	4.33622	-2.01494	1.00495
H	2.34469	-1.85069	2.53718
H	1.87962	-0.66360	-2.30254
H	4.10608	-1.48354	-1.38426
H	-4.83769	-1.31107	1.67645
H	-4.87048	-0.67141	-0.73473
H	-2.74484	-0.26393	-1.93540
H	-0.05772	-0.00911	2.44575
H	0.15763	1.33231	-0.97180
C	-0.43417	3.12291	-0.34727
C	0.23638	4.48959	-0.28015
H	1.06133	4.47188	0.43783
H	0.63464	4.76225	-1.25996
H	-0.47649	5.25478	0.03517
C	-1.56201	3.12177	-1.37774
H	-1.17099	3.38687	-2.36289
H	-2.01409	2.12762	-1.43451
H	-2.33939	3.83901	-1.10290
C	-0.97187	2.71906	1.02724

H	-1.42585	1.72510	0.98338
H	-0.15948	2.69915	1.76027
H	-1.72772	3.42890	1.37358
O	0.58117	2.20291	-0.75448
K	2.67830	1.26890	0.57411
N	-0.34264	-0.42613	-0.98182
K	-0.24312	-3.07912	-1.49899

Intermediate **62b**

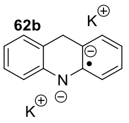

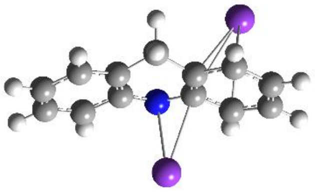

C	-4.00939	-1.00557	-0.42777
C	-2.79837	-1.32408	-1.04061
C	-1.57480	-0.92936	-0.50611
C	-1.53309	-0.15636	0.69076
C	-2.77606	0.14919	1.30178
C	-3.98181	-0.27074	0.75990
C	-0.26600	-1.37467	-1.10549
C	0.73848	0.32331	0.42616
C	0.82162	-0.38959	-0.78125
C	2.04310	-0.40471	-1.54742
H	2.06728	-0.92677	-2.49783
C	3.10799	0.45083	-1.16217
C	3.01802	1.20293	-0.00357
C	1.85822	1.13096	0.83283
H	0.00495	-2.38483	-0.72181
H	-4.94880	-1.32885	-0.85955
H	-2.79778	-1.91168	-1.95552
H	-2.75366	0.72629	2.22113

H	-4.90964	-0.01876	1.26317
H	4.00314	0.50856	-1.77406
H	3.84470	1.84491	0.28531
H	1.76983	1.72038	1.73831
H	-0.36453	-1.50365	-2.18986
N	-0.38768	0.36553	1.22627
K	-0.72225	2.48883	-0.41026
K	2.77019	-1.63079	1.00419

Substrate **67** and Me_3Si radical complex

67

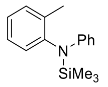

+ Me_3Si•

complex

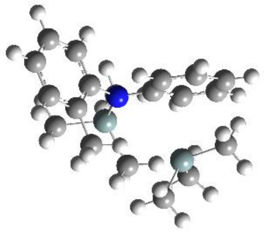

C	-2.60123	1.11783	-1.55053
C	-1.69639	0.86492	-0.52175
C	-1.15933	1.92158	0.22689
C	-1.56321	3.22205	-0.08082
C	-2.47534	3.47469	-1.10047
C	-2.99743	2.41881	-1.84075
H	-2.98898	0.27721	-2.11592
H	-1.15166	4.04843	0.48944
H	-2.77340	4.49351	-1.31837
H	-3.70548	2.60567	-2.63929
C	-0.18054	1.65582	1.33925
H	0.45633	0.80269	1.10106
H	0.45219	2.52766	1.51557
H	-0.70311	1.42693	2.27444
C	-0.19806	-0.99817	-0.90033
C	0.66255	-0.16263	-1.63294

C	0.11553	-2.36896	-0.84764
C	1.78852	-0.67822	-2.26504
H	0.45639	0.89812	-1.70196
C	1.24494	-2.87071	-1.47983
H	-0.53082	-3.05512	-0.31651
C	2.09806	-2.03193	-2.19294
H	2.43456	-0.00077	-2.81332
H	1.45464	-3.93246	-1.41590
H	2.97798	-2.42702	-2.68549
N	-1.32549	-0.49204	-0.23713
Si	3.48459	0.80894	0.56966
C	4.90454	0.33363	-0.57742
H	5.28171	1.19618	-1.13122
H	5.73257	-0.08043	0.00866
H	4.58568	-0.42446	-1.29594
C	2.78732	-0.73716	1.39184
H	2.08589	-0.48268	2.19051
H	2.26612	-1.36571	0.66454
H	3.59950	-1.32554	1.83322
C	4.10679	2.01096	1.88647
H	3.30019	2.30764	2.56104
H	4.89283	1.53929	2.48672
H	4.52306	2.91480	1.43622
Si	-2.22563	-1.34878	1.03602
C	-3.45362	-0.12319	1.73910
H	-2.97100	0.76175	2.15903
H	-4.16177	0.20987	0.97627
H	-4.02034	-0.61181	2.53704
C	-3.20391	-2.79134	0.33600
H	-2.59429	-3.64936	0.04864
H	-3.92249	-3.13333	1.08697

H	-3.76859	-2.46823	-0.54287
C	-1.03913	-1.91779	2.37248
H	-0.20682	-2.50320	1.97629
H	-0.62066	-1.05464	2.89668
H	-1.56763	-2.53425	3.10536

Intermediate **69** and Me_3SiH complex

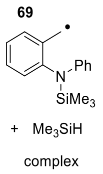

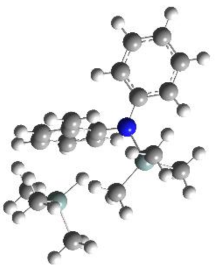

C	-1.16797	1.98671	1.62280
C	-0.84400	1.18720	0.53787
C	0.07060	1.65308	-0.45060
C	0.63323	2.94249	-0.26835
C	0.30583	3.72339	0.82388
C	-0.59917	3.25094	1.77809
H	-1.87308	1.60325	2.35256
H	1.33620	3.30888	-1.00886
H	0.75175	4.70427	0.93895
H	-0.85903	3.86050	2.63487
C	0.40426	0.86579	-1.57174
H	-0.05102	-0.10139	-1.73089
H	2.75400	-1.24443	-1.05522
C	-2.59301	-0.24372	-0.33098
C	-3.29798	-1.45963	-0.37363
C	-3.11198	0.83570	-1.06726
C	-4.45677	-1.58913	-1.12767
H	-2.95177	-2.31240	0.19510
C	-4.27530	0.69359	-1.81460

H	-2.60365	1.79119	-1.05385
C	-4.95907	-0.51660	-1.85849
H	-4.97405	-2.54189	-1.13515
H	-4.64691	1.54676	-2.37097
H	-5.86423	-0.62102	-2.44332
Si	4.06993	-0.61321	-0.75867
C	3.84157	1.24387	-0.58242
H	3.50583	1.69187	-1.52112
H	3.10548	1.48913	0.18786
H	4.79012	1.71345	-0.30611
C	4.74786	-1.32396	0.84353
H	4.82298	-2.41292	0.79449
H	5.74755	-0.92615	1.03915
H	4.11064	-1.06458	1.69199
C	5.25162	-0.98169	-2.17262
H	5.40286	-2.05728	-2.29087
H	6.22639	-0.52355	-1.98423
H	4.87180	-0.58787	-3.11835
N	-1.41616	-0.11913	0.42363
Si	-0.52574	-1.46598	1.17719
C	1.07043	-0.74223	1.83236
H	1.68538	-1.55276	2.23474
H	1.63404	-0.24886	1.03654
H	0.89528	-0.01603	2.62958
C	-0.11868	-2.78126	-0.09623
H	-0.99249	-3.17006	-0.62111
H	0.57413	-2.37752	-0.83913
H	0.37822	-3.62144	0.39827
C	-1.49178	-2.18430	2.61687
H	-1.74610	-1.39171	3.32591
H	-2.41716	-2.68287	2.32400

H	-0.87015	-2.91609	3.14147
H	1.09321	1.24262	-2.31574

Intermediate **67**

67

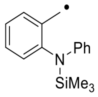

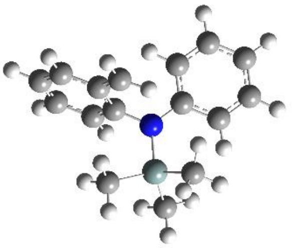

C	1.70753	-0.60035	-1.45000
C	1.12771	-0.39424	-0.20783
C	1.80069	-0.81044	0.97766
C	3.07725	-1.41226	0.83043
C	3.64580	-1.59978	-0.41486
C	2.96283	-1.19770	-1.56604
H	1.16294	-0.28128	-2.33225
H	3.60613	-1.72735	1.72350
H	4.62265	-2.06131	-0.49793
H	3.40398	-1.34615	-2.54402
C	1.22045	-0.63784	2.24981
H	1.74766	-0.96095	3.13754
C	-1.28877	-0.54087	-0.11444
C	-1.20678	-1.94090	-0.01578
C	-2.56885	0.03300	-0.21040
C	-2.35681	-2.72115	-0.00371
H	-0.23847	-2.42022	0.05104
C	-3.70986	-0.75845	-0.19301
H	-2.68153	1.10476	-0.31145
C	-3.61916	-2.14316	-0.08777
H	-2.25767	-3.79799	0.07443
H	-4.68044	-0.28162	-0.27039

H	-4.51121	-2.75666	-0.07725
N	-0.13929	0.26229	-0.11568
Si	-0.12846	2.04060	-0.01279
C	-0.64494	2.78665	-1.65414
H	-1.63452	2.44889	-1.97014
H	-0.66714	3.87807	-1.58381
H	0.06957	2.51398	-2.43545
C	1.63294	2.54120	0.37042
H	2.33186	2.20765	-0.39992
H	1.68381	3.63241	0.42778
H	1.96273	2.13422	1.32968
C	-1.26511	2.61274	1.37301
H	-0.80581	3.45932	1.89054
H	-2.24318	2.93680	1.01166
H	-1.42418	1.81762	2.10588
H	0.23681	-0.20334	2.35927

Intermediate **82**

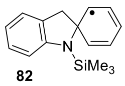

82

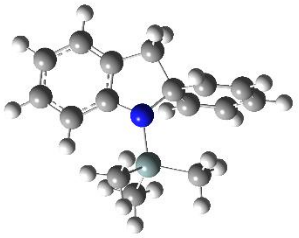

C	1.46646	-0.05475	-0.17105
C	1.68890	-1.43762	-0.25765
C	2.95114	-1.97175	-0.08715
C	4.02759	-1.11759	0.17556
C	3.81099	0.25319	0.25643
C	2.53752	0.80183	0.08951
C	0.38978	-2.10384	-0.61101
H	3.10460	-3.04330	-0.15829

H	5.02235	-1.52157	0.31660
H	4.64335	0.91655	0.46209
H	2.40274	1.87240	0.17117
H	0.35350	-2.32170	-1.68389
C	-0.67472	-1.00590	-0.26361
C	-1.80220	-1.06950	-1.24875
C	-1.14894	-1.19505	1.15289
C	-3.06602	-1.43033	-0.89979
H	-1.54505	-0.85426	-2.28101
C	-2.43150	-1.52382	1.46341
H	-0.39481	-1.07965	1.92642
C	-3.41279	-1.67436	0.45142
H	-3.83128	-1.50454	-1.66437
H	-2.71406	-1.66544	2.50080
H	-4.42709	-1.94615	0.71270
N	0.11781	0.26519	-0.36771
Si	-0.54859	1.87542	-0.02774
C	-0.16828	2.36530	1.74532
H	-0.72361	1.72258	2.43428
H	0.89294	2.27940	1.98800
H	-0.47436	3.39934	1.92840
C	-2.40366	1.87561	-0.25973
H	-2.69271	1.67247	-1.29233
H	-2.90472	1.15243	0.38700
H	-2.76524	2.87488	0.00391
C	0.19224	3.10063	-1.23973
H	-0.03001	2.78965	-2.26389
H	-0.26206	4.08330	-1.08151
H	1.27313	3.21736	-1.15204
H	0.18897	-3.02806	-0.06809

Intermediate **83**

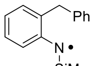

83

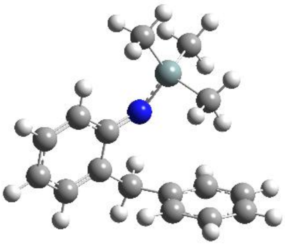

C	-2.60771	-0.58972	-0.44698
C	-1.37674	-0.51306	0.28921
C	-0.77320	-1.76049	0.70997
C	-1.39105	-2.95784	0.40569
C	-2.60201	-2.99118	-0.29638
C	-3.20514	-1.80031	-0.71970
H	-3.07493	0.33496	-0.76196
H	-0.92669	-3.88911	0.71286
H	-3.07004	-3.94300	-0.51699
H	-4.14186	-1.83368	-1.26339
C	0.55047	-1.70656	1.42153
H	0.45524	-1.07026	2.30479
C	1.67716	-1.15887	0.56245
C	2.64541	-0.32473	1.12241
C	1.78365	-1.49197	-0.78827
C	3.69768	0.16388	0.35434
H	2.56539	-0.04576	2.16813
C	2.83294	-1.00253	-1.56150
H	1.03381	-2.13210	-1.24252
C	3.79469	-0.17272	-0.99326
H	4.43752	0.81454	0.80653
H	2.89693	-1.26823	-2.61054
H	4.60947	0.21174	-1.59533
N	-0.75854	0.60805	0.60743
Si	-0.78765	2.25279	0.02705

C	-0.42292	3.33054	1.51402
H	0.51319	3.02102	1.98489
H	-0.33113	4.38002	1.22183
H	-1.21891	3.25550	2.25921
C	0.62207	2.30407	-1.21049
H	0.81798	3.32713	-1.54361
H	1.53248	1.89713	-0.76167
H	0.38185	1.69796	-2.08828
C	-2.37103	2.86167	-0.79262
H	-2.56448	2.35720	-1.74252
H	-3.24210	2.72966	-0.14521
H	-2.27463	3.93147	-1.00221
H	0.81227	-2.71277	1.76273

Intermediate **84**

84

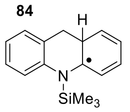

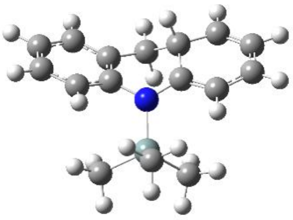

C	3.61569	-1.77828	0.15153
C	2.45814	-2.29861	-0.42332
C	1.26121	-1.59324	-0.38067
C	1.21962	-0.34805	0.26478
C	2.36849	0.15471	0.87581
C	3.56473	-0.55158	0.80559
C	-0.04138	-2.10868	-0.92170
C	-1.18680	-0.33143	0.38922
C	-1.12151	-1.81904	0.13516
C	-2.46848	-2.40185	-0.18221
H	-2.48450	-3.40671	-0.59144

C	-3.62182	-1.75061	0.11428
C	-3.59842	-0.43453	0.64969
C	-2.37937	0.26169	0.73748
H	-0.31000	-1.59302	-1.85176
H	4.54411	-2.33416	0.10354
H	2.48165	-3.26587	-0.91461
H	2.31981	1.09339	1.41653
H	4.45236	-0.14860	1.27875
H	-4.57642	-2.23141	-0.06936
H	-4.52050	0.05754	0.92949
H	-2.38899	1.29801	1.05494
H	0.01768	-3.17826	-1.13303
H	-0.75722	-2.29555	1.06710
N	0.00403	0.38828	0.27411
Si	0.00702	2.11109	-0.24765
C	-0.11481	3.25699	1.22860
H	-0.09918	4.29779	0.89223
H	-1.03474	3.10161	1.79650
H	0.72823	3.11177	1.90884
C	1.60039	2.42806	-1.18219
H	1.48542	3.36242	-1.73940
H	2.47374	2.52759	-0.53730
H	1.79979	1.62896	-1.90127
C	-1.41651	2.37818	-1.43828
H	-1.50865	1.52762	-2.11925
H	-2.37930	2.52213	-0.94829
H	-1.20484	3.26909	-2.03650

Intermediate **84** and KO*t*Bu complex

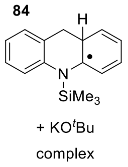

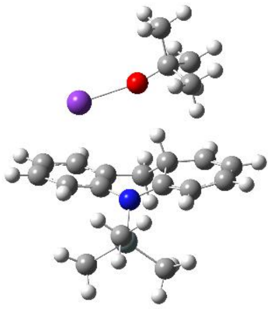

C	0.83482	3.68263	1.08736
C	0.36876	2.60696	1.84564
C	0.57153	1.29349	1.43367
C	1.24405	1.04712	0.22133
C	1.66361	2.12765	-0.56333
C	1.48055	3.43601	-0.12179
C	0.09940	0.09253	2.19992
C	0.57962	-1.27197	0.17007
C	-0.46858	-0.92151	1.19359
C	-1.05172	-2.14537	1.83694
H	-1.63371	-1.99586	2.74073
C	-0.95042	-3.37625	1.27246
C	-0.19251	-3.57009	0.08722
C	0.59226	-2.51475	-0.41686
H	0.93972	-0.36252	2.73945
H	0.68990	4.69823	1.43510
H	-0.14962	2.78663	2.78186
H	2.13783	1.93812	-1.51976
H	1.83366	4.26014	-0.73076
H	-1.44813	-4.22417	1.73002
H	-0.16464	-4.53912	-0.39325
H	1.24761	-2.70530	-1.25911
H	-0.65944	0.37374	2.93316
H	-1.29250	-0.39322	0.65777

C	-3.81844	-0.29674	-0.64172
C	-4.99757	0.21925	-1.49147
H	-5.44880	1.08754	-1.00143
H	-4.62947	0.53292	-2.47342
H	-5.77391	-0.53988	-1.63786
C	-3.21387	-1.52710	-1.34812
H	-2.82753	-1.22847	-2.32793
H	-2.38125	-1.92293	-0.75967
H	-3.94690	-2.32883	-1.49231
C	-4.36853	-0.73999	0.72885
H	-3.54946	-1.12412	1.34355
H	-4.80745	0.12241	1.23976
H	-5.13181	-1.52121	0.64252
O	-2.86887	0.68904	-0.48777
K	-1.52762	2.58064	-0.75453
N	1.51634	-0.28156	-0.17004
Si	3.19374	-0.73851	-0.64938
C	3.32312	-0.95786	-2.50433
H	3.05962	-0.03599	-3.02926
H	4.35149	-1.21509	-2.77454
H	2.67048	-1.75190	-2.87333
C	3.67457	-2.30117	0.26607
H	3.28464	-3.21273	-0.18596
H	4.76600	-2.37039	0.28438
H	3.31970	-2.26050	1.29957
C	4.35438	0.62304	-0.08426
H	4.34253	1.50821	-0.72058
H	4.12957	0.93626	0.93886
H	5.37072	0.21841	-0.09509

Intermediate **85** and HO*t*Bu complex

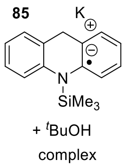

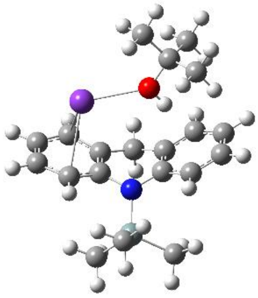

C	1.22282	3.69349	0.82895
C	0.60547	2.75086	1.69802
C	0.77763	1.35110	1.40289
C	1.37012	0.95727	0.22169
C	1.81299	1.91277	-0.74378
C	1.81184	3.29440	-0.35242
C	0.32644	0.29159	2.37435
C	0.47987	-1.26183	0.42110
C	-0.16019	-0.92349	1.62815
C	-1.20909	-1.71842	2.08926
H	-1.70561	-1.44232	3.01452
C	-1.61692	-2.85473	1.39228
C	-0.97383	-3.19169	0.20330
C	0.05364	-2.39232	-0.28685
H	1.15096	0.00577	3.04573
H	1.22534	4.74578	1.09735
H	0.21759	3.05052	2.66389
H	2.29554	1.58882	-1.65571
H	2.26461	4.03374	-1.00508
H	-2.42860	-3.46540	1.76986
H	-1.28888	-4.06293	-0.35951
H	0.51702	-2.62529	-1.23909
H	-0.47303	0.68050	3.01409
H	-2.00857	-0.48329	0.04857

C	-3.80475	-0.13500	-0.71301
C	-4.40291	1.06017	-1.44245
H	-4.37349	1.94854	-0.80581
H	-3.84695	1.25975	-2.36222
H	-5.44378	0.86447	-1.70648
C	-3.81844	-1.37789	-1.59782
H	-3.26852	-1.18911	-2.52246
H	-3.34705	-2.21633	-1.07822
H	-4.84350	-1.66000	-1.84927
C	-4.53512	-0.39293	0.60217
H	-4.07644	-1.23185	1.13231
H	-4.48551	0.49149	1.24145
H	-5.58437	-0.63500	0.41795
O	-2.44238	0.23391	-0.43510
K	-1.06887	2.46350	-0.70837
N	1.50020	-0.43614	-0.07843
Si	3.05203	-1.08407	-0.65543
C	3.15275	-1.10909	-2.52983
H	3.12173	-0.10428	-2.95661
H	4.08780	-1.58064	-2.84713
H	2.32621	-1.68269	-2.95800
C	3.23380	-2.84008	-0.01476
H	2.62565	-3.56653	-0.55569
H	4.28145	-3.13796	-0.11951
H	2.97113	-2.90002	1.04476
C	4.43203	-0.04772	0.07089
H	4.41377	0.97915	-0.29610
H	4.34573	-0.01881	1.16069
H	5.39868	-0.49280	-0.18224

Intermediate **85**

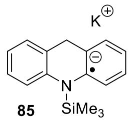

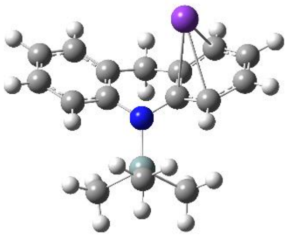

C	2.22549	-3.39764	0.09124
C	1.15589	-3.09908	-0.74981
C	0.61678	-1.81609	-0.80188
C	1.13976	-0.81471	0.03465
C	2.20358	-1.12090	0.88993
C	2.75217	-2.39921	0.90585
C	-0.50166	-1.42978	-1.73667
C	-0.85600	0.48980	-0.21258
C	-1.41846	-0.44632	-1.05456
C	-2.84588	-0.50217	-1.23404
H	-3.26755	-1.17829	-1.96756
C	-3.62460	0.55011	-0.67163
C	-3.05753	1.48620	0.16695
C	-1.66509	1.42424	0.50901
H	-0.07686	-0.98746	-2.65022
H	2.64163	-4.39770	0.11091
H	0.74171	-3.86801	-1.39478
H	2.58477	-0.35670	1.55865
H	3.57823	-2.61845	1.57249
H	-4.68410	0.61197	-0.90098
H	-3.67614	2.26832	0.59505
H	-1.20728	2.18225	1.12991
H	-1.05905	-2.31862	-2.04807
N	0.55424	0.46632	0.02265
K	-2.54706	-1.12822	1.59963

Si	1.51182	1.95853	-0.04131
C	1.69692	2.75310	1.65024
H	2.32235	3.64807	1.57839
H	0.73231	3.04974	2.06851
H	2.17340	2.06804	2.35672
C	0.67927	3.13841	-1.23331
H	-0.29653	3.46879	-0.87489
H	1.31187	4.01840	-1.38143
H	0.53655	2.65460	-2.20351
C	3.21446	1.53641	-0.70989
H	3.84959	1.02301	0.01342
H	3.13661	0.90556	-1.59946
H	3.71702	2.46478	-0.99740

Single point energy calculation of intermediate **69** as an anion

69

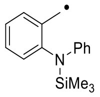

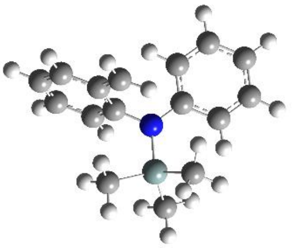

C	1.70753	-0.60035	-1.45000
C	1.12771	-0.39424	-0.20783
C	1.80069	-0.81044	0.97766
C	3.07725	-1.41226	0.83043
C	3.64580	-1.59978	-0.41486
C	2.96283	-1.19770	-1.56604
H	1.16294	-0.28128	-2.33225
H	3.60613	-1.72735	1.72350
H	4.62265	-2.06131	-0.49793
H	3.40398	-1.34615	-2.54402
C	1.22045	-0.63784	2.24981

H	1.74766	-0.96095	3.13754
C	-1.28877	-0.54087	-0.11444
C	-1.20678	-1.94090	-0.01578
C	-2.56885	0.03300	-0.21040
C	-2.35681	-2.72115	-0.00371
H	-0.23847	-2.42022	0.05104
C	-3.70986	-0.75845	-0.19301
H	-2.68153	1.10476	-0.31145
C	-3.61916	-2.14316	-0.08777
H	-2.25767	-3.79799	0.07443
H	-4.68044	-0.28162	-0.27039
H	-4.51121	-2.75666	-0.07725
N	-0.13929	0.26229	-0.11568
Si	-0.12846	2.04060	-0.01279
C	-0.64494	2.78665	-1.65414
H	-1.63452	2.44889	-1.97014
H	-0.66714	3.87807	-1.58381
H	0.06957	2.51398	-2.43545
C	1.63294	2.54120	0.37042
H	2.33186	2.20765	-0.39992
H	1.68381	3.63241	0.42778
H	1.96273	2.13422	1.32968
C	-1.26511	2.61274	1.37301
H	-0.80581	3.45932	1.89054
H	-2.24318	2.93680	1.01166
H	-1.42418	1.81762	2.10588
H	0.23681	-0.20334	2.35927

Intermediate **71**

71

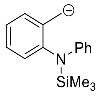

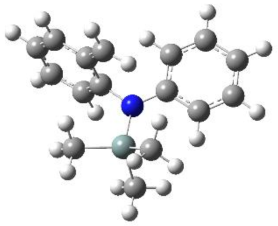

C	1.78605	-0.55858	-1.44686
C	1.13181	-0.42889	-0.23520
C	1.70814	-0.88431	1.01032
C	3.00854	-1.49327	0.85419
C	3.64101	-1.61019	-0.36431
C	3.05167	-1.14515	-1.55405
H	1.27992	-0.17961	-2.33209
H	3.50307	-1.86153	1.74912
H	4.62294	-2.07546	-0.40070
H	3.54892	-1.24185	-2.51073
C	1.08354	-0.73538	2.23784
H	1.55814	-1.10530	3.13947
C	-1.30395	-0.50994	-0.16892
C	-1.25741	-1.91725	-0.09791
C	-2.57749	0.09113	-0.22239
C	-2.42310	-2.66881	-0.07975
H	-0.29124	-2.40276	-0.04683
C	-3.73752	-0.67472	-0.20210
H	-2.66713	1.16822	-0.29286
C	-3.67834	-2.06288	-0.13043
H	-2.34744	-3.74932	-0.01794
H	-4.69851	-0.17337	-0.24957
H	-4.58370	-2.65745	-0.11538
N	-0.14137	0.25141	-0.20253
Si	-0.07073	2.00387	0.01517
C	-0.62118	2.87923	-1.56111

H	-1.63947	2.61093	-1.85331
H	-0.58237	3.96494	-1.42770
H	0.04603	2.61534	-2.38639
C	1.70786	2.46669	0.36063
H	2.34745	2.25433	-0.49902
H	1.77087	3.53483	0.59060
H	2.08188	1.89482	1.21376
C	-1.17017	2.56328	1.44599
H	-0.66292	3.35335	2.00705
H	-2.13464	2.95472	1.11317
H	-1.35695	1.73324	2.13149
H	0.08826	-0.31798	2.31759

Single point energy calculation of intermediate **71** as a neutral molecule

71

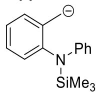

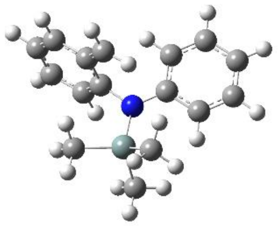

C	1.78605	-0.55858	-1.44686
C	1.13181	-0.42889	-0.23520
C	1.70814	-0.88431	1.01032
C	3.00854	-1.49327	0.85419
C	3.64101	-1.61019	-0.36431
C	3.05167	-1.14515	-1.55405
H	1.27992	-0.17961	-2.33209
H	3.50307	-1.86153	1.74912
H	4.62294	-2.07546	-0.40070
H	3.54892	-1.24185	-2.51073
C	1.08354	-0.73538	2.23784
H	1.55814	-1.10530	3.13947
C	-1.30395	-0.50994	-0.16892

C	-1.25741	-1.91725	-0.09791
C	-2.57749	0.09113	-0.22239
C	-2.42310	-2.66881	-0.07975
H	-0.29124	-2.40276	-0.04683
C	-3.73752	-0.67472	-0.20210
H	-2.66713	1.16822	-0.29286
C	-3.67834	-2.06288	-0.13043
H	-2.34744	-3.74932	-0.01794
H	-4.69851	-0.17337	-0.24957
H	-4.58370	-2.65745	-0.11538
N	-0.14137	0.25141	-0.20253
Si	-0.07073	2.00387	0.01517
C	-0.62118	2.87923	-1.56111
H	-1.63947	2.61093	-1.85331
H	-0.58237	3.96494	-1.42770
H	0.04603	2.61534	-2.38639
C	1.70786	2.46669	0.36063
H	2.34745	2.25433	-0.49902
H	1.77087	3.53483	0.59060
H	2.08188	1.89482	1.21376
C	-1.17017	2.56328	1.44599
H	-0.66292	3.35335	2.00705
H	-2.13464	2.95472	1.11317
H	-1.35695	1.73324	2.13149
H	0.08826	-0.31798	2.31759

Intermediate **73**

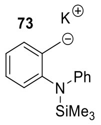

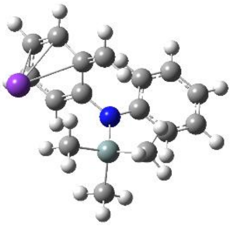

C	-1.23264	-0.73090	1.50441
C	-0.66502	-0.47927	0.27096
C	-1.23375	-1.02728	-0.95322
C	-2.25207	-2.03535	-0.72068
C	-2.77793	-2.28568	0.53061
C	-2.34264	-1.58150	1.66931
H	-0.76677	-0.27207	2.37290
H	-2.61536	-2.59390	-1.57813
H	-3.55035	-3.04275	0.63324
H	-2.74536	-1.78816	2.65164
C	-0.85432	-0.61688	-2.21220
H	-1.26736	-1.09971	-3.09007
C	1.74764	-0.47021	0.11534
C	1.73371	-1.87406	0.02172
C	3.00451	0.16243	0.13968
C	2.91813	-2.59561	-0.04978
H	0.78678	-2.39739	0.00244
C	4.18243	-0.57135	0.06703
H	3.07828	1.23907	0.22673
C	4.15564	-1.95831	-0.03006
H	2.86735	-3.67625	-0.12486
H	5.13044	-0.04560	0.09301
H	5.07480	-2.52808	-0.08598
N	0.55917	0.27468	0.19368
Si	0.53001	2.03539	0.07629

C	1.13897	2.83207	1.66457
H	2.16079	2.53921	1.91489
H	1.11318	3.92237	1.57629
H	0.49534	2.54451	2.50056
C	-1.25852	2.54776	-0.16887
H	-1.85620	2.32813	0.71989
H	-1.30376	3.62653	-0.33993
H	-1.65745	2.05044	-1.05888
C	1.53681	2.62978	-1.40127
H	0.95335	3.34316	-1.98936
H	2.46342	3.12453	-1.10070
H	1.79962	1.79218	-2.05200
H	-0.02440	0.06428	-2.34996
K	-3.59349	0.46198	-0.21215

Substrate **67** and $Me_3Si(H)O\textit{t}BuK$ **25b** complex

67

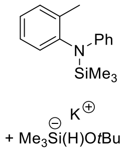

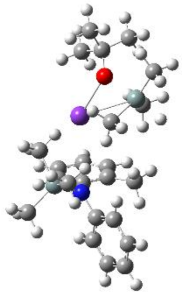

C	-1.46929	-2.43291	0.18495
C	-1.59719	-1.07359	0.50565
C	-1.03965	-0.60014	1.71870
C	-0.36474	-1.50892	2.53805
C	-0.21565	-2.85136	2.19563
C	-0.77485	-3.31405	1.00901
H	-1.94953	-2.81052	-0.70963
H	0.06411	-1.14548	3.46603
H	0.31641	-3.52583	2.85549

H	-0.69805	-4.35948	0.73399
C	-1.16625	0.83740	2.14680
H	-2.17889	1.05105	2.50030
H	-0.46980	1.05680	2.95585
H	-0.96083	1.52024	1.32005
C	-3.39264	0.50793	0.20532
C	-3.54401	1.88427	0.02975
C	-4.34704	-0.19410	0.94639
C	-4.63990	2.54416	0.57578
H	-2.78017	2.43569	-0.50522
C	-5.43077	0.47251	1.50705
H	-4.22715	-1.26344	1.08523
C	-5.58546	1.84347	1.31907
H	-4.74190	3.61413	0.43739
H	-6.16026	-0.08259	2.08523
H	-6.43149	2.36191	1.75362
Si	2.59586	1.73787	0.50728
C	1.17109	1.52355	-0.76602
H	0.29873	0.98270	-0.37364
H	0.80101	2.52480	-1.00493
H	1.50232	1.05234	-1.69454
C	3.85281	3.09244	0.05537
H	4.40505	3.40899	0.94636
H	4.56766	2.81504	-0.72013
H	3.28317	3.96245	-0.28497
C	2.65802	0.65301	2.10328
H	3.14278	-0.32721	2.03586
H	1.64191	0.52711	2.48993
H	3.19430	1.22793	2.86629
H	1.73758	2.78109	1.33659
O	3.51480	0.33444	-0.47563

C	4.84229	-0.13081	-0.44587
C	5.50962	0.18924	-1.79072
H	5.53908	1.26956	-1.94680
H	6.53274	-0.19642	-1.83672
H	4.92946	-0.25586	-2.60341
C	4.81737	-1.65968	-0.26827
H	4.23461	-2.12165	-1.07303
H	5.82250	-2.08851	-0.29933
H	4.37697	-1.92532	0.69980
C	5.69413	0.46286	0.69017
H	5.24416	0.26603	1.66524
H	6.69068	0.01212	0.67972
H	5.81224	1.54028	0.57599
K	1.63708	-1.35609	-0.03576
N	-2.27490	-0.18368	-0.36483
Si	-2.27349	-0.35514	-2.14051
C	-2.37222	1.33053	-2.94468
H	-3.33068	1.82341	-2.77365
H	-1.57034	1.98345	-2.59022
H	-2.24962	1.20188	-4.02452
C	-3.75780	-1.37407	-2.66671
H	-4.67825	-0.91418	-2.29558
H	-3.82328	-1.42118	-3.75728
H	-3.71894	-2.39778	-2.28605
C	-0.67272	-1.14988	-2.71387
H	0.18119	-0.52374	-2.44531
H	-0.5097	-2.16910	-2.36213
H	-0.70247	-1.18432	-3.80741

Intermediate **73**, $Me_3SiOtBu$ **49** and H_2 complex

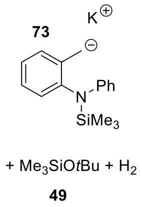

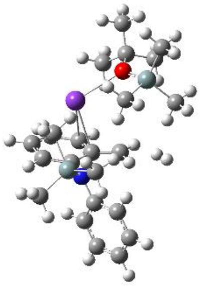

C	-1.70663	2.38389	0.84331
C	-1.52328	1.27098	0.04187
C	-0.55503	1.27936	-1.04663
C	0.06096	2.56845	-1.27727
C	-0.11596	3.64483	-0.43199
C	-0.96980	3.56977	0.68184
H	-2.45130	2.33011	1.63295
H	0.72621	2.66135	-2.13074
H	0.41105	4.57114	-0.64254
H	-1.13827	4.42181	1.32653
C	-0.17718	0.14677	-1.74192
H	-0.69460	-0.79430	-1.61790
H	0.53490	0.23392	-2.55567
H	-0.37218	-3.07598	-0.95564
C	-3.17031	-0.30317	-0.75054
C	-3.39865	-1.64466	-1.07584
C	-3.86862	0.68224	-1.45996
C	-4.32381	-1.99115	-2.05585
H	-2.82139	-2.41526	-0.58268
C	-4.77904	0.33095	-2.44869
H	-3.68122	1.72507	-1.23187
C	-5.02197	-1.00760	-2.74849
H	-4.48064	-3.03760	-2.29241
H	-5.30697	1.11076	-2.98605
H	-5.73407	-1.27802	-3.51872

Si	3.01583	-1.78588	-0.00310
C	1.54045	-1.99253	1.13079
H	0.65625	-1.49764	0.71086
H	1.29349	-3.05290	1.23418
H	1.74580	-1.60880	2.13492
C	4.48215	-2.74426	0.66490
H	5.34702	-2.69266	-0.00171
H	4.78580	-2.38036	1.64971
H	4.20856	-3.79897	0.76402
C	2.57978	-2.40655	-1.71078
H	1.79160	-1.79053	-2.15024
H	2.19650	-3.42754	-1.61936
H	3.43379	-2.43104	-2.38944
H	-0.56794	-3.79027	-1.00767
O	3.31473	-0.12616	0.06203
C	4.38537	0.63988	-0.52149
C	5.59320	0.59260	0.41156
H	5.98550	-0.42283	0.49143
H	6.38887	1.23951	0.03442
H	5.30933	0.93563	1.40911
C	3.86354	2.06867	-0.64348
H	3.67963	2.49964	0.34569
H	4.59656	2.70518	-1.14283
H	2.93749	2.08464	-1.22513
C	4.74619	0.10291	-1.90426
H	3.86398	0.08406	-2.54861
H	5.50231	0.74227	-2.36446
H	5.15882	-0.90751	-1.84032
K	1.25772	1.26676	1.09338
N	-2.24872	0.05326	0.27576
Si	-2.52409	-0.57122	1.90514

C	-1.14003	-0.00522	3.04784
H	-1.00243	1.07661	3.08420
H	-1.38736	-0.34082	4.05974
H	-0.19852	-0.49432	2.78332
C	-4.18580	0.01401	2.55608
H	-4.97685	-0.25594	1.85014
H	-4.41664	-0.45758	3.51553

Intermediate **75**

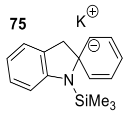

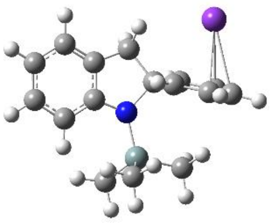

C	1.70972	-0.59325	-0.15837
C	1.22133	-1.90782	-0.28115
C	2.04199	-3.00289	-0.09981
C	3.39442	-2.81081	0.20692
C	3.88545	-1.51575	0.32621
C	3.06130	-0.40148	0.15438
C	-0.22647	-1.82360	-0.65402
H	1.64064	-4.00673	-0.19841
H	4.04987	-3.66072	0.35190
H	4.93097	-1.35906	0.56793
H	3.47997	0.58874	0.27470
H	-0.35200	-1.96957	-1.73438
C	-0.66059	-0.36782	-0.28080
C	-1.67493	0.17603	-1.24733
C	-1.20267	-0.27076	1.12305
C	-2.80578	0.85544	-0.86410
H	-1.42570	0.09530	-2.30229
C	-2.33304	0.43744	1.45167

H	-0.58464	-0.69950	1.90907
C	-3.19681	1.00835	0.48528
H	-3.43572	1.27412	-1.64674
H	-2.58905	0.52659	2.50577
H	-4.06365	1.59044	0.76692
N	0.70834	0.34923	-0.36423
Si	0.97747	2.06883	-0.05328
C	1.52481	2.33314	1.72896
H	0.68913	2.10733	2.39752
H	2.36388	1.69635	2.01668
H	1.81776	3.37485	1.89064
C	-0.54187	3.11239	-0.34371
H	-0.86657	3.07960	-1.38515
H	-1.38103	2.80841	0.28399
H	-0.27615	4.14589	-0.09660
C	2.29369	2.69206	-1.24707
H	1.93103	2.58641	-2.27347
H	2.47189	3.75642	-1.06495
H	3.25231	2.17673	-1.17898
H	-0.83777	-2.56824	-0.13642
K	-3.60008	-1.81451	0.02881

Intermediate **77**

77

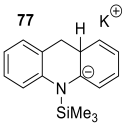

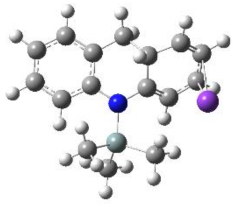

C	-4.12695	-1.76256	-0.14802
C	-2.90363	-2.42204	-0.18425
C	-1.68625	-1.75202	-0.07603
C	-1.68825	-0.33964	0.04633

C	-2.93243	0.31557	0.09989
C	-4.12932	-0.38036	0.00391
C	-0.39280	-2.53198	-0.11021
C	0.70919	-0.38874	0.29196
C	0.76344	-1.62636	-0.57983
C	2.13301	-2.26598	-0.48973
H	2.37445	-3.04624	-1.20543
C	2.94785	-2.05497	0.59864
C	2.71587	-1.03146	1.54728
C	1.55879	-0.21857	1.34702
H	-0.14051	-2.89547	0.89247
H	-5.05492	-2.31530	-0.22744
H	-2.88001	-3.50211	-0.29580
H	-2.97640	1.38783	0.23998
H	-5.06464	0.16531	0.05659
H	3.85127	-2.65620	0.69022
H	3.34863	-0.89877	2.41370
H	1.34851	0.58744	2.04864
H	-0.50042	-3.40925	-0.75845
H	0.52599	-1.34558	-1.61957
N	-0.48844	0.39098	0.12808
K	3.50894	0.34542	-0.86204
Si	-0.42771	2.15445	0.02519
C	1.32843	2.68783	-0.38279
H	2.04812	2.40407	0.38742
H	1.62826	2.29019	-1.35723
H	1.34043	3.77872	-0.46634
C	-0.92008	2.95922	1.64806
H	-0.93354	4.04841	1.54597
H	-1.91184	2.63872	1.97579
H	-0.20986	2.70113	2.43805

C	-1.51861	2.78585	-1.37843
H	-2.46703	3.19694	-1.02649
H	-0.99511	3.57994	-1.91809
H	-1.74380	1.98535	-2.08739

Substrate **68** and Me_3Si radical **24b** complex

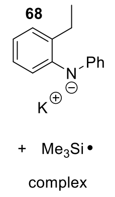

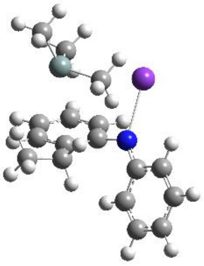

C	-0.16928	1.76466	-1.77078
C	0.32949	1.03763	-0.66535
C	-0.10795	1.44425	0.63572
C	-1.01273	2.49862	0.74117
C	-1.50551	3.18626	-0.37016
C	-1.07018	2.81606	-1.63531
H	0.21323	1.50507	-2.75494
H	-1.36404	2.79205	1.72407
H	-2.21046	3.99855	-0.24004
H	-1.41779	3.34702	-2.51510
C	0.37412	0.70396	1.86472
H	1.44053	0.90271	2.00675
H	0.32145	-0.37052	1.65738
C	2.30077	-0.28947	-0.35377
C	2.86762	-1.59066	-0.34989
C	3.11216	0.74593	0.17844
C	4.13396	-1.83558	0.15630
H	2.27433	-2.40544	-0.75523
C	4.37926	0.48701	0.68410
H	2.72991	1.76206	0.17284

C	4.91089	-0.80210	0.68542
H	4.52334	-2.84876	0.14189
H	4.96594	1.31054	1.07927
H	5.89986	-0.99595	1.08151
Si	-2.77205	-1.06181	0.86287
C	-3.28651	-0.14760	-0.70709
H	-4.17071	0.46312	-0.50836
H	-2.50447	0.53634	-1.05091
H	-3.55604	-0.84638	-1.50967
C	-1.37717	-2.28313	0.49540
H	-1.12095	-2.83684	1.40244
H	-1.69064	-3.02480	-0.25129
H	-0.46326	-1.77829	0.16205
C	-4.25328	-2.00757	1.54755
H	-4.60726	-2.73986	0.81330
H	-5.08115	-1.33358	1.77773
H	-3.98997	-2.54604	2.46043
N	1.07651	-0.09900	-0.91960
K	-0.67903	-1.21680	-2.46691
C	-0.35852	1.02093	3.16621
H	-1.43467	0.84790	3.07779
H	0.02071	0.38460	3.96823
H	-0.20608	2.05886	3.47205

Intermediate **79** and Me_3SiH radical

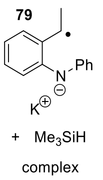

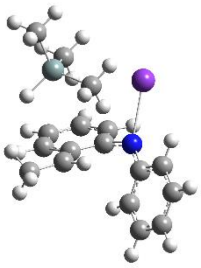

C	-0.21881	1.91119	-1.69509

C	0.35316	1.11811	-0.67346
C	-0.05949	1.40852	0.69480
C	-0.96252	2.48851	0.90512
C	-1.49126	3.23634	-0.13076
C	-1.11861	2.94196	-1.44686
H	0.12485	1.73156	-2.71078
H	-1.26444	2.71513	1.92174
H	-2.18347	4.04356	0.07744
H	-1.50706	3.52952	-2.27124
C	0.36764	0.62349	1.78219
H	0.99709	-0.23313	1.59021
H	-2.53644	-0.26280	2.04740
C	2.32241	-0.24465	-0.44793
C	2.85146	-1.55044	-0.57746
C	3.12493	0.69496	0.24678
C	4.08355	-1.89607	-0.03998
H	2.26261	-2.29044	-1.11174
C	4.35452	0.33815	0.78019
H	2.76229	1.71162	0.35483
C	4.85267	-0.95940	0.65002
H	4.44840	-2.91113	-0.16007
H	4.93939	1.08841	1.30280
H	5.81375	-1.22990	1.06937
Si	-2.79930	-1.14776	0.88712
C	-3.28448	-0.09215	-0.59453
H	-4.18765	0.48011	-0.36704
H	-2.50582	0.63401	-0.84842
H	-3.51757	-0.71277	-1.46724
C	-1.27220	-2.17762	0.49444
H	-0.95485	-2.72918	1.38357
H	-1.49541	-2.92504	-0.27621

H	-0.42118	-1.56184	0.18341
C	-4.22075	-2.30537	1.30705
H	-4.44881	-2.96106	0.46205
H	-5.12650	-1.74520	1.55144
H	-3.97425	-2.93681	2.16408
N	1.13220	0.05795	-1.05204
K	-0.73434	-1.02974	-2.49822
C	0.01976	0.92507	3.20504
H	-1.06358	0.92098	3.37481
H	0.46498	0.19203	3.87755
H	0.37907	1.91679	3.50550

Intermediate **79**

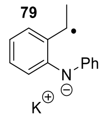

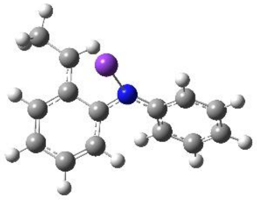

C	-0.33779	-1.73809	0.68800
C	-0.55116	-0.51894	-0.01488
C	-1.92406	-0.22451	-0.43842
C	-2.96141	-1.12767	-0.04376
C	-2.70125	-2.28151	0.65720
C	-1.37349	-2.58912	1.01866
H	0.66800	-1.97575	1.01288
H	-3.98031	-0.90408	-0.34079
H	-3.50835	-2.95382	0.92367
H	-1.16255	-3.49335	1.57902
C	-2.19710	0.89360	-1.23290
H	-1.35064	1.44768	-1.62269
C	1.73219	0.15212	-0.21598
C	2.29601	-1.03303	-0.74134

C	2.63738	1.13740	0.23466
C	3.67077	-1.21826	-0.78726
H	1.63640	-1.79720	-1.13620
C	4.01119	0.94312	0.19094
H	2.23453	2.07139	0.61460
C	4.54589	-0.24001	-0.31573
H	4.06606	-2.13767	-1.20621
H	4.67143	1.72446	0.55220
H	5.61754	-0.39194	-0.35296
N	0.38274	0.43733	-0.21292
K	-1.02157	2.15683	1.23282
C	-3.56991	1.24119	-1.72671
H	-3.96269	0.47635	-2.40999
H	-4.29918	1.32760	-0.91279
H	-3.56325	2.18760	-2.26875

Intermediate **80**

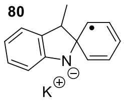

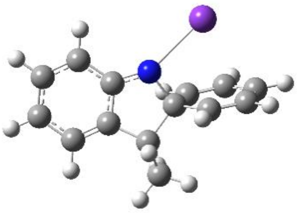

C	1.38254	0.74080	-0.21207
C	1.83758	-0.60680	-0.19176
C	3.15625	-0.93036	0.04067
C	4.09381	0.09315	0.26017
C	3.66708	1.42035	0.22001
C	2.33494	1.76125	-0.01038
C	0.66121	-1.47231	-0.57385
H	3.47363	-1.96974	0.04596
H	5.13220	-0.14482	0.45477
H	4.39009	2.21385	0.38226

H	2.02768	2.80184	-0.02688
H	0.69140	-1.61502	-1.66472
C	-0.52539	-0.49365	-0.28989
C	-1.66257	-0.71163	-1.24260
C	-0.98779	-0.56815	1.14111
C	-2.96155	-0.84073	-0.84789
H	-1.40444	-0.73073	-2.29732
C	-2.30335	-0.65909	1.50639
H	-0.21111	-0.45199	1.89136
C	-3.31834	-0.80189	0.52879
H	-3.74039	-0.97812	-1.59081
H	-2.57864	-0.62628	2.55560
H	-4.35473	-0.90292	0.82560
N	0.04849	0.88609	-0.42806
K	-2.15606	2.16915	-0.04526
C	0.55845	-2.83859	0.08981
H	-0.36860	-3.34231	-0.19930
H	1.39499	-3.47456	-0.20980
H	0.57629	-2.74989	1.17848

Intermediate **86**

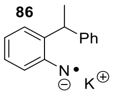

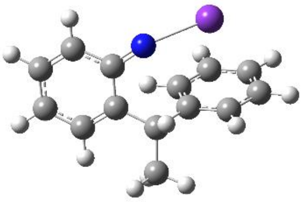

C	2.76418	1.63253	0.05884
C	1.58711	0.96825	-0.45084
C	1.59766	-0.49153	-0.33406
C	2.66953	-1.14124	0.25153
C	3.77428	-0.43642	0.75300
C	3.81230	0.95760	0.65252

H	2.78209	2.71348	-0.03579
H	2.67147	-2.22255	0.33764
H	4.59485	-0.97647	1.21075
H	4.66895	1.50358	1.03409
C	0.39993	-1.24190	-0.87927
H	0.25700	-0.89813	-1.90879
C	-0.88881	-0.90592	-0.14469
C	-2.11461	-1.03577	-0.80744
C	-0.90403	-0.53733	1.20340
C	-3.31907	-0.78188	-0.15536
H	-2.11991	-1.33044	-1.85274
C	-2.10576	-0.27873	1.86022
H	0.03733	-0.43148	1.73220
C	-3.31993	-0.39365	1.18428
H	-4.25579	-0.88841	-0.69098
H	-2.09402	0.01183	2.90470
H	-4.25386	-0.19733	1.69740
N	0.56561	1.63581	-0.94897
K	-1.85108	2.07585	-0.46278
C	0.56679	-2.76724	-0.90257
H	1.45815	-3.05425	-1.46422
H	0.64889	-3.17173	0.10975
H	-0.30140	-3.23056	-1.37575

Intermediate **81**

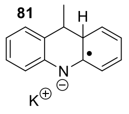

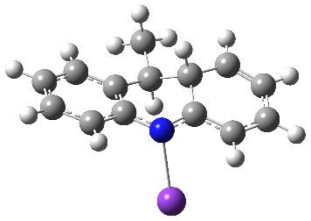

C	3.84365	0.45608	-0.03400
C	2.71998	1.17178	0.38381

C	1.42923	0.70320	0.16293
C	1.24596	-0.52665	-0.52605
C	2.38960	-1.24372	-0.93071
C	3.66746	-0.76065	-0.68913
C	0.17685	1.41683	0.63570
C	-1.08280	-0.29546	-0.66332
C	-0.91752	1.20107	-0.44286
C	-2.22349	1.89393	-0.16050
H	-2.19078	2.95514	0.05763
C	-3.42983	1.27066	-0.25884
C	-3.53376	-0.10419	-0.60211
C	-2.36473	-0.85159	-0.78725
H	-0.18158	0.91145	1.54698
H	4.83725	0.84615	0.15038
H	2.86657	2.11602	0.89541
H	2.24030	-2.18111	-1.45606
H	4.52883	-1.33024	-1.02086
H	-4.33672	1.84029	-0.08123
H	-4.50582	-0.56816	-0.71298
H	-2.42859	-1.89926	-1.07524
H	-0.48003	1.61955	-1.37131
N	0.00307	-1.08487	-0.75233
K	-0.82326	-2.62249	1.20054
C	0.39782	2.88824	0.97060
H	1.15587	3.01072	1.74580
H	0.72414	3.44034	0.08367
H	-0.51952	3.34332	1.34419

Intermediate **81** and KO^{t}Bu complex

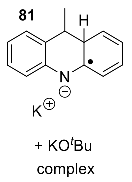

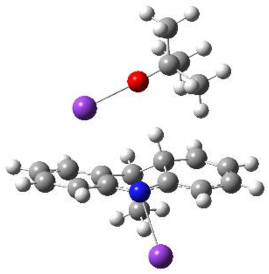

C	1.62312	3.59088	0.79806
C	1.22646	2.47438	1.54126
C	1.16221	1.20510	0.98042
C	1.49831	1.01277	-0.39254
C	1.91995	2.15048	-1.12390
C	1.98072	3.40910	-0.54063
C	0.79133	-0.01625	1.78305
C	0.81081	-1.22609	-0.41597
C	0.01810	-0.97606	0.85290
C	-0.42024	-2.24232	1.53180
H	-0.86453	-2.14294	2.51842
C	-0.34983	-3.45938	0.93192
C	0.21793	-3.60803	-0.36519
C	0.79969	-2.49974	-0.99558
H	1.66704	4.57107	1.25619
H	0.95986	2.59156	2.58848
H	2.17904	2.01329	-2.16865
H	2.30320	4.25751	-1.13484
H	-0.73435	-4.33503	1.44487
H	0.21569	-4.57466	-0.85315
H	1.21397	-2.60674	-1.99673
H	0.12289	0.27878	2.59951
H	-0.88337	-0.40874	0.53741
C	-3.61174	-0.16381	-0.32002
C	-4.95532	0.31602	-0.90771

H	-5.32369	1.16799	-0.32792
H	-4.80362	0.64555	-1.94016
H	-5.72135	-0.46758	-0.90115
C	-3.12790	-1.37322	-1.14868
H	-2.94746	-1.05514	-2.18043
H	-2.18856	-1.75866	-0.74062
H	-3.85889	-2.18995	-1.15948
C	-3.86148	-0.63720	1.12749
H	-2.92428	-1.00112	1.55891
H	-4.21394	0.20597	1.72962
H	-4.60314	-1.44194	1.18455
O	-2.68334	0.85061	-0.34522
K	-1.16874	2.62418	-0.55865
N	1.46521	-0.20648	-1.02044
K	3.69283	-1.59145	-1.23418
C	2.04136	-0.66395	2.38861
H	2.56174	0.03653	3.04547
H	1.77869	-1.55817	2.95856
H	2.73684	-0.96476	1.59817

Intermediate **87** and HO*t*Bu complex

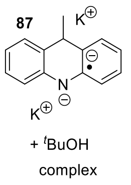

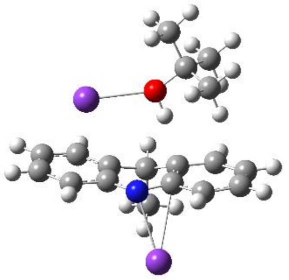

C	1.81578	3.45794	0.77543
C	1.28242	2.37591	1.49738
C	1.14316	1.10929	0.94812
C	1.53098	0.86158	-0.41357
C	2.13062	1.96093	-1.10732

C	2.26587	3.21456	-0.52661
C	0.68333	-0.06494	1.78022
C	0.59730	-1.29011	-0.43174
C	0.04877	-1.10380	0.88386
C	-0.61992	-2.23916	1.48825
H	-0.97186	-2.14960	2.51403
C	-0.85544	-3.40374	0.77986
C	-0.40734	-3.54471	-0.54579
C	0.34738	-2.47715	-1.12728
H	1.92431	4.43328	1.23306
H	0.97547	2.52169	2.53082
H	2.46080	1.78937	-2.12709
H	2.72114	4.01725	-1.09896
H	-1.39860	-4.21735	1.25329
H	-0.60301	-4.45037	-1.10733
H	0.64789	-2.52426	-2.17294
H	-0.05588	0.28997	2.51471
H	-1.41715	0.04915	0.29945
C	-3.34096	0.07649	-0.22600
C	-4.26363	1.14053	-0.80824
H	-4.37622	1.97113	-0.10599
H	-3.85564	1.52349	-1.74822
H	-5.25283	0.72370	-1.01081
C	-3.12260	-1.06451	-1.21648
H	-2.71083	-0.67606	-2.15218
H	-2.41692	-1.79340	-0.80685
H	-4.06631	-1.57226	-1.43389
C	-3.89471	-0.45677	1.09412
H	-3.19953	-1.18789	1.51557
H	-4.02351	0.36173	1.80699
H	-4.86140	-0.94364	0.94093

O	-2.09042	0.73089	0.01976
K	-0.78724	2.78741	-0.74589
N	1.36387	-0.31181	-1.06202
K	3.30479	-2.10332	-1.06778
C	1.87537	-0.63298	2.58384
H	2.28511	0.10559	3.27977
H	1.56271	-1.51782	3.14506
H	2.67657	-0.93188	1.89818

Intermediate **87**

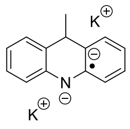

87

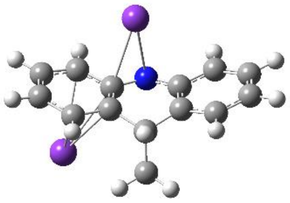

C	4.04193	-0.82799	0.36377
C	2.82946	-1.20083	0.93985
C	1.60534	-0.79607	0.41211
C	1.56317	0.04448	-0.73920
C	2.81034	0.39702	-1.32002
C	4.01473	-0.03164	-0.78519
C	0.29801	-1.31873	0.95727
C	-0.71113	0.48440	-0.46059
C	-0.78992	-0.30624	0.69666
C	-1.99347	-0.31680	1.50478
H	-2.01487	-0.90009	2.41956
C	-3.03285	0.59380	1.20013
C	-2.95723	1.40296	0.07651
C	-1.82082	1.33816	-0.80098
H	4.98182	-1.15771	0.78951
H	2.82607	-1.84164	1.81894
H	2.79038	1.02280	-2.20695

H	4.94428	0.26075	-1.26297
H	-3.90433	0.65252	1.84601
H	-3.76758	2.09011	-0.14680
H	-1.72944	1.98326	-1.66732
H	0.39559	-1.47775	2.03984
N	0.41892	0.58657	-1.25083
K	0.69079	2.58317	0.56868
K	-2.90157	-1.33679	-1.07209
C	-0.03901	-2.68649	0.32302
H	-1.01237	-3.04463	0.67747
H	-0.06224	-2.59140	-0.76862
H	0.71362	-3.43958	0.57316

Substrate **52** and Me_3Si radical **24b** complex

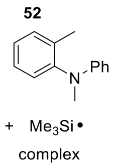

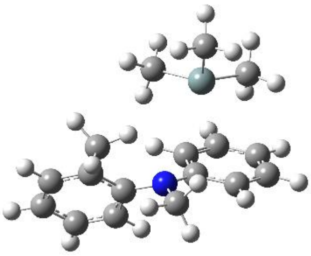

C	2.70584	1.20041	0.01968
C	1.90911	0.09882	0.34995
C	2.38324	-1.20255	0.12349
C	3.65141	-1.35374	-0.44562
C	4.43497	-0.26012	-0.78761
C	3.95734	1.02709	-0.55206
H	2.32404	2.19701	0.21088
H	4.02032	-2.35763	-0.63028
H	5.41065	-0.40946	-1.23436
H	4.56019	1.89144	-0.80400
C	1.58006	-2.42400	0.48926
H	0.52014	-2.18776	0.58755

H	1.69978	-3.19823	-0.27065
H	1.92137	-2.84286	1.44110
C	-0.23913	1.26982	0.45725
C	-0.20002	1.65378	-0.89743
C	-1.20856	1.86642	1.28062
C	-1.06949	2.61501	-1.38834
H	0.52396	1.19817	-1.56149
C	-2.07842	2.82703	0.77077
H	-1.28742	1.59293	2.32366
C	-2.01530	3.21892	-0.55980
H	-1.00975	2.89134	-2.43497
H	-2.81193	3.27204	1.43362
H	-2.69133	3.96993	-0.94875
N	0.62946	0.29463	0.94978
C	0.50987	-0.09348	2.34803
H	-0.48949	-0.49241	2.54399
H	1.23941	-0.87083	2.56315
H	0.70017	0.75134	3.02222
Si	-2.33693	-1.59271	-0.13911
C	-3.57077	-0.23226	-0.54443
H	-3.05210	0.68445	-0.83342
H	-4.21088	-0.54622	-1.37667
H	-4.21140	-0.00372	0.31042
C	-3.25163	-3.20824	0.20693
H	-2.55729	-4.00951	0.46982
H	-3.96457	-3.09310	1.02633
H	-3.80819	-3.52196	-0.68336
C	-1.13734	-1.83803	-1.57635
H	-0.68080	-2.82981	-1.53558
H	-1.66575	-1.74341	-2.53082
H	-0.33555	-1.09559	-1.55532

Intermediate **70** and Me_3SiH complex

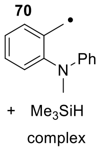

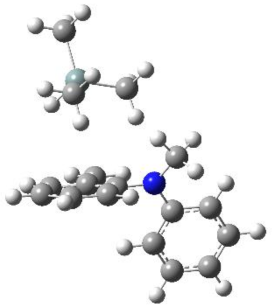

C	-0.04329	1.90556	1.34962
C	-0.28867	0.91521	0.40620
C	0.26168	1.02473	-0.90537
C	1.05366	2.16750	-1.18770
C	1.29403	3.13882	-0.23536
C	0.74563	3.01261	1.04371
H	-0.47750	1.80430	2.33839
H	1.48520	2.25823	-2.17871
H	1.90968	3.99599	-0.48045
H	0.92541	3.77179	1.79497
C	0.05091	0.04035	-1.89326
H	-0.53502	-0.84453	-1.69187
H	0.48800	0.16042	-2.87537
H	3.11119	-0.30572	-1.70202
C	-2.35481	-0.38788	0.28246
C	-3.13978	-1.47788	0.69546
C	-2.92270	0.53128	-0.61838
C	-4.43119	-1.64609	0.20743
H	-2.74895	-2.20134	1.39752
C	-4.21241	0.34677	-1.09675
H	-2.35537	1.39538	-0.93807
C	-4.98083	-0.74265	-0.69443
H	-5.01061	-2.49834	0.54394
H	-4.62243	1.07342	-1.78898

H	-5.98699	-0.87868	-1.07039
Si	3.35237	-1.01259	-0.41441
C	3.42592	0.23869	0.98382
H	4.22959	0.96207	0.82413
H	2.48791	0.79226	1.07082
H	3.61362	-0.26881	1.93481
C	1.97310	-2.25068	-0.10088
H	1.89167	-2.96597	-0.92353
H	2.17407	-2.81261	0.81635
H	1.00824	-1.75055	0.00804
C	4.99348	-1.92521	-0.53333
H	5.20508	-2.45463	0.39983
H	5.81816	-1.23336	-0.72107
H	4.98006	-2.66104	-1.34105
N	-1.05130	-0.23041	0.76271
C	-0.67262	-0.92694	1.98729
H	-1.35119	-0.69822	2.81759
H	-0.66582	-2.00781	1.82848
H	0.33930	-0.62799	2.25624

Intermediate **70**

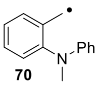

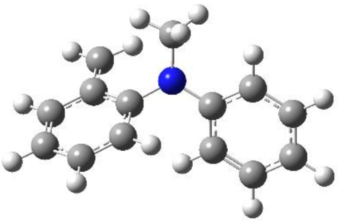

C	1.16616	-1.00831	-0.95868
C	1.07701	0.14502	-0.18855
C	2.19300	0.57757	0.58822
C	3.38746	-0.18786	0.50082
C	3.45866	-1.32964	-0.26979
C	2.34170	-1.75262	-0.99971

H	0.29954	-1.32084	-1.53118
H	4.24805	0.13627	1.07593
H	4.37898	-1.90006	-0.30818
H	2.39340	-2.64651	-1.60915
C	2.11741	1.69948	1.43380
H	1.19128	2.24213	1.55943
H	2.98222	2.00755	2.00597
C	-1.34959	0.29973	-0.01969
C	-1.45682	-0.83945	0.79936
C	-2.51661	0.80539	-0.60972
C	-2.68398	-1.44664	1.00753
H	-0.56826	-1.23613	1.27564
C	-3.74545	0.18824	-0.38380
H	-2.47705	1.67623	-1.24975
C	-3.84331	-0.93994	0.41875
H	-2.73770	-2.32138	1.64558
H	-4.63217	0.59969	-0.85252
H	-4.80076	-1.41664	0.58733
N	-0.11094	0.92814	-0.18377
C	-0.04997	2.16075	-0.95996
H	-0.30693	1.99388	-2.01406
H	-0.73153	2.90372	-0.54085
H	0.96352	2.55481	-0.90925

Intermediate **88**

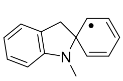

88

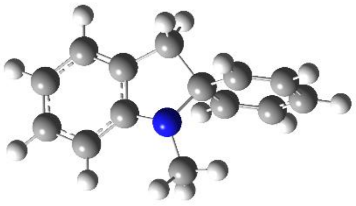

C	1.27727	0.49397	-0.23758
C	1.38202	-0.90491	-0.24066

C	2.58848	-1.51829	0.02632
C	3.71076	-0.72540	0.30542
C	3.59736	0.65964	0.31589
C	2.37812	1.29281	0.05178
C	0.02984	-1.46972	-0.59556
H	2.66747	-2.60014	0.02688
H	4.66327	-1.19302	0.52198
H	4.46675	1.26613	0.54270
H	2.30001	2.37298	0.07541
H	-0.25098	-2.35969	-0.03207
H	-0.01627	-1.70596	-1.66425
C	-0.92719	-0.27298	-0.29251
C	-2.10270	-0.23007	-1.21924
C	-1.34258	-0.29318	1.15464
C	-3.38917	-0.20130	-0.78133
H	-1.87231	-0.20894	-2.27944
C	-2.64239	-0.25319	1.55083
H	-0.53927	-0.32651	1.88535
C	-3.69500	-0.20828	0.60309
H	-4.19918	-0.16849	-1.50142
H	-2.88248	-0.25787	2.60841
H	-4.72491	-0.17633	0.93318
N	-0.02045	0.89051	-0.55324
C	-0.47575	2.20783	-0.15159
H	-1.52786	2.31292	-0.42043
H	-0.36645	2.37919	0.92775
H	0.09092	2.97058	-0.68844

Intermediate **89**

89

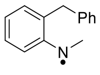

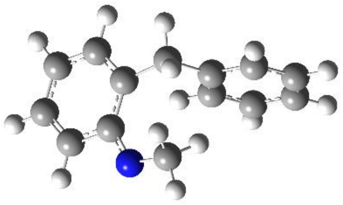

C	-2.90688	0.41332	-0.91768
C	-1.70069	0.79002	-0.24497
C	-1.16870	-0.13750	0.72464
C	-1.87489	-1.31019	0.96636
C	-3.06226	-1.63258	0.30932
C	-3.57668	-0.75877	-0.65041
H	-3.28102	1.11806	-1.65023
H	-1.47268	-2.01085	1.69070
H	-3.57105	-2.56063	0.53990
H	-4.49237	-0.99714	-1.17777
C	0.15384	0.01933	1.43872
H	0.24510	0.99633	1.91309
H	0.17672	-0.70882	2.25483
C	1.37800	-0.22084	0.56862
C	2.63013	0.19074	1.03481
C	1.30144	-0.85674	-0.66958
C	3.77834	-0.02837	0.28394
H	2.70065	0.69351	1.99458
C	2.45195	-1.07610	-1.42551
H	0.33969	-1.18023	-1.05228
C	3.69235	-0.66410	-0.95312
H	4.73993	0.30016	0.66091
H	2.37407	-1.56931	-2.38744
H	4.58523	-0.83251	-1.54327
N	-1.24395	1.99436	-0.63475
C	-0.12277	2.64918	-0.00884

H	-0.30434	2.83054	1.05750
H	0.80900	2.08065	-0.10255
H	0.01967	3.61525	-0.49252

Intermediate **90**

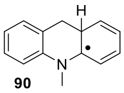

90

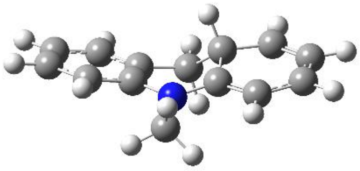

C	-3.65094	-0.92853	-0.12421
C	-2.46537	-1.58961	0.19101
C	-1.25987	-0.90659	0.27485
C	-1.23187	0.47738	0.02195
C	-2.41667	1.13832	-0.31981
C	-3.61631	0.43636	-0.38188
C	0.04732	-1.57688	0.58594
C	1.20074	0.52848	-0.05071
C	1.12624	-0.95548	-0.31601
C	2.46056	-1.63193	-0.20952
H	2.45897	-2.71667	-0.23059
C	3.62591	-0.93704	-0.16080
C	3.62720	0.48085	-0.13323
C	2.41160	1.18835	-0.04679
H	0.32785	-1.41894	1.63392
H	-4.58416	-1.47517	-0.17810
H	-2.47257	-2.65786	0.38152
H	-2.40666	2.19367	-0.55913
H	-4.52430	0.96450	-0.64798
H	4.57033	-1.46931	-0.13178
H	4.56208	1.02526	-0.12514
H	2.44941	2.26563	0.04142

H	-0.02090	-2.65348	0.41609
H	0.75323	-1.08039	-1.35126
N	-0.01053	1.16796	0.14111
C	-0.02068	2.60004	0.39297
H	0.14342	3.17862	-0.52203
H	-0.97365	2.88235	0.83261
H	0.76346	2.84367	1.11004

Intermediate **90** and KO*t*Bu complex

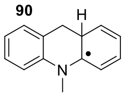

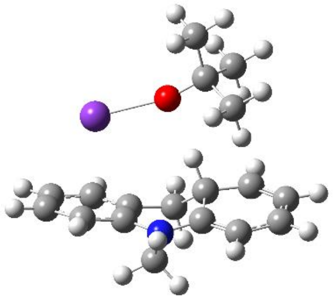

C	3.83782	0.83912	0.89386
C	2.80864	0.25902	1.63924
C	1.84232	-0.54374	1.04676
C	1.89183	-0.77661	-0.34687
C	2.90225	-0.15776	-1.10166
C	3.87144	0.62673	-0.48037
C	0.72513	-1.18122	1.82103
C	-0.27093	-1.89285	-0.33942
C	-0.54079	-1.16244	0.94870
C	-1.75349	-1.67582	1.66131
H	-1.93012	-1.29019	2.66026
C	-2.64006	-2.52454	1.07946
C	-2.41550	-3.02175	-0.22864
C	-1.21687	-2.71616	-0.90645
H	0.97957	-2.21749	2.07357
H	4.59411	1.44327	1.37928

H	2.75789	0.42138	2.71112
H	2.93378	-0.27811	-2.17625
H	4.65043	1.07781	-1.08411
H	-3.53027	-2.82904	1.61876
H	-3.14234	-3.67024	-0.69940
H	-1.05835	-3.15194	-1.88329
H	0.55309	-0.64285	2.75582
H	-0.71486	-0.09585	0.67655
C	-2.38912	1.96054	-0.29505
C	-2.83112	3.27803	-0.96395
H	-2.49634	4.12567	-0.35785
H	-2.36728	3.35885	-1.95190
H	-3.91806	3.34445	-1.08398
C	-2.88618	0.78647	-1.16262
H	-2.39651	0.82571	-2.14096
H	-2.62825	-0.16542	-0.68992
H	-3.97107	0.81156	-1.31443
C	-3.07167	1.86599	1.08473
H	-2.78437	0.92839	1.57005
H	-2.73748	2.69661	1.71420
H	-4.16458	1.89785	1.01530
O	-1.01801	1.92611	-0.15998
K	1.26310	2.44713	-0.11358
N	0.96068	-1.63633	-0.93407
C	1.26221	-2.25514	-2.21601
H	0.74823	-1.75231	-3.04110
H	2.33459	-2.23575	-2.38897
H	0.95322	-3.29982	-2.19064

Intermediate **91** and HO*t*Bu complex

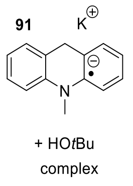

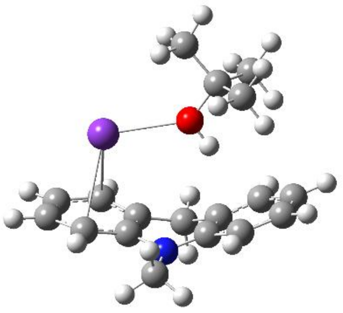

C	3.67428	1.31682	0.85852
C	2.64352	0.75312	1.65023
C	1.85725	-0.32019	1.08704
C	2.03148	-0.68894	-0.23611
C	2.94660	0.00218	-1.08151
C	3.82726	0.96421	-0.46578
C	0.91658	-1.11380	1.94982
C	-0.01829	-1.96511	-0.21424
C	-0.24715	-1.60939	1.13392
C	-1.52067	-1.76729	1.67257
H	-1.68295	-1.48233	2.70779
C	-2.57162	-2.29594	0.92256
C	-2.33515	-2.66624	-0.39648
C	-1.07751	-2.49287	-0.96839
H	1.43551	-1.97145	2.41003
H	4.35375	2.03956	1.30097
H	2.56779	0.96931	2.70890
H	3.09396	-0.28385	-2.11163
H	4.61978	1.40944	-1.05756
H	-3.55292	-2.42019	1.36442
H	-3.13754	-3.07514	-1.00008
H	-0.93202	-2.75290	-2.00834
H	0.55157	-0.49704	2.77865
H	-1.33689	0.28111	0.22083

C	-2.64974	1.69277	-0.25142
C	-2.49200	3.15080	-0.66073
H	-1.96365	3.70844	0.11699
H	-1.93107	3.22351	-1.59637
H	-3.46902	3.61356	-0.81138
C	-3.34925	0.88996	-1.34405
H	-2.79150	0.96175	-2.28060
H	-3.41139	-0.16302	-1.05669
H	-4.36236	1.26446	-1.50873
C	-3.39613	1.57482	1.07459
H	-3.47092	0.52585	1.37428
H	-2.86712	2.12445	1.85639
H	-4.40715	1.97859	0.98407
O	-1.30886	1.19923	-0.08612
K	1.09593	2.19877	-0.37889
N	1.24728	-1.75773	-0.75270
C	1.54560	-2.29017	-2.06752
H	1.12277	-1.68590	-2.88100
H	2.62532	-2.33875	-2.19087
H	1.15293	-3.30423	-2.14156

Intermediate **72**

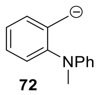

72

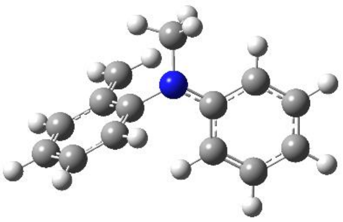

C	1.70981	-0.46451	-1.38487
C	1.13925	0.25461	-0.35113
C	1.77731	0.40877	0.93760
C	3.06412	-0.24527	1.02264
C	3.61137	-0.95808	-0.02069

C	2.95213	-1.09504	-1.25706
H	1.15042	-0.53507	-2.31464
H	3.60696	-0.16992	1.96104
H	4.58141	-1.42833	0.12121
H	3.38571	-1.65775	-2.07374
C	1.21198	1.11346	1.98670
H	0.23056	1.56239	1.90297
H	1.72362	1.17855	2.93993
C	-1.30364	0.21234	-0.24825
C	-1.28491	-1.13421	0.18244
C	-2.56251	0.84120	-0.36258
C	-2.46226	-1.80245	0.47145
H	-0.32917	-1.63083	0.29265
C	-3.73405	0.14929	-0.07022
H	-2.62818	1.87072	-0.68860
C	-3.70503	-1.17600	0.34813
H	-2.40871	-2.83303	0.80648
H	-4.68384	0.66395	-0.17329
H	-4.62061	-1.70762	0.57662
N	-0.13976	0.87405	-0.55866
C	-0.16963	2.31191	-0.75670
H	-0.65270	2.57666	-1.70520
H	-0.69888	2.82583	0.05482
H	0.85805	2.67012	-0.77434

Single-point energy calculation of intermediate **72** as a neutral species

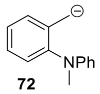

72

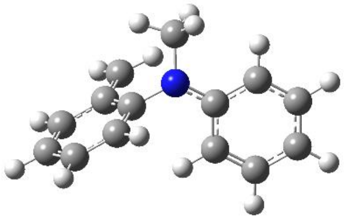

C	1.70981	-0.46451	-1.38487
C	1.13925	0.25461	-0.35113
C	1.77731	0.40877	0.93760
C	3.06412	-0.24527	1.02264
C	3.61137	-0.95808	-0.02069
C	2.95213	-1.09504	-1.25706
H	1.15042	-0.53507	-2.31464
H	3.60696	-0.16992	1.96104
H	4.58141	-1.42833	0.12121
H	3.38571	-1.65775	-2.07374
C	1.21198	1.11346	1.98670
H	0.23056	1.56239	1.90297
H	1.72362	1.17855	2.93993
C	-1.30364	0.21234	-0.24825
C	-1.28491	-1.13421	0.18244
C	-2.56251	0.84120	-0.36258
C	-2.46226	-1.80245	0.47145
H	-0.32917	-1.63083	0.29265
C	-3.73405	0.14929	-0.07022
H	-2.62818	1.87072	-0.68860
C	-3.70503	-1.17600	0.34813
H	-2.40871	-2.83303	0.80648
H	-4.68384	0.66395	-0.17329
H	-4.62061	-1.70762	0.57662
N	-0.13976	0.87405	-0.55866
C	-0.16963	2.31191	-0.75670

H	-0.65270	2.57666	-1.70520
H	-0.69888	2.82583	0.05482
H	0.85805	2.67012	-0.77434

Single-point energy calculation of intermediate **70** as an anion

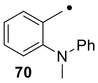

70

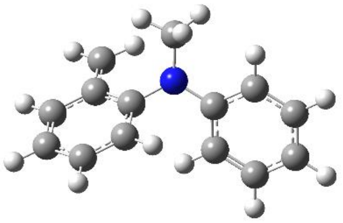

C	1.16616	-1.00831	-0.95868
C	1.07701	0.14502	-0.18855
C	2.19300	0.57757	0.58822
C	3.38746	-0.18786	0.50082
C	3.45866	-1.32964	-0.26979
C	2.34170	-1.75262	-0.99971
H	0.29954	-1.32084	-1.53118
H	4.24805	0.13627	1.07593
H	4.37898	-1.90006	-0.30818
H	2.39340	-2.64651	-1.60915
C	2.11741	1.69948	1.43380
H	1.19128	2.24213	1.55943
H	2.98222	2.00755	2.00597
C	-1.34959	0.29973	-0.01969
C	-1.45682	-0.83945	0.79936
C	-2.51661	0.80539	-0.60972
C	-2.68398	-1.44664	1.00753
H	-0.56826	-1.23613	1.27564
C	-3.74545	0.18824	-0.38380
H	-2.47705	1.67623	-1.24975
C	-3.84331	-0.93994	0.41875
H	-2.73770	-2.32138	1.64558

H	-4.63217	0.59969	-0.85252
H	-4.80076	-1.41664	0.58733
N	-0.11094	0.92814	-0.18377
C	-0.04997	2.16075	-0.95996
H	-0.30693	1.99388	-2.01406
H	-0.73153	2.90372	-0.54085
H	0.96352	2.55481	-0.90925

Intermediate **74**

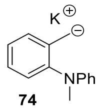

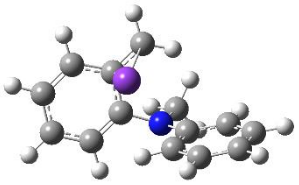

C	-1.71915	0.96573	-1.45403
C	-1.03542	0.91565	-0.25084
C	-1.57834	0.26610	0.91919
C	-2.88047	-0.31551	0.70750
C	-3.53812	-0.26177	-0.50677
C	-2.97412	0.37447	-1.62133
H	-1.23916	1.48119	-2.28120
H	-3.36222	-0.79896	1.55384
H	-4.52069	-0.71720	-0.58976
H	-3.49458	0.42311	-2.56852
C	-0.87559	0.10208	2.11411
H	0.06986	0.59799	2.28554
H	-1.39091	-0.29925	2.98045
C	1.38168	0.72868	-0.24801
C	2.58453	1.03768	0.41662
C	1.36437	-0.42941	-1.05701
C	3.69426	0.20848	0.29962
H	2.64873	1.91831	1.04154

C	2.48175	-1.24771	-1.16034
H	0.47732	-0.64088	-1.64422
C	3.65815	-0.94637	-0.47583
H	4.60085	0.47095	0.83367
H	2.44013	-2.11920	-1.80587
H	4.52828	-1.58487	-0.56074
N	0.26023	1.53385	-0.15586
K	-0.47854	-2.30976	0.52065
C	0.34431	2.78052	0.59027
H	1.14439	3.40479	0.18411
H	-0.60086	3.30701	0.46885
H	0.52244	2.63376	1.66211

Intermediate **76**

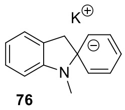

76

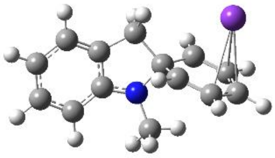

C	1.91921	0.46797	-0.21639
C	1.79530	-0.93129	-0.32081
C	2.86836	-1.75351	-0.05137
C	4.09390	-1.18507	0.33155
C	4.20859	0.19527	0.44399
C	3.12487	1.04169	0.18355
C	0.38452	-1.22789	-0.75463
H	2.76746	-2.83150	-0.12907
H	4.94381	-1.82102	0.54715
H	5.15385	0.62985	0.75022
H	3.22547	2.11510	0.29171
H	0.34548	-1.37034	-1.84182
C	-0.40005	0.06328	-0.38339

C	-1.54526	0.37582	-1.30027
C	-0.89594	0.04483	1.04049
C	-2.65820	1.06166	-0.86741
H	-1.38688	0.21139	-2.36271
C	-2.01169	0.74950	1.42809
H	-0.23283	-0.37719	1.79203
C	-2.92703	1.29973	0.49955
H	-3.38349	1.38546	-1.61168
H	-2.22731	0.83103	2.49207
H	-3.79352	1.86269	0.81939
N	0.72814	1.08422	-0.54625
H	-0.04127	-2.11273	-0.27631
K	-3.28069	-1.59832	0.20188
C	0.48229	2.44112	-0.10998
H	0.54317	2.54733	0.98211
H	1.20665	3.11827	-0.56915
H	-0.51773	2.73170	-0.43369

Intermediate **92**

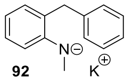

92

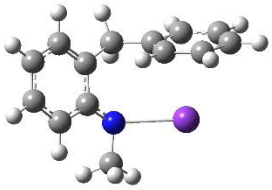

C	-1.71920	0.52591	0.19129
C	-1.46012	-0.84044	0.56764
C	-2.37805	-1.84062	0.29429
C	-3.59577	-1.58699	-0.34721
C	-3.87791	-0.27157	-0.70007
C	-2.98122	0.75820	-0.43884
C	-0.18739	-1.16683	1.29779
H	-2.13939	-2.85632	0.60066

H	-4.29646	-2.38757	-0.54810
H	-4.81877	-0.03449	-1.18816
H	-3.25630	1.76357	-0.73067
H	-0.25109	-2.18315	1.70145
H	-0.07390	-0.48806	2.15080
C	1.08383	-1.07146	0.47516
C	2.32516	-1.01779	1.11940
C	1.06076	-1.02297	-0.92102
C	3.50963	-0.89907	0.39527
H	2.35851	-1.06025	2.20403
C	2.24265	-0.90484	-1.65101
H	0.10251	-1.05611	-1.42852
C	3.47194	-0.83366	-0.99717
H	4.45994	-0.85967	0.91553
H	2.20313	-0.86429	-2.73367
H	4.38996	-0.74155	-1.56534
N	-0.79417	1.47170	0.44804
K	1.74277	1.83760	0.04780
C	-1.20736	2.82071	0.12410
H	-0.42311	3.53550	0.40898
H	-1.41458	2.98277	-0.94947
H	-2.11578	3.14174	0.66036

Intermediate **93**

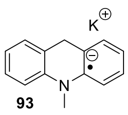

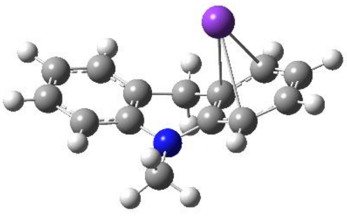

C	3.69494	-1.12071	0.35790
C	2.58860	-1.60522	-0.33948
C	1.51895	-0.77940	-0.66225

C	1.53594	0.56835	-0.24739
C	2.64757	1.05652	0.45161
C	3.71967	0.21491	0.74070
C	0.34407	-1.23827	-1.48549
C	-0.84652	0.71740	-0.53463
C	-0.91426	-0.59424	-0.96949
C	-2.16554	-1.31319	-0.92801
H	-2.22865	-2.30075	-1.36804
C	-3.33555	-0.59014	-0.57796
C	-3.26202	0.71337	-0.13530
C	-1.99354	1.38692	-0.01231
H	0.51248	-0.97173	-2.54099
H	4.52640	-1.77540	0.58880
H	2.56236	-2.64133	-0.66272
H	2.67757	2.08535	0.78570
H	4.57127	0.61116	1.28180
H	-4.30521	-1.07289	-0.65631
H	-4.16882	1.24323	0.13607
H	-1.95764	2.42491	0.28176
H	0.25925	-2.32868	-1.44762
N	0.42258	1.36060	-0.54115
K	-1.63665	-0.81514	1.88576
C	0.49296	2.78464	-0.28208
H	-0.31397	3.28052	-0.81795
H	0.41277	3.03235	0.78518
H	1.43888	3.17100	-0.66195

Substrate **52** and pentavalent silicate **25b** complex

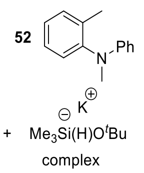

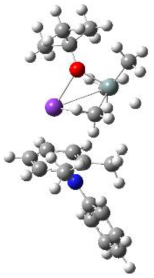

C	1.66366	2.21651	-1.09288
C	1.91367	1.06025	-0.34177
C	1.51944	1.00568	1.01297
C	0.90988	2.13624	1.56716
C	0.67395	3.28852	0.82145
C	1.04632	3.3238	-0.52077
H	1.98540	2.24794	-2.12787
H	0.60494	2.10032	2.60794
H	0.20502	4.14851	1.28443
H	0.87912	4.21464	-1.11484
C	1.74706	-0.21765	1.85747
H	2.74750	-0.20141	2.29957
H	1.01803	-0.26605	2.66669
H	1.67152	-1.13025	1.26397
C	3.78918	-0.49955	-0.48705
C	4.31219	-1.71870	-0.94553
C	4.55748	0.25789	0.41205
C	5.55149	-2.16578	-0.50208
H	3.75018	-2.32633	-1.64209
C	5.79043	-0.20523	0.85106
H	4.19143	1.21661	0.75936
C	6.30044	-1.42008	0.40155
H	5.92927	-3.11319	-0.86903
H	6.36236	0.40016	1.54493
H	7.26397	-1.77475	0.74505

Si	-2.21846	-1.55943	0.73599
C	-0.84854	-1.54840	-0.61232
H	0.05187	-1.02388	-0.26872
H	-0.53695	-2.58747	-0.75504
H	-1.15199	-1.15805	-1.58861
C	-3.41925	-3.03469	0.70046
H	-3.88055	-3.16653	1.68484
H	-4.20724	-2.97402	-0.05078
H	-2.82203	-3.93226	0.51359
C	-2.22912	-0.13899	2.04723
H	-2.78969	0.77089	1.80507
H	-1.19345	0.12970	2.28747
H	-2.64975	-0.54177	2.97459
H	-1.25151	-2.35450	1.71087
O	-3.26829	-0.47002	-0.46791
C	-4.61747	-0.06873	-0.45224
C	-5.34507	-0.72820	-1.63184
H	-5.32071	-1.81483	-1.52847
H	-6.38988	-0.40845	-1.69180
H	-4.84286	-0.46436	-2.56629
C	-4.66792	1.45771	-0.64273
H	-4.15798	1.73641	-1.57157
H	-5.69548	1.82574	-0.70470
H	-4.18815	1.96467	0.20256
C	-5.36232	-0.40779	0.85112
H	-4.86583	0.03539	1.71671
H	-6.38163	-0.01361	0.80739
H	-5.42574	-1.48494	1.00433
K	-1.44973	1.33485	-0.51928
N	2.54769	-0.04718	-0.94819
C	2.07756	-0.52086	-2.24534

H	2.85717	-0.42461	-3.00764
H	1.75941	-1.56497	-2.18859
H	1.21154	0.06380	-2.54802

Intermediate **74**, $Me_3SiOtBu$ **49** and H_2 complex

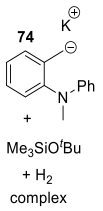

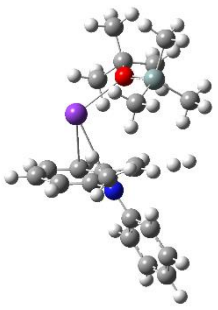

C	1.84593	2.27219	-1.27044
C	1.73122	1.15996	-0.45403
C	0.92943	1.19232	0.76304
C	0.44905	2.51725	1.10823
C	0.57851	3.60383	0.26920
C	1.23114	3.49974	-0.97330
H	2.43874	2.18183	-2.17692
H	-0.06776	2.63148	2.05649
H	0.16585	4.55941	0.57988
H	1.35376	4.35526	-1.62364
C	0.61091	0.07471	1.50195
H	1.04005	-0.89128	1.27549
H	0.02925	0.17523	2.41191
H	1.42188	-2.58681	-0.20311
C	3.46572	-0.52690	-0.11290
C	4.20473	-1.64278	-0.54683
C	3.88135	0.11398	1.07016
C	5.29860	-2.10106	0.18063
H	3.93140	-2.16415	-1.45391
C	4.97468	-0.35620	1.78314

H	3.34404	0.98293	1.42328
C	5.69551	-1.46812	1.35250
H	5.84564	-2.96346	-0.18397
H	5.26880	0.16135	2.68952
H	6.54852	-1.82730	1.91480
Si	-2.44784	-1.79123	-0.41383
C	-1.06158	-1.54746	-1.64840
H	-0.20989	-1.05984	-1.15812
H	-0.70355	-2.51630	-2.00681
H	-1.37284	-0.97562	-2.52849
C	-3.88258	-2.71933	-1.18403
H	-4.67485	-2.91616	-0.45657
H	-4.31628	-2.16350	-2.01926
H	-3.53810	-3.68626	-1.56227
C	-1.78887	-2.74184	1.05437
H	-1.07236	-2.13311	1.61076
H	-1.25893	-3.62357	0.68065
H	-2.57152	-3.08509	1.73275
H	1.38309	-3.32860	-0.23035
O	-2.91160	-0.20746	-0.06607
C	-3.90907	0.29861	0.83831
C	-5.24555	0.36351	0.10310
H	-5.57873	-0.63674	-0.18168
H	-6.01037	0.81341	0.74068
H	-5.14726	0.96747	-0.80187
C	-3.44441	1.69723	1.23623
H	-3.45625	2.36968	0.37279
H	-4.10835	2.12591	1.98945
H	-2.43203	1.65194	1.64891
C	-4.01840	-0.58130	2.08069
H	-3.04687	-0.67240	2.57204

H	-4.72716	-0.14016	2.78448
H	-4.38056	-1.58059	1.82479
K	-1.18724	1.54327	-1.12207
N	2.35918	-0.07234	-0.83532
C	2.26081	-0.49138	-2.22637
H	3.17037	-0.25935	-2.79574
H	2.07541	-1.56678	-2.29901
H	1.41968	0.02061	-2.68974

Intermediate **78**

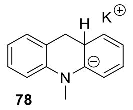

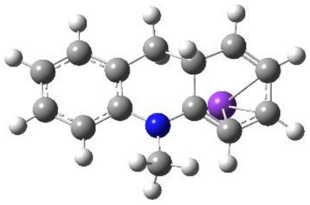

C	3.96326	-1.16294	-0.15535
C	2.71448	-1.68731	0.17325
C	1.57518	-0.89788	0.25770
C	1.67953	0.49983	0.04029
C	2.94027	1.02083	-0.31586
C	4.05832	0.19958	-0.40680
C	0.23809	-1.51578	0.58308
C	-0.72839	0.77778	0.36109
C	-0.73294	-0.48788	1.19477
C	-2.14786	-1.00833	1.34664
H	-2.27417	-1.91682	1.92834
C	-3.25531	-0.26413	1.00761
C	-3.18214	0.95606	0.30574
C	-1.86819	1.44016	0.00207
H	-0.20044	-1.93869	-0.33035
H	4.83322	-1.80444	-0.21879
H	2.61358	-2.75063	0.37107

H	3.05491	2.07199	-0.53897
H	5.01042	0.63955	-0.68182
H	-4.23485	-0.66380	1.26415
H	-4.05747	1.55387	0.09470
H	-1.77743	2.37214	-0.54925
H	0.37240	-2.35948	1.26843
H	-0.28296	-0.24525	2.18115
N	0.57061	1.32339	0.16778
K	-2.37906	-0.91319	-1.68783
C	0.72375	2.75180	-0.01773
H	1.62401	3.10236	0.48931
H	0.78685	3.03978	-1.07548
H	-0.12853	3.25545	0.43281

Intermediate **78** and Me_3Si radical **24b** complex

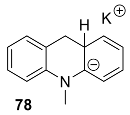

78

+ $Me_3Si^{\bullet}$
complex

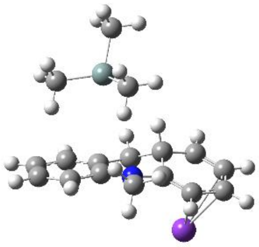

C	-2.14796	-2.84761	-1.16356
C	-1.33822	-1.83944	-1.68218
C	-0.44604	-1.12099	-0.89546
C	-0.37630	-1.39039	0.49434
C	-1.19644	-2.41336	1.01446
C	-2.06180	-3.12858	0.19413
C	0.44553	-0.07579	-1.52276
C	1.47804	0.17616	0.74960
C	1.01661	0.91476	-0.49035
C	2.14472	1.76698	-1.03757
H	1.93412	2.33534	-1.93899

C	3.29034	2.03275	-0.32371
C	3.61731	1.38309	0.88511
C	2.65651	0.44741	1.38630
H	1.26914	-0.57346	-2.05164
H	-2.82847	-3.39454	-1.80420
H	-1.39369	-1.59617	-2.73970
H	-1.14326	-2.67624	2.06164
H	-2.67110	-3.91221	0.63052
H	3.99323	2.75291	-0.73888
H	4.49473	1.64233	1.46041
H	2.88360	-0.08217	2.30788
H	-0.11601	0.47303	-2.28781
H	0.14362	1.53922	-0.20768
Si	-2.85859	1.55472	-0.19081
C	-4.05519	0.10238	-0.15648
H	-4.88231	0.31141	0.53145
H	-4.47562	-0.09738	-1.14480
H	-3.54230	-0.80175	0.18038
C	-3.76639	3.12826	-0.70939
H	-3.08938	3.98489	-0.73972
H	-4.21958	3.01841	-1.69717
H	-4.56540	3.35383	0.00585
C	-2.11265	1.79144	1.52464
H	-1.42684	2.64199	1.54688
H	-2.90440	1.96987	2.26093
H	-1.55227	0.90168	1.82239
N	0.48375	-0.67275	1.31156
C	0.48028	-0.92043	2.73785
H	-0.54595	-1.00167	3.10217
H	1.01854	-1.83663	3.01595
H	0.94578	-0.07376	3.23857

K	3.87041	-0.73556	-1.01862

Intermediate **93** and Me_3SiH complex

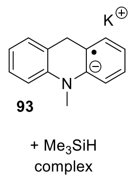

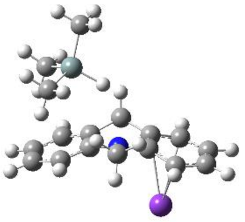

C	-1.26679	-3.31174	-0.33700
C	-0.54786	-2.48823	-1.20310
C	-0.07216	-1.24804	-0.79615
C	-0.27168	-0.83449	0.53871
C	-1.01049	-1.65352	1.40450
C	-1.50920	-2.87644	0.96102
C	0.60008	-0.28131	-1.73447
C	1.48076	0.80652	0.31702
C	1.68520	0.46837	-1.00878
C	2.91031	0.83902	-1.67476
H	3.01316	0.66124	-2.73818
C	3.81190	1.69230	-0.98386
C	3.60779	2.02027	0.33933
C	2.47551	1.50745	1.06626
H	1.01612	-0.81691	-2.59396
H	-1.64507	-4.26793	-0.67732
H	-0.38077	-2.79743	-2.23048
H	-1.17642	-1.35373	2.43150
H	-2.07545	-3.49624	1.64695
H	4.67727	2.09046	-1.50529
H	4.31440	2.66586	0.85008
H	2.29991	1.81298	2.08693
H	-0.15430	0.41624	-2.13361

H	-1.88895	1.54354	-0.16041
Si	-3.32027	1.14995	-0.25011
C	-3.49661	-0.36244	-1.34685
H	-4.55334	-0.58589	-1.52054
H	-3.01855	-0.20804	-2.31780
H	-3.03263	-1.23414	-0.87855
C	-4.29624	2.58828	-0.97151
H	-4.20731	3.48046	-0.34685
H	-3.94126	2.84207	-1.97336
H	-5.35690	2.33243	-1.04502
C	-3.97258	0.75828	1.47198
H	-3.81720	1.59320	2.16018
H	-5.04627	0.55332	1.42975
H	-3.47906	-0.12536	1.88474
N	0.29170	0.36798	0.95170
C	-0.10695	0.94150	2.21952
H	-1.18963	0.85399	2.32587
H	0.37419	0.46066	3.08170
H	0.14579	2.00034	2.22228
K	3.86517	-0.97320	0.38262

Intermediate **93**

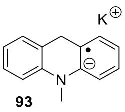

93

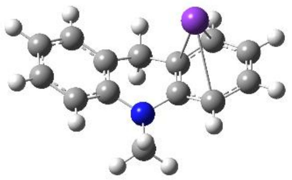

C	3.69494	-1.12071	0.35790
C	2.58860	-1.60522	-0.33948
C	1.51895	-0.77940	-0.66225
C	1.53594	0.56835	-0.24739
C	2.64757	1.05652	0.45161

C	3.71967	0.21491	0.74070
C	0.34407	-1.23827	-1.48549
C	-0.84652	0.71740	-0.53463
C	-0.91426	-0.59424	-0.96949
C	-2.16554	-1.31319	-0.92801
H	-2.22865	-2.30075	-1.36804
C	-3.33555	-0.59014	-0.57796
C	-3.26202	0.71337	-0.13530
C	-1.99354	1.38692	-0.01231
H	0.51248	-0.97173	-2.54099
H	4.52640	-1.77540	0.58880
H	2.56236	-2.64133	-0.66272
H	2.67757	2.08535	0.78570
H	4.57127	0.61116	1.28180
H	-4.30521	-1.07289	-0.65631
H	-4.16882	1.24323	0.13607
H	-1.95764	2.42491	0.28176
H	0.25925	-2.32868	-1.44762
N	0.42258	1.36060	-0.54115
K	-1.63665	-0.81514	1.88576
C	0.49296	2.78464	-0.28208
H	-0.31397	3.28052	-0.81795
H	0.41277	3.03235	0.78518
H	1.43888	3.17100	-0.66195

Transition States

Hydrogen atom abstraction from substrate **50b** by a Me_3Si radical (**TS8**)

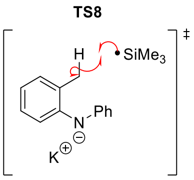

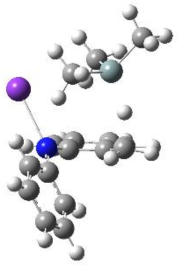

C	0.40427	2.41678	1.03264
C	-0.10692	1.38993	0.20869
C	0.36463	1.34918	-1.14756
C	1.28783	2.31765	-1.57428
C	1.77904	3.30879	-0.73290
C	1.33393	3.35167	0.58653
H	0.01529	2.48496	2.04550
H	1.64363	2.26491	-2.59945
H	2.49441	4.03468	-1.10037
H	1.68986	4.12277	1.26103
C	0.06998	0.22896	-2.02623
H	-0.83330	-0.34567	-1.85949
H	0.32542	0.36657	-3.07458
H	1.04351	-0.77512	-1.68917
C	-2.08800	0.03731	0.27383
C	-2.71859	-1.13271	0.77202
C	-2.81395	0.76641	-0.70453
C	-3.96132	-1.54656	0.31860
H	-2.20315	-1.70564	1.53765
C	-4.05854	0.34146	-1.14905
H	-2.38333	1.67823	-1.10436
C	-4.65175	-0.81925	-0.65284
H	-4.40100	-2.44954	0.73030

H	-4.57831	0.93300	-1.89628
H	-5.62274	-1.14332	-1.00588
Si	2.06969	-1.71748	-0.73691
C	3.06152	-0.44551	0.25617
H	3.89714	-0.08873	-0.35201
H	2.47172	0.44208	0.50791
H	3.49150	-0.88152	1.16602
C	0.90027	-2.68577	0.38817
H	0.52947	-3.57113	-0.13464
H	1.39508	-3.03567	1.30191
H	0.02711	-2.07547	0.64059
C	3.26718	-2.90538	-1.57604
H	3.86926	-3.43159	-0.82788
H	3.94850	-2.37347	-2.24357
H	2.73067	-3.65385	-2.16321
N	-0.88775	0.41412	0.79856
K	0.80371	-0.28886	2.62881

5-*exo*-trig cyclisation TS of intermediate **58b** (**TS9**)

TS9

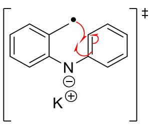

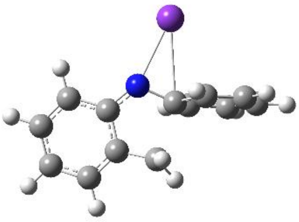

C	1.44698	0.44728	-0.28507
C	1.74565	-0.94993	-0.27651
C	3.00641	-1.40696	0.08016
C	4.03756	-0.50211	0.35105
C	3.77732	0.86744	0.25518
C	2.51189	1.34781	-0.05799
C	0.59315	-1.75510	-0.70394
H	3.20008	-2.47520	0.10607

H	5.02729	-0.85803	0.60883
H	4.57734	1.57582	0.44585
H	2.32138	2.41532	-0.09731
H	0.34848	-2.67774	-0.18351
H	0.39050	-1.77614	-1.77264
C	-0.75832	-0.26177	-0.26621
C	-1.84271	-0.43544	-1.19502
C	-1.07775	-0.61438	1.10401
C	-3.02500	-1.06118	-0.83623
H	-1.68240	-0.10084	-2.21525
C	-2.25609	-1.24519	1.43985
H	-0.32233	-0.41377	1.85840
C	-3.24486	-1.49660	0.47401
H	-3.79632	-1.20630	-1.58587
H	-2.43083	-1.53061	2.47239
H	-4.17165	-1.98521	0.74692
N	0.16012	0.80620	-0.49678
K	-1.79304	2.40980	0.13652

6-aryl cyclisation TS of intermediate **58b** (**TS11**)

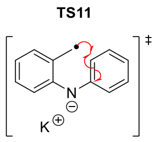

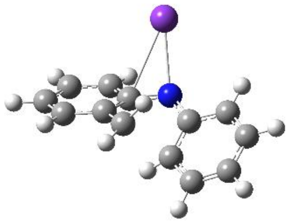

C	-3.27905	-1.71821	0.02375
C	-2.07958	-1.97134	0.69123
C	-0.95875	-1.15320	0.53856
C	-1.00915	-0.06794	-0.40369
C	-2.23749	0.17830	-1.04738
C	-3.35667	-0.62514	-0.83429
C	0.28906	-1.40494	1.24699

C	1.31067	0.29877	-0.47816
C	1.59701	-1.11915	-0.45724
C	2.92726	-1.55776	-0.25589
H	3.13595	-2.62146	-0.30884
C	3.94852	-0.66675	0.01793
C	3.67691	0.71289	-0.01846
C	2.39776	1.17875	-0.27982
H	0.75057	-0.59347	1.80012
H	-4.13597	-2.36268	0.17788
H	-2.01686	-2.80749	1.38160
H	-2.27610	0.98361	-1.77668
H	-4.27868	-0.41092	-1.36376
H	4.95440	-1.02109	0.20894
H	4.47955	1.42325	0.14871
H	2.19867	2.24586	-0.31549
H	0.44044	-2.39474	1.66787
H	0.91522	-1.78455	-0.97503
N	0.05185	0.79083	-0.60652
K	-1.25732	2.70012	0.58627

Ring-opening of intermediate **59b** (**TS10**)

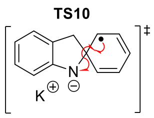

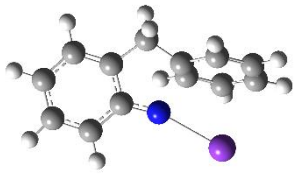

C	1.45416	0.55150	-0.18356
C	1.84645	-0.81774	-0.21702
C	3.14966	-1.20674	0.01555
C	4.14165	-0.24753	0.26045
C	3.78468	1.10125	0.26649
C	2.47087	1.51015	0.06804

C	0.68223	-1.68079	-0.61828
H	3.40797	-2.26184	-0.00869
H	5.16580	-0.54834	0.44204
H	4.54536	1.85351	0.45048
H	2.21167	2.56289	0.09585
H	0.70749	-2.66789	-0.14885
H	0.69176	-1.83332	-1.70502
C	-0.60673	-0.96552	-0.24070
C	-1.74122	-1.10083	-1.11267
C	-0.91512	-0.81070	1.15448
C	-3.03476	-0.94940	-0.65557
H	-1.55838	-1.31961	-2.15962
C	-2.21689	-0.64869	1.59931
H	-0.08903	-0.80161	1.85823
C	-3.29539	-0.70583	0.70639
H	-3.86224	-1.05263	-1.34993
H	-2.40297	-0.50546	2.65838
H	-4.31390	-0.61659	1.06432
N	0.16123	0.84128	-0.40929
K	-2.10262	1.95406	-0.24156

Deprotonation of intermediate **61b** by KO^{t}Bu (**TS12**)

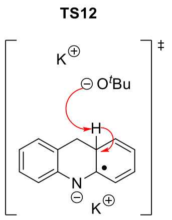

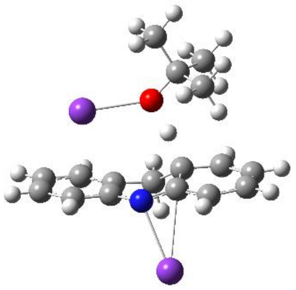

C	-1.88301	-3.41690	0.91282
C	-1.31477	-2.34953	1.62514
C	-1.19246	-1.08007	1.08148
C	-1.62933	-0.82247	-0.25978

C	-2.23704	-1.90853	-0.95101
C	-2.36058	-3.16756	-0.37718
C	-0.63901	0.08818	1.85298
C	-0.68241	1.32608	-0.31710
C	0.09744	1.03363	0.90351
C	0.69537	2.21709	1.53628
H	1.13522	2.09242	2.52346
C	0.78343	3.43217	0.90261
C	0.20647	3.62001	-0.37439
C	-0.54239	2.56311	-0.94536
H	-1.47081	0.61566	2.35391
H	-1.97779	-4.39661	1.36427
H	-0.96046	-2.51135	2.64012
H	-2.58880	-1.72827	-1.96185
H	-2.82675	-3.96666	-0.94497
H	1.29780	4.25746	1.38592
H	0.30321	4.56624	-0.89285
H	-1.00032	2.68948	-1.92571
H	0.03032	-0.26084	2.64664
H	1.09522	0.22029	0.46340
C	3.17345	-0.08722	-0.27869
C	4.03828	-1.15589	-0.95908
H	4.18714	-2.00258	-0.28168
H	3.54289	-1.51482	-1.86749
H	5.02012	-0.76368	-1.24183
C	2.94753	1.08016	-1.25042
H	2.42602	0.72172	-2.14329
H	2.32707	1.84854	-0.78147
H	3.89557	1.53298	-1.55802
C	3.89277	0.42450	0.97727
H	3.27626	1.18306	1.46637

H	4.04824	-0.40174	1.67686
H	4.86425	0.86723	0.73607
O	1.94667	-0.66959	0.07371
K	0.74422	-2.70998	-0.65710
N	-1.50946	0.38007	-0.88358
K	-3.46812	2.12404	-0.52905

Deprotonation of intermediate **61b** by pentavalent silicate **25b**

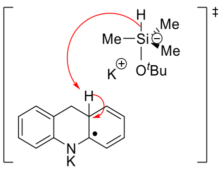

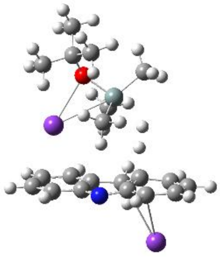

C	0.40660	3.89656	0.80898
C	0.91739	2.76689	1.46426
C	1.47595	1.69863	0.77570
C	1.53346	1.70872	-0.65482
C	1.00423	2.86597	-1.30194
C	0.47852	3.93335	-0.58904
C	2.14953	0.55435	1.49235
C	2.38772	-0.46107	-0.81037
C	2.19556	-0.70027	0.63178
C	2.96022	-1.82064	1.18730
H	3.00226	-1.92280	2.26878
C	3.44678	-2.82884	0.37635
C	3.37462	-2.71019	-1.02625
C	2.87165	-1.51313	-1.59618
H	3.17431	0.87307	1.76466
H	0.00139	4.72822	1.37189
H	0.89381	2.72385	2.55098
H	1.05513	2.89618	-2.38516

H	0.11338	4.80319	-1.12648
H	3.90260	-3.70835	0.82116
H	3.73159	-3.50977	-1.66430
H	2.89350	-1.36317	-2.67125
H	1.65289	0.34550	2.44806
H	0.87882	-1.27715	0.73274
Si	-1.79378	-1.22415	0.59868
C	-1.34892	-0.15133	2.10524
H	-1.08790	-0.82229	2.92708
H	-0.47111	0.47451	1.93492
H	-2.18878	0.47149	2.42782
C	-1.05134	-0.89637	-1.12645
H	-0.76419	-1.85382	-1.57079
H	-1.75072	-0.40884	-1.81472
H	-0.13028	-0.30937	-1.08494
C	-2.30994	-2.98367	1.04635
H	-3.36413	-3.02875	1.33424
H	-1.70581	-3.34273	1.88097
H	-2.15136	-3.66543	0.20661
H	0.03239	-1.80802	0.87795
O	-3.32059	-0.36340	0.35039
C	-4.45949	-0.63231	-0.45592
C	-4.68804	0.59223	-1.34756
H	-3.84905	0.72310	-2.03843
H	-5.59829	0.48800	-1.94308
H	-4.78860	1.48907	-0.72870
C	-5.65330	-0.81568	0.48336
H	-5.77747	0.07485	1.10395
H	-6.57677	-0.98729	-0.07645
H	-5.47925	-1.67064	1.14088
C	-4.31039	-1.87858	-1.33816

H	-4.22401	-2.78258	-0.73442
H	-5.19446	-1.98262	-1.97223
H	-3.43576	-1.80877	-1.98762
K	-1.73627	1.88840	-0.08457
N	2.06185	0.72079	-1.41444
K	5.21868	-0.44559	-0.23267

Deprotonation of the *o*-tolyl ring of substrate **50b** by pentavalent silicate **25b**

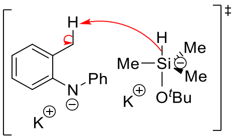

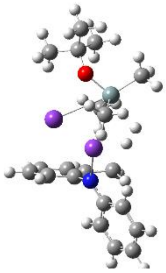

C	-1.61944	1.95580	1.45382
C	-1.81174	1.02590	0.42645
C	-1.27943	1.31723	-0.89128
C	-0.68048	2.59471	-1.05926
C	-0.55383	3.52203	-0.02494
C	-0.99843	3.20140	1.25704
H	-2.03467	1.70893	2.42732
H	-0.33157	2.86541	-2.05223
H	-0.11387	4.49351	-0.22661
H	-0.92220	3.91263	2.07142
C	-1.31586	0.35348	-1.95880
H	-2.13034	-0.36340	-1.91851
H	-1.18869	0.78609	-2.95241
H	-0.16080	-0.69114	-2.07182
C	-3.67049	-0.39977	0.11393
C	-4.28980	-1.67790	0.22453
C	-4.42256	0.57870	-0.59754
C	-5.54263	-1.94480	-0.30460

H	-3.75613	-2.48189	0.72569
C	-5.67400	0.29614	-1.12479
H	-4.00047	1.56935	-0.72438
C	-6.26152	-0.96243	-0.98785
H	-5.96274	-2.93936	-0.18951
H	-6.20491	1.07949	-1.65738
H	-7.23870	-1.17260	-1.40405
Si	2.21161	-1.01928	-0.95593
C	0.82205	-1.42366	0.27366
H	-0.09019	-0.84492	0.09704
H	0.56400	-2.47609	0.11720
H	1.18179	-1.30717	1.30323
C	2.91658	-2.55142	-1.79043
H	3.35659	-2.30616	-2.76017
H	3.68969	-3.01392	-1.16994
H	2.12580	-3.28266	-1.96282
C	2.15805	0.57485	-1.98359
H	2.81504	1.35793	-1.58819
H	1.14460	0.96642	-2.07661
H	2.50019	0.34546	-2.99688
H	0.51748	-1.31656	-2.22066
O	3.33753	-0.54640	0.28203
C	4.74041	-0.28947	0.25929
C	5.41232	-1.34840	1.13351
H	5.23499	-2.34278	0.71713
H	6.49115	-1.18337	1.19359
H	4.99524	-1.31759	2.14269
C	4.95134	1.09985	0.86349
H	4.50516	1.14358	1.86115
H	6.01428	1.33546	0.95426
H	4.49073	1.86415	0.22983

C	5.33836	-0.33037	-1.15081
H	4.85387	0.39110	-1.81167
H	6.40124	-0.08203	-1.10260
H	5.24776	-1.32484	-1.59042
K	1.40352	1.47536	0.87195
N	-2.46337	-0.18141	0.69843
K	-1.49442	-1.94975	2.29031

5-*exo*-trig cyclisation TS of intermediate **64**

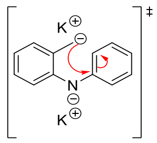

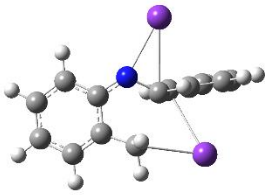

C	0.97340	0.47197	0.17769
C	1.38654	-0.90832	0.41932
C	2.56106	-1.13511	1.13976
C	3.42481	-0.07832	1.50696
C	3.09331	1.22593	1.15661
C	1.87219	1.50445	0.51265
C	0.46979	-1.86331	-0.14971
H	2.86185	-2.16044	1.33948
H	4.34137	-0.28671	2.04742
H	3.75456	2.04467	1.42209
H	1.58196	2.53312	0.32036
H	0.49310	-2.88818	0.21974
H	0.30176	-1.77784	-1.22200
C	-1.17314	-0.40060	-0.12072
C	-2.09064	-0.74285	-1.17847
C	-1.77720	-0.39473	1.19385
C	-3.39066	-1.14977	-0.93455
H	-1.71966	-0.69011	-2.19866

C	-3.07797	-0.80105	1.41753
H	-1.15421	-0.07343	2.02469
C	-3.92059	-1.19491	0.36339
H	-4.02145	-1.41853	-1.77785
H	-3.46433	-0.79222	2.43357
H	-4.94304	-1.50058	0.54395
N	-0.23059	0.65610	-0.39736
K	-2.55072	2.05040	-0.28962
K	3.26003	-0.19686	-1.57339

6-aryl cyclisation TS of intermediate **64**

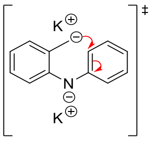

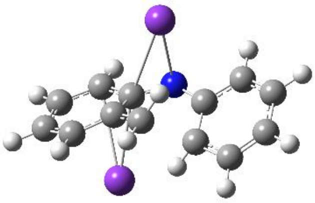

C	-3.36488	0.31499	0.90390
C	-2.18137	0.01519	1.57696
C	-0.90061	0.22968	1.02294
C	-0.84578	0.53858	-0.41471
C	-2.05640	0.98807	-1.01534
C	-3.29046	0.87522	-0.38710
C	0.30865	-0.08633	1.73965
C	1.37170	-0.20993	-0.68146
C	1.21175	-1.25210	0.33427
C	2.40442	-1.93330	0.74637
H	2.31107	-2.78504	1.41380
C	3.66366	-1.47753	0.39530
C	3.81528	-0.42534	-0.52256
C	2.66411	0.15822	-1.06729
H	1.16366	0.58125	1.71691
H	-4.32080	0.18981	1.39929
H	-2.23239	-0.33475	2.60580

H	-1.99326	1.34392	-2.04073
H	-4.19057	1.20216	-0.89657
H	4.54228	-1.97563	0.79436
H	4.79918	-0.10984	-0.84890
H	2.75304	0.93195	-1.82925
H	0.16165	-0.50218	2.73819
H	0.31549	-1.87020	0.25034
N	0.28668	0.50046	-1.16538
K	1.09266	2.65554	0.13473
K	-2.25668	-1.94959	-0.81533

Hydrogen atom abstraction from **67** by a Me_3Si radical (**TS13**)

TS13

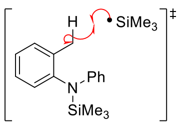

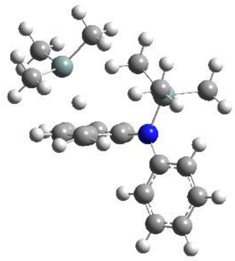

C	-0.86902	2.15704	1.55930
C	-0.59076	1.26386	0.53165
C	0.35158	1.59300	-0.46773
C	1.01251	2.83032	-0.36086
C	0.74214	3.70974	0.67784
C	-0.20578	3.37890	1.64464
H	-1.61155	1.87525	2.29866
H	1.74555	3.09559	-1.11620
H	1.26668	4.65664	0.73242
H	-0.42673	4.06289	2.45498
C	0.68557	0.66267	-1.53338
H	-0.01972	-0.13716	-1.73793
H	1.82118	-0.10769	-1.08085
C	-2.38186	-0.12438	-0.32522

C	-3.07958	-1.34122	-0.43128
C	-2.85791	0.96436	-1.07756
C	-4.19476	-1.45896	-1.25024
H	-2.75874	-2.20723	0.13186
C	-3.97519	0.83253	-1.89359
H	-2.35158	1.91915	-1.02046
C	-4.65647	-0.37598	-1.99225
H	-4.70725	-2.41285	-1.30431
H	-4.31400	1.69352	-2.45889
H	-5.52644	-0.47208	-2.62957
Si	3.41209	-0.64777	-0.85143
C	4.27360	0.71976	0.11332
H	4.16241	1.68097	-0.39420
H	3.86720	0.82293	1.12209
H	5.34352	0.50422	0.19981
C	3.57731	-2.29914	0.03617
H	3.11580	-3.10583	-0.53767
H	4.63702	-2.54039	0.16980
H	3.10810	-2.27147	1.02200
C	4.12871	-0.76188	-2.58726
H	3.62664	-1.53658	-3.17143
H	5.19473	-1.00600	-2.54744
H	4.02038	0.18733	-3.11776
N	-1.25538	-0.00660	0.50152
Si	-0.50401	-1.33492	1.41703
C	1.00234	-0.60513	2.25602
H	1.54275	-1.39758	2.78216
H	1.68171	-0.14014	1.53723
H	0.71977	0.15736	2.98571
C	0.03543	-2.71841	0.26777
H	-0.79752	-3.25966	-0.18383

H	0.65724	-2.31762	-0.53738
H	0.63930	-3.43966	0.82701
C	-1.66643	-1.97497	2.74317
H	-1.96664	-1.15413	3.40016
H	-2.57206	-2.43913	2.34983
H	-1.14680	-2.71979	3.35335
H	1.13486	1.10018	-2.42138

5-*exo*-trig cyclisation TS of intermediate **69** (**TS14**)

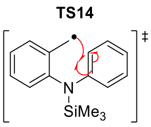

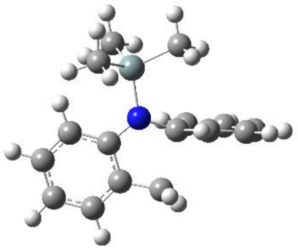

C	1.38007	-0.21455	-0.17977
C	1.47568	-1.60558	-0.38639
C	2.68575	-2.25820	-0.18419
C	3.82658	-1.54196	0.17821
C	3.73918	-0.16475	0.34042
C	2.52689	0.50251	0.17367
C	0.22648	-2.24394	-0.83389
H	2.73844	-3.33128	-0.33355
H	4.76926	-2.05407	0.32584
H	4.61817	0.40482	0.61940
H	2.48961	1.57047	0.34128
H	0.00656	-2.19829	-1.89688
C	-0.98009	-0.62792	-0.16218
C	-2.02212	-0.68310	-1.12937
C	-1.29991	-1.03468	1.16780
C	-3.21791	-1.30892	-0.83977
H	-1.82944	-0.26910	-2.11286

C	-2.50038	-1.66105	1.44213
H	-0.55429	-0.88665	1.94245
C	-3.46123	-1.82381	0.43878
H	-3.98084	-1.38513	-1.60593
H	-2.70740	-2.00667	2.44860
H	-4.40395	-2.30855	0.66044
N	0.09722	0.33319	-0.33742
Si	-0.27387	2.04834	-0.02047
C	-2.10101	2.33286	-0.30221
H	-2.39489	2.15491	-1.33854
H	-2.71754	1.70062	0.34122
H	-2.32042	3.37826	-0.06337
C	0.10608	2.47709	1.76839
H	-0.07423	3.54171	1.94382
H	-0.55798	1.91350	2.43005
H	1.13471	2.25646	2.05833
C	0.69127	3.11992	-1.21757
H	0.47991	2.80955	-2.24415
H	0.36491	4.15836	-1.10806
H	1.77193	3.09489	-1.07434
H	-0.13081	-3.13647	-0.32958

Ring-opening of intermediate **82** (**TS15**)

TS15

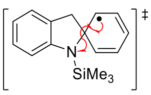

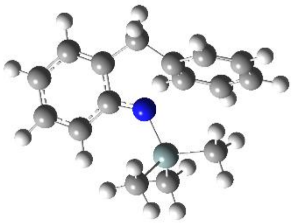

C	1.45395	0.06726	-0.23891
C	1.80843	-1.28903	-0.39481
C	3.08807	-1.73253	-0.12664
C	4.06495	-0.82613	0.29949

C	3.72680	0.51180	0.46539
C	2.43076	0.96498	0.22167
C	0.63937	-2.10284	-0.86999
H	3.33047	-2.78395	-0.24268
H	5.07045	-1.16734	0.51224
H	4.47527	1.21875	0.80509
H	2.19470	2.01069	0.37064
H	0.69856	-3.14120	-0.53553
C	-0.64218	-1.46816	-0.34724
C	-1.81922	-1.58011	-1.15889
C	-0.85064	-1.41480	1.07105
C	-3.07638	-1.58285	-0.60448
H	-1.69246	-1.67324	-2.23209
C	-2.12201	-1.38679	1.61215
H	0.02277	-1.37802	1.71490
C	-3.24431	-1.48216	0.78641
H	-3.94681	-1.66788	-1.24494
H	-2.24565	-1.31583	2.68668
H	-4.23903	-1.48226	1.21518
N	0.14921	0.35820	-0.58403
Si	-0.71103	1.82882	-0.11314
C	-2.50434	1.71441	-0.62879
H	-3.08941	1.07487	0.03219
H	-2.93266	2.72084	-0.59442
H	-2.60334	1.33748	-1.64928
C	0.05456	3.24428	-1.09079
H	0.03859	3.01284	-2.15889
H	-0.54377	4.14706	-0.93346
H	1.08331	3.47394	-0.81081
C	-0.60111	2.17399	1.73106
H	0.43167	2.16282	2.08796

H	-1.02691	3.15479	1.96222
H	-1.16032	1.42121	2.29253
H	0.60428	-2.10434	-1.96525

6-aryl cyclisation TS of intermediate **69** (**TS16**)

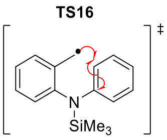

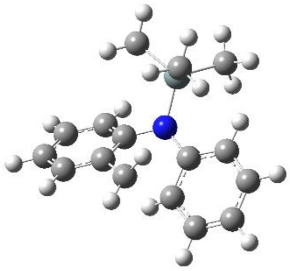

C	-3.59873	-1.74850	-0.03484
C	-2.48827	-2.11021	0.71905
C	-1.28952	-1.39132	0.63803
C	-1.21198	-0.31382	-0.27667
C	-2.31394	0.01323	-1.06003
C	-3.51233	-0.68299	-0.92704
C	-0.06849	-1.82794	1.30175
C	1.19100	-0.35585	-0.43814
C	1.09706	-1.79306	-0.45790
C	2.29153	-2.55812	-0.40168
H	2.21495	-3.63509	-0.49787
C	3.52121	-1.96019	-0.21285
C	3.60176	-0.56442	-0.18590
C	2.45464	0.22029	-0.30708
H	0.52531	-1.09996	1.84429
H	-4.52251	-2.30649	0.06107
H	-2.54377	-2.95529	1.39698
H	-2.22074	0.80915	-1.78934
H	-4.36436	-0.40883	-1.53733
H	4.41897	-2.56082	-0.13140
H	4.56335	-0.07663	-0.08004

H	2.56650	1.29580	-0.28764
H	-0.06153	-2.83046	1.71840
H	0.24858	-2.24551	-0.95683
N	0.01556	0.40838	-0.40593
Si	0.03679	2.11360	0.16555
C	1.04344	3.17753	-1.01448
H	0.51816	4.11905	-1.19459
H	2.03296	3.42228	-0.62222
H	1.17375	2.67657	-1.97677
C	-1.70980	2.77755	0.23977
H	-2.15679	2.91400	-0.74636
H	-2.37197	2.14772	0.83659
H	-1.65399	3.76122	0.71755
C	0.73033	2.13382	1.90855
H	1.71748	1.67271	1.97603
H	0.80906	3.16245	2.27203
H	0.05517	1.59296	2.57817

Deprotonation of intermediate **84** by KO*t*Bu (**TS17**)

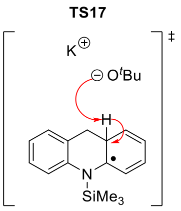

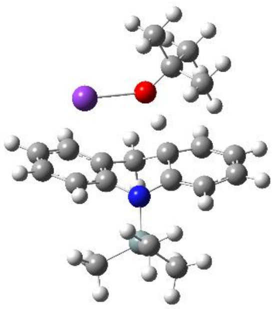

C	0.52979	3.67732	1.10412
C	0.05906	2.56942	1.81526
C	0.33874	1.27010	1.40518
C	1.09821	1.06408	0.23351
C	1.52153	2.17737	-0.50794
C	1.26286	3.47229	-0.06257

C	-0.10774	0.04523	2.15126
C	0.54219	-1.28950	0.14366
C	-0.50923	-1.03093	1.13988
C	-1.08454	-2.26351	1.70064
H	-1.69190	-2.17423	2.59723
C	-0.94631	-3.47792	1.08080
C	-0.14906	-3.60851	-0.07740
C	0.61794	-2.49739	-0.50627
H	0.71990	-0.30910	2.78541
H	0.32745	4.68100	1.45822
H	-0.52108	2.71425	2.72123
H	2.06504	2.02117	-1.43330
H	1.62678	4.31759	-0.63566
H	-1.44921	-4.35011	1.48637
H	-0.06613	-4.55681	-0.59236
H	1.30490	-2.61957	-1.33731
H	-0.94584	0.28680	2.81055
H	-1.48633	-0.41788	0.48319
C	-3.49025	-0.39933	-0.60491
C	-4.36774	0.53097	-1.45449
H	-4.70519	1.38090	-0.85232
H	-3.79818	0.90635	-2.31201
H	-5.25128	0.01477	-1.84174
C	-3.00113	-1.56573	-1.47446
H	-2.41521	-1.18089	-2.31479
H	-2.35972	-2.22855	-0.88843
H	-3.83796	-2.14862	-1.87214
C	-4.32147	-0.94419	0.56483
H	-3.70325	-1.60621	1.17600
H	-4.66842	-0.11676	1.19044
H	-5.19239	-1.50686	0.21484

O	-2.39860	0.33758	-0.12174
K	-1.60369	2.54194	-0.84454
N	1.45242	-0.23806	-0.15657
Si	3.16247	-0.62122	-0.53426
C	3.43064	-0.80863	-2.38104
H	3.16037	0.10780	-2.91255
H	4.48581	-1.01455	-2.58413
H	2.84189	-1.62669	-2.80161
C	3.66299	-2.17729	0.38175
H	3.29388	-3.09163	-0.08213
H	4.75523	-2.22689	0.42019
H	3.28594	-2.14824	1.40778
C	4.24384	0.77533	0.10746
H	4.23886	1.66562	-0.52212
H	3.94870	1.07212	1.11763
H	5.27272	0.40616	0.15366

TS for the deprotonation of the *o*-tolyl ring of substrate **67** by pentavalent silicate **25b**

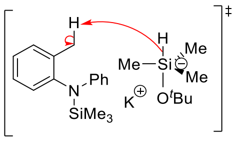

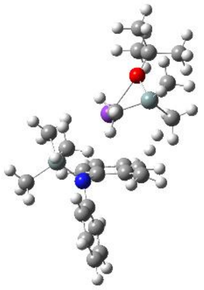

C	-1.44608	1.39907	1.90918
C	-1.52459	0.88081	0.61796
C	-0.85354	1.52402	-0.47665
C	-0.14725	2.71430	-0.13740
C	-0.07367	3.21165	1.15497
C	-0.70381	2.54365	2.21051
H	-2.02798	0.91547	2.68672
H	0.32774	3.26788	-0.94006

H	0.46465	4.13557	1.34005
H	-0.67944	2.93589	3.21948
C	-0.87643	1.03144	-1.82931
H	-1.70227	0.36901	-2.07415
H	-0.74545	1.81467	-2.57649
H	0.25140	0.17850	-2.31092
C	-3.56413	0.08164	-0.38821
C	-4.24369	-0.91816	-1.10074
C	-4.07838	1.38637	-0.43563
C	-5.40501	-0.62825	-1.80752
H	-3.85456	-1.92901	-1.12330
C	-5.23182	1.66898	-1.15625
H	-3.56637	2.18107	0.09288
C	-5.91098	0.66686	-1.84358
H	-5.90656	-1.42351	-2.34729
H	-5.60454	2.68696	-1.17528
H	-6.81046	0.89271	-2.40277
Si	2.57311	-0.46740	-1.39245
C	1.20308	-1.44843	-0.52156
H	0.27759	-0.88606	-0.36769
H	0.93844	-2.27979	-1.17846
H	1.55810	-1.87615	0.42056
C	3.42763	-1.46493	-2.74361
H	2.72446	-2.17672	-3.17926
H	3.77526	-0.81274	-3.54879
H	4.28587	-2.02174	-2.35940
C	2.53399	1.40942	-1.68544
H	2.81100	1.99357	-0.79954
H	1.58222	1.77126	-2.06290
H	3.29424	1.62001	-2.44536
H	0.97523	-0.32165	-2.71795
O	3.62545	-0.48397	0.01015

C	5.02069	-0.25855	0.18403
C	5.70964	-1.61589	0.33732
H	5.60794	-2.20242	-0.57778
H	6.77446	-1.49290	0.55136
H	5.24873	-2.17348	1.15572
C	5.17750	0.54705	1.47518
H	4.72729	0.00525	2.31202
H	6.23067	0.72047	1.70857
H	4.69069	1.52227	1.37258
C	5.64524	0.52168	-0.97629
H	5.20754	1.51832	-1.05746
H	6.71913	0.63405	-0.80874
H	5.50645	-0.00074	-1.92544
K	1.65568	0.73129	1.47348
N	-2.40071	-0.22332	0.35653
Si	-2.24883	-1.81316	1.13273
C	-1.81801	-3.15086	-0.12596
H	-0.97582	-3.75453	0.22269
H	-2.66384	-3.82251	-0.29692
H	-1.53475	-2.70680	-1.08297
C	-0.91122	-1.77957	2.45126
H	0.07513	-1.56464	2.04080
H	-1.12298	-1.08554	3.26520
H	-0.86278	-2.78772	2.87589
C	-3.86456	-2.22690	1.99099
H	-4.70467	-2.28688	1.29685
H	-3.77551	-3.19237	2.49779
H	-4.10049	-1.46909	2.74329

5-*exo*-trig cyclisation TS of intermediate **67**

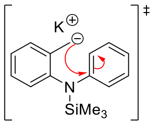

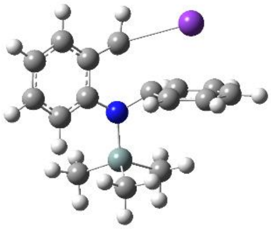

C	-1.50150	0.67994	-0.17248
C	-0.96888	1.93801	-0.54822
C	-1.68563	3.09228	-0.23371
C	-2.93735	3.01685	0.38228
C	-3.47641	1.77587	0.69437
C	-2.75273	0.60629	0.43537
C	0.35006	1.83771	-1.15449
H	-1.27550	4.05923	-0.50931
H	-3.49055	3.92296	0.60165
H	-4.45053	1.70437	1.16414
H	-3.17413	-0.34334	0.73828
H	0.34749	1.31155	-2.11110
C	0.79321	-0.06091	-0.18772
C	1.78025	-0.68317	-1.02988
C	1.16246	0.00734	1.20430
C	3.02764	-1.04094	-0.56328
H	1.51560	-0.86207	-2.06705
C	2.41435	-0.37674	1.65953
H	0.40797	0.35066	1.90615
C	3.39314	-0.87585	0.78837
H	3.73327	-1.48928	-1.25631
H	2.62592	-0.31242	2.72302
H	4.35458	-1.21123	1.15423
N	-0.62898	-0.39703	-0.42956
Si	-1.13431	-2.07546	-0.14220

C	0.18619	-3.29057	-0.68034
H	0.46821	-3.16612	-1.72765
H	1.08865	-3.21338	-0.07110
H	-0.22722	-4.29730	-0.55686
C	-1.45925	-2.42386	1.68042
H	-1.85826	-3.43600	1.79974
H	-0.51677	-2.36781	2.23237
H	-2.15958	-1.72745	2.14435
C	-2.65998	-2.40411	-1.18622
H	-2.94608	-3.45519	-1.08423
H	-3.52371	-1.79334	-0.92253
H	-2.42881	-2.22117	-2.23912
H	0.85094	2.80971	-1.25106
K	2.99727	1.93603	-0.11577

6-aryl cyclisation TS of intermediate **67**

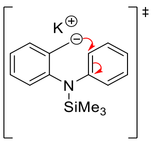

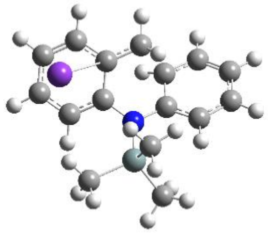

C	-3.14167	-1.79772	-0.92343
C	-2.10725	-2.28738	-0.14310
C	-0.94284	-1.53471	0.15229
C	-0.77674	-0.28143	-0.55560
C	-1.82873	0.17795	-1.36450
C	-3.01830	-0.52797	-1.51583
C	0.17388	-2.11265	0.84856
C	1.63231	-0.36208	-0.32643
C	1.49123	-1.79845	-0.52872
C	2.70688	-2.56963	-0.40752

H	2.68558	-3.60878	-0.71798
C	3.83722	-2.04494	0.17980
C	3.89343	-0.69029	0.53925
C	2.78858	0.12835	0.25975
H	0.68881	-1.52510	1.60127
H	-4.02877	-2.39780	-1.09157
H	-2.19333	-3.27325	0.30485
H	-1.68465	1.08531	-1.93562
H	-3.80005	-0.13610	-2.15523
H	4.70819	-2.67708	0.32520
H	4.79240	-0.25945	0.96127
H	2.87526	1.17789	0.51319
H	0.03958	-3.15348	1.14642
H	0.83661	-2.08739	-1.35168
N	0.44593	0.42345	-0.49321
Si	0.50960	2.16206	-0.11373
C	1.94253	2.95816	-1.03488
H	2.72965	3.30314	-0.36077
H	2.39020	2.24586	-1.73133
H	1.58695	3.82031	-1.60468
C	-1.04383	3.10516	-0.60015
H	-1.16683	3.19283	-1.68142
H	-1.96946	2.70734	-0.17998
H	-0.90820	4.11793	-0.20470
C	0.64815	2.37232	1.75420
H	1.44886	1.76908	2.18485
H	0.83124	3.42052	2.00893
H	-0.28855	2.07768	2.23857
K	-3.01887	0.02944	1.51436

Hydrogen atom abstraction from substrate **68** by a Me_3Si radical (**TS18**)

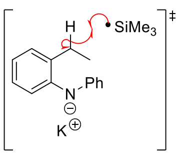

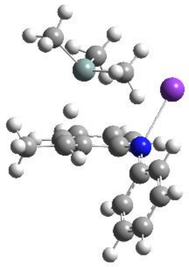

C	-0.30775	2.22739	-1.47691
C	0.13974	1.30443	-0.51007
C	-0.37354	1.45013	0.82461
C	-1.27810	2.48898	1.08931
C	-1.70830	3.37679	0.10610
C	-1.22164	3.23895	-1.18886
H	0.11605	2.15250	-2.47526
H	-1.66854	2.59994	2.09482
H	-2.40981	4.16478	0.35300
H	-1.53068	3.92634	-1.96875
C	-0.10343	0.43029	1.83709
H	0.84033	-0.09391	1.73472
H	-0.95736	-0.62818	1.45394
C	2.11044	-0.05306	-0.37111
C	2.75160	-1.27884	-0.69214
C	2.83062	0.81733	0.49000
C	3.99670	-1.61121	-0.18262
H	2.24172	-1.96293	-1.36443
C	4.07774	0.47181	0.99258
H	2.39227	1.77448	0.75233
C	4.68089	-0.74405	0.67194
H	4.44378	-2.56149	-0.45721
H	4.59141	1.17043	1.64574
H	5.65381	-1.00547	1.06886
Si	-2.04368	-1.65278	0.65189

C	-3.02710	-0.47846	-0.46590
H	-3.84789	-0.04739	0.11416
H	-2.43399	0.37115	-0.81962
H	-3.47803	-1.00101	-1.31817
C	-0.96777	-2.82505	-0.37203
H	-0.67720	-3.68448	0.23749
H	-1.49526	-3.21755	-1.24953
H	-0.04324	-2.32654	-0.67830
C	-3.26144	-2.66492	1.67431
H	-3.91248	-3.25599	1.02136
H	-3.89416	-2.01715	2.28513
H	-2.73724	-3.35386	2.34028
N	0.90573	0.23287	-0.93822
K	-0.71715	-0.65017	-2.75603
C	-0.55298	0.64429	3.26165
H	-1.64049	0.75213	3.33048
H	-0.26519	-0.20679	3.88104
H	-0.10448	1.54447	3.69578

5-*exo*-trig cyclisation TS of intermediate **79** (**TS19**)

TS19

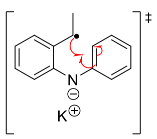

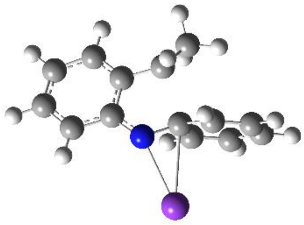

C	1.42188	0.69079	-0.31312
C	1.74166	-0.70171	-0.28060
C	2.99621	-1.13976	0.11885
C	4.00841	-0.21630	0.39885
C	3.73410	1.14816	0.26738
C	2.47126	1.60918	-0.08495
C	0.61107	-1.52337	-0.72864

H	3.20250	-2.20541	0.16699
H	4.99616	-0.55328	0.68792
H	4.52156	1.86922	0.46285
H	2.26988	2.67367	-0.14587
H	0.40819	-1.44566	-1.79679
C	-0.76254	-0.06828	-0.27430
C	-1.85306	-0.29493	-1.18586
C	-1.06486	-0.38188	1.10880
C	-3.01856	-0.93298	-0.79575
H	-1.71035	0.00751	-2.21889
C	-2.22717	-1.02469	1.47675
H	-0.30673	-0.14522	1.85030
C	-3.22179	-1.32706	0.53142
H	-3.79220	-1.11846	-1.53424
H	-2.38521	-1.27895	2.52040
H	-4.13630	-1.82410	0.82905
N	0.12981	1.02030	-0.53783
K	-1.87317	2.55954	0.11227
C	0.31408	-2.85837	-0.12077
H	-0.70630	-3.17151	-0.36161
H	0.99557	-3.62871	-0.50272
H	0.42054	-2.82513	0.96591

Ring-opening of intermediate **80** (**TS20**)

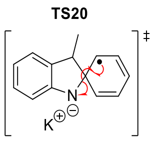

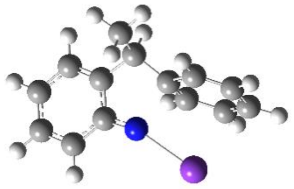

C	1.36819	0.69644	-0.21492
C	1.80263	-0.60809	0.15441
C	3.10760	-0.86083	0.52763

C	4.05791	0.16815	0.52565
C	3.65993	1.44773	0.13517
C	2.34480	1.72803	-0.21575
C	0.70113	-1.62072	-0.04308
H	3.39894	-1.86972	0.80880
H	5.08179	-0.02613	0.81941
H	4.38870	2.25216	0.12196
H	2.05279	2.73369	-0.49816
H	0.72442	-2.37647	0.74945
C	-0.63053	-0.87039	0.09143
C	-1.75950	-1.24997	-0.71351
C	-0.95534	-0.35485	1.39398
C	-3.05848	-1.01412	-0.30701
H	-1.58411	-1.71615	-1.67524
C	-2.26205	-0.12036	1.78857
H	-0.13385	-0.13500	2.06829
C	-3.33525	-0.43839	0.94641
H	-3.87859	-1.30907	-0.95372
H	-2.45354	0.28989	2.77442
H	-4.35773	-0.28959	1.27174
N	0.07762	0.85838	-0.55055
K	-2.21818	1.91904	-0.65835
C	0.88234	-2.32469	-1.39208
H	1.87285	-2.78113	-1.42908
H	0.13935	-3.11035	-1.54870
H	0.81089	-1.60014	-2.20798

6-aryl cyclisation TS of intermediate **79** (**TS21**)

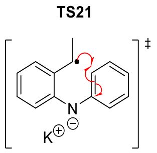

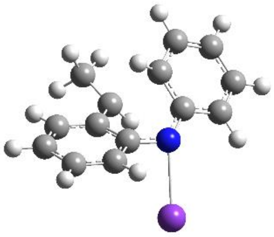

C	-3.39310	0.51601	-0.91438
C	-2.33502	-0.38713	-1.00936
C	-1.07082	-0.11960	-0.44820
C	-0.91576	1.11165	0.28199
C	-1.97695	2.01758	0.31499
C	-3.21544	1.73308	-0.26532
C	0.37494	1.39441	0.90628
C	1.59014	0.72141	-0.74084
C	1.21654	-0.65661	-0.51219
C	2.24872	-1.55967	-0.18357
H	1.98238	-2.60114	-0.02779
C	3.56245	-1.14078	-0.02202
C	3.91914	0.20303	-0.22538
C	2.94888	1.10062	-0.63769
H	-4.34465	0.27714	-1.37697
H	-2.44988	-1.29748	-1.59285
H	-1.84410	2.95718	0.84234
H	-4.02381	2.45172	-0.20545
H	4.32272	-1.86328	0.25499
H	4.94953	0.51819	-0.11084
H	3.22328	2.12296	-0.87813
N	-0.07749	-1.07217	-0.52886
C	0.76781	2.79944	1.24195
H	0.57797	3.47015	0.39830
H	1.83087	2.84894	1.48818

H	0.76551	0.62321	1.56602
H	0.93663	1.33336	-1.35456
H	0.21127	3.19137	2.10368
K	-1.46971	-2.78568	0.83566

Deprotonation of intermediate **81** by KO*t*Bu (**TS22**)

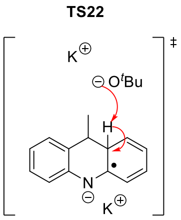

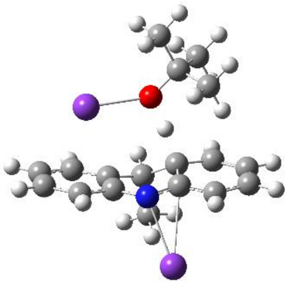

C	-1.40774	-3.62277	0.80434
C	-0.99885	-2.49011	1.52354
C	-0.99365	-1.22010	0.96623
C	-1.40318	-1.02595	-0.39493
C	-1.85178	-2.18306	-1.09462
C	-1.85392	-3.44203	-0.50911
C	-0.62943	0.00721	1.76360
C	-0.72905	1.22018	-0.43234
C	0.02654	1.02638	0.82271
C	0.48091	2.27217	1.45670
H	0.90810	2.20243	2.45524
C	0.45983	3.48386	0.81183
C	-0.09787	3.59933	-0.48220
C	-0.70611	2.46236	-1.06646
H	-1.40992	-4.60257	1.26541
H	-0.67583	-2.59865	2.55642
H	-2.17713	-2.05500	-2.12230
H	-2.19970	-4.29488	-1.08480
H	0.87509	4.36182	1.29773
H	-0.08692	4.54645	-1.00794

H	-1.11667	2.52805	-2.07380
H	0.09438	-0.27304	2.53923
H	1.12621	0.32549	0.42991
C	3.24082	0.24729	-0.27071
C	4.22645	-0.73185	-0.92143
H	4.46553	-1.54047	-0.22368
H	3.78301	-1.16522	-1.82450
H	5.15968	-0.23759	-1.20861
C	2.88833	1.35564	-1.27330
H	2.41066	0.91782	-2.15533
H	2.18722	2.06434	-0.82473
H	3.78170	1.90072	-1.59466
C	3.88998	0.86992	0.97290
H	3.18638	1.56240	1.44207
H	4.13809	0.08493	1.69304
H	4.80393	1.41747	0.72224
O	2.08752	-0.46417	0.09395
K	1.18309	-2.64745	-0.65678
N	-1.39478	0.17384	-1.03282
K	-3.58051	1.63939	-1.05330
C	-1.88145	0.56643	2.45946
H	-2.32118	-0.16576	3.14248
H	-1.63692	1.47469	3.01654
H	-2.64018	0.82670	1.71260

Hydrogen atom abstraction from substrate **52** by a Me_3Si radical (TS23)

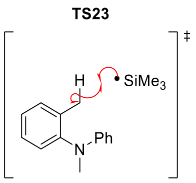

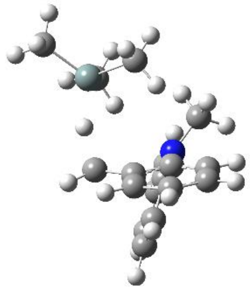

C	-0.05897	2.53371	1.17647
C	-0.09280	1.39776	0.37012
C	0.51263	1.41692	-0.91032
C	1.15536	2.60445	-1.30655
C	1.20135	3.72353	-0.48917
C	0.59006	3.69278	0.76253
H	-0.54608	2.50268	2.14507
H	1.63199	2.62986	-2.28097
H	1.70995	4.61881	-0.82688
H	0.60930	4.56261	1.40771
C	0.56712	0.24264	-1.76510
H	-0.21984	-0.49765	-1.67201
H	0.89240	0.41856	-2.78690
H	1.66507	-0.57051	-1.30185
C	-1.89426	-0.26269	0.31867
C	-2.49585	-1.42965	0.82159
C	-2.53814	0.40967	-0.73543
C	-3.68191	-1.90822	0.27584
H	-2.04054	-1.97196	1.63946
C	-3.72082	-0.08276	-1.26998
H	-2.11235	1.32308	-1.13042
C	-4.30537	-1.24578	-0.77521
H	-4.11960	-2.81181	0.68475
H	-4.19411	0.45935	-2.08082
H	-5.22925	-1.62216	-1.19603

Si	2.48713	-1.58966	-0.25206
C	3.01157	-0.46869	1.16881
H	4.00506	-0.05782	0.97205
H	2.32388	0.37197	1.28308
H	3.05029	-1.01551	2.11594
C	1.14851	-2.81720	0.24255
H	1.06900	-3.61003	-0.50575
H	1.36570	-3.28452	1.20777
H	0.17610	-2.32166	0.30710
C	3.97941	-2.49896	-0.95312
H	4.41738	-3.14791	-0.18765
H	4.75051	-1.79924	-1.28232
H	3.69796	-3.12424	-1.80310
N	-0.69295	0.20203	0.86393
C	-0.37991	-0.19420	2.23460
H	-1.22938	-0.02907	2.90725
H	-0.09852	-1.24954	2.28159
H	0.46800	0.38784	2.58646

5-*exo*-trig cyclisation TS of intermediate **70** (**TS24**)

TS24

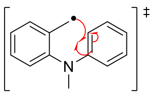

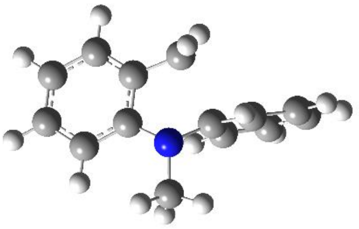

C	1.28171	0.49365	-0.21627
C	1.27170	-0.88949	-0.48641
C	2.40459	-1.64796	-0.23137
C	3.56862	-1.03512	0.24261
C	3.57994	0.33451	0.47055
C	2.43716	1.11016	0.25700
C	-0.01595	-1.36735	-1.01282

H	2.38629	-2.71550	-0.42182
H	4.45594	-1.62733	0.42923
H	4.47949	0.81291	0.84030
H	2.45508	2.16976	0.47785
H	-0.43702	-2.29649	-0.64194
H	-0.23125	-1.14235	-2.05375
C	-1.08845	0.25598	-0.19273
C	-2.21285	0.37744	-1.05349
C	-1.32709	-0.19282	1.14148
C	-3.43765	-0.14307	-0.68334
H	-2.06657	0.83684	-2.02486
C	-2.55557	-0.71431	1.49445
H	-0.50368	-0.17604	1.84848
C	-3.61490	-0.71697	0.58006
H	-4.27162	-0.09173	-1.37379
H	-2.70567	-1.10438	2.49470
H	-4.57810	-1.12270	0.86357
N	0.05423	1.12271	-0.44430
C	-0.10762	2.48267	0.05131
H	-1.10566	2.83382	-0.20712
H	0.01945	2.54349	1.14145
H	0.62187	3.13682	-0.42827

Ring-opening of intermediate **88** (**TS25**)

TS25

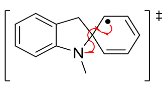

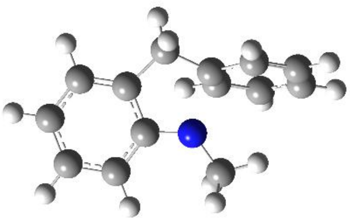

C	1.22296	0.48206	-0.39167
C	1.40675	-0.90352	-0.22655
C	2.62135	-1.40219	0.19774

C	3.67817	-0.52066	0.46354
C	3.49128	0.84847	0.31815
C	2.26056	1.36692	-0.09169
C	0.13604	-1.65773	-0.52714
H	2.75183	-2.47002	0.33923
H	4.63297	-0.90612	0.79948
H	4.30669	1.52891	0.53533
H	2.12182	2.43607	-0.20135
H	0.06236	-2.58224	0.04917
H	0.09446	-1.91763	-1.59040
C	-1.03460	-0.73138	-0.20592
C	-2.20452	-0.75034	-1.03585
C	-1.23427	-0.35321	1.16233
C	-3.40380	-0.24869	-0.58789
H	-2.10736	-1.11967	-2.05086
C	-2.44175	0.15399	1.59803
H	-0.38952	-0.41734	1.84120
C	-3.53477	0.22501	0.72878
H	-4.26149	-0.23425	-1.25060
H	-2.54739	0.48121	2.62600
H	-4.48231	0.62057	1.07307
N	-0.03222	0.80870	-0.87963
C	-0.58625	2.09305	-0.51495
H	-1.66647	2.06556	-0.67500
H	-0.37884	2.38391	0.52213
H	-0.17412	2.85955	-1.18155

6-aryl cyclisation TS of intermediate **70** (**TS26**)

TS26

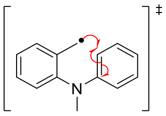

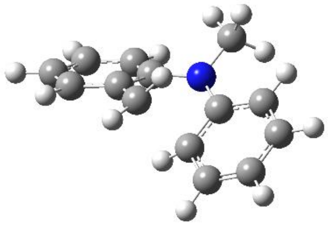

C	-3.62794	-0.94470	-0.05508
C	-2.47118	-1.52585	0.44930
C	-1.26410	-0.81957	0.51682
C	-1.22211	0.49061	-0.02069
C	-2.38340	1.06145	-0.54088
C	-3.58396	0.35921	-0.54103
C	-0.04063	-1.44261	1.00836
C	1.20450	0.51936	-0.18053
C	1.18115	-0.84404	-0.63297
C	2.40267	-1.55052	-0.72325
H	2.39017	-2.54970	-1.14322
C	3.58790	-0.99738	-0.27229
C	3.59403	0.32252	0.18492
C	2.41921	1.07622	0.22177
H	0.54894	-0.92716	1.75799
H	-4.55442	-1.50560	-0.07237
H	-2.49710	-2.54036	0.83267
H	-2.34231	2.05275	-0.97678
H	-4.47462	0.82354	-0.94688
H	4.50887	-1.56612	-0.31080
H	4.52079	0.77908	0.51165
H	2.46292	2.09413	0.58395
H	-0.03118	-2.52574	1.07943
H	0.36624	-1.15884	-1.27379
N	0.00333	1.20139	-0.05270
C	0.03261	2.58895	0.38834

H	0.63836	3.19377	-0.28991
H	-0.97794	2.98590	0.40012
H	0.44537	2.67261	1.40001

Deprotonation of intermediate **90** by KO*t*Bu (**TS27**)

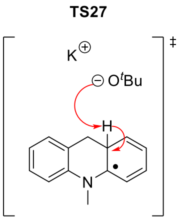

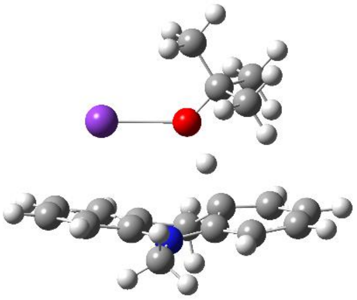

C	3.78540	0.35958	0.99621
C	2.61210	0.07944	1.70722
C	1.56888	-0.64275	1.14660
C	1.67872	-1.10594	-0.18880
C	2.84375	-0.78908	-0.91537
C	3.88749	-0.08267	-0.31825
C	0.31912	-0.99057	1.90293
C	-0.65606	-1.80805	-0.24561
C	-0.87279	-0.96998	0.94536
C	-2.18629	-1.16724	1.57205
H	-2.34544	-0.72162	2.55021
C	-3.21325	-1.81288	0.93554
C	-3.01126	-2.40641	-0.32772
C	-1.70942	-2.41995	-0.88847
H	0.43645	-1.98697	2.35519
H	4.59808	0.90029	1.46524
H	2.50886	0.41627	2.73411
H	2.94239	-1.09168	-1.94928
H	4.78069	0.12488	-0.89685
H	-4.18796	-1.87547	1.40869

H	-3.82288	-2.90036	-0.84591
H	-1.56562	-2.92838	-1.83283
H	0.16515	-0.27904	2.71901
H	-0.83037	0.28442	0.49501
C	-1.83298	2.14520	-0.31543
C	-1.48367	3.46587	-1.01545
H	-0.91858	4.11285	-0.33617
H	-0.87729	3.26955	-1.90656
H	-2.38006	4.00695	-1.33287
C	-2.60508	1.24541	-1.29038
H	-1.98317	1.02670	-2.16398
H	-2.86077	0.29782	-0.80944
H	-3.52814	1.72472	-1.63126
C	-2.70688	2.43991	0.91146
H	-2.95640	1.50311	1.41588
H	-2.15950	3.07683	1.61231
H	-3.63763	2.94420	0.63394
O	-0.63911	1.52274	0.08065
K	1.68332	2.12857	-0.45293
N	0.66360	-1.87980	-0.73424
C	0.93175	-2.66140	-1.92775
H	0.67175	-2.12354	-2.84694
H	1.98428	-2.93540	-1.95616
H	0.35361	-3.58309	-1.88607

Deprotonation of the *o*-tolyl ring of substrate **52** by pentavalent silicate

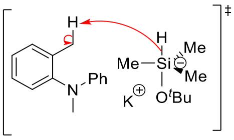

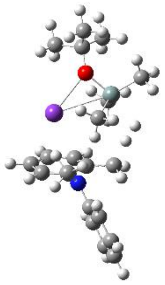

C	1.72578	2.18099	-0.99030
C	1.83854	0.98528	-0.28264
C	1.32912	0.85895	1.05105
C	0.75611	2.04746	1.59037
C	0.65323	3.23180	0.87707
C	1.11788	3.31159	-0.44084
H	2.14838	2.22756	-1.98952
H	0.39873	2.01686	2.61480
H	0.21658	4.10362	1.35301
H	1.06124	4.23577	-1.00216
C	1.35050	-0.36859	1.80093
H	2.11873	-1.08209	1.51702
H	1.30209	-0.22108	2.87994
H	0.17581	-1.28681	1.69073
C	3.75824	-0.51726	-0.45271
C	4.43020	-1.58689	-1.07049
C	4.40297	0.13754	0.61287
C	5.68806	-1.98498	-0.63057
H	3.97337	-2.11876	-1.89389
C	5.65723	-0.27408	1.04049
H	3.91654	0.97001	1.10397
C	6.31526	-1.33790	0.42760
H	6.17826	-2.81448	-1.12776
H	6.12716	0.25114	1.86443
H	7.29454	-1.65191	0.76650

Si	-2.17308	-1.28243	0.57371
C	-0.81327	-1.42375	-0.73940
H	0.12388	-0.94507	-0.45157
H	-0.58189	-2.48718	-0.84691
H	-1.14332	-1.06380	-1.71916
C	-2.94971	-2.93863	1.02352
H	-3.31812	-2.93490	2.05243
H	-3.78364	-3.18486	0.36091
H	-2.20240	-3.72973	0.94483
C	-2.11243	0.02565	1.95680
H	-2.67827	0.93459	1.72057
H	-1.09541	0.29692	2.23377
H	-2.57670	-0.41301	2.84585
H	-0.56087	-1.91612	1.69560
O	-3.29177	-0.47063	-0.50025
C	-4.69496	-0.23512	-0.43514
C	-5.37413	-1.15396	-1.45192
H	-5.20561	-2.20016	-1.18799
H	-6.45188	-0.97452	-1.48612
H	-4.95664	-0.97905	-2.44601
C	-4.91588	1.22668	-0.82911
H	-4.48207	1.41662	-1.81500
H	-5.98017	1.47030	-0.87084
H	-4.44751	1.89123	-0.09593
C	-5.28100	-0.47826	0.95976
H	-4.81946	0.17459	1.70291
H	-6.35373	-0.27075	0.94684
H	-5.14509	-1.51514	1.27212
K	-1.36215	1.52344	-0.69381
N	2.49505	-0.12310	-0.89689
C	2.10814	-0.50087	-2.24958

H	2.88499	-0.24699	-2.98059
H	1.90415	-1.57340	-2.31402
H	1.19196	0.02188	-2.51471

5-*exo*-trig cyclisation of intermediate **74** (**TS28**)

TS28

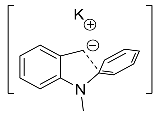

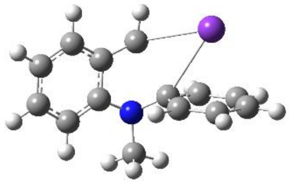

C	1.73909	0.52387	-0.31220
C	1.61240	-0.87531	-0.49273
C	2.64389	-1.69421	-0.03930
C	3.80420	-1.14502	0.51973
C	3.93552	0.23172	0.63421
C	2.89122	1.07716	0.23302
C	0.32658	-1.25907	-1.06018
H	2.55643	-2.77031	-0.15541
H	4.60586	-1.79668	0.84843
H	4.83763	0.66158	1.05394
H	2.98550	2.14754	0.36980
H	0.16698	-0.87852	-2.07114
C	-0.66035	0.54485	-0.28590
C	-1.81498	0.83223	-1.08768
C	-0.91276	0.48984	1.12985
C	-3.08941	0.87023	-0.55122
H	-1.66013	1.01353	-2.14661
C	-2.19412	0.53494	1.65025
H	-0.05678	0.40961	1.79401
C	-3.31985	0.68674	0.82349
H	-3.92783	1.07057	-1.21149
H	-2.32498	0.47772	2.72690

H	-4.31560	0.76394	1.23983
N	0.58954	1.22342	-0.70230
H	0.14701	-2.34089	-1.01570
K	-2.21317	-1.95730	-0.00197
C	0.57351	2.64891	-0.40451
H	0.68095	2.85893	0.66970
H	1.38943	3.13576	-0.94167
H	-0.37073	3.07078	-0.74804

Ring-opening of intermediate **92** (**TS29**)

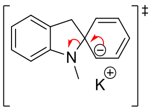

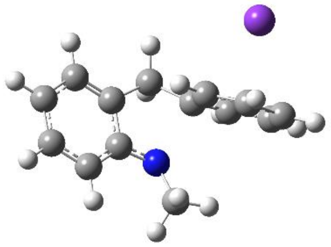

C	1.97483	0.53763	-0.31871
C	1.69176	-0.84471	-0.54192
C	2.58158	-1.83173	-0.17748
C	3.80104	-1.49594	0.43501
C	4.07283	-0.15595	0.69956
C	3.17663	0.85660	0.35593
C	0.31517	-1.01840	-1.11631
H	2.33051	-2.87557	-0.34830
H	4.50569	-2.26841	0.71759
H	5.00272	0.11064	1.19282
H	3.41433	1.89094	0.57916
H	0.31689	-0.82467	-2.19534
C	-0.62290	0.00846	-0.47577
C	-1.70596	0.55194	-1.22866
C	-0.82048	-0.03275	0.93929
C	-2.74760	1.23151	-0.61216

H	-1.64850	0.52123	-2.31215
C	-1.86040	0.65660	1.54495
H	-0.07073	-0.52914	1.54852
C	-2.84503	1.30416	0.78406
H	-3.51027	1.69933	-1.22768
H	-1.92464	0.67074	2.62895
H	-3.65243	1.84627	1.25997
N	0.98777	1.35187	-0.73150
H	-0.05593	-2.03840	-0.95693
K	-3.25542	-1.65179	0.17958
C	0.91737	2.66651	-0.15264
H	0.98469	2.65306	0.94952
H	1.71279	3.33779	-0.51168
H	-0.03954	3.12343	-0.42680

6-aryl cyclisation of intermediate **74** (**TS30**)

TS30

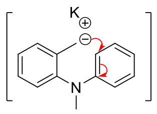

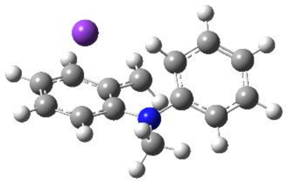

C	3.25586	-0.42799	-1.00305
C	2.05917	-1.04078	-1.36148
C	0.79431	-0.44067	-1.17453
C	0.77114	0.79409	-0.40574
C	1.99492	1.41908	-0.08798
C	3.22361	0.83260	-0.39135
C	-0.42419	-1.09974	-1.53426
C	-1.59889	0.55470	0.12399
C	-1.39608	-0.87160	0.23220
C	-2.58518	-1.66069	0.40582
H	-2.47760	-2.71375	0.64336

C	-3.83721	-1.13044	0.17950
C	-4.00074	0.23716	-0.09724
C	-2.87088	1.05866	-0.11114
H	-1.18975	-0.52823	-2.04537
H	4.20134	-0.90231	-1.24042
H	2.08541	-1.99165	-1.88575
H	1.99502	2.37779	0.41484
H	4.14119	1.35819	-0.15470
H	-4.70947	-1.77298	0.24911
H	-4.98411	0.65984	-0.25851
H	-2.99383	2.11465	-0.32861
H	-0.30231	-2.10514	-1.93851
H	-0.52610	-1.19171	0.80598
N	-0.43843	1.35854	-0.03036
K	2.07617	-1.02945	1.69594
C	-0.43547	2.70383	0.51571
H	0.08202	2.74568	1.48335
H	0.05577	3.39836	-0.17005
H	-1.45812	3.03176	0.67220

Hydrogen atom subtraction from intermediate **78** by Me_3Si radical **24b** (**TS31**)

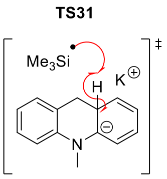

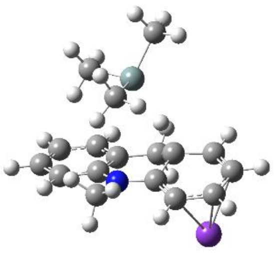

C	2.65510	2.90097	-1.02304
C	1.68601	2.10490	-1.62899
C	0.72857	1.41485	-0.89793
C	0.74619	1.48684	0.51488
C	1.70626	2.31987	1.12167

C	2.64660	3.00657	0.36111
C	-0.30965	0.58452	-1.60772
C	-1.19643	0.00461	0.68405
C	-0.90933	-0.47919	-0.68949
C	-2.02758	-1.25531	-1.27287
H	-1.90394	-1.61238	-2.29135
C	-3.08743	-1.70740	-0.51225
C	-3.27439	-1.29794	0.81863
C	-2.29731	-0.42467	1.38766
H	-1.09465	1.24034	-2.01283
H	3.39121	3.42636	-1.61840
H	1.67019	2.00541	-2.71050
H	1.72405	2.45037	2.19383
H	3.37423	3.63083	0.86725
H	-3.81653	-2.36967	-0.97247
H	-4.09744	-1.66286	1.41758
H	-2.44189	-0.09121	2.40941
H	0.14780	0.08766	-2.47188
H	0.10618	-1.20744	-0.57963
Si	1.85332	-2.06966	-0.21658
C	3.38298	-0.97284	-0.48166
H	4.25906	-1.41544	0.00805
H	3.61153	-0.85869	-1.54483
H	3.22499	0.02727	-0.06699
C	2.29316	-3.80268	-0.87066
H	1.47612	-4.50850	-0.70090
H	2.50073	-3.78263	-1.94346
H	3.18433	-4.18758	-0.35995
C	1.64543	-2.24103	1.66589
H	0.71777	-2.75989	1.92359
H	2.48160	-2.79739	2.10510

H	1.61435	-1.25127	2.12918
N	-0.16239	0.75931	1.28213
C	-0.07993	0.82166	2.72818
H	0.96289	0.83415	3.04395
H	-0.58144	1.70459	3.14432
H	-0.53147	-0.07517	3.14664
K	-3.71784	1.16057	-0.67596
